# Multi-Omics and Experimental Validation Reveal the Protective Effect of Paeoniflorin Against Coronary Heart Disease in Mice via Inhibiting the C3-Cfd-C3aR Pathway

**DOI:** 10.3390/ijms27146236

**Published:** 2026-07-13

**Authors:** Ying Yang, Xiang Li, Wenjing Zong, Sijia Wu, Yingying Li, Danli Tang, Huamin Zhang

**Affiliations:** 1 Institute of Basic Theory for Chinese Medicine, China Academy of Chinese Medical Sciences, Beijing 100700, China; yangyingwac@163.com (Y.Y.); lx15277912206@163.com (X.L.);; 2 Institute of Chinese Materia Medica, China Academy of Chinese Medical Sciences, Beijing 100700, China; 3 Experimental Research Centre of China Academy of Chinese Medical Sciences, Beijing 100700, China

**Keywords:** coronary heart disease, multiomics, paeoniflorin, complement system, traditional Chinese medicine

## Abstract

Coronary heart disease (CHD) is a global cardiovascular disease with high morbidity and mortality, and its complex pathological mechanism poses great challenges to clinical prevention and treatment. Paeoniflorin (PA), a monoterpene glycoside active ingredient from Ranunculaceae plants, has shown potential in cardiovascular protection, but its specific anti-CHD molecular targets and systematic regulatory networks remain unclear. In this study, a mouse model of CHD was established, and a multi-omics strategy combining label-free quantitative proteomics and metabolomics was adopted to explore the mechanism of PA in treating CHD. The results showed that PA significantly improved cardiac function, alleviated myocardial pathological injury and fibrosis, and regulated lipid metabolism in CHD model mice, with the high-dose group showing the optimal effect. Proteomic analysis identified 51 key differentially expressed proteins (DEPs) reversed by PA, which were mainly enriched in complement and coagulation cascades, and neutrophil extracellular trap formation pathways, with the C3-Cfd-C3aR signaling axis as the core hub. Further verification confirmed that PA could downregulate the expression of C3, Cfd, C3aR, and their downstream molecule BTK, thereby inhibiting myocardial inflammatory response and cardiomyocyte apoptosis. In addition, PA downregulated the expression of platelet activation markers ITGA2B/ITGB3. Metabolomic analysis revealed that PA reversed 57 abnormal metabolites in CHD mice, which were enriched in GABAergic synapse, retrograde endocannabinoid signaling and other pathways. Molecular docking confirmed that PA could stably bind to C3, Cfd, C3aR, BTK, and ITGA2B/ITGB3 with strong binding activity. In conclusion, PA exerts anti-CHD effects through a multi-target and multi-pathway synergism, mainly by targeting the C3-Cfd-C3aR axis to inhibit inflammation, apoptosis and platelet activation, and regulating metabolic disorders. This study provides experimental evidence and theoretical support for the clinical application of PA as a multi-target therapeutic drug for CHD.

## 1. Introduction

Coronary heart disease (CHD), also known as coronary atherosclerotic heart disease, is a cardiovascular disease with the highest morbidity and mortality worldwide. The number of cardiovascular disease-related deaths is projected to increase from 20.5 million in 2025 to 35.6 million by 2050 [1], making the prevention and treatment of CHD a major public health challenge that continuously exacerbates the global medical and socioeconomic burden [2,3]. The core pathological basis of CHD is atherosclerosis, a complex pathological network involving multiple links and cell types, including lipid deposition, endothelial injury, chronic inflammation, plaque instability, and thrombosis [4,5,6]. Current pharmacotherapies for CHD target mainly single molecules, which fail to cover the complex pathological mechanisms of atherosclerosis, such as lipid deposition, inflammatory responses, and endothelial damage. Moreover, these therapies are limited by substantial adverse reactions, poor therapeutic response or drug resistance in some patients, and the inability to reverse established lesions. These issues result in a high residual risk, even in patients receiving standard treatment, highlighting the urgent need to develop multi-target and precise therapeutic drugs and technologies [7].

Paeoniflorin (PA), a monoterpene glycoside active ingredient isolated from medicinal plants of the Ranunculaceae family, such as *Paeonia lactiflora* Pall. and *Paeonia veitchii Lynch*, is the core material underlying the pharmacological effects of paeony. Due to its naturally low toxicity and broad pharmacological activity, PA exhibits high translational potential for the prevention and treatment of cardiovascular diseases [8,9]. Domestic and international studies have confirmed that PA exerts myocardial and vascular protective effects through multiple pathways, including regulating lipid metabolism, resisting oxidative stress, inhibiting the release of inflammatory factors, alleviating myocardial ischemia-reperfusion injury, and mitigating myocardial fibrosis [10,11,12]. However, current research on the anti-CHD mechanism of PA still has obvious shortcomings.

To our knowledge, most studies only focus on the preliminary verification of a single pharmacological effect or signaling pathway, lacking systematic target mining and pathway analysis, and the core hub pathways regulating myocardial inflammation, platelet activation, and metabolic disorders remain understudied. Meanwhile, current studies failed to construct a complete regulatory network of “component-target-pathway-phenotype,” leading to insufficient explanation of the integrated mechanism underlying its synergistic multi-target and multi-pathway effects. As a core tool in systems biology, multiomics technology has overcome the limitations of traditional single-omics research [13]. By integrating multidimensional molecular data, such as proteomics and metabolomics, it can comprehensively dissect the complex molecular networks of diseases and accurately screen core targets and key pathways for drug intervention, which has become a pivotal tool for the mechanistic studies of active ingredients from natural medicines [14]. Therefore, in this study, a mouse model of CHD was established [15], and a multi-omics strategy combining label-free quantitative proteomics and metabolomics was adopted to systematically screen differentially expressed proteins (DEPs) and abnormal metabolites after PA intervention and identify potential key targets. Specifically, we aim to identify key target proteins and metabolic pathways modulated by paeoniflorin in coronary heart disease, followed by experimental validation of the role of the C3-Cfd-C3aR-BTK signaling cascade in the mechanisms of inflammation suppression, apoptosis, and platelet activation.

## 2. Results

### 2.1. Paeoniflorin Improves Cardiac Function, Alleviates Myocardial Pathological Injury, and Regulates Lipid Metabolism in CHD Model Mice

**Figure 1:**
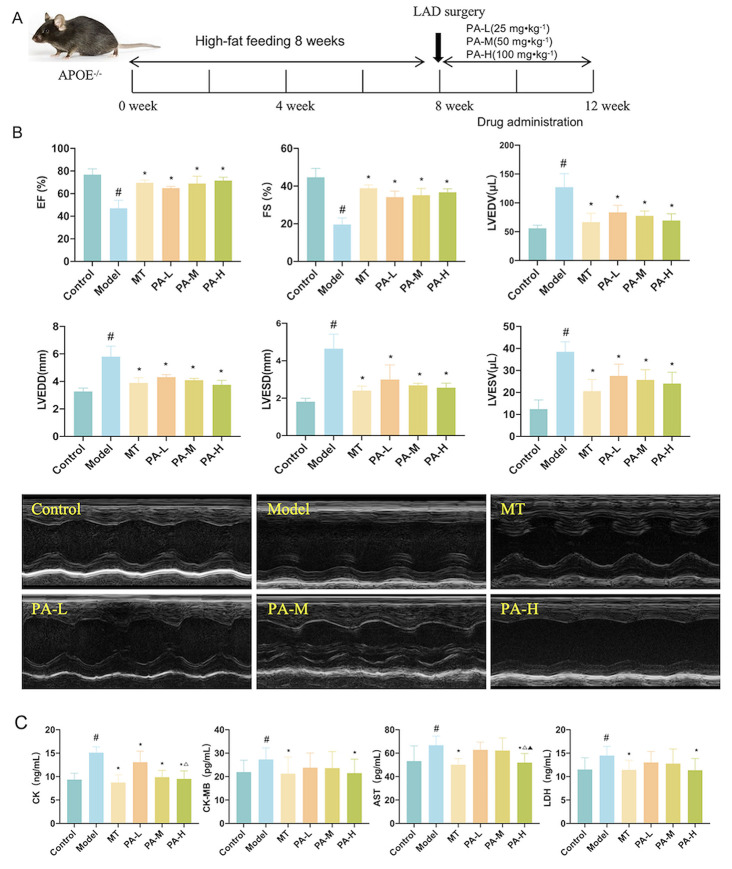
(**A**) Animal flowchart. (**B**) Cardiac function (EF%, FS%, LVESD, LVEDD, LVESV, LVEDV) (*n* = 5). (**C**) The levels of myocardial enzyme (CK, AST, LDH and CK-MB) (*n* = 6). The data are mean ± SD. In comparison to the control group, ^#^ *p* < 0.05. In comparison to the model group, * *p* < 0.05. In comparison to the PA-L group, ^△^ *p* < 0.05. In comparison to the PA-M group ^▲^ *p* < 0.05.

**Figure 2:**
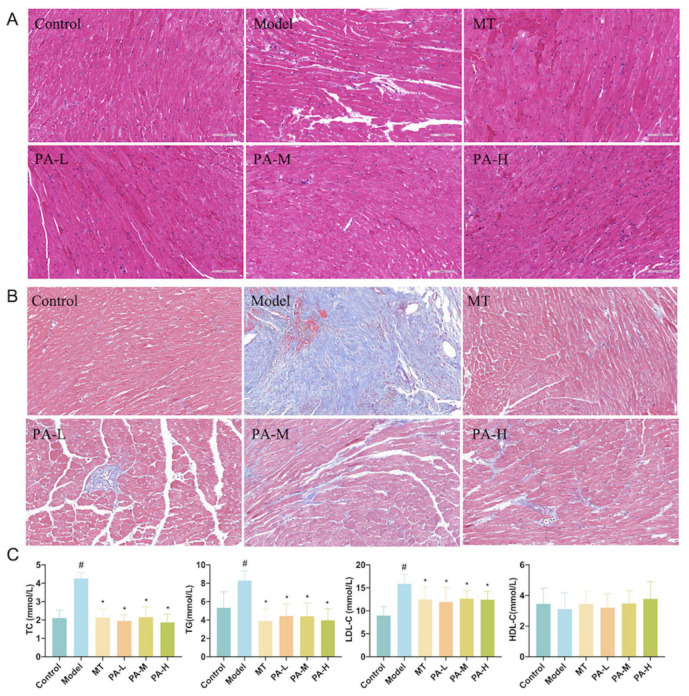
(**A**) H&E (200×) of Myocardium. (**B**) Masson (200×) of Myocardium. (**C**) The levels of blood lipid indicators (TC, TG, HDL-C, LDL-C) (*n* = 6). The data are mean ± SD. In comparison to the control group, ^#^ *p* < 0.05. In comparison to the model group, * *p* < 0.05.

Mice in the model group exhibited significantly impaired cardiac function, decreased left ventricular ejection fraction (EF) and fractional shortening (FS), and significantly increased left ventricular end-systolic/end-diastolic volume and diameter (LVESV/LVEDV and LVESD/LVEDD) (*p* < 0.05). Both metoprolol tartrate (MT) and PA improved cardiac function (Figure 1B). Serum levels of creatine kinase (CK), aspartate aminotransferase (AST), lactate dehydrogenase (LDH), and CK-isoenzyme MB (CK-MB) were significantly elevated in the model group (*p* < 0.05). MT and all doses of PA reduced CK and AST levels (*p* < 0.05) compared with the model group. High-dose PA (PA-H) exerted a more significant reducing effect on CK levels (*p* < 0.05) than low-dose PA (PA-L). PA-H caused a more significant reduction in AST levels (*p* < 0.05) than PA-L and medium-dose PA (PA-M). Whereas, MT and PA-H significantly decreased LDH and CK-MB levels (*p* < 0.05) compared with the model group (Figure 1C). Myocardial hematoxylin-eosin (HE) staining showed a disordered arrangement of myocardial fibers accompanied by necrosis and inflammatory infiltration in the model group; whereas, MT and PA ameliorated fiber arrangement and reduced inflammation (Figure 2A). Masson staining revealed that the myocardial tissues of the control group were mainly composed of red muscle fibers with intact structure and few blue collagen fibers; whereas, in the model group, a large amount of dark blue collagen deposition was observed in the myocardial interstitium, with broken and disordered myocardial fibers and a substantially increased degree of fibrosis. Collagen deposition in the myocardial tissues of mice in the PA dose and MT groups was considerably reduced compared with that in the model group, with a relatively intact myocardial structure and markedly decreased degree of fibrosis, indicating that PA can alleviate fibrosis in model mice (Figure 2B). Serum total cholesterol (TC), triglycerides (TG), and low-density lipoprotein cholesterol (LDL-C) levels increased in the model group (*p* < 0.05); whereas, MT and all doses of PA reduced TC, TG, and LDL-C levels (*p* < 0.05) (Figure 2C). In summary, PA can improve cardiac function and myocardial pathological injury, and regulate blood lipid and myocardial enzyme levels in CHD model mice, with high-dose PA showing the optimal effect. Therefore, samples from the high-dose PA group were selected for subsequent multi-omics analyses.

### 2.2. Proteomic Screening of Core Targets and Pathways of Paeoniflorin in CHD

**Figure 3:**
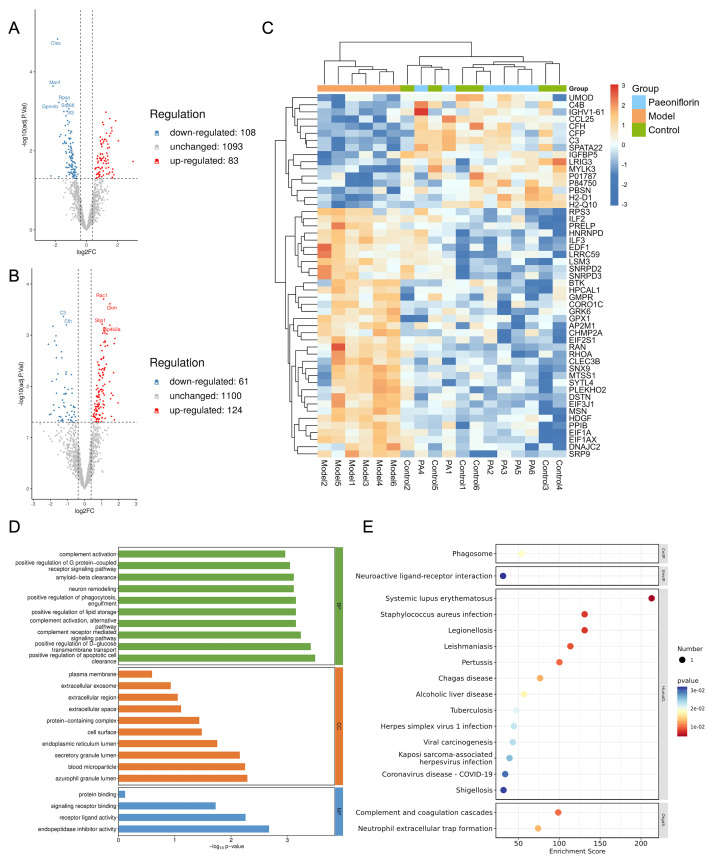
(**A**,**B**) Differential protein volcano plots of the Control group vs. the Model group and the Model group vs. the Paeoniflorin group. (**C**) Heatmap of differential proteins reversed by Paeoniflorin. (**D**) GO enrichment analysis of DEPs reversed by paeoniflorin intervention. (**E**) KEGG enrichment analysis of DEPs reversed by paeoniflorin intervention.

To further explore the core molecular targets and potential action pathways of PA in CHD intervention, label-free quantitative proteomic sequencing analysis was performed on plasma samples from mice in each group. All screened DEPs are listed in Supplementary Table S1. Screening of DEPs revealed 191 DEPs (83 upregulated and 108 downregulated) in the CHD model group compared with the control group (Figure 3A), and 185 DEPs (61 upregulated and 124 downregulated) in the PA-H group compared with the model group (Figure 3B). Intersection analysis of the two sets of DEPs showed that 51 of the 179 abnormally expressed proteins in the model group were substantially reversed in expression trend by PA intervention, suggesting that these 51 proteins are key targets for PA to exert its anti-CHD effects (Figure 3C). Gene Ontology (GO) functional enrichment and Kyoto Encyclopedia of Genes and Genomes (KEGG) pathway enrichment analyses were performed on the reversely expressed key DEPs. GO enrichment results showed that the differentially expressed proteins were mainly substantially enriched in biological processes, such as complement activation, complement receptor-mediated signaling pathway, positive regulation of apoptotic cell process, and regulation of inflammatory response (Figure 3D). KEGG pathway enrichment results indicated that the differentially expressed proteins were mainly enriched in core pathways, including complement and coagulation cascades, and neutrophil extracellular trap formation (Figure 3E). Further pathway correlation analysis revealed that the complement core molecule C3 was highly enriched in all key pathways. Previous studies have confirmed that the C3-complement factor D (Cfd)-complement C3a receptor (C3aR) signaling axis [16,17], as the core pathway of alternative complement activation, can synchronously regulate the homeostasis of left ventricular systolic and diastolic functions and mediate cardiac platelet thrombosis through a unified complement activation cascade, serving as a key molecular hub linking abnormal complement activation, chronic cardiac inflammation, thrombosis, and cardiac dysfunction. Furthermore, Bruton’s tyrosine kinase (BTK), a key non-receptor tyrosine kinase, is the core bridge molecule for the downstream transduction of complement signals that mediate the cardiac inflammatory response, platelet activation, and cardiac dysfunction. Abnormal C3 activation regulates BTK expression through downstream signaling [18], further amplifying the cardiac inflammatory cascade. Comprehensive proteomic enrichment results suggest that PA may exert anti-CHD effects by targeting the C3-Cfd-C3aR signaling axis to inhibit platelet activation, alleviate myocardial inflammatory injury, and improve cardiac function.

### 2.3. Paeoniflorin Inhibits the Activation of the C3-Cfd-C3aR Axis

**Figure 4:**
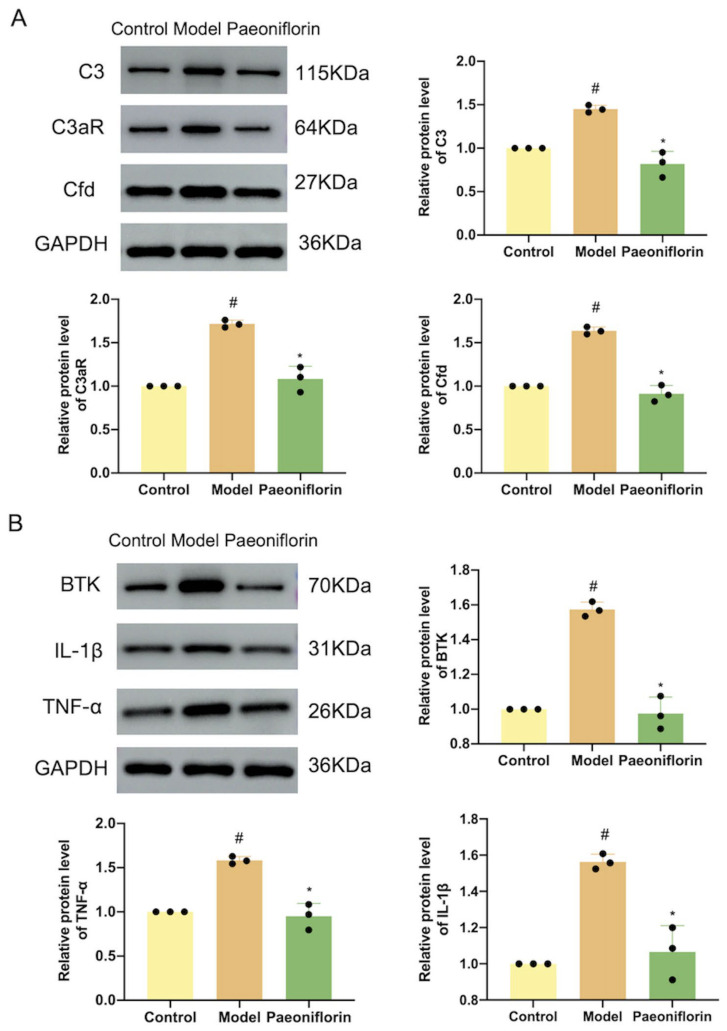
(**A**) The protein expression of C3, Cfd and C3aR (*n* = 3). (**B**) The protein expression of BTK, IL-1β and TNF-α (*n* = 3). All mice were treated with paeoniflorin at 100 mg·kg^−1^. The data are mean ± SD. In comparison to the control group, ^#^ *p* < 0.05. In comparison to the model group, * *p* < 0.05.

Cfd is the rate-limiting enzyme for the activation of the alternative complement pathway, which specifically catalyzes the cleavage of C3 to generate the pro-inflammatory anaphylatoxin C3a. C3a further binds to the specific receptor C3aR on the membranes of cardiomyocytes, vascular endothelial cells, and platelets to initiate downstream pathological signaling cascades, which simultaneously induce cardiomyocyte injury and left ventricular dysfunction, while additionally promoting platelet adhesion, aggregation, and thrombosis and synergistically exacerbating cardiac injury in CHD [16]. Based on the proteomic screening results, the regulatory effect of PA on the C3-Cfd-C3aR axis was verified by measuring protein expression levels. The results showed that the protein expression levels of C3, Cfd, and C3aR were significantly increased in the myocardial tissues of model mice (*p* < 0.05) compared with the control-operated group; whereas, the expression levels of the three proteins were significantly downregulated after PA intervention (*p* < 0.05) (Figure 4A). These results confirm that PA can effectively inhibit the abnormal activation of the C3-Cfd-C3aR signaling axis in CHD model mice by downregulating the protein expression of C3, Cfd, and C3aR.

### 2.4. Paeoniflorin Resists Myocardial Inflammatory Injury and Cardiomyocyte Apoptosis in CHD Mice

**Figure 5:**
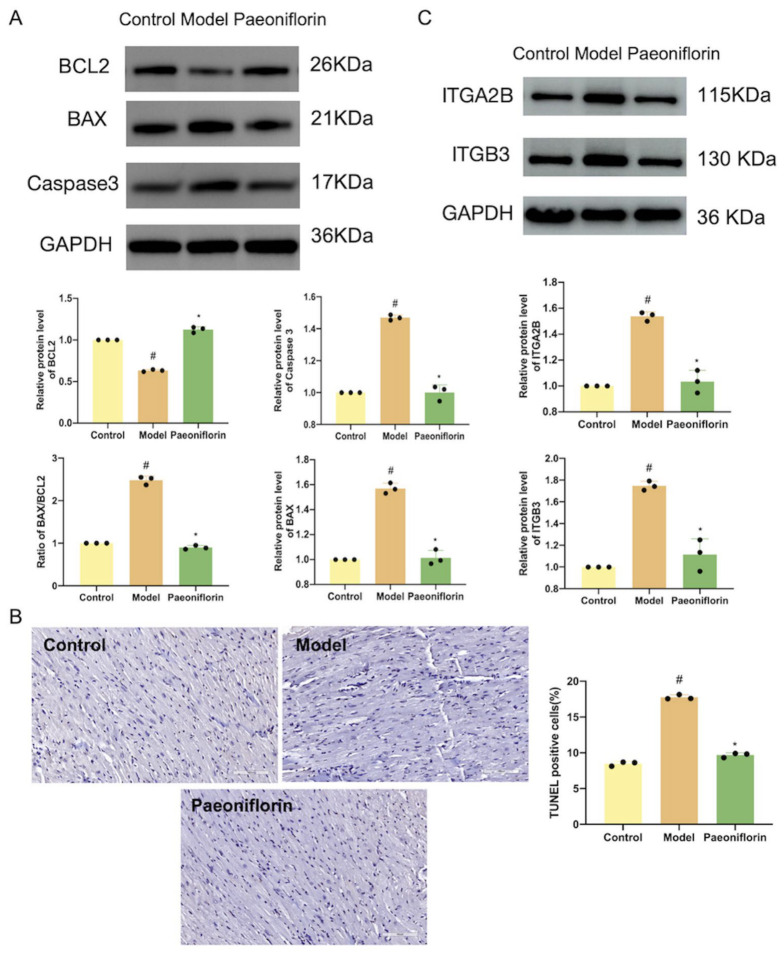
(**A**) The protein expression of BAX, BCL-2 and Caspase-3 (*n* = 3). (**B**) Tunel staining of Myocardium (*n* = 3). (**C**) The protein expression of ITGA2B and ITGB3 (*n* = 3). All mice were treated with paeoniflorin at 100 mg·kg^−1^. The data are mean ± SD. In comparison to the control group, ^#^ *p* < 0.05. In comparison to the model group, * *p* < 0.05.

Previous studies have confirmed that activation of the C3-Cfd-C3aR axis can mediate inflammatory signal transduction through its key downstream molecule BTK, amplify the myocardial inflammatory response, and directly regulate cardiomyocyte apoptosis and fibrosis [19]. Therefore, the expression of downstream inflammation- and apoptosis-related proteins was examined to verify the anti-inflammatory and anti-apoptotic effects of PA. Compared with the control group, the protein expression levels of BTK, pro-inflammatory factors interleukin (IL)-1β, and tumor necrosis factor (TNF)-α in myocardial tissues of model mice were significantly increased (*p* < 0.05); after PA intervention, the expression of BTK, IL-1β, and TNF-α was significantly reduced (*p* < 0.05) (Figure 4B), indicating that PA can block the C3-Cfd-C3aR axis-mediated inflammatory signal transduction by inhibiting BTK expression thus alleviating myocardial inflammatory injury. Furthermore, the expression level of Caspase-3 protein and the Bcl-2-associated X protein (BAX)/B-cell lymphoma (BCL)-2 ratio in the myocardial tissues of model mice were significantly higher than those in the control group (*p* < 0.05). Terminal deoxynucleotidyl transferase dUTP nick-end labeling (TUNEL) assay further confirmed that the cardiomyocyte apoptosis rate was significantly increased in the model group (*p* < 0.05). Compared with the model group, PA intervention significantly reduced the expression level of Caspase-3, the BAX/BCL-2 ratio, and the cardiomyocyte apoptosis rate (*p* < 0.05) (Figure 5A,B). These results indicate that PA can effectively inhibit the myocardial inflammatory response and abnormal cardiomyocyte apoptosis, and alleviate pathological myocardial injury in CHD model mice by regulating the C3-Cfd-C3aR-BTK signaling axis. The specific binding of C3a to C3aR on the platelet membrane is a key mechanism that regulates platelet adhesion, activation, and thrombosis. Integrin αIIbβ3, the most abundant integrin molecule on the platelet membrane, is a heterodimer composed of the αIIb subunit encoded by ITGA2B and the β3 subunit encoded by ITGB3, and is a recognized marker molecule of platelet activation [20]. Therefore, the expression of related proteins was examined to explore the regulatory effects of PA on platelet activation. Compared with the control group, the protein expression levels of ITGA2B and ITGB3 in the myocardial tissues of model mice were significantly increased (*p* < 0.05), and after PA intervention, the expression levels of both were significantly downregulated (*p* < 0.05) (Figure 5C).

### 2.5. Regulatory Effect of Paeoniflorin on Endogenous Metabolites in CHD Mice

**Figure 6:**
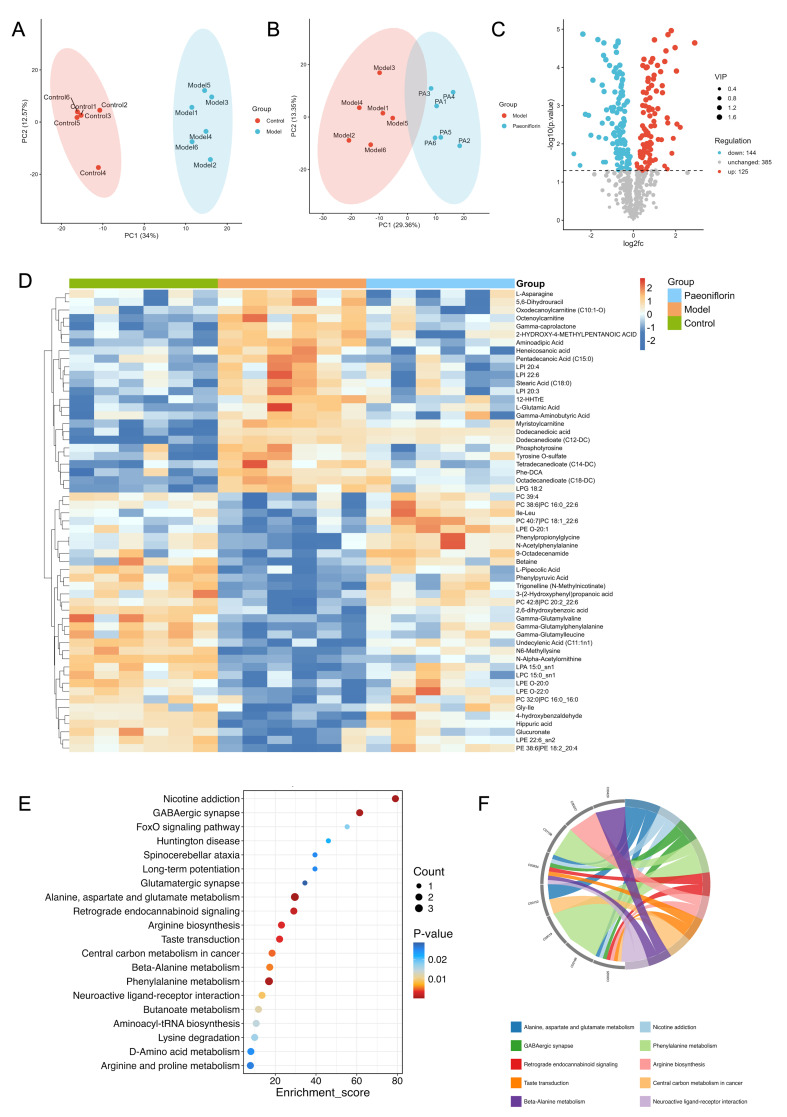
(**A**) Validation of the PCA between control and model groups. (**B**) PCA plot between control and model groups. (**C**) Volcano plots of the Control group vs. the Model group. (**D**) Clustered heatmap of DEMs reversed by paeoniflorin intervention. (**E**) KEGG enrichment analysis of DEMs reversed by paeoniflorin intervention. (**F**) KEGG chord chart of DEMs reversed by paeoniflorin intervention.

Principal component analysis (PCA) showed obvious separation trends between the control and model groups, and between the PA and model groups, indicating significantly different metabolic profiles among the groups (Figure 6A,B). With variable importance in projection (VIP) > 1 and *p* < 0.05 as screening criteria, 269 differential metabolites were identified between the model and control groups, including 144 upregulated and 125 downregulated metabolites in the model group (Figure 6C). Among the 271 differential metabolites, 57 were restored to normal levels following PA treatment (Figure 6D). These metabolites were mainly fatty acids (22.8%) and glycerophospholipids (21.1%) (Table 1). Specifically, various glycerophospholipids in the model group (such as LPC 15:0_sn1, LPE 22:6_sn2, PC 32:0|PC 16:0_16:0, PC 39, PC 40:7|PC 18:1_22:6) were restored to normal levels after PA treatment. Fatty acids such as Myristoylcarnitine, Octenoylcarnitine, and Oxodecanoylcarnitine were also significantly reversed. Further, pathway enrichment analysis showed that the differential metabolites reversed by PA were mainly enriched in the GABAergic synapse, retrograde endocannabinoid signaling, neuroactive ligand-receptor interaction, and phospholipase D signaling pathways (Figure 6E,F).

### 2.6. Molecular Docking Results of Paeoniflorin with C3, Cfd, C3aR, BTK, and ITGA2B/ITGB3

**Figure 7:**
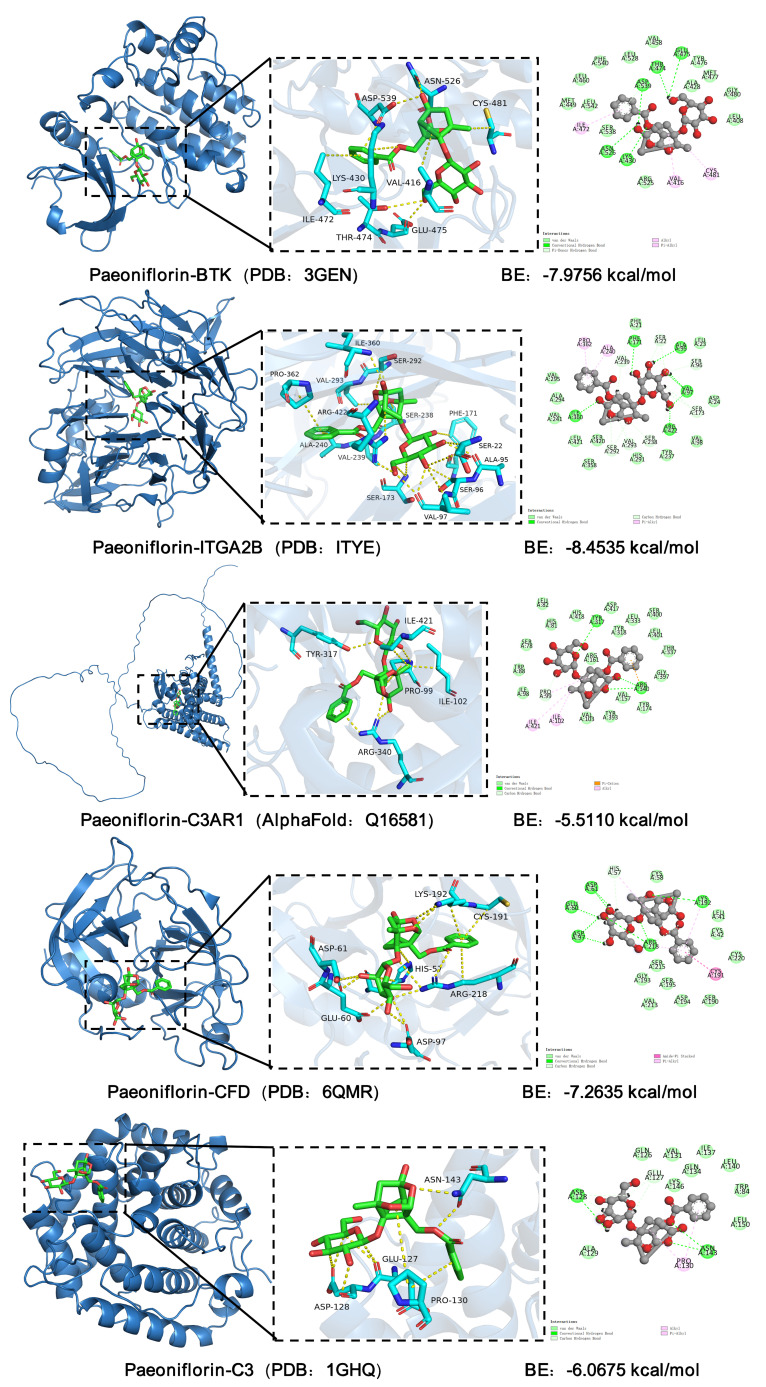
Molecular Docking Results of Paeoniflorin with C3, Cfd, C3aR, BTK, and ITGA2B/ITGB3.

To verify the direct binding activity of PA with the core target proteins screened earlier, molecular docking technology was used to simulate the spatial interaction between PA small molecule and C3, Cfd, C3aR, BTK, and ITGA2B/ITGB3 proteins. The results showed that small molecules of PA accurately docked into the active pocket regions of each target protein and formed stable non-covalent interactions with their amino acid residues, including hydrogen bonds and hydrophobic forces. Quantitative analysis of binding free energy showed that the binding free energies of PA with the above five core target proteins were −7.9756, −6.0675, −5.511, −7.2635 and −8.4535 kcal/mol, respectively (Figure 7), suggesting strong and favorable binding activity between PA and each target protein. These molecular docking results confirmed at the molecular level that PA can regulate the biological functions of target proteins by directly binding to the core targets C3, Cfd, C3aR, BTK, and ITGA2B/ITGB3, thereby exerting anti-CHD effects and providing direct molecular evidence for subsequent mechanistic studies.

## 3. Discussion

CHD is the leading cause of cardiovascular morbidity and mortality worldwide, with a complex pathophysiological process involving multi-cascade reactions, such as myocardial ischemic injury, chronic inflammation, platelet activation, lipid metabolism disorder, and pathological apoptosis of cardiomyocytes. PA is a monoterpene glycoside active compound isolated from paeony. Numerous studies have confirmed that it exerts myocardial protective, anti-inflammatory, antioxidant, and lipid metabolism-regulating effects in various cardiovascular disease models, showing translational potential as a multi-target myocardial protective drug [21,22]. However, the specific molecular targets and systematic regulatory networks of PA in CHD have not been fully elucidated. This study provided new insights into the molecular mechanisms of action of PA in CHD by establishing a mouse model of CHD.

Leakage of myocardial enzymes such as serum CK, AST, LDH, and CK-MB is a reliable marker of cardiomyocyte membrane damage and structural destruction, whereas left ventricular EF and FS are the gold standard indicators for evaluating cardiac systolic function [23]. In this study, the CHD model mice showed significantly decreased EF and FS values, increased LVESV/LVEDV, and elevated serum myocardial enzyme levels, accompanied by a disordered arrangement of myocardial fibers and aggravated interstitial fibrosis, indicating that the model successfully simulated the core pathological phenotype of CHD. After PA intervention, the cardiac systolic function of mice was significantly restored, myocardial enzyme leakage was reduced, and myocardial structural injury and fibrosis were alleviated, which is consistent with previous studies confirming that PA resists myocardial ischemic injury and improves pathological remodeling [24]. Furthermore, PA significantly reduced serum TC, TG, and LDL-C levels in CHD mice and corrected lipid metabolism disorders, which are considered a clear risk factor for coronary atherosclerosis and plaque progression [25]. These results confirm that PA exerts multidimensional myocardial protective effects.

Abnormal activation of the complement system, especially the excessive activation of the alternative pathway, is a key molecular link connecting inflammation, thrombosis, and myocardial injury in cardiovascular diseases [26,27]. Complement C3 is the core molecule of the complement cascade reaction, and can be cleaved into C3a and C3b by C3 convertase, and Cfd is the rate-limiting enzyme for this cleavage process in the alternative complement pathway [16,28]. The anaphylatoxin C3a can bind to the specific receptor C3aR expressed on the surface of cardiomyocytes, vascular endothelial cells, and platelets, triggering pro-inflammatory and pro-apoptotic signaling cascades, disrupting cardiac homeostasis, and promoting pathological remodeling [17]. Increasing evidence indicates that excessive activation of the C3-Cfd-C3aR axis leads to a vicious cycle of cardiac inflammation, platelet aggregation, and cardiomyocyte death, thereby accelerating CHD progression and worsening heart failure prognosis [29,30]. BTK, a non-receptor tyrosine kinase, has recently been confirmed as a key mediator downstream of C3aR signaling, serving as the core bridge for the recruitment of inflammatory cells and the release of cytokines in the injured heart induced by complement activation [19]. In this study, proteomic analysis showed that DEPs in CHD mice were significantly enriched in complement activation and complement receptor-mediated signaling pathways, with C3 as the core hub protein. Subsequent experimental verification confirmed that the expression of C3, Cfd, C3aR, and BTK was significantly upregulated in the myocardial tissues of the CHD model mice, whereas PA intervention significantly downregulated the expression of the four proteins and inhibited the abnormal activation of the C3-Cfd-C3aR-BTK axis. Furthermore, PA reduced the expression of pro-inflammatory factors IL-1β and TNF-α, down-regulated the level of cleaved-Caspase-3 and the BAX/BCL-2 ratio, and decreased TUNEL-positive apoptotic cardiomyocytes, indicating that it can effectively inhibit myocardial inflammation and apoptosis. This result is consistent with previous studies showing that genetic or pharmacological inhibition of Cfd can alleviate myocardial inflammation, reduce cardiomyocyte apoptosis, and improve cardiac function in mice with ischemic heart failure [16]. Notably, to our knowledge, this is the first study to confirm that PA directly targets the C3-Cfd-C3aR-BTK axis to exert anti-inflammatory and anti-apoptotic effects in CHD, thus providing a new molecular explanation for its myocardial protective mechanism.

Excessive platelet activation and subsequent arterial thrombosis are the main triggers of acute coronary events and vascular occlusion in CHD [31]. Integrin αIIbβ3 is the most abundant integrin on the platelet membrane and is a marker molecule of platelet activation that mediates platelet aggregation and stable thrombus formation [32]. Emerging studies have shown that cardiac platelet thrombosis mediated by the C3-Cfd-C3aR axis can obstruct coronary blood flow and exacerbate myocardial ischemia and hypoxia [29]. In this study, ITGA2B and ITGB3 expression was significantly increased in myocardial tissues of the CHD model mice, indicating severe excessive platelet activation; PA intervention significantly down-regulated ITGA2B/ITGB3 expression. These results confirm that PA inhibits coronary thrombosis by blocking C3aR-mediated platelet activation via inhibition of the C3-Cfd-C3aR axis. These findings expand the current understanding of the antithrombotic mechanism of PA and highlight its potential as a dual anti-inflammatory and antiplatelet drug in CHD.

The metabolomic analysis revealed significant metabolic disorders in CHD mice, with differential metabolites mainly comprising glycerophospholipids and fatty acids. After PA intervention, 57 abnormally expressed metabolites were significantly reversed, and pathway enrichment analysis showed that differential metabolites were mainly enriched in glycerophospholipid metabolism, GABAergic synapses, retrograde endocannabinoid signaling, and neuroactive ligand-receptor interaction pathways. The phospholipase D signaling pathway, which is closely related to glycerophospholipid metabolism, regulates platelet activation [33]. PA reverses the abnormal metabolism of various glycerophospholipids, further supporting its inhibitory effects on platelet activation. Importantly, this study found that PA can regulate the GABAergic synapse pathway, a key regulatory pathway for cardiac homeostasis [34,35]. As an important inhibitory neurotransmitter in the central nervous system, GABA reduces blood pressure and slows the heart rate through phasic sympathetic inhibition, constituting an important part of the central cardiovascular regulatory network. Furthermore, endocannabinoids mediate retrograde signaling pathways through CB1/CB2 receptors to regulate cardiac systolic dysfunction [36,37]. The results of this study indicate that PA not only directly inhibits the C3-Cfd-C3aR axis but also restores the homeostasis of the above neuroactive ligand-receptor pathways to exert synergistic myocardial protective effects.

To verify the direct interaction between PA and the predicted core targets, molecular docking was performed with C3, Cfd, C3aR, BTK, and ITGA2B/ITGB3. Molecular docking provides structural evidence that PA directly targets the C3–Cfd–C3aR–BTK axis and platelet integrin, which is consistent with our in vivo experimental findings. PA markedly downregulated C3, Cfd, and C3aR expression. This blockade restrains excessive complement activation and subsequently alleviates myocardial inflammation, cardiomyocyte apoptosis, and platelet overactivation. Meanwhile, PA also produces an indirect, secondary protective effect by remodeling metabolic dysfunction. Metabolomic analysis indicated that PA reversed aberrant metabolites primarily enriched in glycerophospholipid and fatty acid metabolism, thereby restoring metabolic homeostasis. The amelioration of metabolic disorders further relieves myocardial injury as a synergistic regulatory mechanism. In summary, PA mainly protects the myocardium via direct inhibition of the C3–Cfd–C3aR pathway, while the modulation of metabolic profiles serves as an auxiliary and synergistic supporting effect. It further clarifies the direct molecular link between excessive complement activation and myocardial inflammation, cardiomyocyte apoptosis, and aberrant platelet activation, thereby enriching and refining the multi-layer molecular regulatory network underlying the protective effects of PA in CHD.

Nevertheless, several limitations of this study should be acknowledged. The experiments were conducted exclusively in a mouse model, without validation using human clinical samples. The sample size for Western blot validation was limited (*n* = 3 per group), and larger cohorts are required to confirm these findings. Moreover, molecular docking merely predicts potential binding between paeoniflorin and C3-Cfd-C3aR cascade proteins without direct physical interaction evidence. Follow-up SPR, MST, CETSA and inhibitor intervention assays are required to confirm direct binding and axis regulation. Our interpretation of PA-induced antiplatelet activity is only supported by myocardial ITGA2B and ITGB3 expression changes, without complementary platelet functional assays. Further platelet function detection will be conducted in future work to consolidate the above conclusion. Future work will adopt improved animal models, in vitro functional assays and clinical specimen validation to clarify the molecular mechanism of PA and promote its clinical translation.

## 4. Materials and Methods

### 4.1. Establishment and Grouping of Animal Models

One hundred specific-pathogen-free (SPF)-grade female ApoE^−/−^ mice (weighing 20 ± 5 g) were purchased from Beijing Vital River Laboratory Animal Technology Co., Ltd. (Beijing, China; production license number: SCXK (Beijing) 2024-0001) and housed in the SPF Animal Laboratory of the Institute of Basic Theory of Traditional Chinese Medicine, China Academy of Chinese Medical Sciences (laboratory animal use license number: SYXK (Beijing) 2021-0017). The housing environment was controlled at a temperature of 22 ± 1 °C and a relative humidity of 50–60%. This study was approved by the Animal Experimental Ethics Committee of the Institute of Basic Theory of Traditional Chinese Medicine, China Academy of Chinese Medical Sciences (approval number: IBTCMCACMS21-2409-08). After 7 days of adaptive feeding, the 100 ApoE^−/−^ mice were randomly divided into two groups: a control group (*n* = 15) fed with standard maintenance chow, and a high-fat diet group (*n* = 85) fed with a high-fat diet continuously for 8 weeks. At the end of week 8, left anterior descending coronary artery ligation was performed in ApoE^−/−^ mice according to previously reported methods [15]. Seventy-five successfully modeled mice were randomly divided into 5 groups (*n* = 15 per group): model group, MT group (1.3 mg·kg^−1^), low-dose PA group (PA-L, 25 mg·kg^−1^), medium-dose PA group (PA-M, 50 mg·kg^−1^), and high-dose PA group (PA-H, 100 mg·kg^−1^). The dose was selected based on previous studies demonstrating significant pharmacodynamic effects [38]. Mice in the control and model groups were intragastrically administered an equal volume of purified water daily for 4 consecutive weeks (Figure 1A).

### 4.2. Evaluation of Cardiac Function by Echocardiography

Before sample collection, the cardiac function of the mice was evaluated using an ultra-high-resolution small animal ultrasound imaging system (Vevo2100, FUJIFILM Sonosite, Toronto, ON, Canada, MS-250 probe, 21 MHz). Five mice from each group were fasted for 12 h and fixed in the supine position, with the chest depilated. The EF%, FS%, LVESD/LVEDD, and LVESV/LVEDV were measured, with all indices averaged over three cardiac cycles.

### 4.3. HE and Masson Staining

Heart tissues were fixed in 4% paraformaldehyde for 48 h, and 5 μm sections were prepared after dehydration and paraffin embedding. After baking and dewaxing, the sections were stained using HE and Masson staining kits (Servicebio Technology Co., Ltd., Wuhan, China, G1005, G1006) to observe myocardial fibrinoid deposition, collagen hyperplasia, and inflammatory cell infiltration.

### 4.4. Detection of Blood Lipid and Myocardial Enzyme Levels

Serum samples from six mice in each group were collected, and an enzyme-linked immunosorbent assay was used to detect the levels of blood lipids and myocardial enzymes, including CK, LDH, CK-MB, AST, TC, TG, LDL-C, and high-density lipoprotein cholesterol. The kits were purchased from Sesbor (Beijing) Biotechnology Co., Ltd. (Beijing, China; batch numbers: MB-5735A, MB-5900A, MB-5947A, MB-5658A, ADS-W-ZF014, ADS-W-ZF013, ADS-W-D012, and ADS-W-D011) and the experiments were performed strictly in accordance with the manufacturer’s instructions.

### 4.5. Plasma Proteomics Analysis

Six plasma samples were collected from mice in each group, and high-abundance proteins in plasma were removed using the Pierce™ Top14 Abundant Protein Depletion Spin Columns Kit according to the manufacturer’s instructions. Protein concentration was determined using the BSA method. For data-independent acquisition (DIA) analysis, chromatographic separation was performed using a nanoscale Vanquish Neo system, and the separated samples were subjected to DIA mass spectrometry (MS^2^) detection using an astral high-resolution mass spectrometer (Thermo Fisher Scientific, Bremen, Germany). The detection parameters were set as follows: positive ion mode, precursor ion scan range *m*/*z* 380–980, full-scan MS resolution of 240,000 at *m*/*z* 200, normalized automatic gain control (AGC) target of 500%, and maximum injection time of 5 ms. Tandem MS^2^ acquisition was performed in DIA mode with 299 scan windows, an isolation window of 2 *m*/*z*, higher-energy collisional dissociation collision energy of 25 eV, normalized AGC target of 500%, and maximum injection time of 3 ms. DIA data were processed using DIA-NN software (version 1.8.1). The Benjamini–Hochberg false discovery rate (FDR) correction was applied to correct for multiple testing. DEPs were screened with the criteria of |log2^fold change^| > 1 and *p* < 0.05. Functional enrichment analysis of the DEPs was conducted using the KEGG database to explore their biological functions and related signaling pathways.

### 4.6. Western Blotting

Total myocardial protein was extracted using a lysis buffer containing protease and phosphatase inhibitors, and protein concentration was determined using the bicinchoninic acid method. Proteins were separated using 8% sodium dodecyl sulfate–polyacrylamide gel electrophoresis and transferred to a polyvinylidene difluoride membrane, which was blocked with 5% skimmed milk for 1 h and incubated overnight at 4 °C with primary antibodies, including anti-glyceraldehyde 3-phosphate dehydrogenase (Proteintech, Wuhan, China, 60004-1-Ig, 1:5000), anti-C3 (Proteintech, Wuhan, China, 66157-1-Ig, 1:1000), anti-C3aR (Bioss, Beijing, China, bs-2955R, 1:1000), anti-BTK (Proteintech, Wuhan, China, 21581-1-AP, 1:1000), anti-CFD (Proteintech, Wuhan, China, 84851-4-RR, 1:2000), anti-TNFα (Proteintech, Wuhan, China, 17590-1-AP, 1:500), anti-IL-1β (Proteintech, Wuhan, China, 26048-1-AP, 1:1000), Caspase3 (Proteintech, Wuhan, China, 25128-1-AP, 1:1000), BAX (Proteintech, Wuhan, China, 50599-2-Ig, 1:500), and BCL2 antibody (Proteintech, Wuhan, China, 68103-1-Ig, 1:500). After washing with Tris-Buffered Saline with Tween-20, the membrane was incubated with horseradish peroxidase-labeled secondary antibody at 22–25 °C for 1 h. Development was performed using an enhanced chemiluminescence kit (Proteintech, Wuhan, China, PK10001), images were acquired using a Bio-Rad ChemiDoc XRS+ imaging system, and band gray values were analyzed using ImageJ software (version 1.54p).

### 4.7. TUNEL Staining of Myocardial Tissues

Myocardial apoptosis was detected using a TUNEL staining kit (Wuhan Servicebio Technology Co., Ltd., Wuhan, China, G1507), TUNEL-positive cells were identified as cardiomyocytes with clear brown nuclear staining under an optical microscope. Each section was observed at 200× magnification, and five randomly selected non-overlapping fields were analyzed per sample. The number of TUNEL-positive cells and total cardiomyocytes were counted, and the cardiomyocyte apoptotic rate was quantified using ImageJ software.

### 4.8. Plasma Non-Targeted Metabolomics Analysis

Six plasma samples from mice in each group were randomly selected and thawed at 22–25 °C to prepare a pooled quality control (PQC) sample by mixing 10 μL of each sample with vortexing. A total of seven isotopically labeled internal standards were used in this study, including Alanine-D4 (ANPEL, Shanghai, China, CDVD-A301-1061), Methionine-D3 (ANPEL, Shanghai, China, CDVD-A301-1014), Propionylcarnitine-D3 hydrochloride (ANPEL, Shanghai, China, CDVD-A301-1032), Carnitine-D3 hydrochloride (ANPEL, Shanghai, China, CDAA-210058), Aspartic acid-D3 (ANPEL, Shanghai, China, CDAA-270003D3), Tyrosine-D4 (ANPEL, Shanghai, China, CDAA-270016D4), and Succinic acid-2,2,3,3-D4 (Sigma, Beijing, China, 293075). Pre-chilled methanol at −80 °C for more than 30 min was used as the extraction solvent and blank solution. Subsequently, 20 μL of serum, PQC, and blank samples were respectively transferred to 2 mL centrifuge tubes, followed by the sequential addition of 20 μL internal standard working solution and 110 μL pre-chilled methanol extract. The volume ratio of plasma sample to pure methanol extractant was 20:110 (*v*/*v*), resulting in a final methanol volume proportion of approximately 84.6% in the extraction mixture. The mixture was vortexed for 1 min, incubated at −20 °C for >30 min, and centrifuged at 14,000× *g* for 10 min at 4 °C. The supernatant was transferred to a new centrifuge tube, freeze-dried under vacuum at −50 °C with a vacuum pressure of 0.01 mbar for 4 h, and reconstituted with 100 μL of 10% methanol aqueous solution. After vortexing and sonication, the mixture was centrifuged again at 14,000× *g* for 10 min at 4 °C, and the supernatant was transferred to an injection vial for MS^2^ detection.

Chromatographic separation was performed using an ACQUITY UPLC HSS T3 column (1.7 μm × 2.1 mm × 100 mm) with mobile phases consisting of 0.1% formic acid aqueous solution (phase A) and 0.1% formic acid-acetonitrile-methanol solution (phase B) under gradient elution at a flow rate of 0.25 mL·min^−1^, column temperature of 40 °C, and injection volume of 5 μL. Two mobile phase condition was used to improve the metabolite coverage. For condition 1, solvent A (0.1% formic acid in water) and solvent B (0.1% formic acid in acetonitrile/methanol = 4/6) were used to elute the metabolites with a 13.5 min gradient, as follows: 10% B at 0 min, 0.25 mL/min; 40% B at 3 min, 0.25 mL/min; 95% B at 5 min, 0.25 mL/min; 100% B at 8 min, 0.6 mL/min; 100% B at 10 min, 0.6 mL/min; and back to 10% B at 10.5 min, 0.25 mL/min; and equilibrate for 3 min. Samples were analyzed using a Q Exactive HF-X (QE-HF-X) (Thermo Fisher Scientific (Bremen) GmbH, Bremen, Germany) mass spectrometry equipped with a heated electro-spray ionization (HESI) source. All the data was acquired in positive and negative switching mode using Full scan detection, and the PQC were also analyzed with Full scan/ddMS2 to acquire MS2 fragmentation for metabolite identificaiton and annotation. For condition 2, solvent A was 6.5 mM NH4HCO3 in water, solvent B was 6.5 mM NH4HCO3 in methanol. The 12 min gradient was employed: 0 min, 0.25 mL/min, 10% B; 4 min, 0.25 mL/min, 40% B; 6 min, 0.25 mL/min, 95% B; 8 min, 0.4 mL/min, 99% B; 8.5 min, 0.4 mL/min, 99% B; 8.6 min, 0.25 mL/min, 10% B; 12 min, 0.25 mL/min, 10% B. Data acquisition were performed only in negative mode, and Full scan was used for all the samples, and Full scan/ddMS2 was also used for PQC samples. The Full Scan settings were as follows: 60,000 resolution, AGC target, 1 × 10^6^; Maximum IT, 100 ms; scan range, 60 to 900 *m*/*z*. For Full scan/ddMS2 (DDA), Top 20 MS/MS spectral (dd-MS2) @ 15000 were generated with AGC target = 2 × 10^5^, Maximum IT = 25 ms, and (N)CE/stepped NCE = 10, 40, 80 v. Metabolites detection and identification were performed using MS-dial (ver.5.1.230912) by searching against online database (MoNA, GNPS, HMDB and MS-dial database) and in-house database.

After logarithmic transformation of the raw data, OPLS-DA was conducted using the ropls R package (1.38.0) to calculate VIP values. The Benjamini–Hochberg FDR correction was applied to control for multiple testing. Differential metabolites were screened with the criteria of VIP > 1 and *p* < 0.05, and metabolic pathway enrichment analysis was performed using the MetaboAnalyst software (version 5.0).

### 4.9. Molecular Docking

Molecular docking between the core compounds and targets was performed. Molecular Operating Environment 2019 software was used for energy minimization of compounds, pretreatment of target proteins, and identification of active pockets. Subsequently, molecular docking with 50 iterations was conducted using Molecular Operating Environment 2019. The binding activity between compounds and targets was evaluated based on binding energy, and the results were visualized using PyMOL 2.6.0 and Discovery Studio 2019 software.

### 4.10. Statistical Analysis

Data are presented as mean ± standard deviation. An independent samples *t*-test was used for comparisons between two groups, and a one-way analysis of variance was used for comparisons among three or more groups. All statistical analyses were performed using GraphPad Prism software (version 7.0, San Diego, CA, USA). Statistical significance was set at *p* < 0.05.

## 5. Conclusions

PA exerts comprehensive anti-CHD effects through multiple targets and pathways. Its core mechanism is inhibiting the abnormal activation of the C3-Cfd-C3aR-BTK signaling axis, thereby suppressing myocardial inflammation, reducing cardiomyocyte apoptosis, and blocking C3aR-mediated platelet activation. Furthermore, PA can correct glycerophospholipid metabolism disorders, restore homeostasis of GABAergic synapses and retrograde endocannabinoid signaling pathways in the neuroactive ligand-receptor network, and synergistically improve cardiac function and myocardial structural integrity. This study provides new experimental and mechanistic evidence for PA as a candidate drug for CHD treatment and offers new ideas for research on complement-neuroimmune interactions in cardiovascular diseases.

## Figures and Tables

**Table 1:** Paeoniflorin can significantly regulate metabolites.

No.	Metabolite	Category	Trend of Change	
			Model/ Control	PA/ Model
1	Myristoylcarnitine	Fatty Acid Metabolism	↑	↓
2	Octenoylcarnitine	Fatty Acid Metabolism	↑	↓
3	Oxodecanoylcarnitine (C10:1-O)	Fatty Acid Metabolism	↑	↓
4	Phenylpropionylglycine	Fatty Acid Metabolism	↓	↑
5	9-Octadecenamide	Fatty Acid Metabolism	↓	↑
6	Dodecanedioic acid	Fatty Acid, Dicarboxylate	↑	↓
7	Octadecanedioate (C18-DC)	Fatty Acid, Dicarboxylate	↑	↓
8	Dodecanedioate (C12-DC)	Fatty Acid, Dicarboxylate	↑	↓
9	Tetradecanedioate (C14-DC)	Fatty Acid, Dicarboxylate	↑	↓
10	2-HYDROXY-4-METHYLPENTANOIC ACID	Fatty Acid, Monohydroxy	↑	↓
11	Undecylenic Acid (C11:1n1)	Medium Chain Fatty Acid	↓	↑
12	Stearic Acid (C18:0)	Long Chain Fatty Acid	↑	↓
13	Pentadecanoic Acid (C15:0)	Long Chain Fatty Acid	↑	↓
14	LPA 15:0_sn1	Glycerophospholipid	↓	↑
15	LPC 15:0_sn1	Glycerophospholipid	↓	↑
16	LPE 22:6_sn2	Glycerophospholipid	↓	↑
17	LPG 18:2	Glycerophospholipid	↑	↓
18	LPI 20:4	Glycerophospholipid	↑	↓
19	LPI 20:3	Glycerophospholipid	↑	↓
20	PC 32:0|PC 16:0_16:0	Glycerophospholipid	↓	↑
21	PC 39:4	Glycerophospholipid	↓	↑
22	PC 40:7|PC 18:1_22:6	Glycerophospholipid	↓	↑
23	PC 42:8|PC 20:2_22:6	Glycerophospholipid	↓	↑
24	PC 38:6|PC 16:0_22:6	Glycerophospholipid	↓	↑
25	PE 38:6|PE 18:2_20:4	Glycerophospholipid	↓	↑
26	Gamma-Glutamylphenylalanine	Gamma-glutamyl Amino Acid	↓	↑
27	Gamma-Glutamylvaline	Gamma-glutamyl Amino Acid	↓	↑
28	Gamma-Glutamylleucine	Gamma-glutamyl Amino Acid	↓	↑
29	L-Glutamic Acid	Alanine, Aspartate and Glutamate Metabolism	↑	↓
30	L-Asparagine	Alanine, Aspartate and Glutamate Metabolism	↑	↓
31	Gamma-Aminobutyric Acid	Alanine, Aspartate and Glutamate Metabolism	↑	↓
32	N-Acetylphenylalanine	Phenylalanine Metabolism	↓	↑
33	Phenylpyruvic Acid	Phenylalanine Metabolism	↓	↑
34	3-(2-Hydroxyphenyl)propanoic acid	Phenylalanine Metabolism	↓	↑
35	Aminoadipic Acid	Lysine Metabolism	↑	↓
36	N6-Methyllysine	Lysine Metabolism	↓	↑
37	L-Pipecolic Acid	Lysine Metabolism	↓	↑
38	Phosphotyrosine	Tyrosine Metabolism	↑	↓
39	Tyrosine O-sulfate	Tyrosine Metabolism	↑	↓
40	Gly-Ile	Dipeptide	↓	↑
41	Ile-Leu	Dipeptide	↓	↑
42	4-hydroxybenzaldehyde	Benzoate Metabolism	↓	↑
43	Phe-DCA	Bile Acid, amino acid conjungate	↑	↑
44	2,6-dihydroxybenzoic acid	Chemical	↓	↑
45	Betaine	Glycine, Serine and Threonine Metabolism	↓	↑
46	Glucuronate	Amino sugar and Nucleotide sugar Metabolism	↓	↑
47	12-HHTrE	Eicosanoid	↑	↓
48	Trigonelline (N′-Methylnicotinate)	Nicotinate and Nicotinamide Metabolism	↓	↑
49	Gamma-caprolactone	Natural Product	↑	↓
50	5,6-Dihydrouracil	Pyrimidine Metabolism	↑	↓
51	N-Alpha-Acetylornithine	Arginine and Proline Metabolism	↓	↑
52	Heneicosanoic acid	Unclassified	↑	↓
53	LPE O-20:1	Unclassified	↓	↑
54	LPI 22:6	Unclassified	↑	↓
55	LPE O-20:0	Unclassified	↓	↑
56	LPE O-22:0	Unclassified	↓	↑
57	Hippuric acid	Unclassified	↓	↑

↑ upregulated; ↓ downregulated.

## Data Availability

The data that support the findings of this study are available from the corresponding author upon reasonable request.

## Supplementary Materials

The following supporting information can be downloaded at: https://www.mdpi.com/article/10.3390/ijms27146236/s1.

## Abbreviations

The following abbreviations are used in this manuscript:

AGC	Automatic gain control
AST	aspartate aminotransferase
BAX	bcl-2-associated X protein
BCL-2	b-cell lymphoma 2
BTK	bruton’s tyrosine kinase
C3aR	complement C3a receptor
Cfd	complement factor D
CHD	coronary heart disease
CK	creatine kinase
CK-MB	ck-isoenzyme MB
DEPs	differentially expressed proteins
DIA	data-independent acquisition
FS	fractional shortening
GABA	gamma-aminobutyric acid
GO	gene Ontology
HE	hematoxylin-eosin
Il-1β	interleukin-1β
KEGG	kyoto Encyclopedia of Genes and Genomes
LDH	lactate dehydrogenase
LDL-C	low-density lipoprotein cholesterol
LVEDD	left ventricular end-diastolic diameter
LVEDV	left ventricular end-diastolic volume
LVESD	left ventricular end-systolic diameter
LVESV	left ventricular end-systolic volume
MS^2^	mass spectrometry
MT	metoprolol tartrate
PCA	principal component analysis
PA	paeoniflorin
PA-H	high-dose paeoniflorin
PA-L	low-dose paeoniflorin
PA-M	medium-dose paeoniflorin
PQC	pooled quality control
TC	total cholesterol
TG	triglycerides
TNF-α	tumor necrosis factor α
TUNEL	terminal deoxynucleotidyl transferase dUTP nick-end labeling
VIP	variable importance in projection
