# Supplementary material for: Multi-Omics and Experimental Validation Reveal the Protective Effect of Paeoniflorin Against Coronary Heart Disease in Mice via Inhibiting the C3-Cfd-C3aR Pathway
Source: Int J Mol Sci. 2026 Jul 13;27(14):6236. doi: 10.3390/ijms27146236 (PMC13410309; doi:10.3390/ijms27146236)
Supplement: Supplementary file 1 [file ijms-27-06236-s001.zip › Supplementary Materials/ijms-4276706_Metabolomics_Dataset/Σ╗úΦ░óτ╗äσ¡aμèÑσæèΦoúΦ»╗μûçμíú.pdf]

广科安德非靶代谢组学分析

结果文件说明文档

目录

结果文件说明 3

一． 分析思路 3

二． 文档组成 3

三． 文档说明 5

结果文件说明

一. 分析思路

非靶向代谢组（Untargeted Metabolomics）是一种常用的代谢组学研究方法，主要研究思路为对比实验组和对照组，检测样本中含有的代谢物，并获取定量信息，找到不同组别之间具有统计学意义的差异代谢物，可解释所发现的代谢物与生物过程或生物状态之间的联系。

此报告中涵盖了本次非靶代谢组学实验的基本数据结果、数据质控、差异分析以及后续富集分析的结果。基于质谱检测得到的 Raw 文件，后续分析思路如下：1）根据样本的来源情况构建代谢组数据库，然后采用质谱原始数据解析软件 MS-DIAL 进行代谢物数据库检索；2）基于实验过程中加入内标代谢物进行质控分析；3）基于 HMDB 和 KEGG 数据库对鉴定到的代谢物进行注释分类；4）数据预处理：代谢物的定量结果进行对数转换、中心化和缺失值填补处理；5）定量重复性分析：包含标准品的 CV 分析、所有样本的 PCA 分析和 PCC 分析；6）差异统计分析：包含单因素的差异分析、多因素的正交偏最小二乘判别分析；7）代谢物分类统计：包含 KEGG 和 HMDB 化合物分类统计；8）基于 KEGG pathway 数据库，采用 Fisher's exact test 和 MSEA 算法对差异分析结果中的代谢物进行富集分析，从而筛选重要的通路。

二. 文档组成

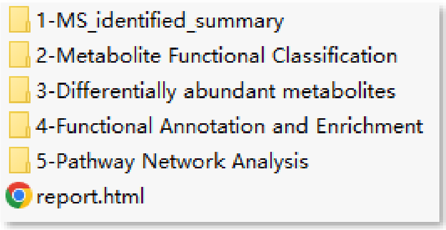

1) **质谱数据基本分析文件**
   - 文档名称：**1-MS_identified_summary**
   - 文档内容：质谱鉴定到的代谢物定性和定量信息、数据质控结果以及样本重复性检验结果。
2) **代谢物注释文件**
   - 文档名称：**2-Metabolite Functional Classification**
   - 文档内容：基于 HMDB 和 KEGG 数据库对代谢物类别以及其参与的生物学通路进行注释与分类。
3) **差异代谢物文件**
   - 文档名称：**3-Differentially abundant metabolites**
   - 文档内容：根据单维（T.test）和多元统计分析（OPLS-DA）结果筛选到的差异代谢物结果。

4) **差异代谢物功能注释与富集文件**
   - 文档名称：**4-Functional Annotation and Enrichment**
   - 文档内容：差异代谢物 HMDB 与 KEGG 通路注释和富集结果。
5) **差异代谢物富集通路网络图**
   - 文档名称：**5-Pathway Network Analysis**
   - 文档内容：差异代谢物富集通路网络分析结果。
6) **项目报告文档**
   - 文档名称：**report.html**
   - 文档内容：项目信息，客户单位信息，实验流程，生物信息分析结果展示，实验材料与方法，数据分析方法以及分析使用的软件版本。

三． 文档说明

0_Report.html

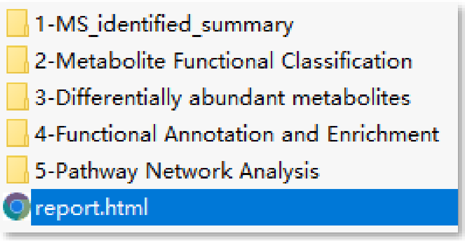

hmtl 网页版报告文档基本囊括了质谱下机后数据分析所产生的所有重要图表结果，其内容如下：1. 研究概述 2. 技术路线 3. 分析流程 4.分析结果（包括质谱鉴定到的代谢物基本信息、数据质控、代谢物注释、样品重复性检验、差异代谢物筛选及其通路富集结果） 5. 材料与方法 6. Method and Materials (English version) 。

1-MS_identified_summary

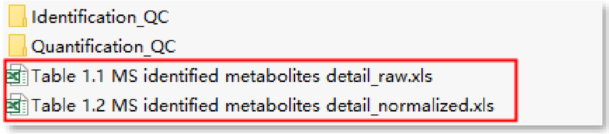

本文件夹下包含两个 Excel 格式文件，分别是质谱鉴定的代谢物丰度定量原始数据（Table 1.1 MS identified metabolites detail_raw.xls）和标准化后的数据（Table 1.2 MS Identified metabolites detail_normalized.xls）。为了消除系统误差和允许不同样本/代谢物件进行对比，我们对质谱定量数据进行了一系列标准化处理，流程包括: 1）log2 对数转换；2）缺失值过滤（剔除缺失值超过样本数 1/3 的代谢物）；3）中位数归一化（列） + 固定值（默认 15）；4）KNN 缺失值填补。

1） Table 1.1-1.2 MS identified metabolites detail_ .xls 表头信息解读如下：

Metabolite name	Category1	Category2	HMDB ID	Alignment ID	Average Rt(min)	Average Mz	Adduct type	Mode	S/N average	Control1	Control2	Control3	Control4	Control5	G1	G2	G3	G4	G5	PQC-1	PQC-2	PQC-3
(9Z,12R)-12-Hydr	Lipid	Fatty Acid,	HMDB0003	Meta_329	6.833	297.24304	[M-H]-	Negativ	12.91	319348	1508971	1310525	1189172	830746	274136	474971	1673597	1050114	1368286	1243172	1199185	954163
1,5-Naphthalened	Xenobiotic	Chemical	HMDB0024	Meta_116	3.263	159.09151	[M+ACN+H]+	Positive	6.52	5540357	5286879	5486704	6495771	5305720	5574826	4494596	3781028	4052654	3938635	5456075	6365857	4360688
1-Methylcytosine	Nucleotide	Pyrimidine (	HMDB0024	Meta_56	1.241	126.06297	[M+H]+	Positive	22.61	11116239	10669569	11668826	12960779	8773876	12409341	12593533	11158995	12276121	12357440	11307644	10943198	10317143

Metabolite name	代谢物名称
Category1	代谢物一级分类
Category2	代谢物二级分类
HMDB ID	代谢物 HMDB ID 号
Alignment ID	代谢物在分析时的编号
Average Rt(min)	质谱信息:平均滞留时间（min）
Average Mz	质谱信息:平均质荷比
Adduct type	加合物类型
Mode	正负极模式
S/N average	信噪比均值—检测信号强度与背景噪音的比率，是反应峰的相对强度和质量的重要指标，S/N 值越高，数据的可靠性越好。
Case1-3	样本数据
PQC1-3	质控样本

2）Identification_QC

Identification_QC 文件夹下包含代谢物定性后的内标物质控分析结果，QC plot 展示了内标物在不同样本中的信号强度。在理想情况下，同一内标物在不同样本间的信号强度应大致处于相同水平，代表样本质量和实验稳定性强。

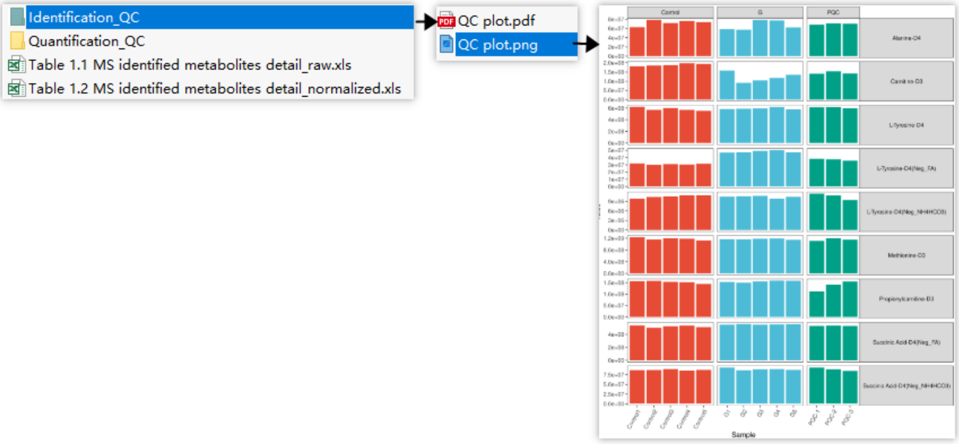

4) Quantification_QC

Quantification_QC 文件夹下包含代谢物定量的质控分析结果，由于项目有生物重复或技术重复样本，因此需要进行定量重复性检验分析，图 A-D 既是从不同维度下检验生物学重复或技术重复样本的定量结果是否符合统计学上的一致性。

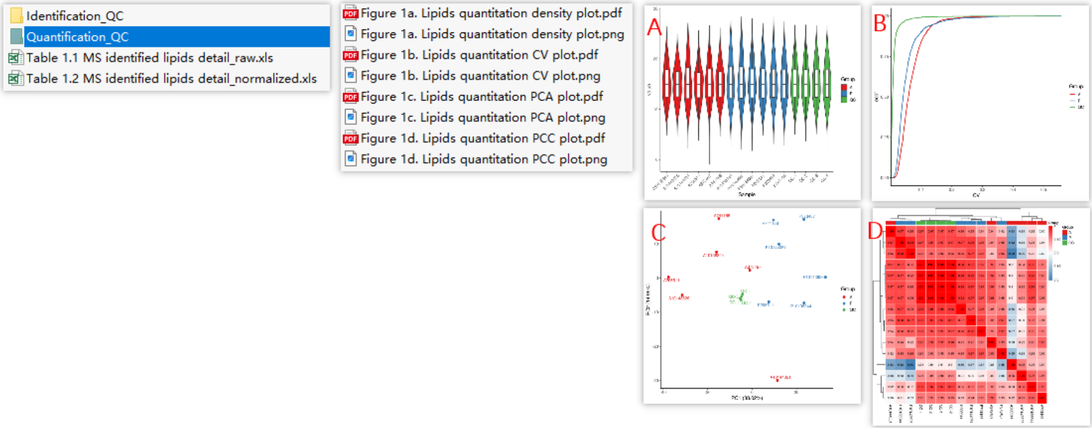

（**图 A**）：采用小提琴图和箱线图结合的方式来展示每个样本中代谢物相对丰度的整体分布情况，横坐标为不同样本，纵坐标为标准化后的代谢物相对丰度分布，理论上不同样本应处于同一水平线上。（**图 B**）：根据各组重复样本间代谢物定量值的变异系数（CV）绘制的累计曲线图，整体 CV 值越小，定量重复性越好。（**图 C**）：所有样本的代谢物定量主成分分析（PCA）结果展示图，图中样本间的聚集程度代表样本间的差异性大小。理论上相同组别的生物学/技术重复样本更倾向于聚集在一起，PQC 样本是所有样本混合制成的质控样本，因此 PQC 样本的聚集情况代表了实验条件的稳定性和一致性。（**图 D**）：所有样本两两之间的皮尔森相关系数热图。皮尔森系数是衡量两组数据相关程度的值，当皮尔森系数越接近 1 为正相关（红色），越接近-1 为负相关（蓝色），接近 0 为不相关。

2-Metabolite Functional Classification

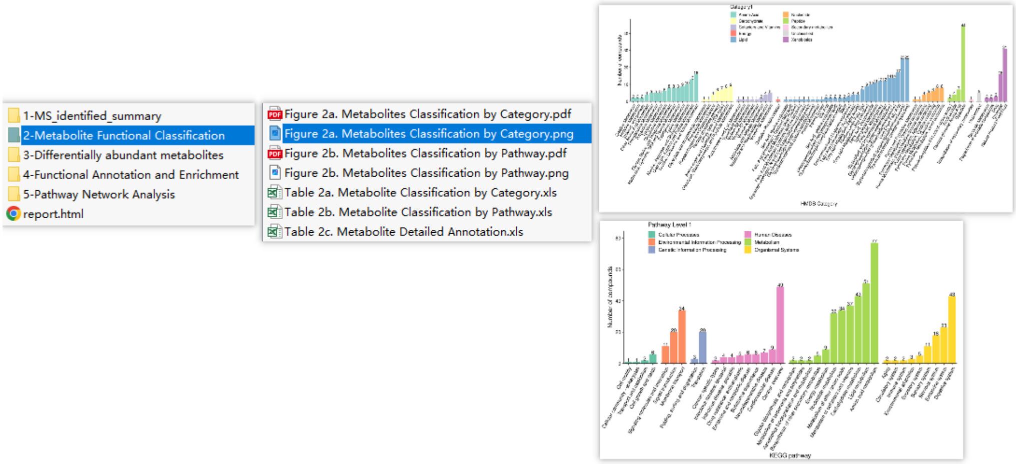

为了透彻了解样本中代谢物的潜在功能，我们对检出的代谢物进行了简单的分类统计，分类基于 HMDB 和 KEGG compound 数据库对代谢物的功能定义。

Figure 2a. Metabolites Classification by Category

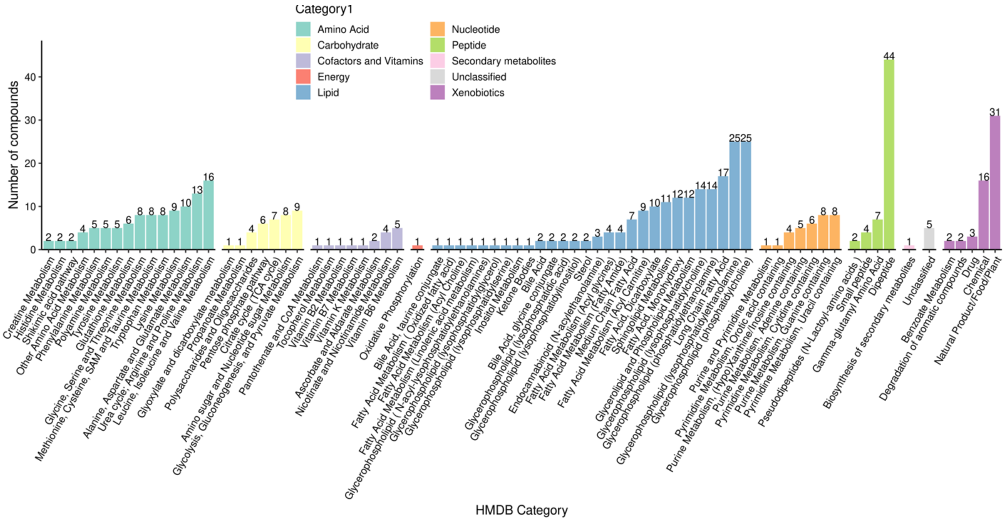

Table 2a. Metabolite Classification by Category

Category1	Category2	Freq	Metabolites
Amino Acid	Alanine, Aspartate and Glutamate Metabolism	10	Gamma-Aminobutyric Acid ; Hydroxyasparagine ; L-Alanine ; L-Asparagine ; L-Aspartic Acid
Amino Acid	Creatine Metabolism	2	Creatine ; Creatinine

根据 HMDB 和 KEGG compound 数据库对鉴定到的代谢进行分类，如 Table 2a 中表头展示，Category1 和 Category2 分别代表代谢物的主要类别和细分类别，Freq 代表归属这一类的代谢物数量，Metabolites 是对应的代谢物名称。柱形图中的纵坐标代表代谢物数量，

柱子的颜色代表代谢物的一级分类（Category1），横轴代表二级分类（Category2）。

Figure 2b. Metabolites Classification by Pathway

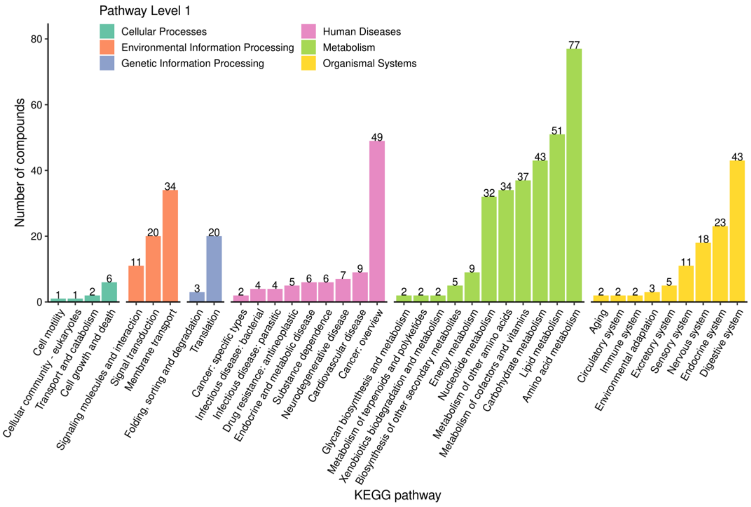

Table 2b. Metabolite Classification by Pathway

Pathway.level.1	Pathway.level.2	Freq	Metabolites	HMDBs
Cellular Processes	Cell growth and death	6	Glutathione ; L-Glutamic Acid ; Arachidonic Acid (C20:4n6)	HMDB0000125;HMDB0000148;HMDB0001043;HMDB0001049;HMDB0001893;HMDB0003337
Cellular Processes	Cell motility	1	Acetylcholine	HMDB0000895
Cellular Processes	Cellular community - euk	1	L-Glutamic Acid	HMDB0000148

依据 KEGG Pathway 数据库，我们将不同代谢物所属的信号通路进行了注释，如 Table 2b 中表头展示，Pathway level 1 和 Pathway level 2 是通路的一级和二级分类，Freq 是所属通路代谢物的数量，Metabolites 是代谢物名称，HMDBs 是所属通路下代谢物的 HMDB IDs。柱形图展现方式与前者一致，因此不再赘述。

Table 2c. Metabolite Detailed Annotation

Metabolite name	Category1	Category2	HMDB ID	chemical_formula	inchikey	drugbank_id	foodb_id	chebi_id	cpd_id	Alignment ID	Average Rt(min)	Average Mz	Adduct type	Mode	Control1	Control2
(9Z,12R)-12-Hydrc	Lipid	Fatty Acid, M	HMDB0034297	C18H34O3	WBHHMMIMDMUBKC-XFXZXTDPSA-N		FDB01264(	CHEBI:85639		Meta_329	6.833	297.24304	[M-H]-	Negative_F	11.332	13.649
1,5-Naphthalened	Xenobiotics	Chemical	HMDB0244231	C10H10N2	KQSABULTKYLFEV-UHFFFAOYSA-N			CHEBI:53003		Meta_116	3.263	159.09151	[M+ACN+H]	Positive	15.449	15.458
1-Methylcytosine	Nucleotide	Pyrimidine M	HMDB0243939	C5H7N3O	HWPZZUQOWRWFDB-UHFFFAOYSA-N			CHEBI:39624		Meta_56	1.241	126.06297	[M+H]+	Positive	16.454	16.471

Table 2c 代谢物详细注释表格相比于 Table 1.2 多了几列注释信息，其中包括 chemical_formula 代谢物的化学式；inchikey 数据库 ID；drugbank 数据库 ID；foodb 数据库 ID；CheBI 数据库 ID；cpd_id KEGG 数据库 ID（便于客户查询其他感兴趣的数据库）。

3-Differentially abundant metabolites

差异代谢物的分析与筛选根据生物重复样本量以及客户需求而变化，详细信息见 html 版报告。通常在进行代谢组学差异分析时，我们会用到多元统计分析（OPLS-DA）和单维统计分析（T-test），并使用三个条件来筛选差异代谢物：变化倍数（FoldChange），p 值和 VIP 值。我们默认使用的阈值是 Pvalue<0.05, |FoldChange|>0, VIP>1，实际阈值会根据客户数据变化，详见 html 版报告 Session 4.5 差异分析。

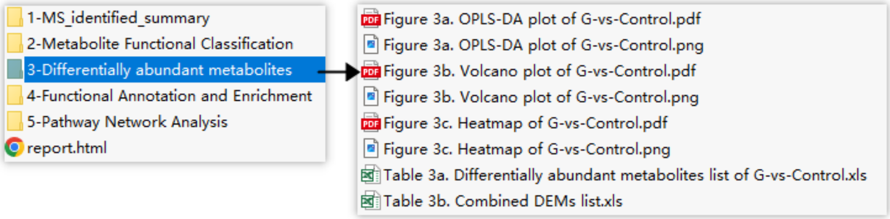

Table 3a. Differentially abundant metabolites list of Case-vs-Control

Alignment ID	Metabolite name	Category1	Category2	HMDB ID	log2fc	p.value	fdr	VIP	regulation	Control1	Control2	Control3	Control4	Control5	G1	G2	G3	G4	G5
Meta_111	L-Histidine	Amino Acid	Histidine Metabolism	HMDB0000177	-1.053	0.00056	0.000557	1.250872	down	14.685	14.578	14.96	15.38	14.9	13.972	13.621	14.217	13.489	13.938
Meta_112	Orotic acid	Nucleotide	Pyrimidine Metabolism, Orotic acid containing	HMDB0000226	-2.513	0.00048	0.000478	1.300458	down	12.827	12.613	12.268	11.941	12.7	9.901	8.961	10.656	10.654	9.611
Meta_115	Homostachydrine	Xenobiotics	Natural Product/Food/Plant	HMDB0004827	-1.071	2.17E-06	2.17E-06	1.373883	down	17.621	17.446	17.502	17.71	17.607	16.573	16.377	16.541	16.344	16.694

3-Differentially abundant metabolites 文件夹中的 Table 3a 表格文件分别展示了不同组别进行差异分析后的结果，每个 excel 文件对应一个比较组，其表头中包含以下几列：Alignment ID、Metabolite name、Category1、Category2、HMDB ID、log2fc（对数转换后的差异倍数=标准化后的 Case 组平均值减去 Control 组平均值）、p.value（T.test 计算出的显著性 p value，默认 p.value<0.05 为显著）、fdr（校正后的 p 值，由于代谢物鉴定数量有限，默认不进行 p 值校正，如客户有校正需求，可进行个性化售后）、VIP（根据 OPLS-DA 模型得到的 VIP 值，值大小代表某代谢物对于两组差异/模型的贡献度）、regulation（代谢物上下调）、Case1-3~Control1-3（样本数据）。

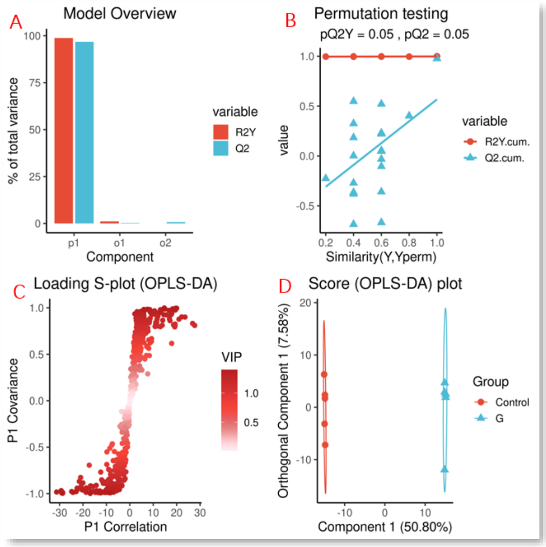

Figure 3a. OPLS-DA plot of Case-vs-Control

为了进一步区分两组样本间的代谢谱差异，我们运用有监督的判别模型——正交偏最小二乘法判别模型（OPLS-DA）进行分析，该方法的特点是可以去除自变量 X 中与分类变量 Y 无关的数据变化，即过滤与分组无关的噪音信号，从而更好地聚焦于预期关注的生物学信息。

上图为 OPLS-DA 分析产生的结果图:

（**图 A**）：柱状图展示 OPLS-DA 模型主成分数目选择，柱子的高度代表主成分对整体变量的解释程度，R2Y 和 Q2 分别用来评价 OPLS-DA 模型的解释能力和预测能力，R2Y 和 Q2 的累计值越大，说明模型越稳定可靠。

（**图 B**）：OPLS-DA 模型交叉验证图，横坐标表示置换检验的置换保留度（与原模型 Y 变量顺序一致的比例，置换保留度为 1 的点即为原模型的 R2 和 Q2 值），纵坐标表示 200 次置换检验中 200 个模型的准确率，其中 R2Y（红色圆点）和 Q2（蓝色三角）分别表示缩减模型对 Y 矩阵的解释率以及模型的预测能力，两条虚线分别表示 R2Y 和 Q2 的回归线，理论上 R2Y 和 Q2 数值越接近 1 说明模型越好，越低说明模型的拟合准确性越差，通常情况下，R2、Q2 高于 0.5 较好，高于 0.4 即可接受。图上方展示了 R2Y 和 Q2 的 p 值（pR2Y 和 pQ2），表示有多大可能性 200 个随机模型中有表现优于本 OPLS-DA 模型的结果（例如当

Q2 和 R2Y 的 P 值均为 0.005 时，说明 200 次置换检验中只有 1 个随机分组模型结果优于本 OPLS-DA 模型)，一般情况下 P<0.05 时模型最佳。

（**图 C**）：载荷图（Loading S-plot）是 OPLS-DA 分析中常用的展示图，能够帮助我们理解模型中每个变量的影响程度。图中的横坐标表示主成分与代谢物的相关系数，纵坐标表示主成分与代谢物的协相关系数。每个点代表一个变量（代谢物），其位置反映了该代谢物对模型的贡献，离原点（0,0）越远的点，表示该变量对主成分的贡献越大，即这个变量在区分组间样本上的影响力越大，因此越靠近两个角的代谢物重要度越强。点的颜色代表代谢物对模型的贡献度（VIP 值），通常情况下我们通过 VIP 值大于 1 来筛选对模型有重要贡献的代谢物。

（**图 D**）：OPLS-DA 得分图，OPLS-DA 通过正交信号校正，过滤掉与分组无关的信息，从而能更好的区分组间差异，提高模型的效能。得分图的横坐标表示正交旋转过程中的主要成分的解释度，所以横坐标的方向可以看到组间的差异；纵坐标表示正交旋转过程中的正交成分的解释度，所以纵坐标可以看到组内样本间的差异。

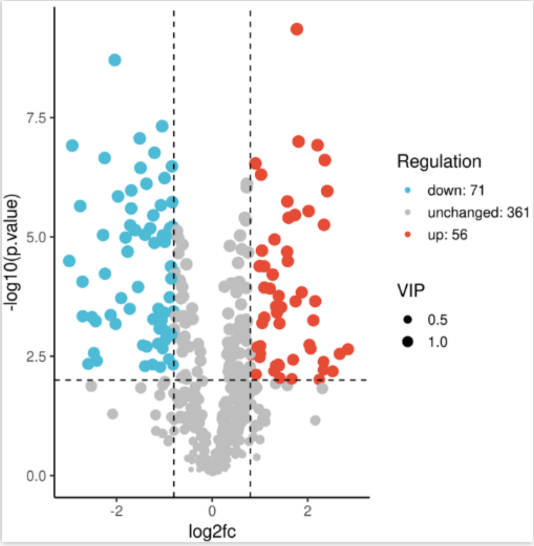

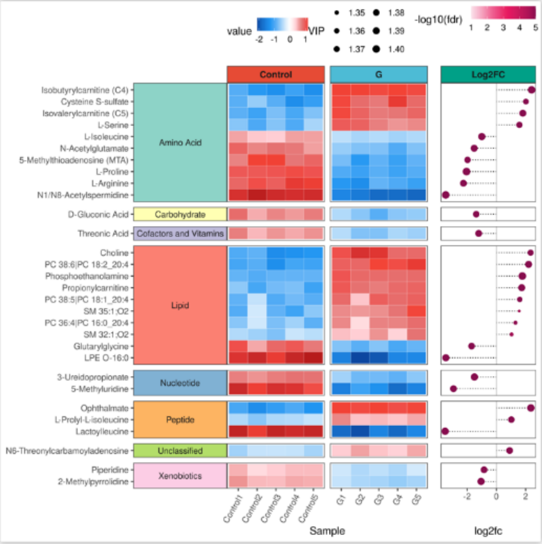

Figure 3b. Volcano plot & Figure 3c. Heatmap of Case-vs-Control

左图为差异分析的火山图，横轴为对数转换后的差异倍数（log2fc），纵轴为 log10 调整后的 P 值，红色为显著上调代谢物，蓝色为显著下调代谢物。右图是用筛选出的差异代谢物绘

制的热图，纵轴每一列代表一个样本，横轴每一行代表一个差异代谢物，格子颜色的深浅代表差异代谢物的相对丰度，红色代表相对高丰度，蓝色代表相对低丰度。最右侧一列是差异倍数点图，圆圈大小代表某代谢物的 VIP 值，圆圈距离 0 坐标轴的位置代表代谢物的 log2fc 值，距离 0 越远代表差异越大，该代谢物越值得关注。

4-Functional Annotation and Enrichment

为透彻了解比较组中差异代谢物的潜在功能，我们对上一步筛选出的差异代谢物进行了简单的分类统计，分类基于 HMDB 和 KEGG compound 数据库对代谢物的功能定义和参与通路。

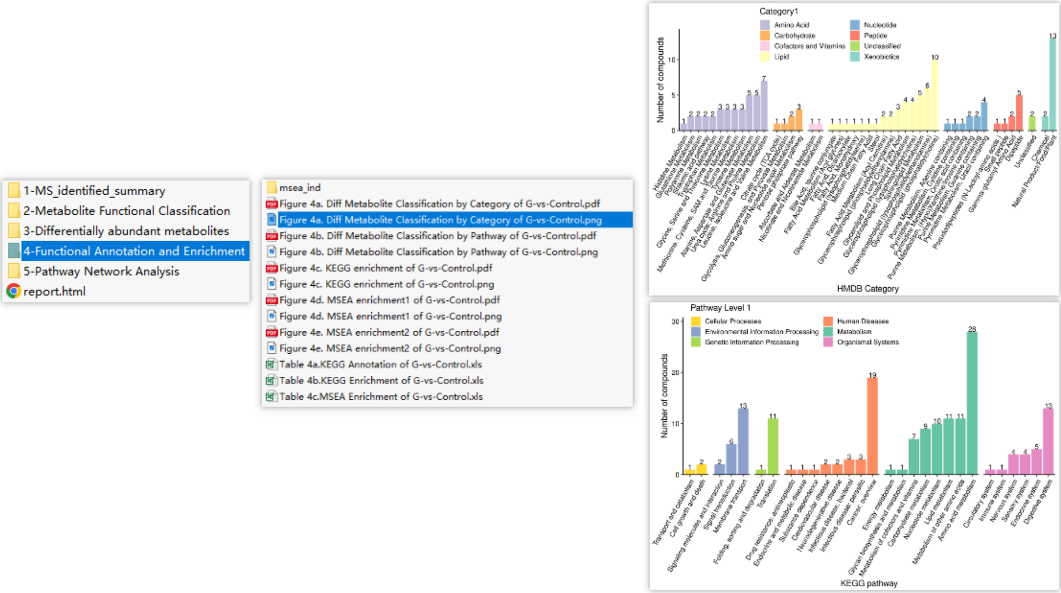

以上柱形图的解读方式与文件夹 **2-Metabolite Functional Classification** 一致，旨在对筛选出的差异代谢物的类别及其参与的代谢通路有个大致了解。

基于 KEGG 通路数据库，我们对上述筛选到的差异代谢物进行了详细注释和深入分析，使用 Fisher’s exact test（即费希尔精确检验）来计算差异代谢物是否在某条通路中有显著富集的 P value，并利用气泡图的方式来展现差异代谢物的通路富集结果，默认 p-value < 0.05 为显著富集。KEGG 注释信息详见 Table 4a.KEGG Annotation of Case-vs-Control.xls，KEGG 富集结

果详见 Table 4b.KEGG enrichment of Case-vs-Control.xls。

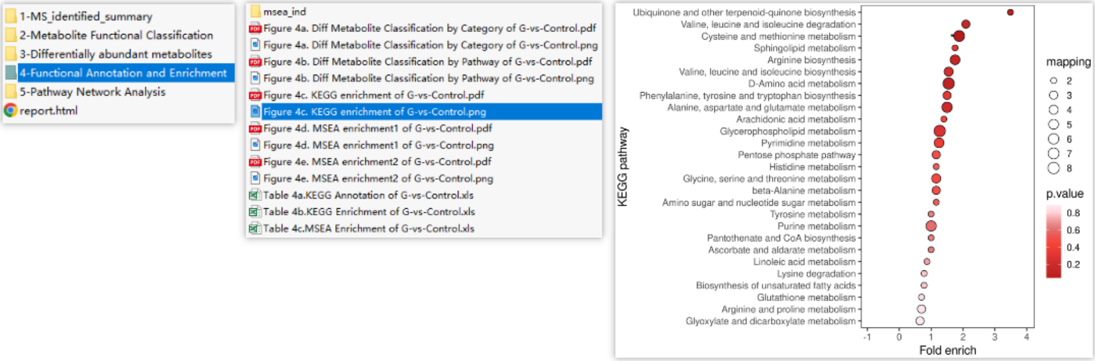

Figure 4c. KEGG enrichment of Case-vs-Control

以上气泡图中默认展示最显著富集的前 20 个通路的结果，纵轴为 KEGG 通路，横轴为差异代谢物在该通路中所占比例相比于鉴定代谢物所占比例的变化倍数（Fold enrich），气泡颜色深浅表示富集显著性 P value（颜色越深越显著），气泡大小表示通路中的差异代谢物个数，气泡边上的*号代表富集显著程度（*代表 P value<0.05, **代表 P value <0.01）。

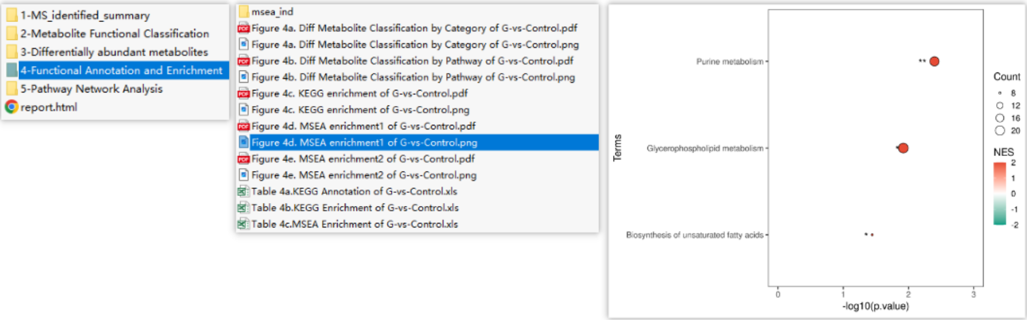

Figure 4d. MSEA enrichment1 of Case-vs-Control

MSEA(Metabolite Set Enrichment Analysis)，即代谢物集富集分析，它的基本原理是使用预定义的代谢物集（通常来自功能注释或先前实验的结果），将代谢物按照在两组样本中的差异倍数（log2fc）排序，然后检验预先设定的代谢物集合是否在这个排序表的顶端或者底端富集。不同于上述的 KEGG 富集只关注差异代谢物，MSEA 富集关注所有检测到的代谢物丰度变化，因此更容易关注到一些丰度产生细微变化的代谢物对整体通路的影响。如以

上气泡图中所示，纵轴是 KEGG 通路，横轴是-log10 转换后的 P 值，气泡大小代表该通路下富集到的代谢物数量，气泡边上的*号代表富集显著程度（*代表 P value<0.05,**代表 P value<0.01），气泡颜色代表 NES（Normalized Enrichment Score）标准化富集得分，以评估代谢集成员在排序列表的两段富集的程度。正值的 ES 表示代谢集在列表的顶部富集（即该通路下大部分代谢物丰度上调），负值代表代谢集在列表的底部富集（即该通路下大部分代谢物丰度下调）。

Figure 4e. MSEA enrichment2 of Case-vs-Control

MSEA 富集结果图展示了各通路代谢集中富集到的代谢物所处位置，其解读方式与 GSEA 富集结果图一致，图的上半部分使用柱形图展示所有代谢物按照 Log2fc 由高到低排序的结果，下半部分为 Enrichment Score 的折线图，横轴为代谢物的排序信息，纵轴为对应的 Running ES，在折线图中有个峰值，该峰值就是这个通路代谢物集的 Enrichment Score，峰值之前的代谢物就是该通路下的核心代谢物，折线的颜色代表了 ES 值的正负，绿色是负值，代表该通路下的核心代谢物大部分丰度下调；红色是正值，代表该通路下的核心代谢物大部分丰度上调。

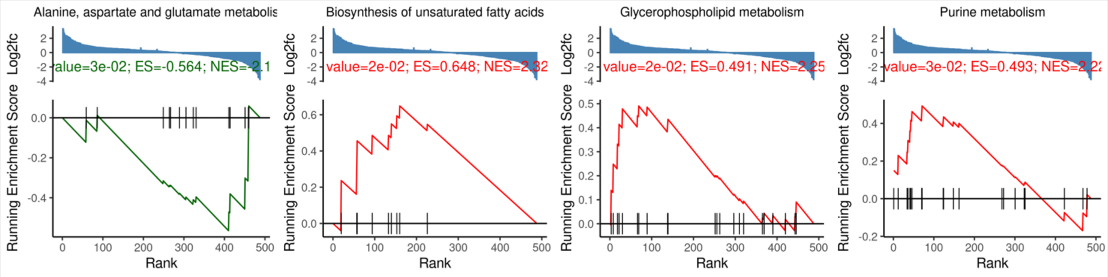

为便于客户后续分析中的图片取用，我们将每个 MSEA 富集的通路图以单图形式储存在 **4-Functional Annotation and Enrichment/msea_ind/Case-vs-Control** 路径下，如下图所示。

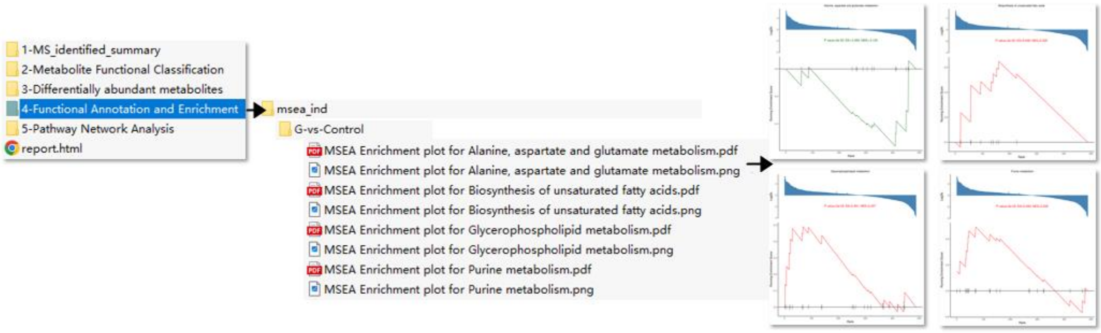

5-Pathway Network Analysis

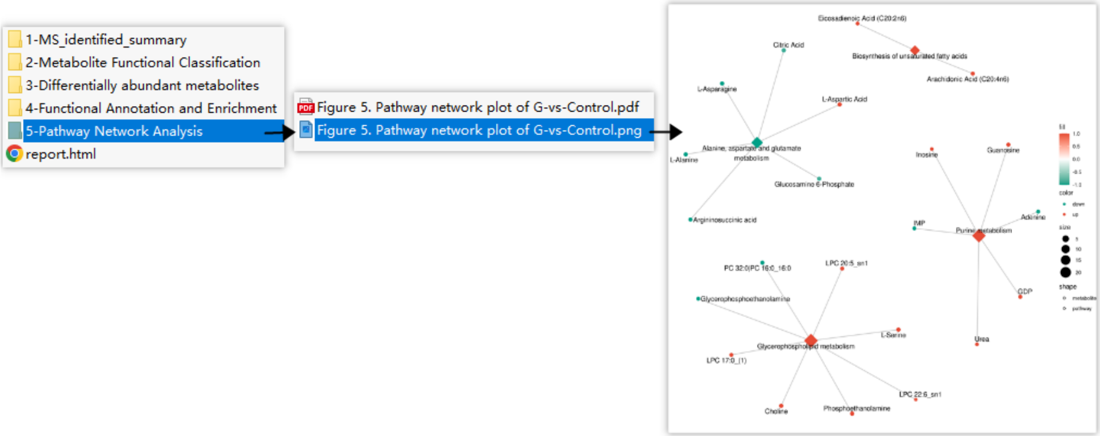

Figure 5. Pathway network plot of Case-vs-Control

基于 MSEA 富集结果，我们筛选出 P value<0.05 的通路和通路中的差异代谢物构建代谢调控网络。如上图所示：圆形代表差异代谢物，其中红色代表上调，绿色代表下调，大小表示 VIP 值；菱形表示通路，其中红色表示该通路 ES 是正值，绿色表示该通路 ES 是负值，大小表示通路中检测到代谢物的数目；连线表示代谢物参与该通路过程。
