# Supplementary material for: Multi-Omics and Experimental Validation Reveal the Protective Effect of Paeoniflorin Against Coronary Heart Disease in Mice via Inhibiting the C3-Cfd-C3aR Pathway
Source: Int J Mol Sci. 2026 Jul 13;27(14):6236. doi: 10.3390/ijms27146236 (PMC13410309; doi:10.3390/ijms27146236)
Supplement: Supplementary file 1 [file ijms-27-06236-s001.zip › Supplementary Materials/ijms-4276706_Metabolomics_Dataset/report.html]

 代谢组学分析报告 


# 代谢组学分析报告

#### 

#### 报告创建日期：2025-10-29

# 1 研究概述

代谢组学旨在对细胞、组织和生物体中的小分子化合物进行全面的定性和定量分析，以研究当机体受到内在或外部的刺激时的响应机制。近年来，代谢组学联合基因组、转录组和蛋白质组学，可以对生物系统的整体进行研究。代谢组学分析必须在分子层面，对疾病、疾病进展、治疗干预、基因修饰或环境变化相关的，即使是最轻微的变化，进行准确的定量分析。疾病、药物或环境的变化会干扰代谢通路中代谢物的浓度和代谢路径。而细胞内环境和外环境会进行相应的调整，以维持稳态。这些扰动和调整体现为细胞和生物体液的成分变化，而这些变化是具有特征性的（即“指纹”）。我们可以通过光谱/质谱技术与多变量统计方法相结合的方式来进行评估，以深入了解生物系统的分子响应机制。小分子的表达变化与观察到的表型变化密切相关，因而在功能基因组学研究中，为了确定未知基因的功能，代谢组学受到越来越多的关注。

# 2 技术路线

非靶代谢组学，利用高分辨率LC-MS/MS平台对样本中的代谢物检测，无偏向性、尽可能多地检测细胞、组织、器官、或体液等生物样本内所有的小分子代谢物，再结合本地自建代谢物数据库进行信息匹配实现代谢物鉴定，该产品包含从代谢物提取，上机检测，生信分析全套非靶代谢组学服务。技术路线如下图2-1所示：


**图2-1：** 非靶代谢组学实验流程图

# 3 分析流程

基于质谱检测得到的Raw文件，1）根据样本的来源情况构建代谢组数据库，然后采用质谱原始数据解析软件MS-DIAL进行代谢物数据库检索；2）基于实验过程中加入内标代谢物进行质控分析；3）基于HMDB和KEGG数据库对鉴定到的代谢物进行注释；4）数据预处理：代谢物的定量结果进行缺失填补、归一化和对数变换处理；5）定量重复性分析：包含标准品的CV分析、所有样本的PCA分析和PCC分析；6）差异统计分析：包含单因素的差异分析、多因素的正交偏最小二乘判别分析；7）代谢物分类统计：包含KEGG和HMDB化合物分类统计；8）基于KEGG
pathway数据库，采用Fisher’s exact
test和MSEA方法计算，差异代谢物显著富集通路。分析流程如下图3-1所示：


**图3-1：** 非靶代谢组学分析流程图

- 注:
  - 代谢物数据库包括Metlin，MoNA，GNPS，HMDB，以及自建数据库。

# 4 分析结果

## 4.1 基本信息

### 4.1.1 样本分组信息

本项目实验设计样本分组如下表所示：

### 4.1.2 代谢物检测信息

本研究对18个样本进行了非靶代谢组学检测，共检测到662种代谢物。详细结果如下表所示：

## 4.2 数据质控

质谱下机的数据，在搜库完成后，需要通过不同维度的质控评价，保证结果质量符合标准：包括内标物在不同样本的峰形态、保留时间和信号强度。如下图4-2展示了各样本中内标物质的信号强度：


**图4-2：** 标准物质在不同样本中的信号强度

## 4.3 代谢物注释

为了了解样本中代谢物的潜在功能，我们对检出的代谢物进行了简单的分类。分类方法基于HMDB和KEGG
compound数据库对代谢物的功能定义。分类统计结果如下图4-3-1和4-3-2所示：


**图4-3-1：** 检出代谢物HMDB分类统计


**图4-3-2：** 检出代谢物KEGG pathway分类统计

## 4.4 样品重复性检验

对于生物重复或技术重复样本，需要检验生物重复或技术重复样本的定量结果是否符合统计学上的一致性。这里分别采用了定量分布估计、皮尔森相关性（Pearson’s
Correlation Coefficient,
PCC）、主成分分析（PCA）和变异系数（CV）四种统计分析方法评估重复性。

### 4.4.1 定量分布图

采用小提琴图和箱线图相结合的方式展示了每个样本中代谢物丰度的整体分布情况，重复样本之间其分布形态应更相似。


**图4-4-1：** 各样本中代谢物定量分布图

### 4.4.2 变异系数（CV）

各组重复样本间代谢物定量值的变异系数绘制的累计曲线图，整体CV值越小，定量重复性越好。


**图4-4-2：** 各样本中代谢物定量分布图

### 4.4.3 主成分分析（PCA）

所有样本的代谢物定量主成分分析结果展示图，图中样本间的聚集程度代表样本的差异性大小。


**图4-4-3：** 主成分分析图

### 4.4.4 皮尔森相关性（PCC）

所有样本两两之间的皮尔森相关系数绘制的热图。此系数用于度量两组数据的线性相关程度：皮尔森相关系数接近-1为负相关，接近1为正相关，接近0为不相关。


**图4-4-4：** 皮尔森相关性系数热图

## 4.5 差异分析

差异代谢物分析包括多元统计分析和单维统计分析，其中多元统计能捕捉到具有相互关联的差异性变量有利于代谢调控网络研究；单维的统计能独立分析单个变量的
统计学意义，在数据分析中起到验证和补充的作用；因此代谢组学中使用多元统计和单维统计同时筛选到的差异变量应该是最重要和最值得关注的差异代谢物。

### 4.5.1 单因素统计分析

T-test和Wilcox-test检验方法适用于只研究一个试验因素的情况，目的在于正确判断该试验因素各处理的相对效果；用于检验两组样本的均值是否存在显著差异。其中T-test适用于小样本的正态分布的连续变量，Wilcox-test适用于大样本的任何分布形态的连续变量或秩序变量。基于本研究的样本情况，采用了T-test方法计算两组样本之差异显著性p
value。

**图4-5-1：** 差异分析火山图

### 4.5.2 多因素统计分析

正交最小偏二乘判别分析（OPLS-DA）是一种多因变量到多自变量的回归建模方法。它是一种有监督的判别分析统计方法。该方法的特点是可以去除自变量X中与分类变量Y无关的数据变化，使分类信息主要集中在一个主成分上，有效滤除与分类信息无关的噪音，从而使得模型简单易懂，提高了模型的解析能力和有效性。其在代谢组学分析中应用较多。通过建立代谢物表达量与分组关系之间的模型，OPLS-DA可以更好地获取组间差异信息，还可以对样本的分组进行预测。OPLS-DA分析结果如下图所示：

**图4-5-2：** OPLS-DA分析模型评估图

1. OPLS-DA主成分数目选择。R2Y和Q2分别用来评价OPLS-DA模型的解释能力和预测能力，R2Y和Q2的累计值越大，说明模型越稳定可靠。
2. OPLS-DA模型验证。横坐标表示置换检验的置换保留度（与原模型Y变量顺序一致的比例，置换保留度为1的点即为原模型的R2和Q2值），纵坐标表示R2Y（红色圆点）和Q2（蓝色三角）置换检验的取值，两条虚线分别表示R2和Q2的回归线。
3. S-plot是OPLS-DA分析中常用的展示图，可以了解代谢物在两组样本中的相对含量变化。S-plot图的横坐标表示主成分与代谢物的协方差，纵坐标表示主成分与代谢物的相关性系数。S-plot图一般用来挑选在正交信号校正过程中与最主要成分的相关性比较强的代谢物，从另一方面同时也可以挑选与分类变量相关性强的代谢物。越靠近两个角的代谢物重要度越强。点的颜色代表代谢物对模型的贡献度（VIP）值越大贡献度越高。
4. OPLS-DA得分图。OPLS-DA得分图通过正交旋转，过滤掉与分组无关的信息，从而能更好的区分组间差异，提高模型的效能。Comp1第一预测主成分解释度，orthogonal
   Comp1第一正交成分解释度。

### 4.5.3 差异代谢物筛选

基于上述两种统计分析方法，我们对研究中各组样本之间进行了两两比较分析，按照p.value
< 0.05,VIP > 1, abs(log2fc) >
0标准筛选出差异代谢物。分析结果如下表所示：

**图4-5-3：** 显著差异代谢物热图

### 4.5.4 差异代谢物统计表

## 4.6 差异代谢物分类

### 4.6.1 HMDB化合物分类统计

**图4-6-1：** HMDB化合物分类统计图

### 4.6.2 KEGG pathway分类统计

**图4-6-2：** KEGG pathway分类统计图

## 4.7 差异代谢物通路富集

基于KEGG通路数据库，我们对上述分析获得的差异代谢物进行了深入分析。KEGG通路数据库是连接已知分子间相互作用的信息网络，如代谢通路、复合物、生化反应等。KEGG通路主要包括：代谢、遗传信息处理、环境信息处理、细胞过程、人类疾病、药物开发等。

### 4.7.1 费希尔精确检验

此处运用Fisher’s exact test即费希尔精确检验计算显著性P
value，目的是发现差异代谢物是否在某些功能类型有显著性的富集趋势。通过气泡图的方式展现差异代谢物显著富集（P
value <
0.05）到的功能分类和通路。气泡图中给出了最显著富集的前20个分类的结果，纵轴为KEGG通路，横轴为差异代谢物在该通路中所占比例相比于鉴定代谢物所占比例的变化倍数（Fold
enrichment）的Log2转换后的数值。圆圈颜色表示富集显著性P
value，圆圈大小表示通路中的差异代谢物个数。

**图4-7-1：** 基于费希尔精确检验的KEGG通路富集分析气泡图

### 4.7.2 MSEA

MSEA(Metabolite Set Enrichment
Analysis），即代谢物集富集分析，它的基本思想是使用预定义的代谢物集（通常来自功能注释或先前实验的结果），将代谢物按照在两类样本中的差异表达程度排序，然后检验预先设定的代谢物集合是否在这个排序表的顶端或者底端富集。代谢物集合富集分析检测代谢物集合而不是单个代谢物的表达变化，因此可以包含这些细微的表达变化，预期得到更为理想的结果。

**图4-7-2：** 基于MSEA的KEGG通路富集分析气泡图
**图4-7-3：** MSEA显著富集通路(p value<0.05)折线图

### 4.7.3 代谢网络分析

基于MSEA富集结果，我们筛选出p
value<0.05的通路和通路中的差异代谢物构建代谢调控网络。如下图所示：圆形代表差异代谢物，其中红色表示上调，绿色表示下调，大小表示VIP值；菱形表示通路，其中红色表示通路被激活，绿色表示受到抑制，大小表示通路中检测代谢物的数目；连线表示代谢物参与该通路过程。

**图4-7-4：** 差异代谢物调控网络


---

# 5 材料与方法

注：仅供参考

## 5.1 实验方法

### 5.1.1 样本前处理

**血清/血浆/尿液** 1.
将血清从-80℃冰箱中取出，置于冰上或者4℃冰箱中，待血样完全解冻后，涡旋10s混匀，12000xg,
4℃离心1min后，置于冰上待用；  
2. PQC样本准备：每个血清样本取10μL混合成Pool QC
(PQC)样本，涡旋混匀；  
3. SST（System suitability
test）样本准备：将内标工作液使用10%甲醇/90%水溶液（定容溶液）稀释五倍后作为SST样本；  
4.
空白溶液：使用提取溶剂作为空白溶液（-80℃预冷半个小时以上的甲醇）；  
5. 取20μL血清、20μL
PQC、20μL空白溶液分别于2ml的离心管中，加入20μL内标工作液，之后加入110μL提取溶剂（-80℃预冷半个小时以上的甲醇），涡旋振荡提取1min；-20℃放置半小时以上；  
6. 14000xg，4℃离心10
min，将上清转移到一新的2ml离心管中，真空冷冻干燥后，复溶于100μL的
10%甲醇/90%水溶液（定容溶液），涡旋30s，超声1min，14000xg，4℃离心10
min，上清转移到进样小瓶中进质谱分析。

**贴壁细胞**

1. 细胞收取：使用1xPBS清洗细胞两次后，立即将细胞培养皿放置于干冰上，如不立即提取的话可以转移到-80℃冰箱保存（封口膜密封）；
2. 细胞破碎和代谢物提取：在细胞培养皿中加入1ml
   80%甲醇溶液（含内标混合液），使提取液覆盖整个培养皿表面，置于-80℃冰箱15min使酶充分失活。使用细胞刮棒将细胞刮取到提取液中，将细胞提取液转移到2ml离心管中；
3. 14000xg，4℃离心10 min，将上清转移到一新的2ml离心管中；
4. PQC准备：每个样本取（3）中等量的上清溶液混合为PQC样本；
5. 真空冷冻干燥后，复溶于100μL的
   10%甲醇/90%水溶液，涡旋30s，超声1min，14000xg，4℃离心10
   min，上清转移到进样小瓶中进质谱分析。

**悬浮细胞**

1. 细胞收取：300xg，4℃离心5min，弃掉上清培养基，收集于2ml
   EP管中，之后使用1xPBS清洗细胞两次，每次清洗后通过离心（300xg，4℃离心5min）得到细胞沉淀。在-80℃放置15min使酶充分失活。，如不立即提取的话可以转移到-80℃冰箱保存；
2. 细胞破碎和代谢物提取：加入200μL
   80%甲醇溶液（含内标混合液），使用超声破碎仪裂解细胞，再加入800μL，80%甲醇溶液（含内标混合液），涡旋1min；
3. 14000xg，4℃离心10 min，将上清转移到一新的2ml离心管中；
4. PQC准备：每个样本取（3）中等量的上清溶液混合为PQC样本；
5. 真空冷冻干燥后，复溶于100μL的
   10%甲醇/90%水溶液，涡旋30s，超声1min，14000xg，4℃离心10
   min，上清转移到进样小瓶中进质谱分析。

**组织样本**

1. 组织破碎：使用研钵（加入液氮）或者其他装置破碎组织；
2. 组织研磨和代谢物提取：称取5mg破碎组织于2ml
   EP管中，记录下准确重量。加入400μL
   80%甲醇溶液（含内标混合液），使用组织研磨仪充分研磨组织形成匀浆；再加入600μL
   80%甲醇溶液（含内标混合液），涡旋1min；
3. 14000xg，4℃离心10 min，将上清转移到一新的2ml离心管中；
4. PQC准备：每个样本取（3）中等量的上清溶液混合为PQC样本；
5. 真空冷冻干燥后，复溶于100μL的
   10%甲醇/90%水溶液，涡旋30s，超声1min，14000xg，4℃离心10
   min，上清转移到进样小瓶中进质谱分析。

### 5.1.2 色谱条件

**正离子模式和负离子模式同时采集**

1. 色谱柱：Waters ACQUITY BEH C18 Column (1.7 µm\* 2.1 mm \* 100
   mm);
2. 流动相A：0.1%甲酸水溶液，流动相B：0.1%甲酸乙腈/甲醇（40/60)溶液；
3. 色谱柱柱温40 °C，进样量：5μL，梯度洗脱条件如下：

**仅用于负离子模式采集**

1. 色谱柱：Waters ACQUITY BEH C18 Column (1.7 µm\* 2.1 mm \* 100
   mm);
2. 流动相A：6.5mM碳酸氢铵水溶液，流动相B：6.5mM碳酸氢铵甲醇溶液;
3. 色谱柱柱温40 °C，进样量：5μL，梯度洗脱条件如下：

### 5.1.3 LC-MS 液相质谱分析

使用装备有冷藏自动进样器（温度为10°C）和柱加热器（温度为40°C）的Vanquish
Flex UPLC，采用Waters ACQUITY BEH C18柱（1.7 µm×2.1 mm×100
mm）分离代谢物。为提高代谢物覆盖率，采用了两种移动相条件。对于条件1，溶剂A（0.1%甲酸在水中）和溶剂B（乙腈/甲醇=4/6中的0.1%甲酸）被用来在13.5分钟的梯度中洗脱代谢物，具体如下：0分钟时B为10%，流速0.25ml/min；3分钟时B为40%，流速0.25ml/min；5分钟时B为95%，流速0.25ml/min；8分钟时B为100%，流速0.6ml/min；10分钟时B为100%，流速0.6ml/min；10.5分钟时回到10%的B，流速0.25ml/min，并平衡3分钟。样品采用带加热电喷雾离子源的Q
Exactive
HF-X（QE-HF-X）质谱仪进行分析。所有数据均采用正负离子切换模式进行全扫描检测，而PQC样品还采用全扫描/ddMS2模式以获取代谢物识别和注释所需的MS2碎片化信息。对于条件2，溶剂A为6.5mM
NH4HCO3溶于水中，溶剂B为6.5mM
NH4HCO3溶于甲醇中。采用了12分钟的梯度：0分钟，流速0.25ml/min，B为10%；4分钟，流速0.25ml/min，B为40%；6分钟，流速0.25ml/min，B为95%；8分钟，流速0.4ml/min，B为99%；8.5分钟，流速0.4ml/min，B为99%；8.6分钟，流速0.25ml/min，B回到10%；12分钟，流速0.25ml/min，B为10%。数据采集仅在负离子模式下进行，所有样品均采用全扫描模式，而PQC样品还采用全扫描/ddMS2模式。全扫描设置如下：分辨率为60,000，AGC目标为1e6，最大IT为100毫秒，扫描范围为60至900
m/z。对于全扫描/ddMS2（DDA），采用AGC目标=2e5、最大IT=25毫秒和（N）CE/阶梯NCE=10、40、80V，生成前20个MS/MS谱（dd-MS2）@
15000。代谢物的检测和鉴定是通过MS-dial（版本5.1.230912）软件搜索在线数据库（MoNA、GNPS、HMDB和MS-dial数据库）和内部数据库完成的。

## 5.2 生物信息学分析方法

### 5.2.1 数据预处理

原始定量值经过Log2对数转换后，采用中位数归一化方法使每个样本中代谢物定量中位数为15。针对包含定量缺失值的代谢物，首先删除在大于30%样本定量缺失的代谢物，然后采用KNN方法对剩余缺失值进行填补。

### 5.2.2 数据质控

质控样本（PQC）由样本提取物混合制备而成，用于分析样本在相同的处理方法下的重复性。为了确保检测过程稳定性，我们会往样本中加入已知浓度的内标，内标的响应差异越小，说明检测过程越稳定，数据质量越高。

### 5.2.3 样本重复性检验

**CV值分布图**  
基于对数转换后的原始数据，计算其变异系数（Cofficient of
Variation，CV值），即原始数据标准差与原始数据平均数的比值，以反应数据离散程度。使用累计分布曲线展示不同CV值出现的频率，整体CV值越小，代表实验数据越稳定，定量重复性越好。

**主成分分析PCA**  
基于对数转换后的原始数据，使用 R 的内置统计函数 prcomp 进行 PCA
分析，设置 prcomp 函数参数 scale = True
对数据进行标准化处理，并取前两个主成分对降维后的数据进行可视化。

**皮尔森相关性PCC**  
基于对数转换后的原始数据，采用Pearson皮尔森相关性系数来衡量不同样本间的线性关系强度和方向，使用R中基础函数
cor()，并设定缺失值处理方法 use 参数为
pairwise.complete.obs，相关性结果通过R包Pheatmap以热图的形式展现。

### 5.2.4 代谢物注释与分类

基于 HMDB （Human Metabolome Database）代谢物数据库（https://hmdb.ca/metabolites ）和 KEGG（Kyoto
Encyclopedia of Genes and Genomes）通路数据库（https://www.genome.jp/kegg/pathway.html
），对所有检测到的代谢物进行注释、功能定义和分类，并使用ggplot2（version
3.4.4）包进行可视化。

### 5.2.5 鉴定差异代谢物

基于对数转换后的原始数据，结合单因素统计分析和多因素统计分析的方法来鉴定筛选差异代谢物，首先使用R中基础函数
t.test 或 wilcox.test 对数据进行单因素统计分析（T-test 或
Wilcox-test）来检验两组样本的均值是否存在显著差异（P value <
0.05），接着使用ropls
R包进行多因素统计分析–正交偏最小二乘判别分析（Orthogonal Partial Least
Square-Discriminant Analysis,OPLS-DA），默认参数orthoI设置为NA。
基于OPLS-DA模型（生物学重复 ≥ 3）得到的变量重要性投影（Variable
Importance in
Projection,VIP），可以用于初步筛选不同样本或组别间的差异代谢物（默认阈值：VIP>1），同时结合单变量分析的P
value/FDR(生物学重复 ≥
2)或差异倍数（FoldChange）值来进一步筛选出差异代谢物。一般认为，同时满足P
value<0.05，VIP>1.0的变量为差异代谢物。

### 5.2.6 代谢物通路富集

**KEGG富集分析**  
基于KEGG（Kyoto Encyclopedia of Genes and Genomes）通路数据库（https://www.genome.jp/kegg/pathway.html），使用费希尔精确检验（Fisher’s
exact test）对上一步鉴定到的差异代谢物进行KEGG通路富集，以P
value代表差异代谢物是否在某些功能通路中有显著性富集，当P value <
0.05时，该通路被认为是显著富集。而Fold
Enrich（富集倍数）为对应通路中GeneRatio与Background
Ratio的比值，该值越大表示富集程度越大。

**MSEA富集分析**  
基于所有鉴定到的代谢物和KEGG通路数据库，使用 R 包 corto（version
1.2.4）进行代谢集富集分析（Metabolite Set Enrichment
Analysis，MSEA），参数 np（Number of Permutations）设置为
500。与常见的基因集富集分析（GSEA）类似，代谢集富集分析（MSEA）不需要指定显著上调或下调的差异代谢物，其分析思路是设定一系列代谢集，每个代谢集代表某种生物学功能，将代谢组数据富集到这些代谢集，从而鉴定到有显著性差异的代谢集和相关通路。

**代谢网络分析**  
基于MSEA富集结果，我们通过设定P
value阈值（通常小于0.05）筛选显著富集的通路和通路中的差异代谢物，并使用
R 包 ggraph（version
2.1.0）绘制代谢调控网络图，用颜色和形状来区分代谢物和通路，以及代谢物的差异倍数（FC）。

## 5.3 分析软件列表及其版本

# 6 Methods and Materials（English Version）

Note: For reference only

## 6.1 Experimental Methods

### 6.1.1 Sample Preparation

**Serum/Plasma/Urine**  
1. Take serum from the -80°C freezer and place it on ice or in a 4°C
refrigerator. After the serum is completely thawed, vortex for 10
seconds, centrifuge at 12000xg, 4°C for 1 minute, then place it on ice
for later use;  
2. PQC sample preparation: Mix 10μL from each serum sample to create a
Pool QC (PQC) sample, vortex to mix well;  
3. SST (System Suitability Test) sample preparation: Dilute the internal
standard working solution five times with a 10% methanol/90% water
solution (standard solution) as the SST sample;  
4. Blank solution: Use extraction solvent as a blank solution (methanol
pre-cooled in a -80°C freezer for more than half an hour).  
5. Take 20μL serum, 20μL PQC, and 20μL blank solution each in a 2ml
centrifuge tube, add 20μL internal standard working solution, then add
110μL extraction solvent (methanol pre-cooled in a -80°C freezer for
more than half an hour), vortex and shake for 1 minute; place at -20°C
for at least half an hour; 6. Centrifuge at 14000xg, 4°C for 10 minutes,
transfer the supernatant to a new 2ml centrifuge tube, vacuum
freeze-dry, resuspend in 100μL of 10% methanol/90% water solution
(standard solution), vortex for 30 seconds, sonicate for 1 minute,
centrifuge at 14000xg, 4°C for 10 minutes, transfer the supernatant to a
sample vial for mass spectrometry analysis.

**Adherent Cells**  
1. Cell collection: Wash cells twice with 1xPBS, then immediately place
the cell culture dish on dry ice. If not extracted immediately, it can
be stored in a -80°C freezer (sealed with film);  
2. Cell lysis and metabolite extraction: Add 1ml of 80% methanol
solution (containing a mixture of internal standards) to the cell
culture dish to cover the entire surface of the dish, place in a -80°C
freezer for 15 minutes to inactivate enzymes. Scrape the cells into the
extraction solution using a cell scraper and transfer the cell extract
to a 2ml centrifuge tube;  
3. Centrifuge at 14000xg, 4°C for 10 minutes, transfer the supernatant
to a new 2ml centrifuge tube;  
4. PQC preparation: Mix an equal amount of supernatant from each sample
in step (3) to create a PQC sample;  
5. After vacuum freeze-drying, resuspend in 100μL of 10% methanol/90%
water solution, vortex for 30 seconds, sonicate for 1 minute, centrifuge
at 14000xg, 4°C for 10 minutes, transfer the supernatant to a sample
vial for mass spectrometry analysis.

**Suspension Cells**  
1. Cell collection: Centrifuge at 300xg, 4°C for 5 minutes, discard the
supernatant medium, collect in a 2ml EP tube, then wash cells twice with
1xPBS. After each wash, obtain cell pellets by centrifugation (300xg,
4°C for 5 minutes). Place at -80°C for 15 minutes to fully deactivate
enzymes. If not extracted immediately, it can be stored in a -80°C
freezer  
2. Cell lysis and metabolite extraction: Add 200μL of 80% methanol
solution (containing a mixture of internal standards), use an ultrasonic
cell disruptor to lyse cells, then add 800μL of 80% methanol solution
(containing a mixture of internal standards), vortex for 1 minute;  
3. Centrifuge at 14000xg, 4°C for 10 minutes, transfer the supernatant
to a new 2ml centrifuge tube;  
4. PQC preparation: Mix an equal amount of supernatant from each sample
in step (3) to create a PQC sample;  
5. After vacuum freeze-drying, resuspend in 100μL of 10% methanol/90%
water solution, vortex for 30 seconds, sonicate for 1 minute, centrifuge
at 14000xg, 4°C for 10 minutes, transfer the supernatant to a sample
vial for mass spectrometry analysis.

**Tissue Samples**  
1. Tissue disruption: Crush tissue using a mortar and pestle (add liquid
nitrogen) or other devices;  
2. Tissue grinding and metabolite extraction: Weigh 5mg of crushed
tissue into a 2ml EP tube and record the exact weight. Add 400μL of 80%
methanol solution (containing a mixture of internal standards), use a
tissue homogenizer to thoroughly grind the tissue into a homogenate;
then add 600μL of 80% methanol solution (containing a mixture of
internal standards), vortex for 1 minute;  
3. Centrifuge at 14000xg, 4°C for 10 minutes, transfer the supernatant
to a new 2ml centrifuge tube;  
4. PQC preparation: Mix an equal amount of supernatant from each sample
in step (3) to create a PQC sample;  
5. After vacuum freeze-drying, resuspend in 100μL of 10% methanol/90%
water solution, vortex for 30 seconds, sonicate for 1 minute, centrifuge
at 14000xg, 4°C for 10 minutes, transfer the supernatant to a sample
vial for mass spectrometry analysis.

### 6.1.2 Conditions for Chromatography

**Simultaneous Collection in Positive Ion Mode and Negative Ion
Mode**  
1. Chromatography column: Waters ACQUITY BEH C18 Column (1.7 µm\* 2.1 mm
\* 100 mm);  
2. Mobile phase A: 0.1% formic acid in water, mobile phase B: 0.1%
formic acid acetonitrile/methanol (40/60) solution;  
3. Column temperature: 40 °C, injection volume: 5μL, gradient elution
conditions as follows:

**Collection in Negative Ion Mode only**  
1. Chromatography column：Waters ACQUITY BEH C18 Column (1.7 µm\* 2.1 mm
\* 100 mm);  
2. Mobile phase A：6.5mMAmmonium bicarbonate aqueous solution, mobile
phase B: 6.5mM ammonium bicarbonate methanol solution;  
3. Column twmperature: 40 °C，Injection volumn：5μL，gradient elution
conditions as follows：

### 6.1.3 LC-MS Analysis

Metabolites were separated using a Waters ACQUITY BEH C18 Column (1.7
µmx2.1 mm x100 mm) on a Vanquish Flex UPLC equipped with a refrigerated
autosampler (10°C) and column heater (40°C).

Two moblie phase condition was used to improve the metabolite
coverage. For condition 1, solvent A (0.1% formic acid in water) and
solvent B (0.1% formic acid in acetonitrile/methanol=4/6) were used to
elute the metabolites with a 13.5 min gradient, as follows: 10% B at 0
min, 0.25ml/min; 40% B at 3 min, 0.25ml/min; 95 % B at 5 min,
0.25ml/min; 100 % B at 8 min, 0.6ml/min; 100 % B at 10 min, 0.6ml/min;
and back to 10 % B at 10.5 min, 0.25ml/min; and equilibrate for 3min.
Samples were analyzed using a Q Exactive HF-X (QE-HF-X) mass
spectrometry equipped with a heated electro-spray ionization (HESI)
source. All the data was acquired in positive and negtive switching mode
using Full scan detection, and the PQC were also analyzed with Full
scan/ddMS2 to acquire MS2 fragementation for metaoblite identificaiton
and annotation. For conditon 2, solvent A was 6.5mM NH4HCO3 in water,
solvent B was 6.5mM NH4HCO3 in methanol. The 12 min gradient was
employed: 0 min, 0.25ml/min, 10% B; 4 min, 0.25ml/min, 40%B; 6 min,
0.25ml/min, 95%B; 8 min, 0.4ml/min, 99%B; 8.5 min, 0.4ml/min, 99%B; 8.6
min, 0.25ml/min, 10%B; 12 min, 0.25 ml/min, 10%B. Data acquisiton were
performed only in negative mode, and Full scan was used for all the
samples, and Full scan/ddMS2 was also used for PQC samples.

The Full Scan settings were as follows: 60,000 resolution, AGC
target, 1e6; Maximum IT, 100 ms; scan range, 60 to 900 m/z. For Full
scan/ddMS2(DDA), Top 20 MS/MS spectral (dd-MS2) @ 15000 were generated
with AGC target = 2e5, Maximum IT=25 ms, and (N)CE/stepped NCE = 10, 40,
80v. Metabolites detection and identification were performed using
MS-dial (ver.5.1.230912) by searching against online database (MoNA,
GNPS, HMDB and MS-dial database) and in-house database.

## 6.2 Bioinformatics Analysis

### 6.2.1 Quality Control of Data

Quality control samples (PQC) are prepared by mixing sample extracts
to analyze the reproducibility of samples under the same processing
conditions. To ensure the stability of the detection process, internal
standards with known concentrations are added to the samples. The
smaller the response difference of the internal standards, the more
stable the detection process and the higher the data quality.

### 6.2.2 Sample Reproducibility Test

**CV Distribution Plot**  
Based on the log2 transformed raw data, the cofficient of variation (CV)
values are calculated, which is the ratio of the standard deviation to
the mean of the original data, reflecting the degree of data dispersion.
The cumulative distribution curve is used to display the frequency of
different CV values. A smaller overall CV value indicates greater
stability of experimental data and better quantitative
repeatability.

**Principal Component Analysis (PCA)**  
Using the built-in statistical function `prcomp` in Rstudio
on the log2 transformed raw data for PCA analysis, with the
`scale = True` parameter set to standardize the data. The
visualization is performed by plotting the reduced dimensionality data
based on the first two principal components.

**Pearson Correlation Coefficient (PCC)**  
Based on the log2 transformed raw data, Pearson correlation coefficient
is used to measure the strength and direction of linear relationships
between different samples. The basic function `cor()` in R is
utilized, with the `use` parameter set to
`pairwise.complete.obs` for handling missing values. The
correlation results are presented in the form of a heatmap using the R
package `Pheatmap (version 1.0.12)`.

### 6.2.3 Metabolite Annotation & Classification

Utilizing the Human Metabolome Database (HMDB) and the Kyoto
Encyclopedia of Genes and Genomes (KEGG) pathway database, all detected
metabolites are annotated, functionally defined, and classified.
Visualization is performed using the
`ggplot2 (version 3.4.4)` package.

### 6.2.4 Differential Metabolite Analaysis

Based on the log2 transformed raw data, differential metabolites are
identified using both univariate and multivariate statistical analysis.
Initially, the `t.test` function in R is employed for
univariate analysis to test for significant differences in means between
two groups of samples (P value < 0.05). Subsequently, multivariate
analysis (Orthogonal Partial Least Square-Discriminant Analysis ,
OPLS-DA) are carried out using `ropls` R package with
`orthoI = NA` set as default parameter. The Variable
Importance in Projection (VIP) obtained from the OPLS-DA model
(biological replicates ≥ 3) is used to preliminarily screen differential
metabolites between different samples or groups (default threshold: VIP
> 1). In addition, the P value/FDR (biological replicates ≥ 2) or
fold change (FC) values from univariate analysis further aids in the
selection of differential metabolites. Generally, variables meeting both
criteria of P value < 0.05 and VIP > 1.0 are considered as
differential metabolites.

### 6.2.5 Differential Metabolite Pathway Enrichment

**KEGG Enrichment Analysis**  
Utilizing the KEGG pathway database, Fisher’s exact test is employed to
perform KEGG pathway enrichment analysis on the identified differential
metabolites. The P value indicates whether differential metabolites are
significantly enriched in certain functional pathways. A pathway is
considered significantly enriched when P value < 0.05. The Fold
Enrichment represents the ratio of GeneRatio to Background Ratio in the
corresponding pathway, with higher values indicating greater
enrichment.

**MSEA Enrichment Analysis**  
Based on all identified metabolites and the KEGG pathway database,
Metabolite Set Enrichment Analysis (MSEA) is conducted using the R
package corto (version 1.2.4), with the parameter `np`
(Number of Permutations) set to `500`. Similar to Gene Set
Enrichment Analysis (GSEA), MSEA does not require specifying
significantly upregulated or downregulated differential metabolites.
Instead, it sets a series of metabolite sets, each representing a
biological function, for enrichment analysis. Significant differential
metabolite sets and associated pathways are identified through this
analysis.

**Metabolic Network Analysis**  
Based on MSEA enrichment results, significant enriched pathways and
differential metabolites within pathways are filtered using a P value
threshold (usually <0.05), and a metabolic regulation network diagram
is constructed using the R package `ggraph (version 2.1.0)`.
Colors and shapes are used to differentiate metabolites and pathways, as
well as indicate the fold change (FC) of metabolites.
