# Supplementary material for: Multi-Omics and Experimental Validation Reveal the Protective Effect of Paeoniflorin Against Coronary Heart Disease in Mice via Inhibiting the C3-Cfd-C3aR Pathway
Source: Int J Mol Sci. 2026 Jul 13;27(14):6236. doi: 10.3390/ijms27146236 (PMC13410309; doi:10.3390/ijms27146236)

广科安德 Astral 定量蛋白组分析

结果文件说明文档

目录

结果文件说明 3

一. 分析思路 3

二. 文档组成 3

三. 文档说明 6

结果文件说明

一． 分析思路

此报告中涵盖了本次实验基本数据结果、质控分析、差异分析以及后续富集分析的结果。分析思路如下：1）质控分析：评估本次实验数据的好坏，当实验数据质量过关后进行下一步分析；2）差异分析：根据客户的实验设计进行差异分析，并依照阈值来筛选差异表达蛋白；3）功能注释：将差异表达蛋白做不同层面的功能注释；4）功能分类：分析差异表达蛋白在不同功能中所占的比例；5）富集分析：找到差异表达蛋白显著富集的功能；6）功能富集聚类分析；7）蛋白互作网络分析；8）KEGG 通路展示。

二． 文档组成

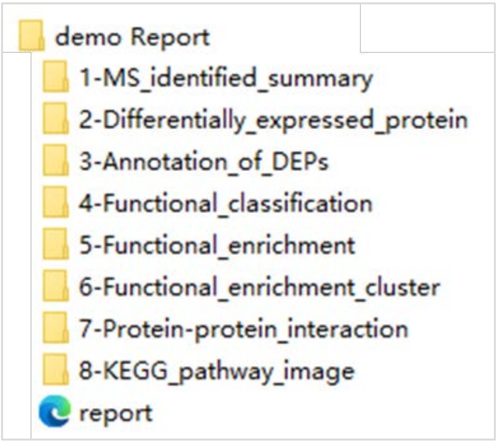

1) **质谱数据基本分析文件**

   - 文档名称：1-MS_identified summary
   - 文档内容：蛋白质谱鉴定与定量信息、鉴定质控信息、定量质控信息。

2) **差异表达蛋白分析文件**

- 文档名称：2-Differentially_expressed_protein
- 文档内容：实验各个比较组显著差异表达蛋白、差异表达蛋白分布图。

3) **鉴定和差异表达蛋白注释文件**

- 文档名称：3-Annotation_of_DEPs
- 文档内容：所有鉴定到的蛋白和差异表达蛋白的亚细胞结构定位、GO、KEGG 通路、蛋白结构域注释信息。

4) **差异表达蛋白功能分类文件**

- 文档名称：4-Functional_classification
- 文档内容：差异表达蛋白 GO 二级分类统计、亚细胞结构分类统计。

5) **差异表达蛋白功能富集分析文件**

- 文档名称：5-Functional_enrichment
- 文档内容：差异表达蛋白所有 GO 分类富集分析、KEGG 通路富集分析、蛋白结构域富集分析。

6) **差异表达蛋白功能富集聚类分析文件**

- 文档名称：6-Functional_enrichment_cluster
- 文档内容： 不同比较组差异表达蛋白的 GO 富集的聚类分析、KEGG 通路富集聚类分析、蛋白结构域富集聚类分析。

7) **差异表达蛋白互作网络分析文件**

- 文档名称：7-Protein-protein_interaction
- 文档内容：将鉴定到的差异表达蛋白与蛋白互作网络数据库比对，将结果进行可视化展示。

8) **显著富集 KEGG 通路可视化文件**

- 文档名称：8-KEGG_pathway_iamge
- 文档内容：在差异表达蛋白 KEGG 通路富集分析中得到的显著富集的通路，以网页和图片形式可视化的通路示意图。在通路示意图中用指定颜色标注了差异表达的蛋白。

9) **项目报告文档**

- 文档名称：report.html
- 文档内容： 项目信息，客户信息，实验流程，生物信息分析结果展示，实验材料与方法，生物信息分析方法。

三． 文档说明

0_Report.html

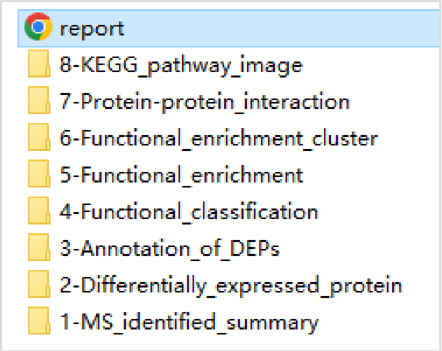

html 格式的项目报告文档内容如下：1 研究概述、2 技术路线、3 分析流程、4 分析结果(涵盖了本项目蛋白质组学分析结果包括质控结果，蛋白功能注释，定量分析，样本重复性检验，差异蛋白筛选及其后续一系列功能富集、聚类和蛋白互作等分析结果)、5 材料与方法。

1-MS_identified_summary（质谱数据基本分析文件）

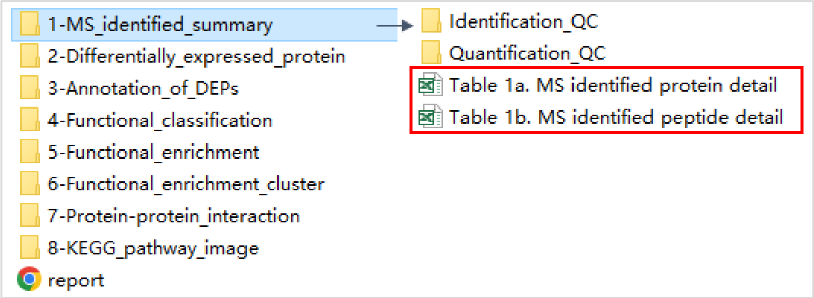

本文件夹下包含两个 Excel 格式文件，分别是质谱鉴定的蛋白定量数据（Table 1a. MS identified protein detail）和肽段定量数据（Table 1b. MS identified peptide detail）。为了消除系统误差和允许不同样本/蛋白间进行对比，我们对定量数据进行了一系列的中心化和归一化处理，处理细节详见 html 格式报告中 4.4 蛋白定量分析。

1）Table 1a 内容如下：

Protein accession	Gene name	Protein description	PSMs	Peptides	Unique peptides	Intensity	Case1	Case2	Case3
A0A075B6H7	IGKV3-7	Probable non-functional immunoglobulin kappa variable 3-7	5	1	1			2.25	0.322
A0A075B6H9	IGLV4-69	Immunoglobulin lambda variable 4-69	11	2	2	187817.3	-0.329	1.818	0.402

Protein accession	蛋白在数据库中的编号，数据库主要来源有 UniProtKB/NCBI，其他物种特有的数据库（拟南芥数据库:TAIR），以及客户提供的数据库。
Gene name	蛋白对应的基因名称。
Protein description	数据库中蛋白功能的详细描述信息。
PSMs	二级谱图和理论谱图的匹配数。
Peptides	鉴定到的对应蛋白的肽段数量。
Unique peptides	特异性肽段数量。
Intensity	蛋白丰度的总和。
Case1-3	样本分组。

2）Table 1b 内容如下：

Protein accession	Charge	Modifications	Mass error [ppm]	Missed cleavages	Experiment	Intensity	Sequence	id	Length	Proteotypic	PSMs	Peptides	Unique peptides
P55011	2	AAAAAAAAAAAAAAAAGAGAGAK	-1.384382312	0	Case1	3698.44	AAAAAAAAAAAAAAAAGAGAGAK	AAAAAAAAAAAAAAAAGAGAGAK2	22	1	67	19	18
P55036	3	AAAASAAEAGIATTGTEDSDDALLK	2.618833573	0	Case1	2507.5	AAAASAAEAGIATTGTEDSDDALLK	AAAASAAEAGIATTGTEDSDDALLK3	25	1	48	9	7
P05154	2	AAAATGTIFTFR	3.991445195	0	Case1	6788.5	AAAATGTIFTFR	AAAATGTIFTFR2	12	1	61	14	14
Q6P2E9	2	AAADTLQGPMQAAYR	-0.312318712	0	Case1	6254.15	AAADTLQGPMQAAYR	AAADTLQGPMQAAYR2	15	1	98	28	28

Protein accession:	蛋白在数据库中的编号，数据库主要来源有 UniProtKB/NCBI，其他物种特有的数据库（拟南芥数据库：TAIR），以及客户提供的数据库。
Charge	肽段带电荷数。
Modifications	肽段上是否有修饰（仅适用于蛋白修饰组学分析）。
Mass error	检测到的肽段实际质量与理论质量之差，值越小代表鉴定越可靠。
Experiment	不同实验批次。
intensity	肽段的总丰度。
Sequence	肽段。
id	肽段的 id。
Length	肽段长度。
Proteotypic	肽段序列所属蛋白是否唯一。
PSMs	二级谱图和理论谱图的匹配数。
Peptides	鉴定到的对应该蛋白的肽段数量。
Unique peptides	特异性肽段数量。

3）Identification_QC

文件夹下含有蛋白定性后不同维度下的质控分析结果，QC1-QC4 为不同质控维度：包括肽段质量误差分布、肽段长度分布、肽段氨基酸频率、肽段信号强度分布等。图表详细信息见 html 格式的报告。

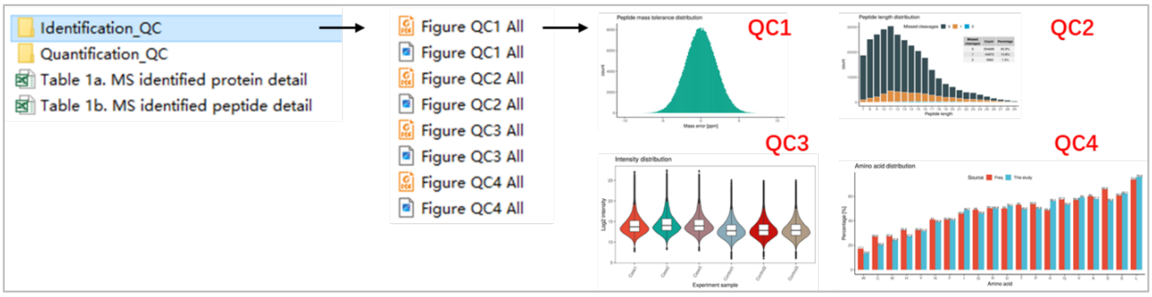

4）Quantification_QC

Quantification_QC 文件夹下包含蛋白定量的质控分析结果，由于项目有生物或技术重复样本，因此需要进行定量重复性检验分析，图 A-D 既是从不同维度下检验生物学重复或技术重复样本的定量结果是否符合统计学上的一致性。

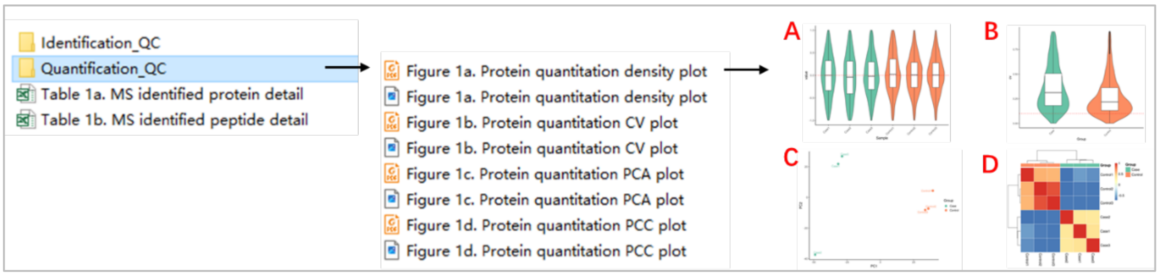

(图 A)：横坐标为不同样本，纵坐标为归一化后的蛋白相对定量值分布，理论上不同样本应处于同一水平线上。**(图 B)**：各组重复样本间蛋白定量值的相对标准差（RSD）绘制的箱线图，整体 RSD 值越小，定量重复性越好。**(图 C)**：所有样本的蛋白定量主成分分析结果展示图，图中样本间的聚集程度代表样本间的差异性大小。图中可以看出相同组别的生物重复样本更倾向于聚集在一起，因此样本重复性较好。**(图 D)**：所有样本两两之间皮尔森相关系数热图。皮尔森系数是度量两组数据相关程度的值。当皮尔森系数越接近-1 为负相关（蓝色），越接近 1 为正相关（红色）。越接近 0 为不相关。

2-Differentially_expressed_protein （差异表达蛋白的分析）

差异表达蛋白的分析与筛选根据生物重复样本量以及客户需求而变化，详细信息见html格式报告。通常在差异分析后，我们使用两个条件来筛选差异蛋白：P 值和变化倍数（Foldchange)。根据以往文献，筛选得到的差异表达蛋白个数控制在整体可定量蛋白数的10%左右，且尽量保证差异表达蛋白的个数大于 100 个，有利于后期的生物学统计分析。报告中，我们默认使用的阈值是 Pvalue<0.05, |Foldchange| > 2，具体阈值会根据客户数据变化，见 html 格式的报告。

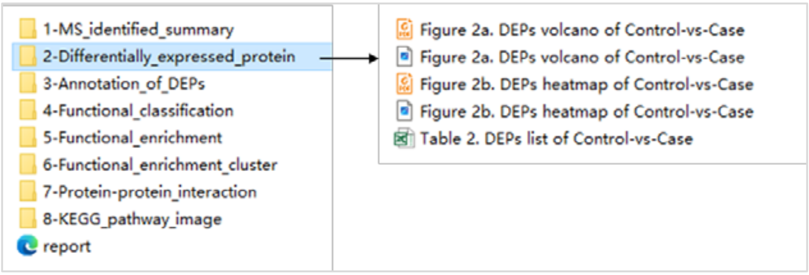

2-Differentially_expressed_protein 文件夹中的 Excel 格式文件是不同组别进行差异分析得到的差异表达蛋白。每个 Excel 文件中包含以下几列——Protein accession（蛋白编号），Gene name（基因名称），Protein description（蛋白描述），蛋白相对表达量，log2FC（log2 转换后的差异倍数），P.value（P 值），adj.P.Val（BH 校正后的 P 值），Regulation（蛋白上下调描述）。

Protein accession	Gene name	Protein description	Control1	Control2	Control3	Case1	Case2	Case3	log2FC	P.Value	adj.P.Val	Regulation
A0A075B6H7	IGKV3-7	Probable non-functional immunoglobulin kappa variable 3-7	-1.248	-0.182	-1.096		2.25	0.322	-2.128	0.0161	0.0528	unchanged
A0A075B6R9	IGKV2D-24	Probable non-functional immunoglobulin kappa variable 2D-24	-2.578	-1.422	-2.327	1.164	1.787	3.421	-4.233	0.000242	0.00662	down-regulated
P08603	CFH	Complement factor H	0.079	0.317	0.572	-0.886	0.583	-0.619	0.63	0.159	0.258	unchanged
P08637	FCGR3A	Low affinity immunoglobulin gamma Fc region receptor III-A	-1.751		-1.003	0.743	2.059		-2.778	0.0042	0.0233	down-regulated
P08648	ITGA5	Integrin alpha-5	0.334	0.125	0.174	-0.227	-0.154	-0.207	0.407	0.0497	0.112	unchanged
P08670	VIM	Vimentin	1.148	1.136	1.538	-1.007	-1.062	-1.708	2.533	2.94E-05	0.00251	up-regulated

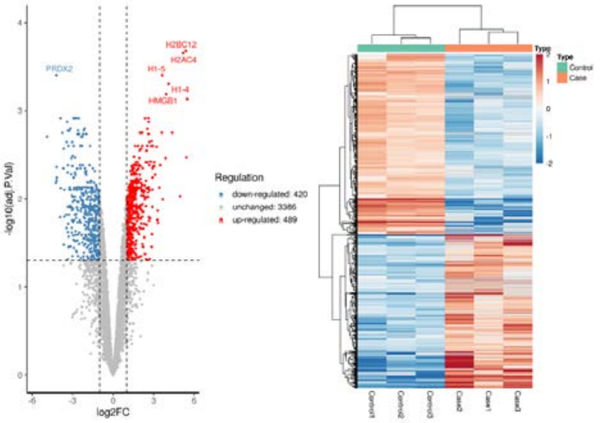

左图为差异表达分析的火山图，横轴为对数转换后的差异倍数（log2FC），纵轴为 log10 调整后的 P 值，红色为显著上调蛋白，蓝色为显著下调蛋白。右图是用筛选出的差异表达蛋白绘制的热图，横轴的每一格代表一个样本（颜色代表组别），纵轴的每一格代表一个差异表达蛋白，格子颜色的深浅代表蛋白相对表达量，红色代表表达量高，蓝色代表表达量低。

3-Annotation_of_DEPs （蛋白功能注释）

为了透彻了解不同蛋白质的功能特性，我们对鉴定到的差异表达蛋白进行全方位的功能注释。包含基因本体论（Gene Ontology，GO）、蛋白结构域（Protein domain）、KEGG 通路、KOG 功能分类以及亚细胞结构定位（Subcellular localization）等方面进行了详细的注释。

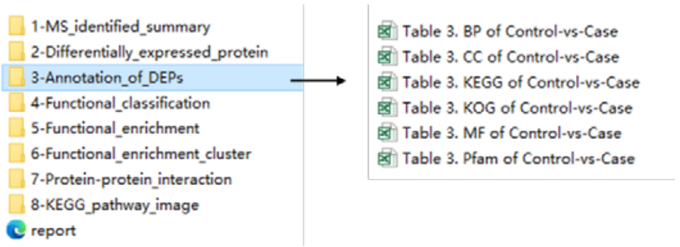

-GO 注释

（Annotation_of_DEPs 文件夹下的命名中含有 BP、CC 和 MF 的为 GO 注释的文件）。GO 又名基因本论，是一个主要的生物信息学分析方法，用于统一的表述所有物种中基因和基因产物的属性。 GO 主要分为 3 个大类：（1）Cellular Component 细胞组

成： 细胞的组成是一个广义的结构对象；这可能是一个亚细胞结构(如内质网或核)又或者是一个蛋白生产组件(如核糖体,蛋白酶体或蛋白二聚体). （2）Molecular Function 分子功能： 分子功能描述生物活动,如发生在分子水平上的催化反应或蛋白结合。(3）Biological Process 生物过程： 生物过程是一系列分子功能相互配合的事件。

蛋白的 GO 注释信息主要来源于 UniProt-GOA 数据库(http://www.ebi.ac.uk/GOA/)，首先, 将蛋白的 ID 转换为 UniProtKB 数据库的 ID，然后根据 UniProKB 的 ID 在 UniProt-GOA 中找到对应的 GO 注释信息。最后，每个蛋白的分类根据 Gene Ontology annotation 可以分为 3 大类: 生物过程（BP)，分子功能（MF)，细胞组成（CC)。以 BP 表格为例 （其他功能注释表格形式类似，因此不做赘述)

Protein accession	Category	ID	GO levels	Terms	Gene nam	Protein descripti	Control1	Control2	Control3	Case1	Case2	Case3	log2FC	P.Value	adj.P.Val	Regulation
A0A075B6I0	Biological process	GO:005089	2	response t	IGLV8-61	Immunoglobulir	-1.026		-2.237	1.251	1.709	0.379	-2.744	0.00218	0.0164	down-regulated
A0A075B6I0	Biological process	GO:000695	3	immune re	IGLV8-61	Immunoglobulir	-1.026		-2.237	1.251	1.709	0.379	-2.744	0.00218	0.0164	down-regulated
A0A075B6I0	Biological process	GO:000237	2	immune sy	IGLV8-61	Immunoglobulir	-1.026		-2.237	1.251	1.709	0.379	-2.744	0.00218	0.0164	down-regulated
A0A075B6I0	Biological process	GO:000815	1	biological_	IGLV8-61	Immunoglobulir	-1.026		-2.237	1.251	1.709	0.379	-2.744	0.00218	0.0164	down-regulated
A0A075B6P5	Biological process	GO:000815	1	biological_	IGKV2-28	Immunoglobulir	-0.805	-1.353	-1.594	-0.344	2.247	1.895	-2.517	0.00739	0.0326	down-regulated
A0A075B6P5	Biological process	GO:000237	2	immune sy	IGKV2-28	Immunoglobulir	-0.805	-1.353	-1.594	-0.344	2.247	1.895	-2.517	0.00739	0.0326	down-regulated

Protein accession	蛋白在数据库中的编号
Category	GO 的分类，一共分为三大类
ID	该 GO term 在 GO 分类体系中的特定编号。
GO levels	该 GO term 在 GO 分类系统中的层级，层级数字越大，分类描述越具体。
Terms	GO 条目。
Gene name	基因名。
Protein Description	蛋白的详细描述。
Control1/Case1	样本信息。
Log2FC	蛋白表达差异倍数的对数转换。
P.value	使用 Fisher 检验得到的富集检验 P 值。
adj.P.Val	使用 BH（Benjamini &Hochberg）法矫正过的 P 值。
Regulation	差异蛋白表达模式（up-regulated 为上调，down-regulated 为下调)。

-KEGG 注释

（Annotation_of_DEPs 文件夹下的命名中含有 KEGG 的为 KEGG 注释的文件）。KEGG 是连接已知分子间相互作用的信息网络，如代谢通路、复合物，生化反应。KEGG 途径主要包括:代谢、遗传信息处理、环境信息处理、细胞过程,人类疾病、药物开发等。

-蛋白结构域注释（Pfam/InterPro）

（Annotation_of_DEPs 文件夹下的命名中含有 Pfam 的为 Pfam 注释的文件）。蛋白质结构域是在进化上非常保守的一段蛋白序列、结构或者功能独立存在的其他蛋白质链。每个域形成一个紧凑的三维结构,通常可以独立稳定和折叠。许多蛋白质包含多个结构域，一个结构域可能出现在各种不同的蛋白质。简言之，蛋白结构域是指在不同蛋白质分子中重复出现的某些组分，具有相似的序列、结构和功能，是蛋白质进化的单元。结构域的长度约在 25 个氨基酸和 500 个氨基酸长度之间。 Pfam 数据库是一个很常用的蛋白家族数据库，是以 UniProt 数据库收录的蛋白数据为依据创建的，在本项目中被用于对差异表达蛋白进行结构域注释。

-KOG 功能分类注释

（Annotation_of_DEPs 文件夹下的命名中含有 KOG 的为 KOG 注释的文件）。KOG 是 Clusters of orthologous groups for eukaryotic complete genomes （真核生物蛋白相邻类的聚簇）的缩写。构成每个 KOG 的蛋白都是被假定为来自于一个祖先蛋白，或是 orthologs 或是 paralogs。Orthologs 是指来自于不同物种的由垂直家系（物种形成）进化而来的蛋白，并且通常保留与原始蛋白相同的功能。而 paralogs 是那些在一定物种中的来源于基因复制的蛋白，可能会进化出新的与原来有关的功能。通过鉴定蛋白与数据库的比对，可以很好的预测蛋白质的功能。数据库链接：EggNOG Database | Orthology predictions and functional annnotaion (embl.de)

-亚细胞结构定位

真核生物组织细胞中的蛋白，依据与其结合的膜结构的差异，被定位到细胞内的各种元件上。

4-Functional_classification （差异表达蛋白功能分类）

为了阐释差异表达蛋白的生物学作用，我们根据不同比较组以及其上调或下调的表达模式，将筛选得到的差异表达蛋白及其功能注释结果进行分类统计。4-Functional_classification 文件夹内包含两类文件，一是展现统计结果的 Excel 文件，二是将统计结果可视化的 pdf/png 文件。

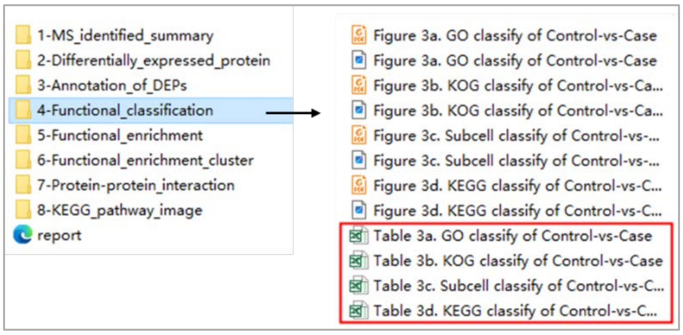

以 GO 注释中的 BP（生物过程）为例：Category 是功能注释的名称或分类（包括 Biological process，Molecular function，Cellular component，KEGG，COG，Pfam 和 subcell 等），Terms 是蛋白功能注释项，Regulation 则是差异蛋白的表达模式（上调 or 下调），Freq 是指该注释项下有多少差异蛋白富集。

Category	Terms	Regulation	Freq
Biological process	locomotion	down-regulated	3
Biological process	biological adhesion	down-regulated	4
Biological process	signaling	down-regulated	4
Biological process	developmental proce	down-regulated	7
Biological process	immune system proce	down-regulated	7
Biological process	metabolic process	down-regulated	7
Biological process	localization	down-regulated	8

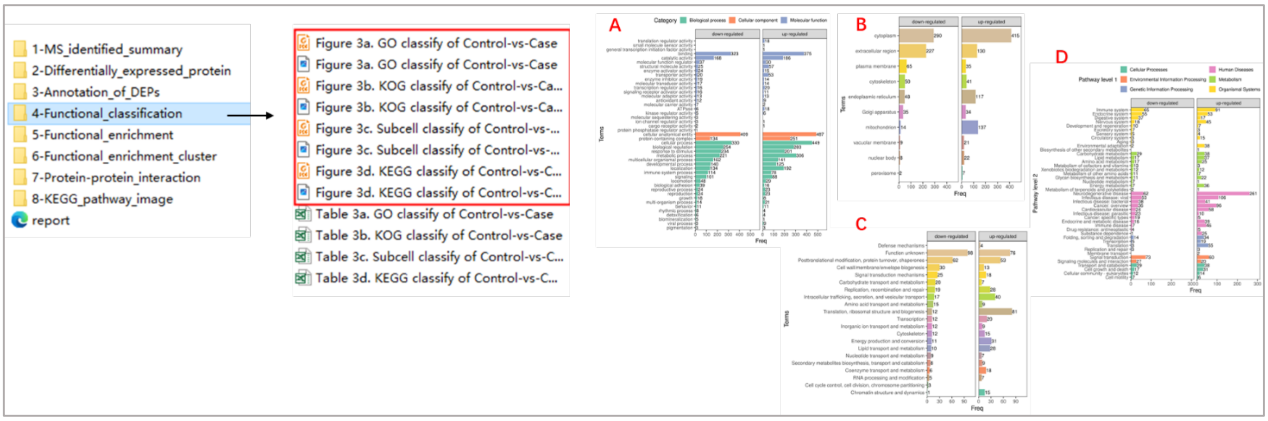

-A．GO 注释结果

根据表达模式将差异表达蛋白分为两类（down-regulated 下调，up-regulated 上调），Category 中的不同颜色代表 GO 注释的三大类，分别是生物过程，细胞组分，分子功能。纵轴是富集到的 GO 注释项（Terms），横轴是特定 Terms 下差异蛋白的数量（Freq）。

-B．KEGG 注释结果：

根据表达模式将差异表达蛋白分为两类（down-regulated 下调，up-regulated 上调），Pathway level 1 中不同颜色代表 KEGG 通路信息的不同分级。纵轴是富集到的 KEGG 通路（Terms），横轴是特定 Terms 中上调/下调差异蛋白的数量（Freq）。

-C．KOG 注释结果：

根据表达模式将差异表达蛋白分为两类（down-regulated 下调，up-regulated 上调），横轴 Freq 代表蛋白数量，不同 Terms 代表差异表达蛋白与 KOG 数据库比对后预测的蛋白功能，可为后续分析以及猜想提供参考。

-D.Subcell 亚细胞结构定位注释结果：

根据表达模式将差异表达蛋白分为两类（down-regulated 下调，up-regulated 上调），横轴 Freq 代表蛋白数量，不同 Terms 代表差异表达蛋白通过软件预测的亚细胞定位，可为后续分析以及猜想提供参考。

5-Functional_enrichment （差异表达蛋白功能富集）

我们对各比较组中的差异表达蛋白分别进行了 GO 分类、KEGG 通路和蛋白结构域三个层面的富集分析（此处运用 Fisher’ s exact test 即费希尔精确检验计算显著性 P value），目的是发现差异表达蛋白是否在某些功能类型有显著性的富集趋势。4-Functional_enrichment 文件夹内包含两类文件，一是展现富集结果的 Excel 文件，二是将统计结果可视化的 pdf/png 文件。

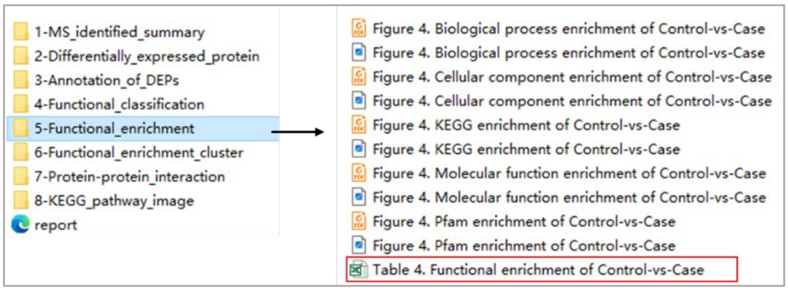

以 GO 富集结果为例：Excel 表格中 Fun Category 代表富集的数据库类型，此处是 GO 中的生物过程（Biological process），Regulation 代表差异蛋白的表达模式（up/down-regulated 上调、下调），Terms 指富集到的注释信息，mapping 代表富集到同一个 term 的蛋白数量，Fold enrich 代表富集倍数，p.value 和 fdr 都可代表富集显著性，fdr 是根据差异蛋白数矫正过的 P 值，Gene names 表示该通路中的蛋白基因名，Proteins 表示该通路中蛋白的 Uniprot ID。由于蛋白组学中能够鉴定到的差异蛋白数量有限，因此我们使用原始 p 值<0.05 来筛选即可。

Fun Category	Regulation	Terms	mapping	Fold enrich	p.value	fdr	Gene names	Proteins
Biological process	down-regulated	actin cytoskeleton organization	10	1.1	0.422	1	XIRP2;EPB41L2	A4UGR9;O43491;P02549;P05787;P
Biological process	down-regulated	actin filament bundle assembly	2	3.06	0.139	1	ELN;FSCN1	P15502;Q16658
Biological process	down-regulated	actin filament bundle organization	4	2.73	0.059	1	SPTA1;ELN;AD	P02549;P15502;P35612;Q16658
Biological process	down-regulated	actin filament organization	4	1.06	0.527	1	SPTA1;ELN;AD	P02549;P15502;P35612;Q16658
Biological process	down-regulated	actin filament-based process	18	1.28	0.167	1	XIRP2;EPB41L2	A4UGR9;O43491;O60234;O75044;

通过气泡图的方式展现差异表达蛋白显著富集 (P value < 0.05) 到的功能分类和通路。气泡图中给出了最显著富集的前 20 个分类的结果，纵轴为功能分类或通路，横轴为差异表达蛋白在该功能类型中所占比例相比于鉴定蛋白所占比例的变化倍数（Fold enrichment）

的 Log2 转换后的数值。圆圈颜色表示富集显著性 P value，颜色越深越显著，圆圈大小则表示功能类或通路中差异蛋白的个数。

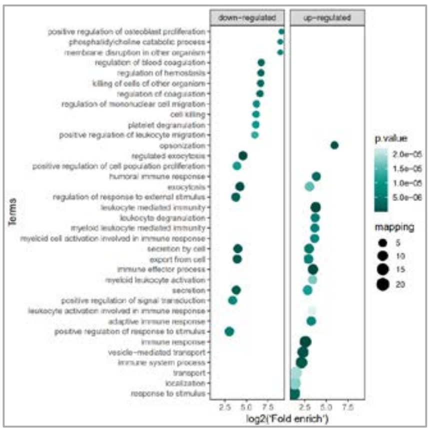

6-Functional_enrichment_cluster （功能富集聚类分析）

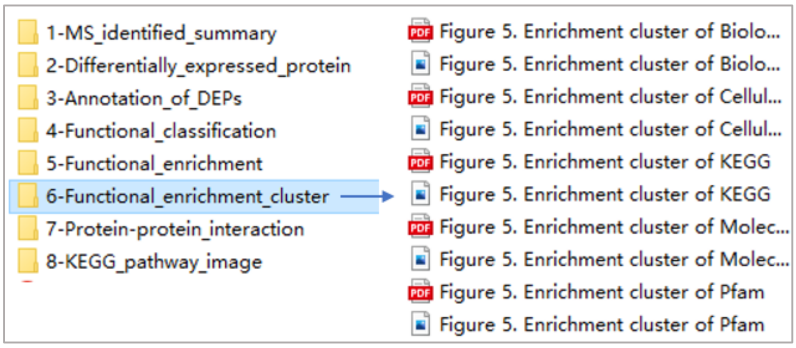

当实验设计较为复杂，比较组较多时，为了更加直观地查看不同比较组功能富集的结果，并找出共同趋势，我们将各个比较组的差异蛋白富集到的 term 取了并集，使用 p.value 绘制热图，如下图所示：

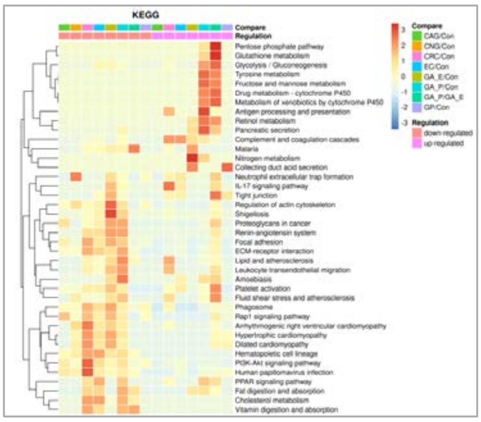

此图为 KEGG 富集聚类分析得到的热图（heatmap），横向代表不同比较组中的差异表达蛋白，纵向为差异表达所富集到的相关生理过程与功能（GO、KEGG pathway、Protein domain）的描述。不同比较组的差异表达蛋白与功能描述对应的色块表示富集程度强弱，即-log10 对数转换后的 p 值大小，越红代表功能富集越显著。热图上方的注释行（Compare 和 Regulation）分别代表了实验的比较组别和差异蛋白表达模式。从图中我们可以观察到，某些通路常出现在上调蛋白中，而某些通路则在下调蛋白中共享，可以为下一步猜想和实验验证奠定基础。

7-Protein-protein_interaction （蛋白互作网络分析）

为了进一步了解差异表达蛋白之间的功能关系以及其潜在的生物学影响，我们将鉴定到的差异蛋白做了蛋白互作网络分析。7-Protein-protein_interaction 文件夹下有两类文件，一类是蛋白互作网络分析的参数文件（此处可忽略），另一类则是 html 格式文件（主要的结果文件）。

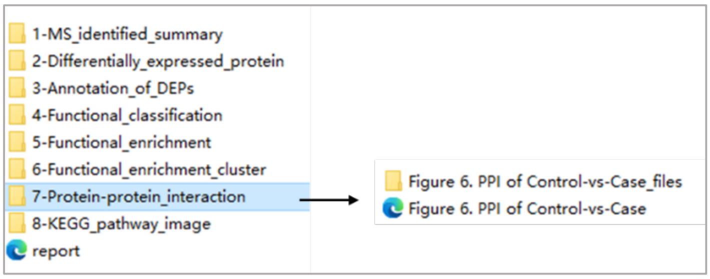

我们将各比较组中根据差异倍数筛选得到的差异蛋白数据库编号或蛋白序列，通过与 STRING（v.11.0）蛋白互作网络数据库比对后，按照 confidence score > 0.7 （high confidence）提取得到差异蛋白互作关系。然后通过 R package “visNetwork”工具对差异蛋白互作网络进行可视化展示。如下图所示：图中圆圈表示差异蛋白，不同颜色代表蛋白的差异表达情况（绿色为下调蛋白，橙色为上调蛋白），圆圈越大，代表的差异倍数越大。为

了能清晰的展示蛋白与蛋白之间的互作关系，我们筛选出了前 50 个互作关系最紧密的蛋白绘制了蛋白互作网络，如下图：

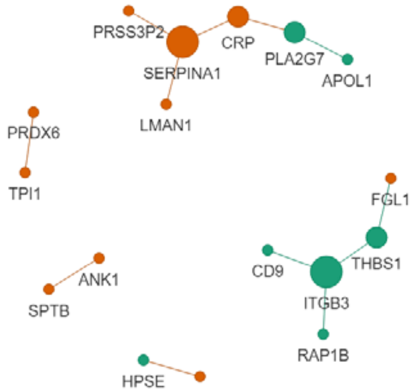

8-KEGG_pathway_image（KEGG 通路图）

为了深入探究差异蛋白在富集到的通路中所处的位置和重要性，我们将 KEGG 富集分析得到的 KEGG 通路进行了 html 网页形式以及图片形式的可视化展现。如下图，8-KEGG_pathway_image 下的子文件夹按照各对比组命名。

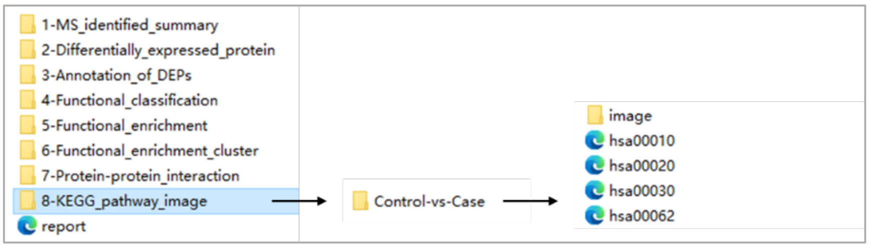

每个文件夹下包含上下调蛋白富集到的通路可视化结果。可视化效果图如下：红色代表上调表达蛋白，蓝色代表下调表达蛋白。网页版可点击某一蛋白了解更加详细内容。

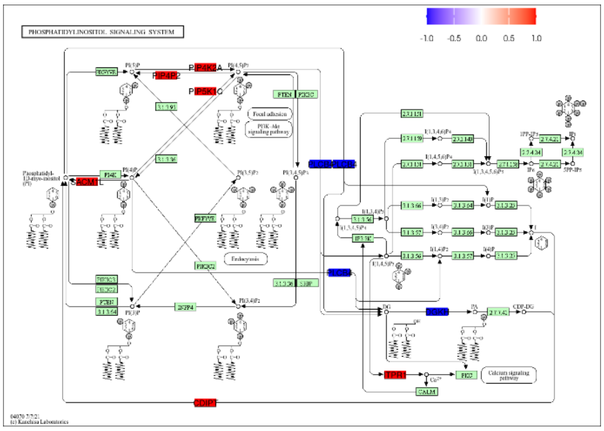

Supplement: Supplementary file 1 [file ijms-27-06236-s001.zip › Supplementary Materials/ijms-4276706_Proteomics_Dataset/Report description.pdf]
