# Supplementary material for: Multi-Omics and Experimental Validation Reveal the Protective Effect of Paeoniflorin Against Coronary Heart Disease in Mice via Inhibiting the C3-Cfd-C3aR Pathway
Source: Int J Mol Sci. 2026 Jul 13;27(14):6236. doi: 10.3390/ijms27146236 (PMC13410309; doi:10.3390/ijms27146236)
Supplement: Supplementary file 1 [file ijms-27-06236-s001.zip › Supplementary Materials/ijms-4276706_Proteomics_Dataset/report.html]

 Astral DIA 定量蛋白质组学分析报告 


# Astral DIA 定量蛋白质组学分析报告

#### 

#### 报告创建日期：2025-10-29

# 1 研究概述

蛋白质组学本质上是在大规模水平上研究不同条件下蛋白质的特征，包括蛋白质的表达水平、翻译后的修饰、蛋白质与蛋白质相互作用等，由此获得蛋白质水平上的关于疾病机理、细胞代谢等过程的整体而全面的认识。基于质谱平台的定量蛋白质组学技术是一种用于精确定量蛋白质样本中存在的不同蛋白质的方法。该方法通常使用质谱技术，如液相色谱-串联质谱（LC-MS/MS）和数据依赖性采集（DDA）或数据独立采集（DIA），来同时鉴定和定量蛋白质样本中存在的不同蛋白质。与非定量蛋白质组学技术相比，基于质谱平台的定量蛋白质组学技术需要使用已知浓度的标准品进行定量，因此适用于需要精确定量的研究和已知样本的分析。该技术可以用于研究蛋白质表达、信号转导、代谢途径等方面。

# 2 技术路线

本项目采用基于Orbitrap
Astral质谱仪检测的DIA蛋白质组学技术路线，对样本进行高深度的蛋白鉴定和高准确性的蛋白定量。Orbitrap
Astral质谱仪在检测通量、蛋白组覆盖深度、灵敏度及精准定量等多个维度具有卓越性能，与DIA定量技术的完美结合，检测更稳定，大大推动蛋白质组学在肿瘤、疾病、生长发育、植物等领域的应用。Astral
高分辨质谱仪集合了四级杆质量分析器、 Orbitrap 质量分析器和
Astral质量分析器，显著扩大了研究的范围和视角。前端（离子源至四极杆）最大限度提高仪器的灵敏度和耐用性。
Orbitrap 质量分析器能够以高分辨率采集全景全扫描数据。 Astral
质量分析器能够快速（高达 200Hz）、灵敏地采集高动态范围 HRAM， 与
Orbitrap 质量分析器的采集完全同步。因此， Astral
质谱仪在多种数据采集策略下都具有出色表现。 Astral 质谱仪+全扫描 DIA
技术，极大提升了质谱的鉴定能力。

本项目实验设计样本分组如下表2-1.

**表2-1：** 实验设计样本分组表

DIA定量蛋白质组实验示例流程见下图2-1，主要包括：1）从组织或体液样本中提取蛋白；2）将获得的蛋白采用胰蛋白酶消化为肽段；3将肽段脱盐处理后在Orbitrap
Astral质谱仪上进行DIA (data independent acquisition)
检测；4）对所有LC-MSMS检测得到的所有DIA原始数据导入DIA-NN进行分析。5）基于DIA-NN输出的蛋白定量结果进行生物信息分析流程。详细数据分析流程参见图3-1。


**图2-1：** Astral高深度DIA定量蛋白质实验流程图

# 3 分析流程

基于质谱检测得到的Raw文件，1）根据样本的来源情况构建样本特异性蛋白数据库，然后采用DIA数据分析软件DIA-NN进行蛋白数据库检索；2）基于数据库搜索的结果进行肽段和蛋白水平的质控分析；3）对鉴定到的蛋白进行常见功能注释，包括GO、KEGG、
Protein
domain、COG/KOG、STRING数据库注释等；4）蛋白的定量分析：包含定量分布统计和重复性分析；5）差异蛋白筛选：包含差异显著性检验，差异数目统计，差异蛋白火山图和表达量热图；6）差异蛋白功能分类统计分析：包含GO二级分类、KEGG通路二级分类、亚细胞定位分类、COG/KOG分类统计；7）基于分类统计结果，采用Fisher’s
exact
test方法计算，差异蛋白显著富集生物功能或通路；8）通过蛋白互作网络（Protein-protein
interaction, PPI）分析，筛选实验条件关键调控蛋白。分析流程如下图：

- 注:
  - 蛋白质数据库由客户提供或来源于UniProt数据库。若UniProt数据库中没有该物种，需要客户指定数据库来源（其他物种特异性数据库、转录组测序等）；
  - 若项目无生物学或技术重复，则不能进行定量重复性分析；
  - 本项目具体分析内容以此报告为准。

# 4 分析结果

## 4.1 DIA-NN工作流程

DIA-NN 利用深度神经网络 (DNNs)
来区分真实信号和噪声，并采用了新的量化和干扰校正策略。DIA-NN
的工作流程是完全自动化的 (图 1)，DIA-NN
使用内源性肽进行保留时间校正，自动执行质量校正，自动确定搜索参数（保留时间窗口和提取质量精度）。

DIA-NN 工作流程以肽段中心法开始，该方法基于母离子的集合
(对来自一个母离子的多个碎片离子进行注释)，这些母离子可由谱图库单独提供，也可经由
silico 中的 DIA-NN 从蛋白质序列数据库 (无库模式)
中自动生成。然后，DIA-NN 生成一个阴性对照库
(即诱饵母离子)，提取每个目标离子或诱饵母离子的谱图，并在母离子的假定保留时间附近识别由母离子和碎片离子洗脱曲线组成的假定洗脱峰。用一组能反映峰特征（包括碎片离子的共洗脱、质量精度、检测到的离子和参考谱图的相似性）的分数来描述每个洗脱峰。使用线性分类器的迭代训练为每个母离子选择最佳候选峰。下表是搜库结果经过数据过滤后的鉴定的肽段和蛋白数总体情况：

**DIA-NN 工作流程图**

**表3-1** 蛋白质组学分析结果统计

- 表头说明:
  - MS/MS: 总谱图数，质谱分析产生的二级谱图数；
  - MS/MS Identified: 有效谱图数，与数据库匹配的二级谱图数；
  - MS/MS Identified [%]: 有效谱图率，有效谱图数占总谱图数的比例；
  - Peptides Identified: 鉴定肽段数，有效谱图解析出的肽段数；
  - Unique peptides:
    特异性肽段数，鉴定到的肽段中为蛋白特异性的肽段数；
  - Identified proteins：鉴定蛋白数，特异性肽段对应的蛋白数。

## 4.2 数据质控

质谱下机的数据，在搜库完成后，需要通过不同维度的质控评价，保证结果质量符合标准：包括肽段质量误差分布、肽段长度分布、肽段氨基酸频率、肽段信号强度分布。

### 4.2.1 肽段质量误差

实际检测质量与理论质量之间的误差总体呈正态分布。根据质谱仪型号的不同，分布范围有所差别。通常肽段质量误差分布范围要求在±10ppm内。

### 4.2.2 肽段长度分布

大部分肽段分布在7-20个氨基酸，符合基于酶解和质谱碎裂方式的一般规律。质谱鉴定到的肽段长度分布符合质控要求。蛋白通过胰蛋白酶酶切为肽段的过程中容许漏切率小于15%。

### 4.2.3 肽段信号强度分布

不同样本标签的信号强度代表样本中肽段量的总体分布。采用非标定量技术路线时，所有样本采用相同的参数进行质谱分析，理论上不同样本来源的信号强度总体分布应基本一致。质控要求信号强度中值偏差小于20%.

### 4.2.4 氨基酸频率分布

理论上鉴定肽段的氨基酸频率分布与数据库中氨基酸频率分布应基本一致。由于实验流程的选择偏差，会对精氨酸R、赖氨酸K(蛋白通过胰蛋白酶酶切过程)和半胱氨酸C(蛋白提取的还原烷基化过程)的频率分布造成一定影响。质控要求K/R的频率与数据库差异小于30%，C的频率差异小于10%。

## 4.3 蛋白功能注释

为了透彻了解不同蛋白质的功能特性，我们对鉴定到的蛋白质进行全方位的功能注释。包含基因本体论（Gene
Ontology，GO）、蛋白结构域（Protein
domain）、KEGG通路、COG/KOG功能分类以及亚细胞结构定位（Subcellular
localization）等方面进行了详细的注释。注释结果详见文件夹“3-Annotation\_of\_DEPs/”

## 4.4 蛋白定量分析

搜库结果中给出了每个蛋白在不同样本中的信号强度值（Reporter
Intensity）。根据这个信息通过如下步骤计算出蛋白的相对定量值：

1. 首先蛋白在不同样本中的原始信号强度（Intensity）通过Log2的对数变换，计算公式如下：（其中i表示样本，j表示蛋白）
   \(I\_{ij}=Log2(Intensity\_{ij})\)
2. 然后对于每个蛋白，将Log2对数转换后的值（I）进行中心化变换，得到蛋白的相对定量值（U）。计算公式如下：其中i表示样本，j表示蛋白。计算公式:
   \(U\_{ij} = I\_{ij}-Mean(I\_j)\)
3. 为了消除不同样本在质谱检测中上样量的系统误差，蛋白相对定量值（U）需要采用中位数归一化方法进行校正（NR）。计算公式:
   \(NR\_{ij} = U\_{ij} - Median(Ui)\)

## 4.5 样品重复性检验

对于生物重复或技术重复样本，需要检验生物重复或技术重复样本的定量结果是否符合统计学上的一致性。这里分别采用了皮尔森相关性（Pearson’s
Correlation Coefficient,
PCC）、主成分分析（PCA）和相对标准差（RSD）三种统计分析方法评估重复性。

### 4.5.1 PCC

所有样本两两之间的皮尔森相关系数绘制的热图。此系数用于度量两组数据的线性相关程度：皮尔森相关系数接近-1为负相关，接近1为正相关，接近0为不相关。

### 4.5.2 PCA

所有样本的蛋白定量主成分分析结果展示图，图中样本间的聚集程度代表样本的差异性大小。

### 4.5.3 RSD

各组重复样本间蛋白定量值的相对标准差（RSD）绘制的箱线图，整体RSD值越小，定量重复性越好。

## 4.6 差异蛋白筛选

- **三次重复及以上：**
  - 首先挑出需要比较的样本，将每个蛋白在多次重复样本中的相对定量值均值之比作为差异倍数（Fold
    Change，FC）。例如计算样本A与样本B之间蛋白差异倍数。计算公式如下：其中R表示蛋白相对定量值，i表示样本，k表示蛋白。
    \(FC\_{A/B,k} = Mean(R\_{ik},i\in A) /
    Mean(R\_{ik},i\in B)\)
  - 为了判断差异的显著性，将每个蛋白在比较组样本中的相对定量值进行T检验，计算相应的P
    value，以此作为显著性指标，默认P value <
    0.05。为了让检验数据符合T检验需求的正态分布。检验前，蛋白相对定量值需要经过Log2对数转换。计算公式如下：
    \(P\_{ik} = T.test(Log(R\_{ik}, i\in A),
    Log(R\_{ik}, i\in B))\)
- **两次重复：**
  - 首先挑出需要比较的样本，将每个蛋白在两次重复比较组中的相对定量值均值作为差异倍数（Fold
    Change，FC）。例如计算样本分组A与样本分组B之间蛋白差异倍数。计算公式如下：其中R表示蛋白相对定量值，i表示样本，k表示蛋白。
    \(FC\_{A/B,k} = Mean((R\_{ik},i\in
    A)/(R\_{ik},i\in B))\)
  - 为了判断差异的显著性，计算每个蛋白在两个比较组中的标准变异系数（CV）作为显著性指标，默认CV
    <0.1。计算公式如下：
    \(CV = SD(A\_{1k}/B\_{1k}, A\_{2k}/B\_{2k}) /
    Mean(A\_{1k}/B\_{1k}, A\_{2k}/B\_{2k})\)
- **无重复：**
  - 首先挑出需要比较的样本，将每个蛋白在样本中的相对定量值之比作为差异倍数（Fold
    Change，FC）。例如计算样本A与样本B之间蛋白的差异倍数。计算公式如下：其中R表示蛋白相对定量值，k表示蛋白。
    \(FC\_{A/B,k} = R\_{Ak} / R\_{Bk}\)
  - 为了判断差异的显著性，将每个蛋白的肽段相对定量值在比较组中进行T检验，计算相应的P
    value，以此作为显著性指标，默认P value <
    0.05。为了让检验数据符合T检验要求的正态分布。检验前，肽段相对定量值需要经过Log2对数转换。计算公式如下：其中U表示肽段相对定量值，j表示肽段。
    \(P\_k = T.test(Log(U\_{Aj}), Log(U\_{Bj}),
    j∈k)\)

通过上述差异分析，当 后的P value < 0.05或CV < 0.05
，以差异表达量变化超过1.3作为显著上调的变化阈值，小于1/1.3作为显著下调的变化阈值。本项目所有差异表达的蛋白汇总数据详见下表:

### 4.6.1 差异蛋白统计表

### 4.6.2 差异蛋白详细表

### 4.6.3 差异蛋白火山图

### 4.6.4 差异蛋白热图

## 4.7 差异蛋白功能分类

### 4.7.1 GO二级分类

Gene
Ontology（GO）即基因本体论，是一个重要的生物信息学分析方法和工具，用于表述基因和基因产物的各种属性。GO注释分为3大类：生物过程（Biological
Process），细胞组分（Cellular Component）和分子功能（Molecular
Function），从不同角度阐释蛋白的生物学作用。我们将GO分类中的三大类分别进行了差异蛋白富集分析。

### 4.7.2 KEGG通路分类

#### 4.7.2.1 KEGG pathway image

### 4.7.3 COG功能分类

COG，即Clusters of Orthologous Groups of
proteins。构成每个COG的蛋白都是被假定为来自于一个祖先蛋白，Orthologs是指来自于不同物种、由垂直家系（物种形成）进化而来的蛋白，并且典型的保留与原始蛋白相同的功能。COG的中文释义即“同源蛋白簇”。COG分为两类，一类是原核生物，另一类是真核生物。原核生物的一般称为COG数据库；真核生物的一般称为KOG数据库。我们通过数据库比对，将差异表达蛋白进行了COG/KOG功能分类统计。详见：4-Functional\_classification/

### 4.7.4 亚细胞定位

真核生物组织细胞中的蛋白，依据与其结合的膜结构的差异，被定位到细胞内的各种元件上。基于此，我们使用WolF
Psort软件对蛋白进行亚细胞结构注释。相对于真核细胞，原核细胞一般没有细胞内膜，没有核膜包裹的成型细胞核，细胞内无染色体，DNA链未螺旋化，并以游离的形式存在于细胞质中，细胞质内也无任何有膜的细胞器（如线粒体或叶绿体）。基于此，我们使用PSORTb（v3.0）软件对蛋白进行亚细胞结构注释

## 4.8 差异蛋白功能富集

我们对各比较组中的差异表达蛋白分别进行了GO分类、KEGG通路和蛋白结构域三个层面的富集分析（此处运用Fisher’s
exact test即费希尔精确检验计算显著性P
value），目的是发现差异表达蛋白是否在某些功能类型有显著性的富集趋势。通过气泡图的方式展现差异表达蛋白显著富集（P
value <
0.05）到的功能分类和通路。气泡图中给出了最显著富集的前20个分类的结果，纵轴为功能分类或通路，横轴为差异表达蛋白在该功能类型中所占比例相比于鉴定蛋白所占比例的变化倍数（Fold
enrichment）的Log2转换后的数值。圆圈颜色表示富集显著性P
value，圆圈大小表示功能类或通路中的差异蛋白个数。详见：5-Functional\_enrichment/

### 4.8.1 GO富集

Gene
Ontology（GO）即基因本体论，是一个重要的生物信息学分析方法和工具，用于表述基因和基因产物的各种属性。GO注释分为3大类：生物过程（Biological
Process），细胞组分（Cellular Component）和分子功能（Molecular
Function），从不同角度阐释蛋白的生物学作用。我们将GO分类中的三大类分别进行了差异蛋白富集分析。

#### 4.8.1.1 Biological process

#### 4.8.1.2 Molecular function

#### 4.8.1.3 Cellular component

### 4.8.2 KEGG通路富集

KEGG是连接已知分子间相互作用的信息网络，如代谢通路、复合物、生化反应等。KEGG通路主要包括：代谢、遗传信息处理、环境信息处理、细胞过程、人类疾病、药物开发等。

### 4.8.3 蛋白结构域富集

蛋白质结构域是指在不同蛋白质分子中重复出现的某些组分，具有相似的序列、结构和功能，是蛋白质进化的单元。结构域的长度通常在25到500个氨基酸长度之间。

## 4.9 功能富集聚类分析

## 4.10 蛋白互作网络分析

将比较组中根据差异分析结果筛选得到的差异蛋白数据库编号或蛋白序列，通过与STRING（v.11.0）蛋白互作网络数据库比对后，按照confidence
score > 0.7 （high confidence）提取得到差异蛋白互作关系。然后通过R
package
“visNetwork”工具对差异蛋白互作网络进行可视化展示。如下图所示：图中圆圈表示差异蛋白，不同颜色代表蛋白的差异表达情况（绿色为下调蛋白，红色为上调蛋白）。为了能清晰的展示蛋白与蛋白之间的互作关系，我们筛选出了前50个互作关系最紧密的蛋白绘制了蛋白互作网络，详见：7-Protein-protein\_interaction/。

---

# 5 材料与方法

## 5.1 实验方法

### 5.1.1 蛋白提取

细胞类样品：样品从-80℃冰箱中取出，插入冰上解冻后，将事先预混的裂解液（8
M尿素，1%蛋白酶抑制剂）缓慢加入到样品管中，在超声波破碎仪中超声裂解。离心（4℃，15000
g，10
min），去除细胞残渣，上清液转移至新离心管，使用BCA法测定上清蛋白浓度。  
动物组织：样品从－80
°C冰箱中取出，在液氮预冷的研钵中放入适量组织样品充分研磨成粉，收集粉末于新离心管中，将事先预混的裂解液（8
M尿素，1%蛋白酶抑制剂）缓慢加入到样品管中，在超声波破碎仪中超声裂解。离心（4℃，15000
g，10
min），去除细胞残渣，上清液转移至新离心管，使用BCA法测定上清蛋白浓度。  
血清项目除高丰度法：样品从-80℃冰箱中取出，插入冰上解冻后，离心（4℃，15000
g，10 min），去除沉淀，上清液转移至新离心管中，按照Thermo
公司生产的Pierce™ Top 14 Abundant Protein Depletion Spin Columns Kit
试剂盒说明书所述进行去除高丰度蛋白，最后使用BSA法进行蛋白浓度测定。  
上清类（超滤法）：样品从-80℃冰箱中取出，置于冰上，待其完全化冻后，离心（4℃，15000
g，10
min），去除杂质。转移上清液至超滤离心管(millipore)中，离心浓缩（4℃，5000
g）上清液至0.5
mL，加入等体积8M尿素置换两次，最后使用BCA法进行蛋白浓度测定。

### 5.1.2 酶解

沉淀酶解：根据蛋白浓度测定值，每个样品各取等量蛋白，并用裂解液将体积补齐至一致，缓慢加入适量体积TCA使其终浓度为20%，充分涡旋混匀后，4℃静置沉淀2h。离心（4℃，15000
g，5
min），收集蛋白沉淀，用-20°C预冷的丙酮洗涤沉淀3次。待丙酮挥发干后，将沉淀悬浮于100
mM的TEAB中，水浴超声复溶，以1:
50的比例（蛋白酶：蛋白，m/m）加入胰蛋白酶，37℃酶解过夜。加入终浓度为5
mM二硫苏糖醇（DTT）56℃还原30 min。之后加入终浓度为15
mM碘乙酰胺（IAA）室温避光反应15
min，反应完成后，加入适量体积的10%TFA对样品进行酸化处理使其pH值在2-3之间，用Strata
X（Phenomenex）除盐后真空冷冻干燥后备用，肽段经水充分复溶后用Pierce™
Quantitative Peptide Assays & Standards试剂盒（Thermo
Scientific）进行肽段定量。
FASP酶解：根据蛋白浓度测定值，每个样品各取等量蛋白，用裂解液将体积补齐至一致，加入终浓度为5
mM二硫苏糖醇（DTT）56℃还原30 min，之后加入终浓度为15
mM碘乙酰胺（IAA）室温避光反应15
min。将反应好的样品转移至超滤离心管(millipore)中，离心（室温，12000
g，20 min），用8 M 尿素置换3次，再用25mM
HEPES置换尿素3次，第一次以1:50的比例（蛋白酶：蛋白，m/m）加入胰蛋白酶，37℃酶解过夜，第二次以1:100的比例（蛋白酶：蛋白，m/m）加入胰蛋白酶，37℃酶解4h。离心（室温，12000
g，20 min）后用纯净水冲洗超滤管内壁，离心（室温，12000 g，20
min）后合并两次离出液。后加入适量体积的10%TFA对样品进行酸化处理使其pH值在2-3之间，用Strata
X（Phenomenex）除盐后真空冷冻干燥，肽段经水充分复溶后用Pierce™
Quantitative Peptide Assays & Standards试剂盒（Thermo
Scientific）进行肽段定量。

### 5.1.3 液相色谱-质谱联用分析

DIA 分析采用纳升流速 Vanquish Neo
系统(赛默飞)进行色谱分离，纳升级高效液相色谱分离后的样品用 Astral
高分辨质谱仪（Thermo Scientific）进行
DIA（数据非依赖）质谱分析。检测模式：正离子，母离子扫描范围为
380-980m/z，一级质谱分辨率为 240000 at 200 m/z， Normalized AGC Target
为500%， Maximum IT 为 5ms。 MS2 采用 DIA 数据采集模式，设置 299
个扫描窗口， Isolation Window为 2m/z， HCD Collision Energy 为 25ev，
Normalized AGC Target 为 500%， Maximum IT 为 3ms。

### 5.1.4 数据分析

DIA 数据采用 DIA-NN 软件进行数据处理。软件参数设置如下：酶为
trypsin， max miss cleavage
site为1，固定修饰为Carbamidomethyl(C)，动态修饰设定为Oxidation(M)和Acetyl(Protein
N-term)，数据库检索鉴定到的蛋白必须通过设定的过滤参数 FDR<1%。

## 5.2 生物信息学分析方法

### 5.2.1 蛋白注释方法

**Gene Ontology注释**

Gene
Ontology分析（GO分析），是一种能够将基因与基因产物（如蛋白质）的各项信息有机的联系在一起进而提供统计学信息的生物信息学分析方法。在蛋白质组学项目中，GO主要有下列用途：1、作为蛋白及基因的各项信息的数据库；
2、提供蛋白及基因的各种信息，并按照信息对蛋白及基因进行分类；3、作为一个工具，为项目中的所有蛋白提供最全面的信息注释和分类服务。GO分析主要包括三个方面：1、细胞组分：指细胞的特定成分，在GO系统中它应当是细胞中较大组分的组成元件。比如解剖学上的某些细胞结构（糙面内质网、细胞核等），或一系列基因产物，如一些复杂成分的基础结构（核糖体，蛋白二聚体等）。2、分子功能：主要描述分子的化学活性，比如能够在分子层面表现出来的催化活性或结合活性。3、生物过程：生物体内一系列分子有序的执行某项特定功能，被称为生物过程。GO注释是将鉴定到的蛋白利用eggnog-mapper软件（v2.0）进行注释分析。该软件基于EggNOG数据库，目前最新版是第5版，涵盖了5090个生物（477个真核生物、4445个代表性细菌和168个古细菌）以及2502个病毒的全基因组编码蛋白质序列。这里提取每个蛋白质注释结果中的GO
ID，然后按照细胞组分，分子功能和生物过程对蛋白进行功能分类。

**蛋白结构域注释**

蛋白的结构域是指蛋白质中在序列上具有保守性，且一般情况下可以独立行使功能的特定蛋白区域，是分子功能的结构元件，一般由25至500个氨基酸构成。这些区域在空间上相对紧凑、结构上相对稳定、能够独立的被折叠为功能性的结构。一个蛋白质可能拥有多个结构域，一个结构域也可能存在于多种蛋白质中。项目数据中，基于Pfam数据库及相应的PfamScan工具对鉴定到的蛋白质进行蛋白结构域注释。

**KEGG通路注释**

Kyoto Encyclopedia of Genes and
Genomes（KEGG）能够将当前已知的蛋白互作网络信息，比如通路及相关复合体（Pathway数据库）、基因及基因产物（Gene数据库）、生物内复合物及相关反应（Compound
and
Reaction数据库）等信息进行整合。KEGG的通路主要包括：代谢、遗传信息处理、环境信息处理、细胞过程、人类疾病、药物开发等。我们基于KEGG通路数据库对蛋白通路进行注释，将鉴定到的蛋白质进行BLAST比对（blastp，evalue
≤
1e-4），对于每一条序列的BLAST比对结果，选取比对得分（score）最高的比对结果进行注释。

**亚细胞定位**

真核生物组织细胞中的蛋白，依据与其结合的膜结构的差异，被详尽的定位到细胞内各种元件上。真核细胞主要的亚细胞定位包括：胞外、细胞质、细胞核、线粒体、高尔基体、内质网、过氧化物酶体、液泡、细胞骨架、核质、核基质以及核糖体等。基于此，我们使用预测亚细胞定位的WolF
Psort软件对所提交的蛋白进行亚细胞定位注释。相对于真核细胞，原核细胞一般没有细胞内膜，没有核膜包裹的成型细胞核，细胞内无染色体，DNA链未螺旋化，并以游离的形式存在于细胞质中，细胞质内也无任何有膜的细胞器（如线粒体或叶绿体）。此外，根据《伯杰氏细菌鉴定手册》，原核生物分为四大类：“有细胞壁的革兰氏阴性真细菌”、“有细胞壁的革兰氏阳性真细菌”、“无细胞壁的真细菌”、“古细菌”。基于此，我们使用PSORTb软件（v3.0）对以上四类原核生物中鉴定到蛋白质进行亚细胞结构预测分析。

**COG/KOG**

COG，即Clusters of Orthologous Groups of
proteins。构成每个COG的蛋白都是被假定为来自于一个祖先蛋白，Orthologs是指来自于不同物种、由垂直家系（物种形成）进化而来的蛋白，并且典型的保留与原始蛋白相同的功能。COG的中文释义即“同源蛋白簇”。COG分为两类，一类是原核生物，另一类是真核生物。原核生物的一般称为COG数据库；真核生物的一般称为KOG数据库。相较于其它数据库，比如NCBI的COG数据库，EggNOG提供了更全面的物种和更多蛋白质序列的同源分类，并对每个同源基因簇进行了系统发育树构建和功能注释。

### 5.2.2 蛋白质功能富集

**GO富集分析**

蛋白的GO注释被分为3个大类：生物过程、细胞组分、分子功能。使用费希尔精确检验（Fisher’s
exact
test）对差异表达蛋白进行GO富集显著性分析（以鉴定到的蛋白为背景），P
value < 0.05被认为是显著的。

**KEGG通路富集分析**

Kyoto Encyclopedia of Genes and
Genomes（KEGG）数据库用于差异表达蛋白的通路富集分析。使用费希尔精确检验（Fisher’s
exact
test）对差异表达蛋白进行通路富集显著性分析（以鉴定到的蛋白为背景），P
value < 0.05被认为是显著的。

**蛋白结构域富集分析**

Pfam数据库用于分析差异表达蛋白的功能结构域的富集情况。费希尔精确检验方法被用于检验差异表达蛋白在以鉴定蛋白为背景下的结构域富集显著性，P
value < 0.05被认为是显著的。

### 5.2.3 基于蛋白功能富集的聚类分析

基于不同分组的差异表达蛋白（或者不同差异倍数的差异表达蛋白）功能富集的聚类分析用于研究其在特定功能（GO、KEGG通路、蛋白结构域）上存在的潜在联系和差异。首先收集所用蛋白分组富集到的功能分类信息和对应的富集P
value值，然后筛选出至少在一个蛋白分组中为显著富集（P value <
0.05）的功能分类。筛选得到的P
value数据矩阵首先经过以-Log10为底的对数变换，然后将变换后的数据矩阵对各功能分类运用Z变换。最后将Z变换后得到的数据集使用层次聚类（欧式距离，平均连接聚类）方法做单边聚类分析。聚类关系使用R语言包pheatmap绘制出的热图进行可视化展示。

### 5.2.4 蛋白互作网络分析

将比较组中筛选得到的差异表达蛋白数据库编号或蛋白序列，通过与STRING（v.11.0）蛋白互作网络数据库比对后，按照confidence
score > 0.7（high confidence）提取得到差异表达蛋白互作关系。然后通过R
package “visNetwork”工具对差异表达蛋白互作网络进行可视化展示。

# 6 材料与方法 (英文版仅供参考)

## 6.1 Experimental Methods

**Protein Extraction**

**Cell samples:** Samples were taken from the -80°C
refrigerator and inserted on ice to thaw, then pre-mixed lysis solution
(8 M urea, 1% protease inhibitor) was slowly added to the sample tubes
and lysed by sonication in an ultrasonic crusher. Centrifugation (4°C,
15000 g, 10 min) was performed to remove cellular debris, and the
supernatant was transferred to a new centrifuge tube, and the
supernatant protein concentration was determined using the BCA
method.

**Animal tissues:** Samples were taken from the -80 °C
refrigerator, and an appropriate amount of tissue samples were
thoroughly ground into powder in a mortar pre-cooled with liquid
nitrogen, the powder was collected in a new centrifuge tube, and the
pre-mixed lysis solution (8 M urea, 1% protease inhibitor) was slowly
added to the sample tube, and the sample was lysed by ultrasonic lysis
in the ultrasonic crusher. Centrifugation (4°C, 15000 g, 10 min) was
performed to remove the cell residue, and the supernatant was
transferred to a new centrifuge tube, and the supernatant protein
concentration was determined using the BCA method.

**Serum Project Depletion High Abundance Method:**
Samples were taken from the -80°C refrigerator, thawed by insertion on
ice, centrifuged (4°C, 15000 g, 10 min), precipitate was removed, and
the supernatant was transferred to a new centrifuge tube and the
high-abundant protein was removed as described in the Pierce™ Top 14
Abundant Protein Depletion Spin Columns Kit from Thermo Scientific. The
protein concentration was determined using the BCA method.

**Supernatants (ultrafiltration):** Samples were taken
from the -80°C refrigerator, placed on ice, allowed to thaw completely,
and centrifuged (4°C, 15000 g, 10 min) to remove impurities. The
supernatant was transferred to an ultrafiltration centrifuge tube
(millipore), centrifuged to concentrate (4°C, 5000 g) the supernatant to
0.5 mL, and an equal volume of 8 M urea was added to replace it twice,
and finally the protein concentration was determined using the BCA
method.

**Enzyme Digestion**

**Precipitation enzyme digestion:** Based on the protein
concentration determination, equal amounts of protein were taken from
each sample and the volume was adjusted with lysis buffer to be
consistent. A suitable volume of TCA was slowly added to achieve a final
concentration of 20%, followed by thorough vortex mixing and incubation
at 4 ℃ for 2 hours for precipitation. After centrifugation (4 ℃, 15000
g, 5 min), the protein precipitates were collected and washed three
times with pre-cooled acetone at -20 ° C. After evaporating the acetone,
the precipitate was suspended in 100 mM TEAB, sonicated in a water bath,
and digested overnight at 37°C with trypsin at a ratio of 1: 50
(protease: protein, m/m). Reduction was performed by adding 5 mM
dithiothreitol (DTT) at 56°C for 30 min, followed by alkylation with 15
mM iodoacetamide (IAA) at room temperature in the dark for 15 min. After
completion of the reaction, the sample pH was adjusted to 2-3 with an
appropriate amount of 10% TFA, and desalting was carried out using
Strata X (Phenomenex) followed by vacuum freeze-drying. Peptide
quantification was performed using the Pierce™ Quantitative Peptide
Assays & Standards kit (Thermo Scientific) after complete
dissolution of peptides in water.

**FASP digestion:** Based on the protein concentration
determination, equal amounts of protein were taken from each sample and
the volume was adjusted with lysis buffer to achieve the same level.
Then, 5 mM DTT was added, followed by reduction at 56℃ for 30 min.
Subsequently, 15 mM IAA was added for reaction at room temperature in
the dark for 15 min. The reaction mixture was transferred to a
centrifugal filter unit (millipore), centrifuged (room temperature,
12000 g, 20 min), and washed three times with 8 M urea and then three
times with 25 mM HEPES. Trypsin digestion was performed overnight at
37°C at a ratio of 1:50 (protease: protein, w/w) for the first digestion
and for 4 hours at 37°C at a ratio of 1:100 (protease: protein, w/w) for
the second digestion. After centrifugation (room temperature, 12000 g,
20 min), the filter unit was washed with distilled water, and the
eluates from two centrifugation rounds were combined. Acidification was
performed by adding 10% TFA to adjust the pH to 2-3, followed by
desalting using Strata X (Phenomenex) and vacuum freeze-drying. Peptide
quantification was carried out using the Pierce™ Quantitative Peptide
Assays & Standards kit (Thermo Scientific) after complete
dissolution of peptides in water.

**Tandem Mass Tag (TMT)** After equal amounts of
peptides were taken from each sample, they were vacuum freeze-dried. The
peptides were then dissolved in 100mM HEPES, and peptide labeling was
conducted according to the instructions of the labeling kit. The simple
procedure is as follows: Before opening the labeling reagent, it was
equilibrated to room temperature. Then, it was fully dissolved in 100%
acetonitrile. After taking an appropriate amount of labeling reagent and
mixing it with the peptides, the mixture was incubated at room
temperature for 1 hour. After labeling, equal amounts of peptides were
mixed together. After desalting with a Tip, 1μg was taken for mass
spectrometry to check the labeling efficiency. Once the labeling
efficiency passed quality control, the labeled peptides were mixed with
an appropriate amount of 5% hydroxylamine and incubated for 15 minutes
to quench the reaction. After mixing, desalting was performed, followed
by vacuum freeze-drying for storage.

**HPLC Fractionation** The peptides were fractionated
using high-pH reversed-phase HPLC with an Agilent 300Extend C18 column
(3.5 μm particle size, 4.6 mm inner diameter, 250 mm length). Mobile
phase A is 2% acetonitrile solution (pH 9.0), mobile phase B is 98%
acetonitrile solution (pH 9.0). The column temperature was maintained at
40°C, and detection was carried out at a wavelength of 214 nm. The
gradient conditions were as follows: 0-5 min, 6% B; 5-8 min, 6-8% B;
8-54 min, 8-32% B; 54-60 min, 32-95% B; 60-65 min, 95% B; 65-70 min,
95-5% B; 70-75 min, 5-95% B; 75-80 min, 95% B; 80-83 min, 95-5% B; 83-90
min, 5% B. The flow rate was set at 1 ml/min. Fractions were collected
from 9 to 57 min, with 48 min required to separate 48 components.
Subsequently, the peptides were combined into 16 fractions, and the
combined fractions were vacuum freeze-dried for later use.

**LC-MS/MS Analysis**

**DDA:** The LC-MS/MS system comprises an Easy-nLC 1200
coupled with a Q Exactive HFX mass spectrometer. Mobile phase A was an
aqueous solution containing 0.1% formic acid and 2% acetonitrile; mobile
phase B was an aqueous solution containing 0.1% formic acid and 80%
acetonitrile. The length of the homemade analytical column was 20 cm,
and the packing material used was ReproSil-Pur C 18, 1.9 μm particles
from Dr. Maisch GmbH. 1 μg of peptide was dissolved in mobile phase A
and then separated using EASY-nLC 1200 ultra-high-performance liquid
chromatography (UHPLC) system. The liquid phase gradient was set: 0-26
min, 7%-22% B; 26-34 min, 22%-32% B; 34-37 min, 32%-80% B; 37-40 min,
80% B. The liquid phase flow rate was maintained at 450 nL/min.The
separated peptides were injected into the NanoFlex ion source for
atomization and then into the Q Exactive HF-X for mass spectrometry
analysis. The ion source voltage was set at 2.1 kV, the primary mass
spectrometry (MS) scanning range was set at 400-1200 with a resolution
of 60,000 (MS Resolution), and the secondary mass spectrometry (MS)
scanning range was set at 100 m/z with a resolution of 15,000 (MS2
Resolution). The data-dependent scanning (DDA) mode sets the TOP 20
precursor ions to enter the HCD collision cell sequentially for
fragmentation and then sequentially for secondary mass spectrometry
analysis. The automatic gain control (AGC) was set to 5E4, the signal
threshold was set to 1E4, and the maximum injection time was set to 22
ms. In order to avoid repeated scans of high-abundance peptides, the
dynamic exclusion time for tandem mass spectrometry was set to 30 s.

**Astral DIA:** The analysis utilizes the nanoflow rate
Vanquish Neo system (Thermo Fisher Scientific) for chromatographic
separation, and samples post high-efficiency liquid chromatography
separation are subjected to Data Independent Acquisition (DIA) mass
spectrometry using the Astral high-resolution mass spectrometer (Thermo
Scientific). The detection mode is positive ion, with a precursor ion
scan range of 380-980 m/z, and a primary mass spectrometry resolution of
240,000 at 200 m/z. Normalized AGC Target is set at 500%, with a Maximum
IT of 5ms. MS2 employs the DIA data acquisition mode, with 299 scan
windows set, an Isolation Window of 2m/z, HCD Collision Energy set at
25ev, Normalized AGC Target at 500%, and a Maximum IT of 3ms.

**Database Search**

**DDA:** Secondary mass spectrometry data were searched
using Maxquant (v1.6.15.0). The data type is TMT proteomics data based
on secondary reporter ion quantification. The secondary spectrum used
for quantification requires that the proportion of precursor ions in the
primary spectrum be greater than 75%. The database source is xxxx of the
Uniprot database, and common contamination libraries are added to the
database. Contaminating proteins were deleted during data analysis; the
digestion method was set to Trypsin/P; the maximum number of missed
cleavage sites was set to 2; First search and Main search were set to 20
ppm and 5 ppm for the precursor ion, respectively, and 20 ppm for the
secondary fragment ions. The fixed modification was set to be cysteine
alkylation, and the variable modifications were set to be oxidation of
methionine and acetylation of the protein N terminus. The FDR for
protein identification and PSM identification was set to be 1%.

**DIA:** The DIA data is processed using the DIA-NN
software. The software parameters are set as follows: the enzyme used is
trypsin, with a maximum missed cleavage site set to 1. Fixed
modification is Carbamidomethyl (C), and dynamic modifications are set
to Oxidation (M) and Acetyl (Protein N-term). Proteins identified
through database retrieval must pass the set filtering parameter of
FDR<1%.

## 6.2 Bioinformatic Analysis Methods

**Gene Ontology Annotation** Gene Ontology analysis (GO
analysis) is a bioinformatics analysis method that can link information
of biological/cellular processes to genes and gene products (such as
proteins) and provide statistical information. In proteomics projects,
GO is mainly used for the following purposes: 1) as a database of
information on proteins and genes; 2) provide a variety of information
on proteins and genes and classify proteins and genes according to the
information; 3) as a tool to provide the most comprehensive information
annotation and classification services for all proteins in the project.
GO analysis mainly includes three aspects: 1. Cellular Components (CC):
refers to specific components in cells, which should be the constituent
elements of larger components in the GO system. For example,
anatomically certain cellular structures (rough endoplasmic reticulum,
nucleus, etc.), or a series of gene products, such as the basic
structure of some complex components (ribosomes, protein dimers, etc.).
2. Molecular Function (MF): it mainly describes the chemical activity of
molecules, such as catalytic activity or binding activity that can be
manifested at the molecular level. 3. Biological Process (BP): a series
of molecules in the organism performs a specific function in an orderly
way, which is known as a biological process. GO annotation is the
annotation analysis of identified proteins using Gene Ontology database
(https://geneontology.org/). GO IDs of each protein were
extracted and classified into three main categories, including cellular
components, molecular functions, and biological processes.

**Protein Domain Annotation** The protein domains are
specific protein regions in proteins that are conserved in sequence and
can generally perform their functions independently, and are structural
elements of molecular function, generally consisting of 25 to 500 amino
acids. These regions are relatively spatially compact, structurally
stable, and capable of being independently folded into functional
structures. A protein may possess multiple domains, and a single domain
may be present in multiple proteins. In the project data, protein
domains were annotated for the identified proteins based on the Pfam
database.

**KEGG Pathway Annotation** The Kyoto Encyclopedia of
Genes and Genomes (KEGG) is capable of integrating information on
currently known protein interaction networks, such as pathways and
related complexes (Pathway database), genes and gene products (Gene
database), and biochemical complexes and related reactions (Compound and
Reaction database), etc. KEGG pathways mainly include: metabolism,
genetic information processing, environmental information processing,
cellular processes, human diseases, and drug development. We annotated
protein pathways based on the KEGG pathway database.

**Subcellular Localization** Proteins in eukaryotic
tissue cells are exhaustively localized to various intracellular
components based on differences in the structure of the membranes to
which they bind. The major subcellular localizations of eukaryotic cells
include: extracellular, cytoplasmic, nucleus, mitochondria, Golgi
apparatus, endoplasmic reticulum, peroxisomes, vesicles, cytoskeleton,
nucleoplasm, nuclear matrix, and ribosomes. In contrast to eukaryotic
cells, prokaryotic cells generally do not have an intracellular
membrane, do not have a molded nucleus encapsulated by a nuclear
membrane, have no intracellular chromosomes, have DNA strands that are
un-packed and are present in the cytoplasm as free circular strands, and
do not have any membranous organelles (e.g., mitochondria or
chloroplasts) in the cytoplasm. Based on the database, we performed
subcellular structure prediction analysis on the identified
proteins.

**COG/KOG** COG, Clusters of Orthologous Groups of
proteins, is a term used to describe proteins that are assumed to be
derived from a single ancestral protein; “Orthologs” are proteins that
are derived from a different species, evolved from a vertical lineage
(speciation), and typically retain the same function as the original
protein; the Chinese meaning of COG is “Clusters of Orthologous
Proteins”. COGs are categorized into two groups: prokaryotes and
eukaryotes. The prokaryotic ones are generally called COG databases,
while the eukaryotic ones are generally called KOG databases. Compared
with other databases, such as NCBI’s COG database, EggNOG provides a
more comprehensive homology classification of species and protein
sequences, as well as phylogenetic tree construction and functional
annotation for each homologous gene cluster.

**Protein Functional Enrichment** GO terms, KEGG pathway
and protein domain enrichment analysis of DEPs was performed using
Fisher’s exact test (with the identified proteins as background), and a
P value < 0.05 was considered significant.

**Cluster analysis based on protein functional
enrichment** To examine potential links and differences in
certain fun ctions (GO, KEGG pathway, protein structural domains) across
several comparison groups, cluster analysis is done based on functional
enrichment results of DEPs from different comparison groups. Based on
the functional enrichment results of the comparison groups,
significantly enriched terms were filtered using P value < 0.05 in at
least one comparison groups. Prior to being Z-transformed for each
functional classification, the filtered P value data matrix underwent a
log transformation with -Log10 as the base. Finally, one-sided cluster
analysis utilising hierarchical clustering (euclidean distance, average
linkage clustering) was performed on the dataset acquired after
Z-transformation. Heatmaps were plotted using the R package
“pheatmap”.

**Protein-Protein Interaction** The differentially
expressed protein database accession or sequence screened in comparison
groups were matched with the STRING (v.11.0) protein interaction network
database to get the protein-protein interactions with high confidence
using confidence score > 0.7 as threshold. Next, we used the R
package “visNetwork” to visualize the differential expressed
protein-protein interaction network.
