# Supplementary material for: Multi-Omics and Experimental Validation Reveal the Protective Effect of Paeoniflorin Against Coronary Heart Disease in Mice via Inhibiting the C3-Cfd-C3aR Pathway
Source: Int J Mol Sci. 2026 Jul 13;27(14):6236. doi: 10.3390/ijms27146236 (PMC13410309; doi:10.3390/ijms27146236)
Supplement: Supplementary file 1 [file ijms-27-06236-s001.zip › Supplementary Materials/ijms-4276706_Metabolomics_Dataset/4-Functional Annotation and Enrichment/Figure 4e. MSEA enrichment2 of Model-vs-Paeoniflorin.pdf]

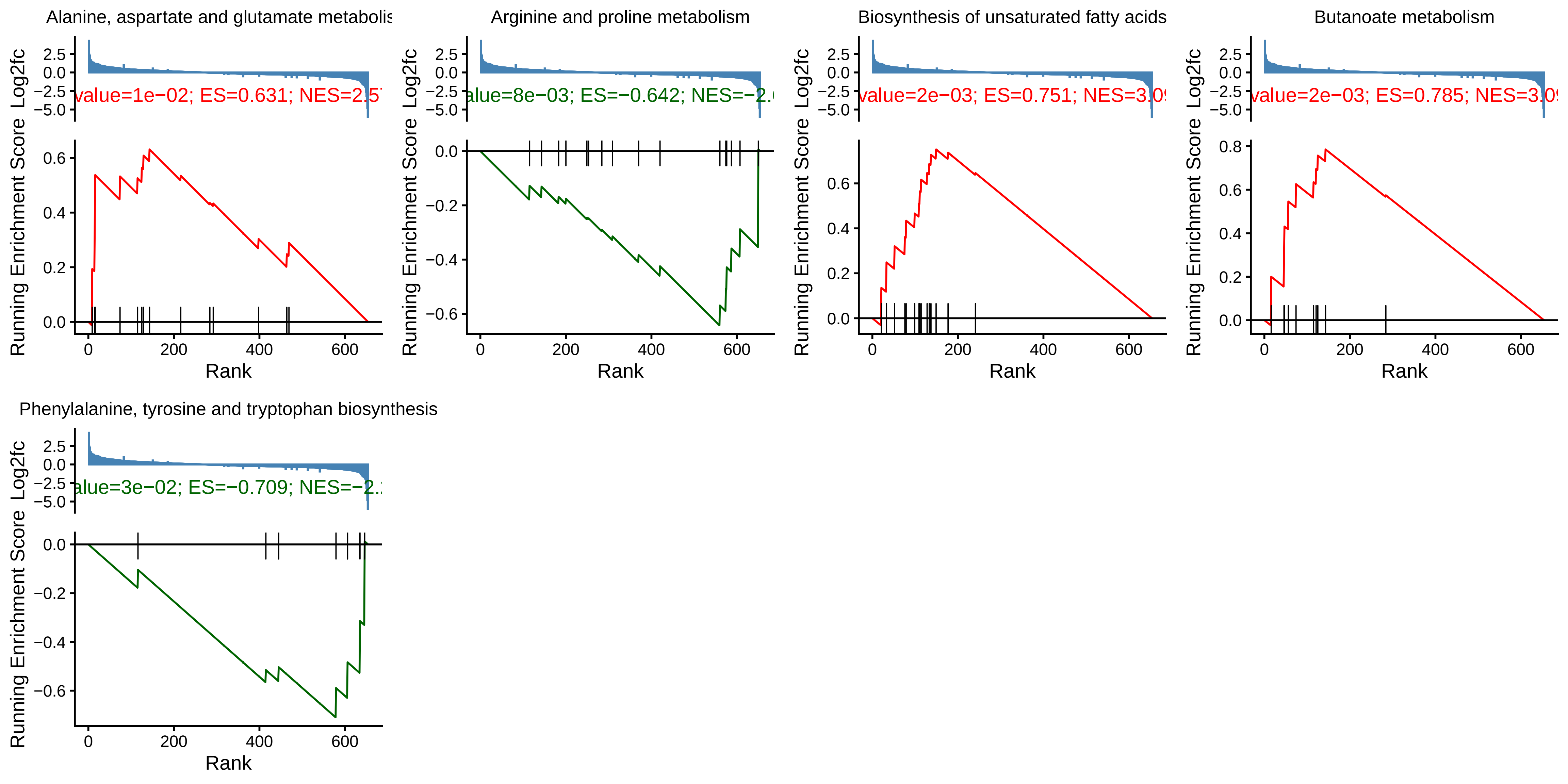
Alanine, aspartate and glutamate metabolis
value=1e−02; ES=0.631; NES=2.5
Arginine and proline metabolism
alue=8e−03; ES=−0.642; NES=−2.
Biosynthesis of unsaturated fatty acids
value=2e−03; ES=0.751; NES=3.0
Butanoate metabolism
value=2e−03; ES=0.785; NES=3.0
Phenylalanine, tyrosine and tryptophan biosynthesis
alue=3e−02; ES=−0.709; NES=−2.
Log2fc
Running Enrichment Score
Rank
