# Supplementary material for: Multi-Omics and Experimental Validation Reveal the Protective Effect of Paeoniflorin Against Coronary Heart Disease in Mice via Inhibiting the C3-Cfd-C3aR Pathway
Source: Int J Mol Sci. 2026 Jul 13;27(14):6236. doi: 10.3390/ijms27146236 (PMC13410309; doi:10.3390/ijms27146236)
Supplement: Supplementary file 1 [file ijms-27-06236-s001.zip › Supplementary Materials/ijms-4276706_Metabolomics_Dataset/4-Functional Annotation and Enrichment/Figure 4d. MSEA enrichment1 of Control-vs-Model.pdf]

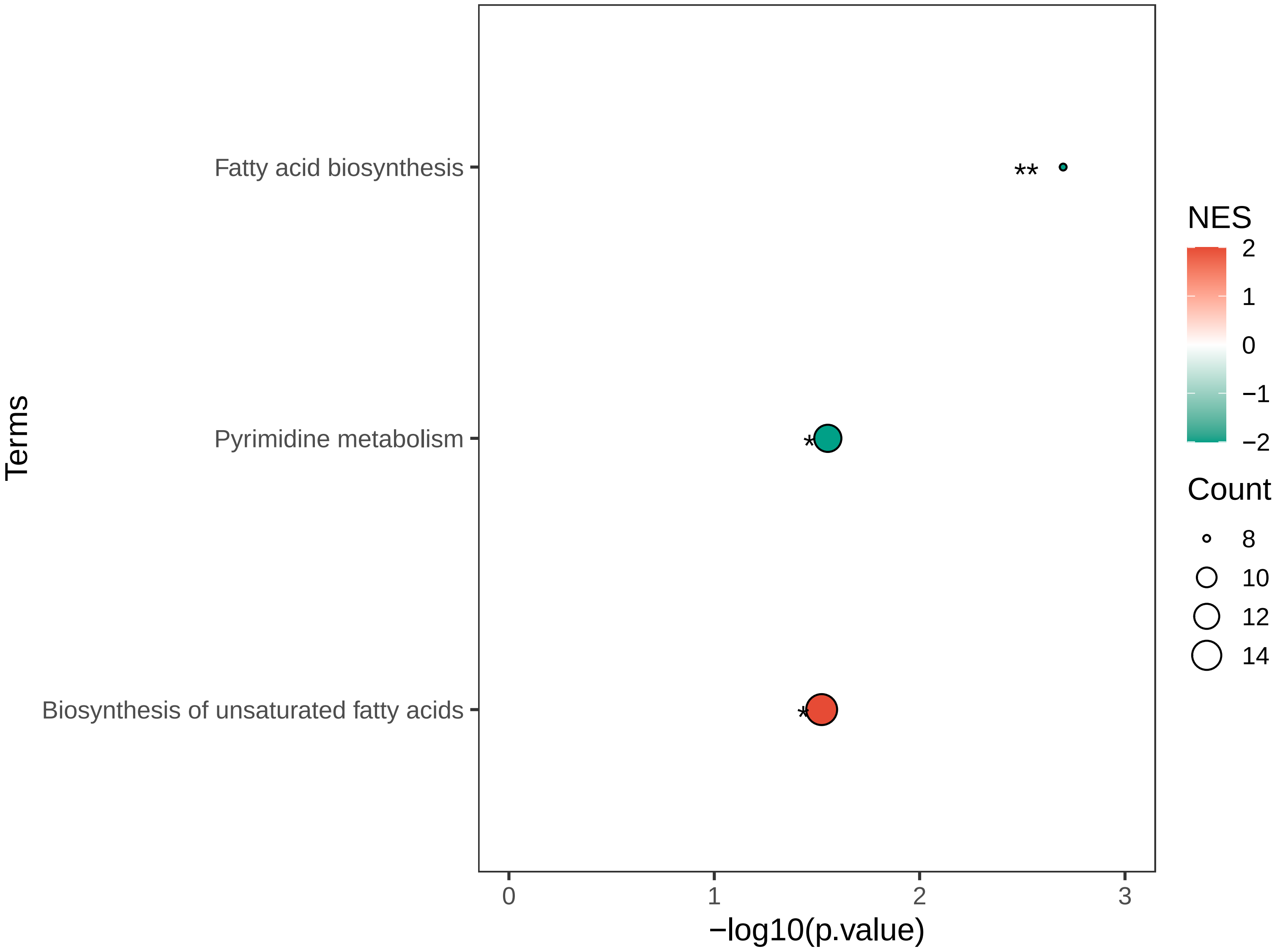

Fatty acid biosynthesis
Pyrimidine metabolism
Biosynthesis of unsaturated fatty acids
Terms
**
*
*
0
1
2
3
−log10(p.value)
NES
2
1
0
−1
−2
Count
8
10
12
14
