# Supplementary material for: Multi-Omics and Experimental Validation Reveal the Protective Effect of Paeoniflorin Against Coronary Heart Disease in Mice via Inhibiting the C3-Cfd-C3aR Pathway
Source: Int J Mol Sci. 2026 Jul 13;27(14):6236. doi: 10.3390/ijms27146236 (PMC13410309; doi:10.3390/ijms27146236)
Supplement: Supplementary file 1 [file ijms-27-06236-s001.zip › Supplementary Materials/ijms-4276706_Proteomics_Dataset/8-KEGG_pathway_image/Model-vs-Paeoniflorin/mmu04370.html]

KEGG PATHWAY: VEGF signaling pathway - Mus musculus (house mouse)


# VEGF signaling pathway - Mus musculus (house mouse)


[
Pathway menu
| Organism menu
| Pathway entry
| Show description
| Download
| Help
]

There is now much evidence that VEGFR-2 is the major mediator of VEGF-driven responses in endothelial cells and it is considered to be a crucial signal transducer in both physiologic and pathologic angiogenesis. The binding of VEGF to VEGFR-2 leads to a cascade of different signaling pathways, resulting in the up-regulation of genes involved in mediating the proliferation and migration of endothelial cells and promoting their survival and vascular permeability. For example, the binding of VEGF to VEGFR-2 leads to dimerization of the receptor, followed by intracellular activation of the PLCgamma;PKC-Raf kinase-MEK-mitogen-activated protein kinase (MAPK) pathway and subsequent initiation of DNA synthesis and cell growth, whereas activation of the phosphatidylinositol 3' -kinase (PI3K)-Akt pathway leads to increased endothelial-cell survival. Activation of PI3K, FAK, and p38 MAPK is implicated in cell migration signaling.


##### Option

Scale:


100%

Image resolution:


 High

##### Background color

Organism

##### Search

##### ID search

##### Color


KGML

Image (png) file 1x

Image (png) file 2x
