# Supplementary material for: Multi-Omics and Experimental Validation Reveal the Protective Effect of Paeoniflorin Against Coronary Heart Disease in Mice via Inhibiting the C3-Cfd-C3aR Pathway
Source: Int J Mol Sci. 2026 Jul 13;27(14):6236. doi: 10.3390/ijms27146236 (PMC13410309; doi:10.3390/ijms27146236)
Supplement: Supplementary file 1 [file ijms-27-06236-s001.zip › Supplementary Materials/ijms-4276706_Proteomics_Dataset/8-KEGG_pathway_image/Model-vs-Paeoniflorin/mmu04720.html]

KEGG PATHWAY: Long-term potentiation - Mus musculus (house mouse)


# Long-term potentiation - Mus musculus (house mouse)


[
Pathway menu
| Organism menu
| Pathway entry
| Show description
| Download
| Help
]

Hippocampal long-term potentiation (LTP), a long-lasting increase in synaptic efficacy, is the molecular basis for learning and memory. Tetanic stimulation of afferents in the CA1 region of the hippocampus induces glutamate release and activation of glutamate receptors in dendritic spines. A large increase in [Ca2+]i resulting from influx through NMDA receptors leads to constitutive activation of CaM kinase II (CaM KII) . Constitutively active CaM kinase II phosphorylates AMPA receptors, resulting in potentiation of the ionic conductance of AMPA receptors. Early-phase LTP (E-LTP) expression is due, in part, to this phosphorylation of the AMPA receptor. It is hypothesized that postsynaptic Ca2+ increases generated through NMDA receptors activate several signal transduction pathways including the Erk/MAP kinase and cAMP regulatory pathways. The convergence of these pathways at the level of the CREB/CRE transcriptional pathway may increase expression of a family of genes required for late-phase LTP (L-LTP).


##### Option

Scale:


100%

Image resolution:


 High

##### Background color

Organism

##### Search

##### ID search

##### Color


KGML

Image (png) file 1x

Image (png) file 2x
