# Supplementary material for: Multi-Omics and Experimental Validation Reveal the Protective Effect of Paeoniflorin Against Coronary Heart Disease in Mice via Inhibiting the C3-Cfd-C3aR Pathway
Source: Int J Mol Sci. 2026 Jul 13;27(14):6236. doi: 10.3390/ijms27146236 (PMC13410309; doi:10.3390/ijms27146236)
Supplement: Supplementary file 1 [file ijms-27-06236-s001.zip › Supplementary Materials/ijms-4276706_Proteomics_Dataset/8-KEGG_pathway_image/Model-vs-Paeoniflorin/mmu04921.html]

KEGG PATHWAY: Oxytocin signaling pathway - Mus musculus (house mouse)


# Oxytocin signaling pathway - Mus musculus (house mouse)


[
Pathway menu
| Organism menu
| Pathway entry
| Show description
| Download
| Help
]

Oxytocin (OT) is a nonapeptide synthesized by the magno-cellular neurons located in the supraoptic (SON) and paraventricular (PVN) nuclei of the hypothalamus. It exerts a wide variety of central and peripheral effects. However, its best-known and most well-established roles are stimulation of uterine contractions during parturition and milk release during lactation. Oxytocin also influences cardiovascular regulation and various social behaviors. The actions of OT are all mediated by one type of OT receptor (OTR). This is a transmembrane receptor belonging to the G-protein-coupled receptor superfamily. The main signaling pathway is the Gq/PLC/Ins3 pathway, but the MAPK and the RhoA/Rho kinase pathways are also activated, contributing to increased prostaglandin production and direct contractile effect on myometrial cells. In the cardiovascular system, OTR is associated with the ANP-cGMP and NO-cGMP pathways, which reduce the force and rate of contraction and increase vasodilatation.


##### Option

Scale:


100%

Image resolution:


 High

##### Background color

Organism

##### Search

##### ID search

##### Color


KGML

Image (png) file 1x

Image (png) file 2x
