# Supplementary material for: Multi-Omics and Experimental Validation Reveal the Protective Effect of Paeoniflorin Against Coronary Heart Disease in Mice via Inhibiting the C3-Cfd-C3aR Pathway
Source: Int J Mol Sci. 2026 Jul 13;27(14):6236. doi: 10.3390/ijms27146236 (PMC13410309; doi:10.3390/ijms27146236)
Supplement: Supplementary file 1 [file ijms-27-06236-s001.zip › Supplementary Materials/ijms-4276706_Proteomics_Dataset/8-KEGG_pathway_image/Model-vs-Paeoniflorin/mmu05330.html]

KEGG PATHWAY: Allograft rejection - Mus musculus (house mouse)


# Allograft rejection - Mus musculus (house mouse)


[
Pathway menu
| Organism menu
| Pathway entry
| Show description
| Download
| Help
]

Allograft rejection is the consequence of the recipient's alloimmune response to nonself antigens expressed by donor tissues. After transplantation of organ allografts, there are two pathways of antigen presentation. In the direct pathway, recipient T cells react to intact allogeneic MHC molecules expressed on the surface of donor cells. This pathway would activate host CD4 or CD8 T cells. In contrast, donor MHC molecules (and all other proteins) shed from the graft can be taken up by host APCs and presented to recipient T cells in the context of self-MHC molecules - the indirect pathway. Such presentation activates predominantly CD4 T cells. A direct cytotoxic T-cell attack on graft cells can be made only by T cells that recognize the graft MHC molecules directly. Nontheless, T cells with indirect allospecificity can contribute to graft rejection by activating macrophages, which cause tissue injury and fibrosis, and are also likely to be important in the development of an alloantibody response to graft.


##### Option

Scale:


100%

Image resolution:


 High

##### Background color

Organism

##### Search

##### ID search

##### Color


KGML

Image (png) file 1x

Image (png) file 2x
