# Supplementary material for: Multi-Omics and Experimental Validation Reveal the Protective Effect of Paeoniflorin Against Coronary Heart Disease in Mice via Inhibiting the C3-Cfd-C3aR Pathway
Source: Int J Mol Sci. 2026 Jul 13;27(14):6236. doi: 10.3390/ijms27146236 (PMC13410309; doi:10.3390/ijms27146236)
Supplement: Supplementary file 1 [file ijms-27-06236-s001.zip › Supplementary Materials/ijms-4276706_Proteomics_Dataset/8-KEGG_pathway_image/Model-vs-Paeoniflorin/mmu04940.html]

KEGG PATHWAY: Type I diabetes mellitus - Mus musculus (house mouse)


# Type I diabetes mellitus - Mus musculus (house mouse)


[
Pathway menu
| Organism menu
| Pathway entry
| Show description
| Download
| Help
]

Type I diabetes mellitus is a disease that results from autoimmune destruction of the insulin-producing beta-cells. Certain beta-cell proteins act as autoantigens after being processed by antigen-presenting cell (APC), such as macrophages and dendritic cells, and presented in a complex with MHC-II molecules on the surface of the APC. Then immunogenic signals from APC activate CD4+ T cells, predominantly of the Th1 subset. Antigen-activated Th1 cells produce IL-2 and IFNgamma. They activate macrophages and cytotoxic CD8+ T cells, and these effector cells may kill islet beta-cells by one or both of two types of mechanisms: (1) direct interactions of antigen-specific cytotoxic T cells with a beta-cell autoantigen-MHC-I complex on the beta-cell, and (2) non-specific inflammatory mediators, such as free radicals/oxidants and cytokines (IL-1, TNFalpha, TNFbeta, IFNgamma).
Type I diabetes is a polygenic disease. One of the principle determining genetic factors in diabetes incidence is the inheritance of mutant MHC-II alleles. Another plausible candidate gene is the insulin gene.


##### Option

Scale:


100%

Image resolution:


 High

##### Background color

Organism

##### Search

##### ID search

##### Color


KGML

Image (png) file 1x

Image (png) file 2x
