# Supplementary material for: Multi-Omics and Experimental Validation Reveal the Protective Effect of Paeoniflorin Against Coronary Heart Disease in Mice via Inhibiting the C3-Cfd-C3aR Pathway
Source: Int J Mol Sci. 2026 Jul 13;27(14):6236. doi: 10.3390/ijms27146236 (PMC13410309; doi:10.3390/ijms27146236)
Supplement: Supplementary file 1 [file ijms-27-06236-s001.zip › Supplementary Materials/ijms-4276706_Proteomics_Dataset/8-KEGG_pathway_image/Model-vs-Paeoniflorin/mmu04014.html]

KEGG PATHWAY: Ras signaling pathway - Mus musculus (house mouse)


# Ras signaling pathway - Mus musculus (house mouse)


[
Pathway menu
| Organism menu
| Pathway entry
| Show description
| Download
| Help
]

The Ras proteins are GTPases that function as molecular switches for signaling pathways regulating cell proliferation, survival, growth, migration, differentiation or cytoskeletal dynamism. Ras proteins transduce signals from extracellular growth factors by cycling between inactive GDP-bound and active GTP-bound states. The exchange of GTP for GDP on RAS is regulated by guanine nucleotide exchange factors (GEFs) and GTPase-activating proteins (GAPs). Activated RAS (RAS-GTP) regulates multiple cellular functions through effectors including Raf, phosphatidylinositol 3-kinase (PI3K) and Ral guanine nucleotide-dissociation stimulator (RALGDS).


##### Option

Scale:


100%

Image resolution:


 High

##### Background color

Organism

##### Search

##### ID search

##### Color


KGML

Image (png) file 1x

Image (png) file 2x
