# Supplementary material for: Multi-Omics and Experimental Validation Reveal the Protective Effect of Paeoniflorin Against Coronary Heart Disease in Mice via Inhibiting the C3-Cfd-C3aR Pathway
Source: Int J Mol Sci. 2026 Jul 13;27(14):6236. doi: 10.3390/ijms27146236 (PMC13410309; doi:10.3390/ijms27146236)
Supplement: Supplementary file 1 [file ijms-27-06236-s001.zip › Supplementary Materials/ijms-4276706_Proteomics_Dataset/8-KEGG_pathway_image/Model-vs-Paeoniflorin/mmu05150.html]

KEGG PATHWAY: Staphylococcus aureus infection - Mus musculus (house mouse)


# Staphylococcus aureus infection - Mus musculus (house mouse)


[
Pathway menu
| Organism menu
| Pathway entry
| Show description
| Download
| Help
]

Staphylococcus aureus can cause multiple forms of infections ranging from superficial skin infections to food poisoning and life-threatening infections. The organism has several ways to divert the effectiveness of the immune system: secreting immune modulating proteins that inhibit complement activation and neutrophil chemotaxis or lysis, modulating the sensitivity to cationic antimicrobial peptides (such as defensin) by increasing the positive net charge of its cytoplasmic membrane, and expression of superantigens that prevent development of a normal immune response or cause an emetic response when ingested.


##### Option

Scale:


100%

Image resolution:


 High

##### Background color

Organism

##### Search

##### ID search

##### Color


KGML

Image (png) file 1x

Image (png) file 2x
