# Supplementary material for: Multi-Omics and Experimental Validation Reveal the Protective Effect of Paeoniflorin Against Coronary Heart Disease in Mice via Inhibiting the C3-Cfd-C3aR Pathway
Source: Int J Mol Sci. 2026 Jul 13;27(14):6236. doi: 10.3390/ijms27146236 (PMC13410309; doi:10.3390/ijms27146236)
Supplement: Supplementary file 1 [file ijms-27-06236-s001.zip › Supplementary Materials/ijms-4276706_Proteomics_Dataset/8-KEGG_pathway_image/Model-vs-Paeoniflorin/mmu04613.html]

KEGG PATHWAY: Neutrophil extracellular trap formation - Mus musculus (house mouse)


# Neutrophil extracellular trap formation - Mus musculus (house mouse)


[
Pathway menu
| Organism menu
| Pathway entry
| Show description
| Download
| Help
]

Neutrophils play a central role in innate immune defense. One of the mechanisms of neutrophil action is the formation of neutrophil extracellular traps (NETs), the extracellular structures composed of chromatin coated with histones, proteases and granular and cytosolic proteins that help catch and kill microorganisms. NETs are formed by a process known as "NETosis" that can be triggered by microorganisms and endogenous stimuli, such as damage-associated molecular patterns and immune complexes, and involves activation in most cases of nicotinamide adenine dinucleotide phosphate (NADPH)-oxidase, which produces reactive oxygen species (ROS). Recent study has reported that there are two different mechanisms of NETosis, including a lytic NETosis and a vital NETosis. Lytic NETosis begins with nuclear delobulation and the disassembly of the nuclear envelope and continues with loss of cellular polarization, chromatin decondensation and plasma membrane rupture. Vital NETosis can occur independently of cell death and involves the secreted expulsion of nuclear chromatin that is accompanied by the release of granule proteins through degranulation.


##### Option

Scale:


100%

Image resolution:


 High

##### Background color

Organism

##### Search

##### ID search

##### Color


KGML

Image (png) file 1x

Image (png) file 2x
