# Supplementary material for: Multi-Omics and Experimental Validation Reveal the Protective Effect of Paeoniflorin Against Coronary Heart Disease in Mice via Inhibiting the C3-Cfd-C3aR Pathway
Source: Int J Mol Sci. 2026 Jul 13;27(14):6236. doi: 10.3390/ijms27146236 (PMC13410309; doi:10.3390/ijms27146236)
Supplement: Supplementary file 1 [file ijms-27-06236-s001.zip › Supplementary Materials/ijms-4276706_Proteomics_Dataset/8-KEGG_pathway_image/Model-vs-Paeoniflorin/mmu04015.html]

KEGG PATHWAY: Rap1 signaling pathway - Mus musculus (house mouse)


# Rap1 signaling pathway - Mus musculus (house mouse)


[
Pathway menu
| Organism menu
| Pathway entry
| Show description
| Download
| Help
]

Rap1 is a small GTPase that controls diverse processes, such as cell adhesion, cell-cell junction formation and cell polarity. Like all G proteins, Rap1 cycles between an inactive GDP-bound and an active GTP-bound conformation. A variety of extracellular signals control this cycle through the regulation of several unique guanine nucleotide exchange factors (GEFs) and GTPase activating proteins (GAPs). Rap1 plays a dominant role in the control of cell-cell and cell-matrix interactions by regulating the function of integrins and other adhesion molecules in various cell types. Rap1 also regulates MAP kinase (MAPK) activity in a manner highly dependent on the context of cell types.


##### Option

Scale:


100%

Image resolution:


 High

##### Background color

Organism

##### Search

##### ID search

##### Color


KGML

Image (png) file 1x

Image (png) file 2x
