# Supplementary material for: Multi-Omics and Experimental Validation Reveal the Protective Effect of Paeoniflorin Against Coronary Heart Disease in Mice via Inhibiting the C3-Cfd-C3aR Pathway
Source: Int J Mol Sci. 2026 Jul 13;27(14):6236. doi: 10.3390/ijms27146236 (PMC13410309; doi:10.3390/ijms27146236)
Supplement: Supplementary file 1 [file ijms-27-06236-s001.zip › Supplementary Materials/ijms-4276706_Proteomics_Dataset/8-KEGG_pathway_image/Model-vs-Paeoniflorin/mmu05203.html]

KEGG PATHWAY: Viral carcinogenesis - Mus musculus (house mouse)


# Viral carcinogenesis - Mus musculus (house mouse)


[
Pathway menu
| Organism menu
| Pathway entry
| Show description
| Download
| Help
]

There is a strong association between viruses and the development of human malignancies. We now know that at least six human viruses, Epstein-Barr virus (EBV), hepatitis B virus (HBV), hepatitis C virus (HCV), human papilloma virus (HPV), human T-cell lymphotropic virus (HTLV-1) and Kaposi's associated sarcoma virus (KSHV) contribute to 10-15% of the cancers worldwide. Via expression of many potent oncoproteins, these tumor viruses promote an aberrant cell-proliferation via modulating cellular cell-signaling pathways and escape from cellular defense system such as blocking apoptosis. Human tumor virus oncoproteins can also disrupt pathways that are necessary for the maintenance of the integrity of host cellular genome. Viruses that encode such activities can contribute to initiation as well as progression of human cancers.


##### Option

Scale:


100%

Image resolution:


 High

##### Background color

Organism

##### Search

##### ID search

##### Color


KGML

Image (png) file 1x

Image (png) file 2x
