# Supplementary material for: Multi-Omics and Experimental Validation Reveal the Protective Effect of Paeoniflorin Against Coronary Heart Disease in Mice via Inhibiting the C3-Cfd-C3aR Pathway
Source: Int J Mol Sci. 2026 Jul 13;27(14):6236. doi: 10.3390/ijms27146236 (PMC13410309; doi:10.3390/ijms27146236)
Supplement: Supplementary file 1 [file ijms-27-06236-s001.zip › Supplementary Materials/ijms-4276706_Proteomics_Dataset/8-KEGG_pathway_image/Model-vs-Paeoniflorin/mmu04520.html]

KEGG PATHWAY: Adherens junction - Mus musculus (house mouse)


# Adherens junction - Mus musculus (house mouse)


[
Pathway menu
| Organism menu
| Pathway entry
| Show description
| Download
| Help
]

Cell-cell adherens junctions (AJs), the most common type of intercellular adhesions, are important for maintaining tissue architecture and cell polarity and can limit cell movement and proliferation. At AJs, E-cadherin serves as an essential cell adhesion molecules (CAMs). The cytoplasmic tail binds beta-catenin, which in turn binds alpha-catenin. Alpha-catenin is associated with F-actin bundles directly and indirectly. The integrity of the cadherin-catenin complex is negatively regulated by phosphorylation of beta-catenin by receptor tyrosine kinases (RTKs) and cytoplasmic tyrosine kinases (Fer, Fyn, Yes, and Src), which leads to dissociation of the cadherin-catenin complex. Integrity of this complex is positively regulated by beta -catenin phosphorylation by casein kinase II, and dephosphorylation by protein tyrosine phosphatases. Changes in the phosphorylation state of beta-catenin affect cell-cell adhesion, cell migration and the level of signaling beta-catenin. Wnt signaling acts as a positive regulator of beta-catenin by inhibiting beta-catenin degradation, which stabilizes beta-catenin, and causes its accumulation. Cadherin may acts as a negative regulator of signaling beta-catenin as it binds beta-catenin at the cell surface and thereby sequesters it from the nucleus. Nectins also function as CAMs at AJs, but are more highly concentrated at AJs than E-cadherin. Nectins transduce signals through Cdc42 and Rac, which reorganize the actin cytoskeleton, regulate the formation of AJs, and strengthen cell-cell adhesion.


##### Option

Scale:


100%

Image resolution:


 High

##### Background color

Organism

##### Search

##### ID search

##### Color


KGML

Image (png) file 1x

Image (png) file 2x
