# Supplementary material for: Multi-Omics and Experimental Validation Reveal the Protective Effect of Paeoniflorin Against Coronary Heart Disease in Mice via Inhibiting the C3-Cfd-C3aR Pathway
Source: Int J Mol Sci. 2026 Jul 13;27(14):6236. doi: 10.3390/ijms27146236 (PMC13410309; doi:10.3390/ijms27146236)
Supplement: Supplementary file 1 [file ijms-27-06236-s001.zip › Supplementary Materials/ijms-4276706_Proteomics_Dataset/8-KEGG_pathway_image/Model-vs-Paeoniflorin/mmu04062.html]

KEGG PATHWAY: Chemokine signaling pathway - Mus musculus (house mouse)


# Chemokine signaling pathway - Mus musculus (house mouse)


[
Pathway menu
| Organism menu
| Pathway entry
| Show description
| Download
| Help
]

Inflammatory immune response requires the recruitment of leukocytes to the site of inflammation upon foreign insult. Chemokines are small chemoattractant peptides that provide directional cues for the cell trafficking and thus are vital for protective host response. In addition, chemokines regulate plethora of biological processes of hematopoietic cells to lead cellular activation, differentiation and survival.
The chemokine signal is transduced by chemokine receptors (G-protein coupled receptors) expressed on the immune cells. After receptor activation, the alpha- and beta-gamma-subunits of G protein dissociate to activate diverse downstream pathways resulting in cellular polarization and actin reorganization. Various members of small GTPases are involved in this process. Induction of nitric oxide and production of reactive oxygen species are as well regulated by chemokine signal via calcium mobilization and diacylglycerol production.


##### Option

Scale:


100%

Image resolution:


 High

##### Background color

Organism

##### Search

##### ID search

##### Color


KGML

Image (png) file 1x

Image (png) file 2x
