# Supplementary material for: Multi-Omics and Experimental Validation Reveal the Protective Effect of Paeoniflorin Against Coronary Heart Disease in Mice via Inhibiting the C3-Cfd-C3aR Pathway
Source: Int J Mol Sci. 2026 Jul 13;27(14):6236. doi: 10.3390/ijms27146236 (PMC13410309; doi:10.3390/ijms27146236)
Supplement: Supplementary file 1 [file ijms-27-06236-s001.zip › Supplementary Materials/ijms-4276706_Proteomics_Dataset/8-KEGG_pathway_image/Model-vs-Paeoniflorin/mmu04664.html]

KEGG PATHWAY: Fc epsilon RI signaling pathway - Mus musculus (house mouse)


# Fc epsilon RI signaling pathway - Mus musculus (house mouse)


[
Pathway menu
| Organism menu
| Pathway entry
| Show description
| Download
| Help
]

Fc epsilon RI-mediated signaling pathways in mast cells are initiated by the interaction of antigen (Ag) with IgE bound to the extracellular domain of the alpha chain of Fc epsilon RI. The activation pathways are regulated both positively and negatively by the interactions of numerous signaling molecules. Mast cells that are thus activated release preformed granules which contain biogenic amines (especially histamines) and proteoglycans (especially heparin). The activation of phospholipase A2 causes the release of membrane lipids followed by development of lipid mediators such as leukotrienes (LTC4, LTD4 and LTE4) and prostaglandins (especially PDG2). There is also secretion of cytokines, the most important of which are TNF-alpha, IL-4 and IL-5. These mediators and cytokines contribute to inflammatory responses.


##### Option

Scale:


100%

Image resolution:


 High

##### Background color

Organism

##### Search

##### ID search

##### Color


KGML

Image (png) file 1x

Image (png) file 2x
