# Supplementary material for: Multi-Omics and Experimental Validation Reveal the Protective Effect of Paeoniflorin Against Coronary Heart Disease in Mice via Inhibiting the C3-Cfd-C3aR Pathway
Source: Int J Mol Sci. 2026 Jul 13;27(14):6236. doi: 10.3390/ijms27146236 (PMC13410309; doi:10.3390/ijms27146236)
Supplement: Supplementary file 1 [file ijms-27-06236-s001.zip › Supplementary Materials/ijms-4276706_Proteomics_Dataset/8-KEGG_pathway_image/Model-vs-Paeoniflorin/mmu05167.html]

KEGG PATHWAY: Kaposi sarcoma-associated herpesvirus infection - Mus musculus (house mouse)


# Kaposi sarcoma-associated herpesvirus infection - Mus musculus (house mouse)


[
Pathway menu
| Organism menu
| Pathway entry
| Show description
| Download
| Help
]

Kaposi sarcoma-associated herpesvirus (KSHV), also known as human herpesvirus 8 (HHV-8), is the most recently identified human tumor virus, and is associated with the pathogenesis of Kaposi's sarcoma (KS), primary effusion lymphoma (PEL), and Multicentric Castleman's disease (MCD). Like all other herpesviruses, KSHV displays two modes of life cycle, latency and lytic replication, which are characterized by the patterns of viral gene expression. Genes expressed in latency (LANA, v-cyclin, v-FLIP, Kaposins A, B and C and viral miRNAs) are mainly thought to facilitate the establishment of life long latency in its host and survival against the host innate, and adaptive immune surveillance mechanisms. Among the viral proteins shown to be expressed during lytic replication are potent signaling molecules such as vGPCR, vIL6, vIRFs, vCCLs, K1 and K15, which have been implicated experimentally in the angiogenic and inflammatory phenotype observed in KS lesions. Several of these latent viral and lytic proteins are known to transform host cells, linking KSHV with the development of severe human malignancies.


##### Option

Scale:


100%

Image resolution:


 High

##### Background color

Organism

##### Search

##### ID search

##### Color


KGML

Image (png) file 1x

Image (png) file 2x
