# Supplementary material for: Multi-Omics and Experimental Validation Reveal the Protective Effect of Paeoniflorin Against Coronary Heart Disease in Mice via Inhibiting the C3-Cfd-C3aR Pathway
Source: Int J Mol Sci. 2026 Jul 13;27(14):6236. doi: 10.3390/ijms27146236 (PMC13410309; doi:10.3390/ijms27146236)
Supplement: Supplementary file 1 [file ijms-27-06236-s001.zip › Supplementary Materials/ijms-4276706_Proteomics_Dataset/8-KEGG_pathway_image/Model-vs-Paeoniflorin/mmu05171.html]

KEGG PATHWAY: Coronavirus disease - COVID-19 - Mus musculus (house mouse)


# Coronavirus disease - COVID-19 - Mus musculus (house mouse)


[
Pathway menu
| Organism menu
| Pathway entry
| Show description
| Download
| Help
]

Coronavirus disease of 2019 (COVID-19) is a highly contagious respiratory infection that is caused by the severe acute respiratory syndrome coronavirus 2 (SARS-CoV-2). SARS-CoV-2 infects alveolar epithelial cells [mainly alveolar epithelial type 2 (AEC2) cells] through the angiotensin-converting enzyme 2 (ACE2) receptor. Upon the occupancy of ACE2 by SARS-CoV-2, the increased serum level of free Angiotensin II (Ang II) due to a reduction of ACE2-mediated degradation promotes activation of the NF-kappa B pathway via Ang II type 1 receptor (AT1R), followed by interleukin-6 (IL-6) production. SARS-CoV-2 also activates the innate immune system; macrophage stimulation triggers the overproduction of pro-inflammatory cytokines, including IL-6, and the "cytokine storm", which results in systemic inflammatory response syndrome and multiple organ failure. The combined effects of complement activation, dysregulated neutrophilia, endothelial injury, and hypercoagulability appear to be intertwined to drive the severe features of COVID-19.


##### Option

Scale:


100%

Image resolution:


 High

##### Background color

Organism

##### Search

##### ID search

##### Color


KGML

Image (png) file 1x

Image (png) file 2x
