# Supplementary material for: Multi-Omics and Experimental Validation Reveal the Protective Effect of Paeoniflorin Against Coronary Heart Disease in Mice via Inhibiting the C3-Cfd-C3aR Pathway
Source: Int J Mol Sci. 2026 Jul 13;27(14):6236. doi: 10.3390/ijms27146236 (PMC13410309; doi:10.3390/ijms27146236)
Supplement: Supplementary file 1 [file ijms-27-06236-s001.zip › Supplementary Materials/ijms-4276706_Proteomics_Dataset/8-KEGG_pathway_image/Model-vs-Paeoniflorin/mmu04360.html]

KEGG PATHWAY: Axon guidance - Mus musculus (house mouse)


# Axon guidance - Mus musculus (house mouse)


[
Pathway menu
| Organism menu
| Pathway entry
| Show description
| Download
| Help
]

Axon guidance represents a key stage in the formation of neuronal network. Axons are guided by a variety of guidance factors, such as netrins, ephrins, Slits, and semaphorins. These guidance cues are read by growth cone receptors, and signal transduction pathways downstream of these receptors converge onto the Rho GTPases to elicit changes in cytoskeletal organization that determine which way the growth cone will turn.


##### Option

Scale:


100%

Image resolution:


 High

##### Background color

Organism

##### Search

##### ID search

##### Color


KGML

Image (png) file 1x

Image (png) file 2x
