# Supplementary material for: Multi-Omics and Experimental Validation Reveal the Protective Effect of Paeoniflorin Against Coronary Heart Disease in Mice via Inhibiting the C3-Cfd-C3aR Pathway
Source: Int J Mol Sci. 2026 Jul 13;27(14):6236. doi: 10.3390/ijms27146236 (PMC13410309; doi:10.3390/ijms27146236)
Supplement: Supplementary file 1 [file ijms-27-06236-s001.zip › Supplementary Materials/ijms-4276706_Proteomics_Dataset/8-KEGG_pathway_image/Model-vs-Paeoniflorin/mmu04024.html]

KEGG PATHWAY: cAMP signaling pathway - Mus musculus (house mouse)


# cAMP signaling pathway - Mus musculus (house mouse)


[
Pathway menu
| Organism menu
| Pathway entry
| Show description
| Download
| Help
]

cAMP is one of the most common and universal second messengers, and its formation is promoted by adenylyl cyclase (AC) activation after ligation of G protein-coupled receptors (GPCRs) by ligands including hormones, neurotransmitters, and other signaling molecules. cAMP regulates pivotal physiologic processes including metabolism, secretion, calcium homeostasis, muscle contraction, cell fate, and gene transcription. cAMP acts directly on three main targets: protein kinase A (PKA), the exchange protein activated by cAMP (Epac), and cyclic nucleotide-gated ion channels (CNGCs). PKA modulates, via phosphorylation, a number of cellular substrates, including transcription factors, ion channels, transporters, exchangers, intracellular Ca2+ -handling proteins, and the contractile machinery. Epac proteins function as guanine nucleotide exchange factors (GEFs) for both Rap1 and Rap2. Various effector proteins, including adaptor proteins implicated in modulation of the actin cytoskeleton, regulators of G proteins of the Rho family, and phospholipases, relay signaling downstream from Rap.


##### Option

Scale:


100%

Image resolution:


 High

##### Background color

Organism

##### Search

##### ID search

##### Color


KGML

Image (png) file 1x

Image (png) file 2x
