# Supplementary material for: Multi-Omics and Experimental Validation Reveal the Protective Effect of Paeoniflorin Against Coronary Heart Disease in Mice via Inhibiting the C3-Cfd-C3aR Pathway
Source: Int J Mol Sci. 2026 Jul 13;27(14):6236. doi: 10.3390/ijms27146236 (PMC13410309; doi:10.3390/ijms27146236)
Supplement: Supplementary file 1 [file ijms-27-06236-s001.zip › Supplementary Materials/ijms-4276706_Proteomics_Dataset/8-KEGG_pathway_image/Model-vs-Paeoniflorin/mmu05320.html]

KEGG PATHWAY: Autoimmune thyroid disease - Mus musculus (house mouse)


# Autoimmune thyroid disease - Mus musculus (house mouse)


[
Pathway menu
| Organism menu
| Pathway entry
| Show description
| Download
| Help
]

The classification of autoimmune throid disease (AITD) includes Hashimoto's thyroiditis (HT) or chronic autoimmune thyroiditis and its variants, Graves' disease (GD) and autoimmune atrophic thyroiditis or primary myxedema. HT is characterized by the presence of goitre, thyroid autoantibodies against thyroid peroxidase (TPO) and thyroglobulin (Tg) in serum and varying degrees of thyroid dysfunction. During HT, self-reactive CD4+ T lymphocytes (Th) recruit B cells and CD8+ T cells (CTL) into the thyroid. Disease progression leads to the death of thyroid cells and hypothyroidism. Both autoantibodies and thyroid-specific cytotoxic T lymphocytes (CTLs) have been proposed to be responsible for autoimmune thyrocyte depletion. In GD, the TSH-R is the most important autoantigen. Antibodies directed against it mimic the effects of the hormone on thyroid cells, TSH, stimulating autonomous production of thyroxine and triiodothyronine and causing hyperthyroidism. The presence of TSH-R-blocking antibodies that bind the TSH receptor in a similar fashion to the antibodies in patients with Grave's disease but that block rather than activate the receptor explains some cases of atrophic hypothyroidism.


##### Option

Scale:


100%

Image resolution:


 High

##### Background color

Organism

##### Search

##### ID search

##### Color


KGML

Image (png) file 1x

Image (png) file 2x
