# Supplementary material for: Multi-Omics and Experimental Validation Reveal the Protective Effect of Paeoniflorin Against Coronary Heart Disease in Mice via Inhibiting the C3-Cfd-C3aR Pathway
Source: Int J Mol Sci. 2026 Jul 13;27(14):6236. doi: 10.3390/ijms27146236 (PMC13410309; doi:10.3390/ijms27146236)
Supplement: Supplementary file 1 [file ijms-27-06236-s001.zip › Supplementary Materials/ijms-4276706_Proteomics_Dataset/8-KEGG_pathway_image/Model-vs-Paeoniflorin/mmu04530.html]

KEGG PATHWAY: Tight junction - Mus musculus (house mouse)


# Tight junction - Mus musculus (house mouse)


[
Pathway menu
| Organism menu
| Pathway entry
| Show description
| Download
| Help
]

Tight junctions (TJs) are essential for establishing a selectively permeable barrier to diffusion through the paracellular space between neighboring cells. TJs are composed of at least three types of transmembrane protein -occludin, claudin and junctional adhesion molecules (JAMs)- and a cytoplasmic 'plaque' consisting of many different proteins that form large complexes. These are proposed to be involved in junction assembly, barrier regulation, cell polarity, gene transcription, and other pathways.


##### Option

Scale:


100%

Image resolution:


 High

##### Background color

Organism

##### Search

##### ID search

##### Color


KGML

Image (png) file 1x

Image (png) file 2x
