# Supplementary material for: Multi-Omics and Experimental Validation Reveal the Protective Effect of Paeoniflorin Against Coronary Heart Disease in Mice via Inhibiting the C3-Cfd-C3aR Pathway
Source: Int J Mol Sci. 2026 Jul 13;27(14):6236. doi: 10.3390/ijms27146236 (PMC13410309; doi:10.3390/ijms27146236)
Supplement: Supplementary file 1 [file ijms-27-06236-s001.zip › Supplementary Materials/ijms-4276706_Proteomics_Dataset/8-KEGG_pathway_image/Model-vs-Paeoniflorin/mmu04662.html]

KEGG PATHWAY: B cell receptor signaling pathway - Mus musculus (house mouse)


# B cell receptor signaling pathway - Mus musculus (house mouse)


[
Pathway menu
| Organism menu
| Pathway entry
| Show description
| Download
| Help
]

B cells are an important component of adaptive immunity. They produce and secrete millions of different antibody molecules, each of which recognizes a different (foreign) antigen. The B cell receptor (BCR) is an integral membrane protein complex that is composed of two immunoglobulin (Ig) heavy chains, two Ig light chains and two heterodimers of Ig-alpha and Ig-beta. After BCR ligation by antigen, three main protein tyrosine kinases (PTKs) -the SRC-family kinase LYN, SYK and the TEC-family kinase BTK- are activated. Phosphatidylinositol 3-kinase (PI3K) and phospholipase C-gamma 2 (PLC-gamma 2) are important downstream effectors of BCR signalling. This signalling ultimately results in the expression of immediate early genes that further activate the expression of other genes involved in B cell proliferation, differentiation and Ig production as well as other processes.


##### Option

Scale:


100%

Image resolution:


 High

##### Background color

Organism

##### Search

##### ID search

##### Color


KGML

Image (png) file 1x

Image (png) file 2x
