# Supplementary material for: Multi-Omics and Experimental Validation Reveal the Protective Effect of Paeoniflorin Against Coronary Heart Disease in Mice via Inhibiting the C3-Cfd-C3aR Pathway
Source: Int J Mol Sci. 2026 Jul 13;27(14):6236. doi: 10.3390/ijms27146236 (PMC13410309; doi:10.3390/ijms27146236)
Supplement: Supplementary file 1 [file ijms-27-06236-s001.zip › Supplementary Materials/ijms-4276706_Proteomics_Dataset/8-KEGG_pathway_image/Model-vs-Paeoniflorin/mmu05100.html]

KEGG PATHWAY: Bacterial invasion of epithelial cells - Mus musculus (house mouse)


# Bacterial invasion of epithelial cells - Mus musculus (house mouse)


[
Pathway menu
| Organism menu
| Pathway entry
| Show description
| Download
| Help
]

Many pathogenic bacteria can invade phagocytic and non-phagocytic cells and colonize them intracellularly, then become disseminated to other cells. Invasive bacteria induce their own uptake by non-phagocytic host cells (e.g. epithelial cells) using two mechanisms referred to as zipper model and trigger model. Listeria, Staphylococcus, Streptococcus, and Yersinia are examples of bacteria that enter using the zipper model. These bacteria express proteins on their surfaces that interact with cellular receptors, initiating signalling cascades that result in close apposition of the cellular membrane around the entering bacteria. Shigella and Salmonella are the examples of bacteria entering cells using the trigger model. These bacteria use type III secretion systems to inject protein effectors that interact with the actin cytoskeleton.


##### Option

Scale:


100%

Image resolution:


 High

##### Background color

Organism

##### Search

##### ID search

##### Color


KGML

Image (png) file 1x

Image (png) file 2x
