# Supplementary material for: Multi-Omics and Experimental Validation Reveal the Protective Effect of Paeoniflorin Against Coronary Heart Disease in Mice via Inhibiting the C3-Cfd-C3aR Pathway
Source: Int J Mol Sci. 2026 Jul 13;27(14):6236. doi: 10.3390/ijms27146236 (PMC13410309; doi:10.3390/ijms27146236)
Supplement: Supplementary file 1 [file ijms-27-06236-s001.zip › Supplementary Materials/ijms-4276706_Proteomics_Dataset/8-KEGG_pathway_image/Model-vs-Paeoniflorin/mmu03040.html]

KEGG PATHWAY: Spliceosome - Mus musculus (house mouse)


# Spliceosome - Mus musculus (house mouse)


[
Pathway menu
| Organism menu
| Pathway entry
| Show description
| Download
| Help
]

After transcription, eukaryotic mRNA precursors contain protein-coding exons and noncoding introns. In the following splicing, introns are excised and exons are joined by a macromolecular complex, the spliceosome. The standard spliceosome is made up of five small nuclear ribonucleoproteins (snRNPs), U1, U2, U4, U5, and U6 snRNPs, and several spliceosome-associated proteins (SAPs). Spliceosomes are not a simple stable complex, but a dynamic family of particles that assemble on the mRNA precursor and help fold it into a conformation that allows transesterification to proceed. Various spliceosome forms (e.g. A-, B- and C-complexes) have been identified.


##### Option

Scale:


100%

Image resolution:


 High

##### Background color

Organism

##### Search

##### ID search

##### Color

##### Related Brite

- 03041  Spliceosome


KGML

Image (png) file 1x

Image (png) file 2x
