# Supplementary material for: Multi-Omics and Experimental Validation Reveal the Protective Effect of Paeoniflorin Against Coronary Heart Disease in Mice via Inhibiting the C3-Cfd-C3aR Pathway
Source: Int J Mol Sci. 2026 Jul 13;27(14):6236. doi: 10.3390/ijms27146236 (PMC13410309; doi:10.3390/ijms27146236)
Supplement: Supplementary file 1 [file ijms-27-06236-s001.zip › Supplementary Materials/ijms-4276706_Proteomics_Dataset/8-KEGG_pathway_image/Model-vs-Paeoniflorin/mmu05205.html]

KEGG PATHWAY: Proteoglycans in cancer - Mus musculus (house mouse)


# Proteoglycans in cancer - Mus musculus (house mouse)


[
Pathway menu
| Organism menu
| Pathway entry
| Show description
| Download
| Help
]

Many proteoglycans (PGs) in the tumor microenvironment have been shown to be key macromolecules that contribute to biology of various types of cancer including proliferation, adhesion, angiogenesis and metastasis, affecting tumor progress. The four main types of proteoglycans include hyaluronan (HA), which does not occur as a PG but in free form, heparan sulfate proteoglycans (HSPGs), chondroitin sulfate proteoglycans (CSPGs), dematan sulfate proteoglycans (DSPG) and keratan sulfate proteoglycans (KSPGs) [BR:00535]. Among these proteoglycans such as HA, acting with CD44, promotes tumor cell growth and migration, whereas other proteoglycans such as syndecans (-1~-4), glypican (-1, -3) and perlecan may interact with growth factors, cytokines, morphogens and enzymes through HS chains [BR:00536], also leading to tumor growth and invasion. In contrast, some of the small leucine-rich proteolgycans, such as decorin and lumican, can function as tumor repressors, and modulate the signaling pathways by the interaction of their core proteins and multiple receptors.


##### Option

Scale:


100%

Image resolution:


 High

##### Background color

Organism

##### Search

##### ID search

##### Color


KGML

Image (png) file 1x

Image (png) file 2x
