# Supplementary material for: Multi-Omics and Experimental Validation Reveal the Protective Effect of Paeoniflorin Against Coronary Heart Disease in Mice via Inhibiting the C3-Cfd-C3aR Pathway
Source: Int J Mol Sci. 2026 Jul 13;27(14):6236. doi: 10.3390/ijms27146236 (PMC13410309; doi:10.3390/ijms27146236)
Supplement: Supplementary file 1 [file ijms-27-06236-s001.zip › Supplementary Materials/ijms-4276706_Proteomics_Dataset/8-KEGG_pathway_image/Model-vs-Paeoniflorin/mmu05210.html]

KEGG PATHWAY: Colorectal cancer - Mus musculus (house mouse)


# Colorectal cancer - Mus musculus (house mouse)


[
Pathway menu
| Organism menu
| Pathway entry
| Show description
| Download
| Help
]

Colorectal cancer (CRC) is the second largest cause of cancer-related deaths in Western countries. CRC arises from the colorectal epithelium as a result of the accumulation of genetic alterations in defined oncogenes and tumour suppressor genes (TSG). Two major mechanisms of genomic instability have been identified in sporadic CRC progression. The first, known as chromosomal instability (CIN), results from a series of genetic changes that involve the activation of oncogenes such as K-ras and inactivation of TSG such as p53, DCC/Smad4, and APC. The second, known as microsatellite instability (MSI), results from inactivation of the DNA mismatch repair genes MLH1 and/or MSH2 by hypermethylation of their promoter, and secondary mutation of genes with coding microsatellites, such as transforming growth factor receptor II (TGF-RII) and BAX. Hereditary syndromes have germline mutations in specific genes (mutation in the tumour suppressor gene APC on chromosome 5q in FAP, mutated DNA mismatch repair genes in HNPCC).


##### Option

Scale:


100%

Image resolution:


 High

##### Background color

Organism

##### Search

##### ID search

##### Color


KGML

Image (png) file 1x

Image (png) file 2x
