# Supplementary figures and images for: Multi-Omics and Experimental Validation Reveal the Protective Effect of Paeoniflorin Against Coronary Heart Disease in Mice via Inhibiting the C3-Cfd-C3aR Pathway (part 2 of 2)
Source: Int J Mol Sci. 2026 Jul 13;27(14):6236. doi: 10.3390/ijms27146236 (PMC13410309; doi:10.3390/ijms27146236)

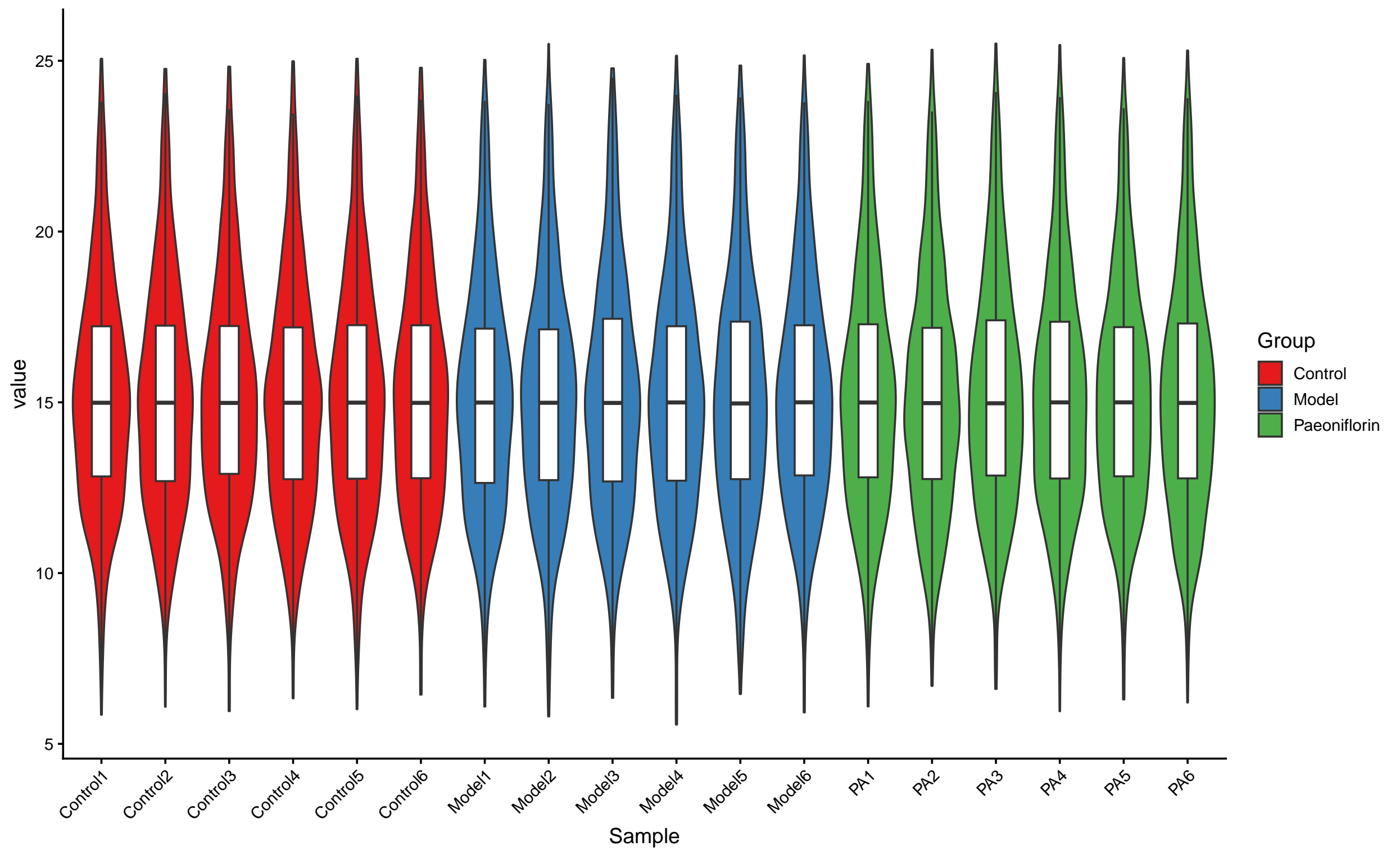

Supplement: Supplementary file 1 [file ijms-27-06236-s001.zip › Supplementary Materials/ijms-4276706_Metabolomics_Dataset/1-MS_identified_summary/Quantification_QC/Figure 1a. Metabolites quantitation density plot.pdf]

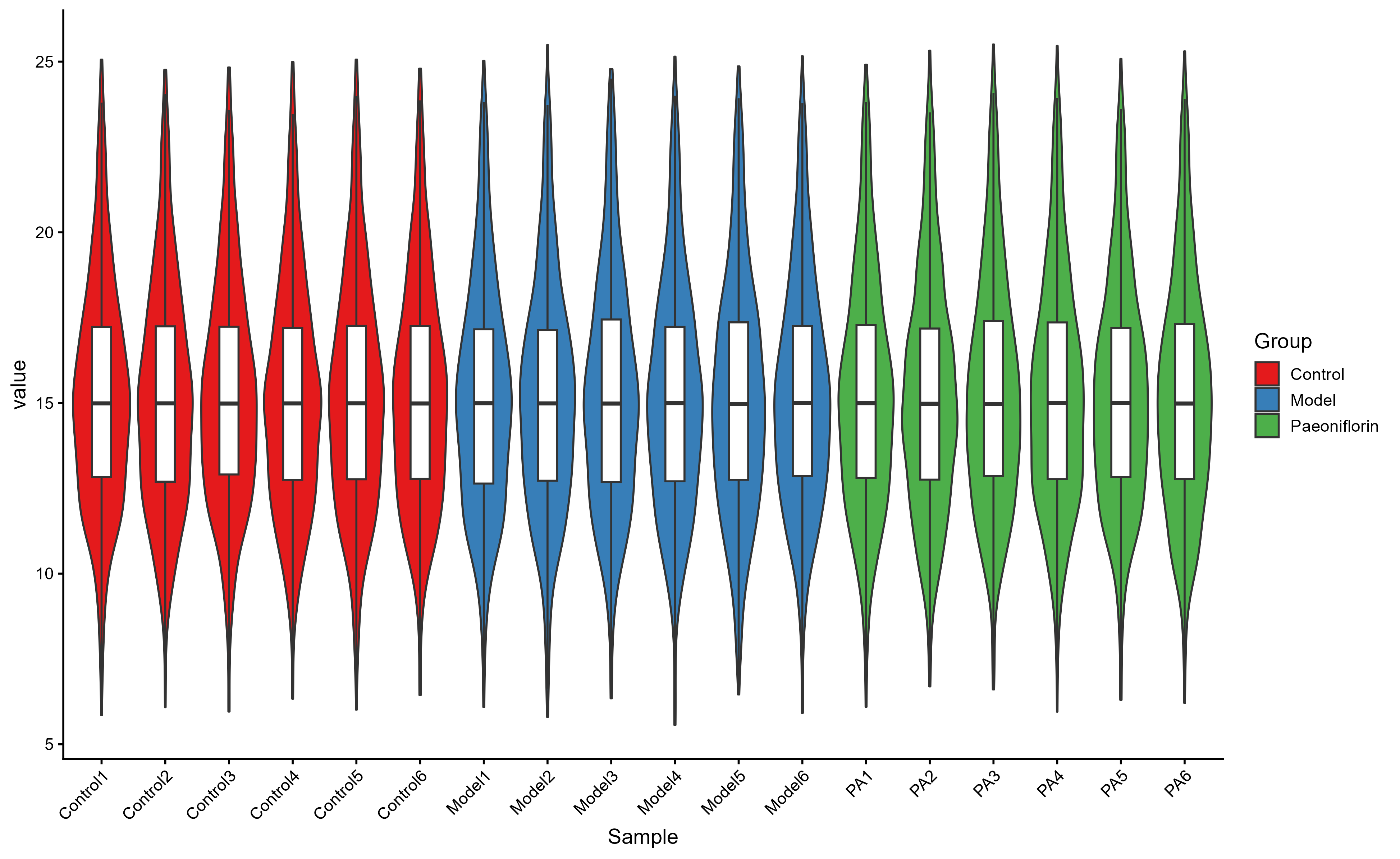

Supplement: Supplementary file 1 [file ijms-27-06236-s001.zip › Supplementary Materials/ijms-4276706_Metabolomics_Dataset/1-MS_identified_summary/Quantification_QC/Figure 1a. Metabolites quantitation density plot.png]

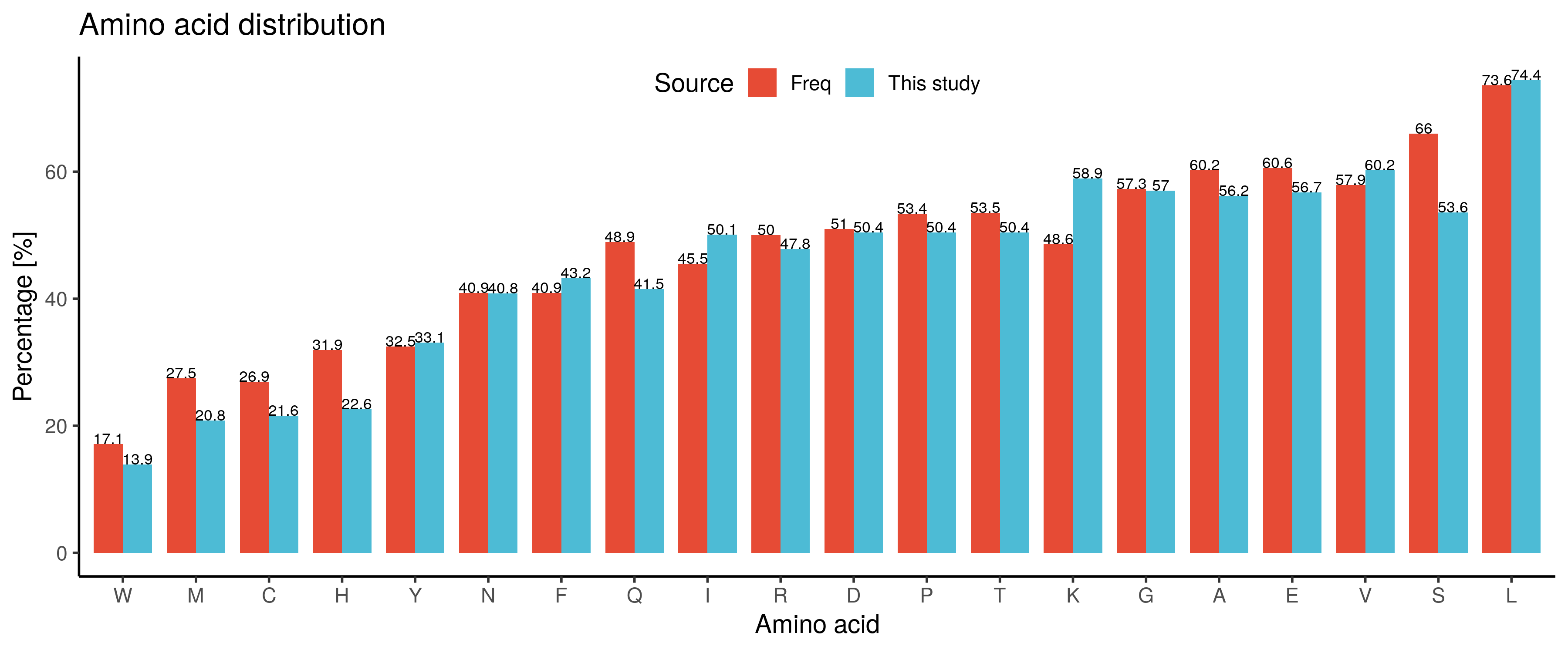

Supplement: Supplementary file 1 [file ijms-27-06236-s001.zip › Supplementary Materials/ijms-4276706_Proteomics_Dataset/1-MS_identified_summary/Identification_QC/Figure QC4 All.png]

# Amino acid distribution

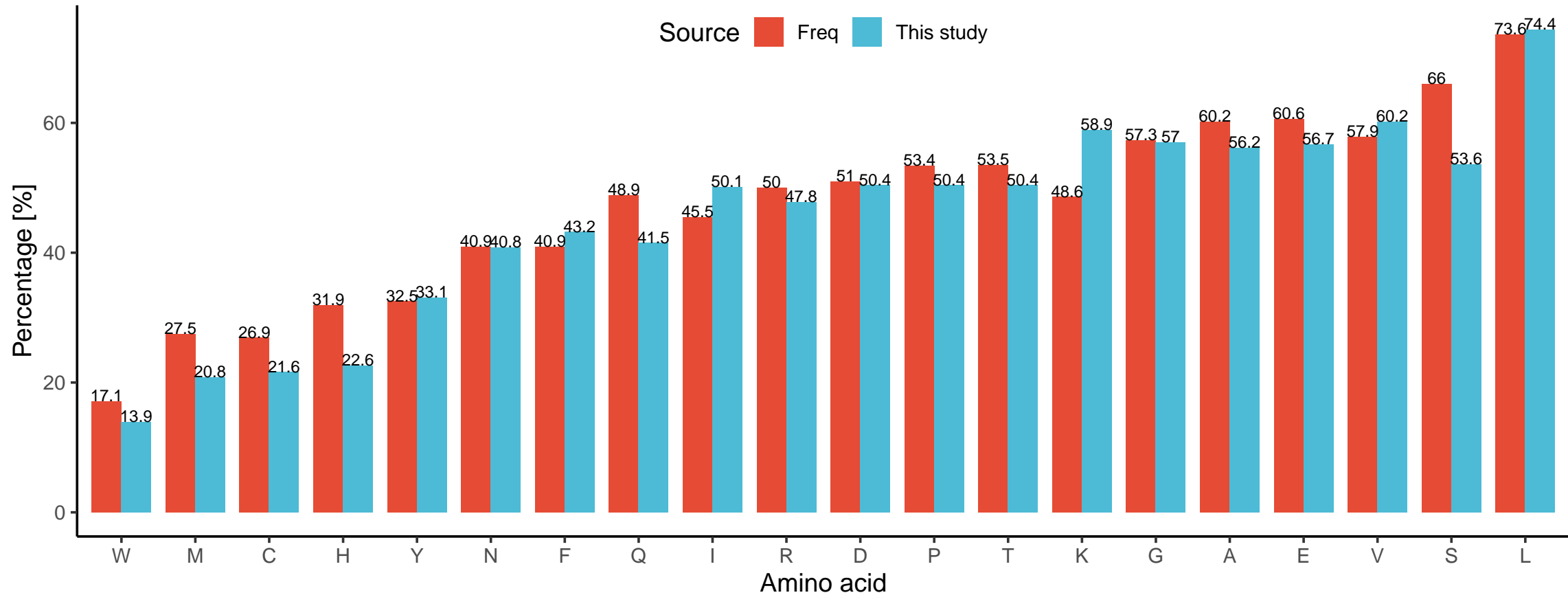

Supplement: Supplementary file 1 [file ijms-27-06236-s001.zip › Supplementary Materials/ijms-4276706_Proteomics_Dataset/1-MS_identified_summary/Identification_QC/Figure QC4 All.pdf]

Peptide mass tolerance distribution

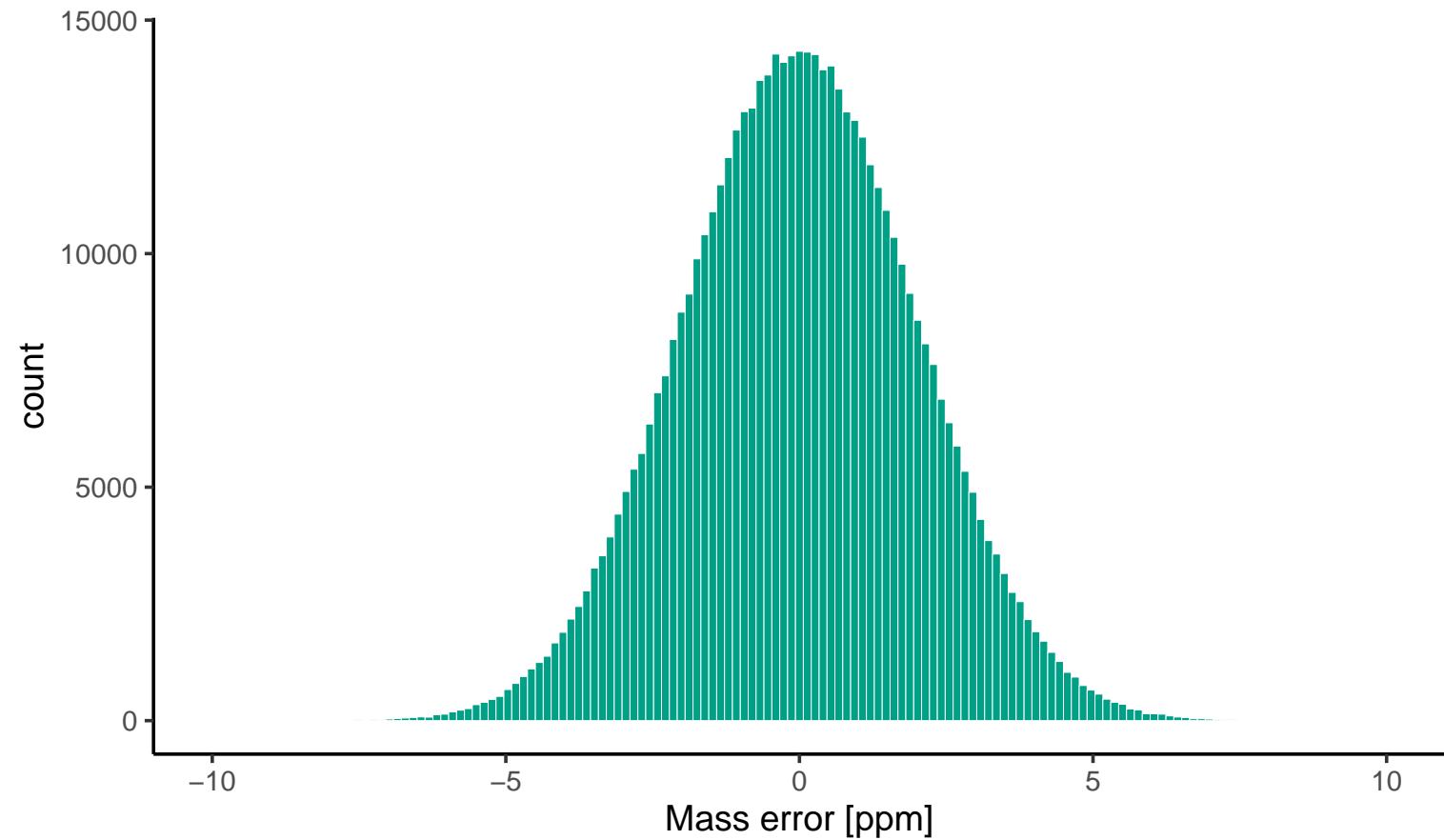

Supplement: Supplementary file 1 [file ijms-27-06236-s001.zip › Supplementary Materials/ijms-4276706_Proteomics_Dataset/1-MS_identified_summary/Identification_QC/Figure QC1 All.pdf]

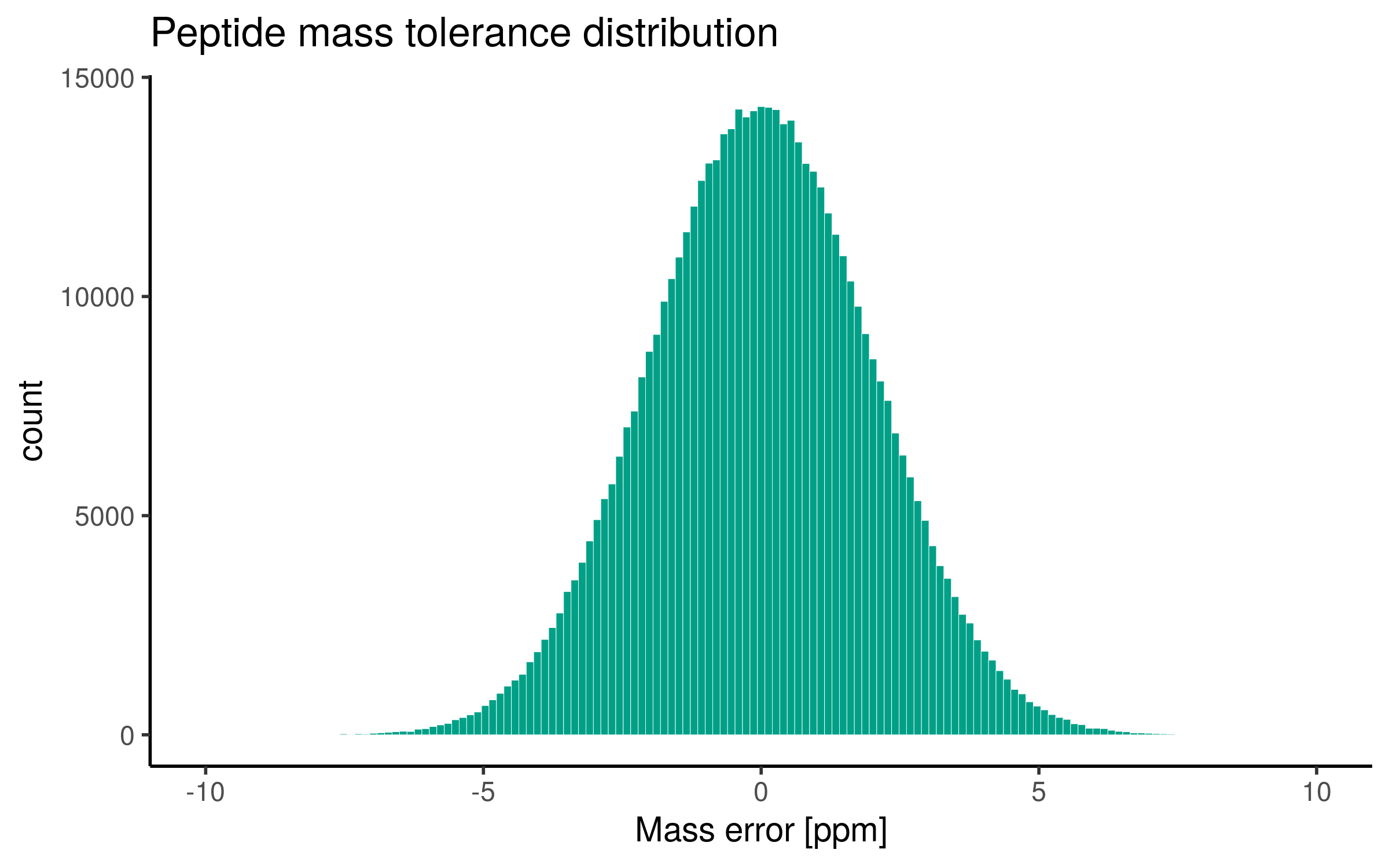

Supplement: Supplementary file 1 [file ijms-27-06236-s001.zip › Supplementary Materials/ijms-4276706_Proteomics_Dataset/1-MS_identified_summary/Identification_QC/Figure QC1 All.png]

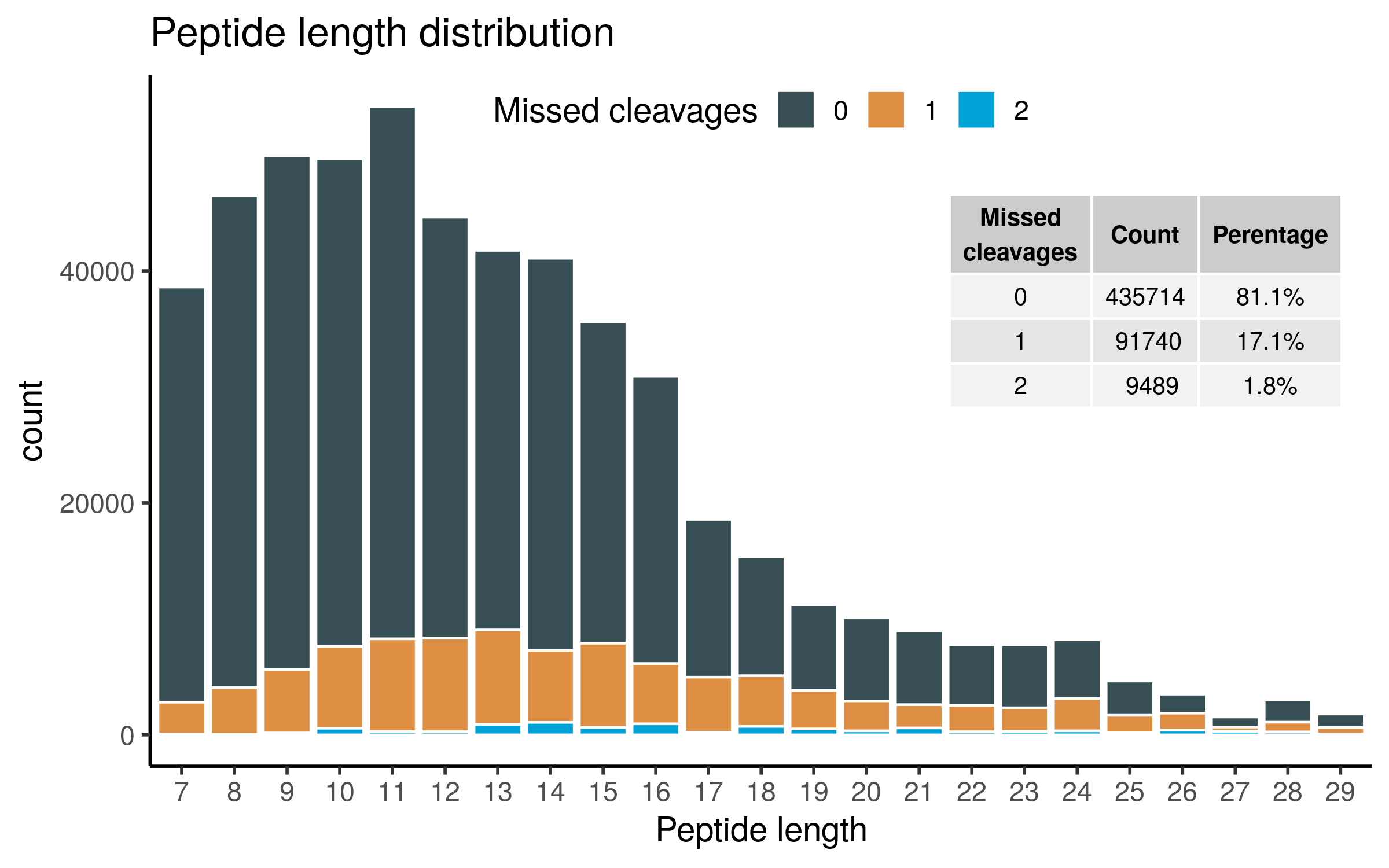

Supplement: Supplementary file 1 [file ijms-27-06236-s001.zip › Supplementary Materials/ijms-4276706_Proteomics_Dataset/1-MS_identified_summary/Identification_QC/Figure QC2 All.png]

# Peptide length distribution

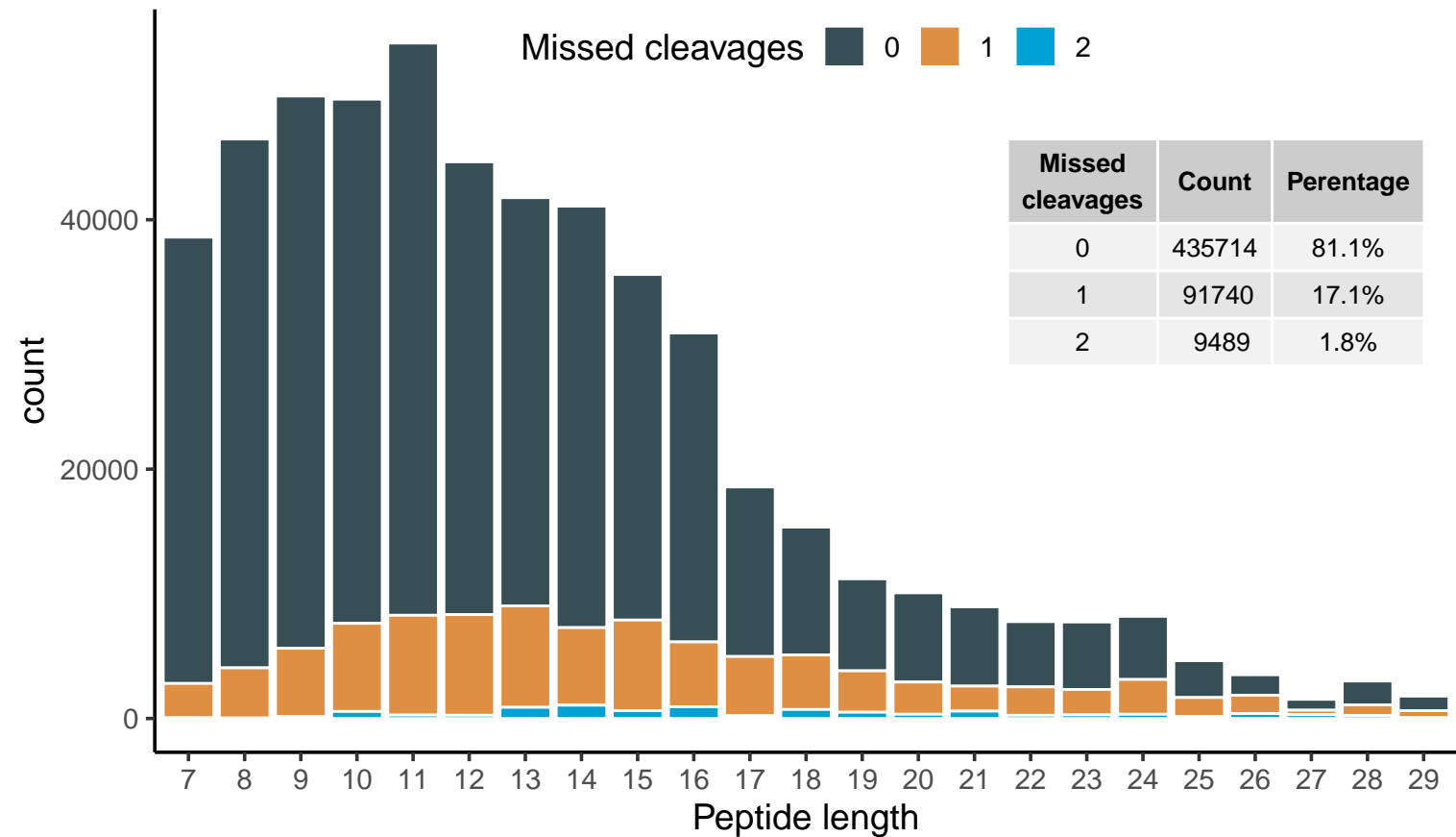

Supplement: Supplementary file 1 [file ijms-27-06236-s001.zip › Supplementary Materials/ijms-4276706_Proteomics_Dataset/1-MS_identified_summary/Identification_QC/Figure QC2 All.pdf]

# Intensity distribution

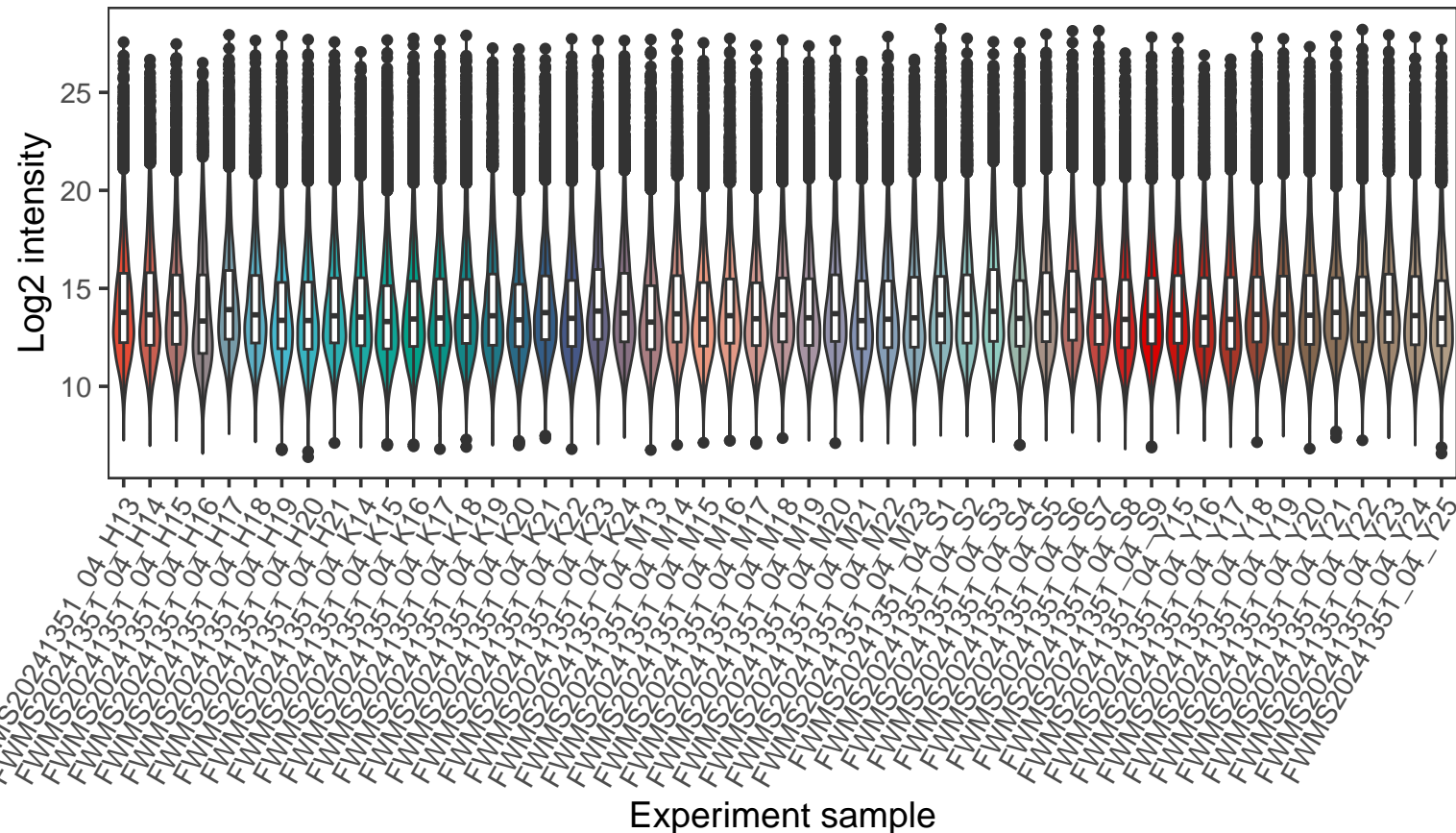

Supplement: Supplementary file 1 [file ijms-27-06236-s001.zip › Supplementary Materials/ijms-4276706_Proteomics_Dataset/1-MS_identified_summary/Identification_QC/Figure QC3 All.pdf]

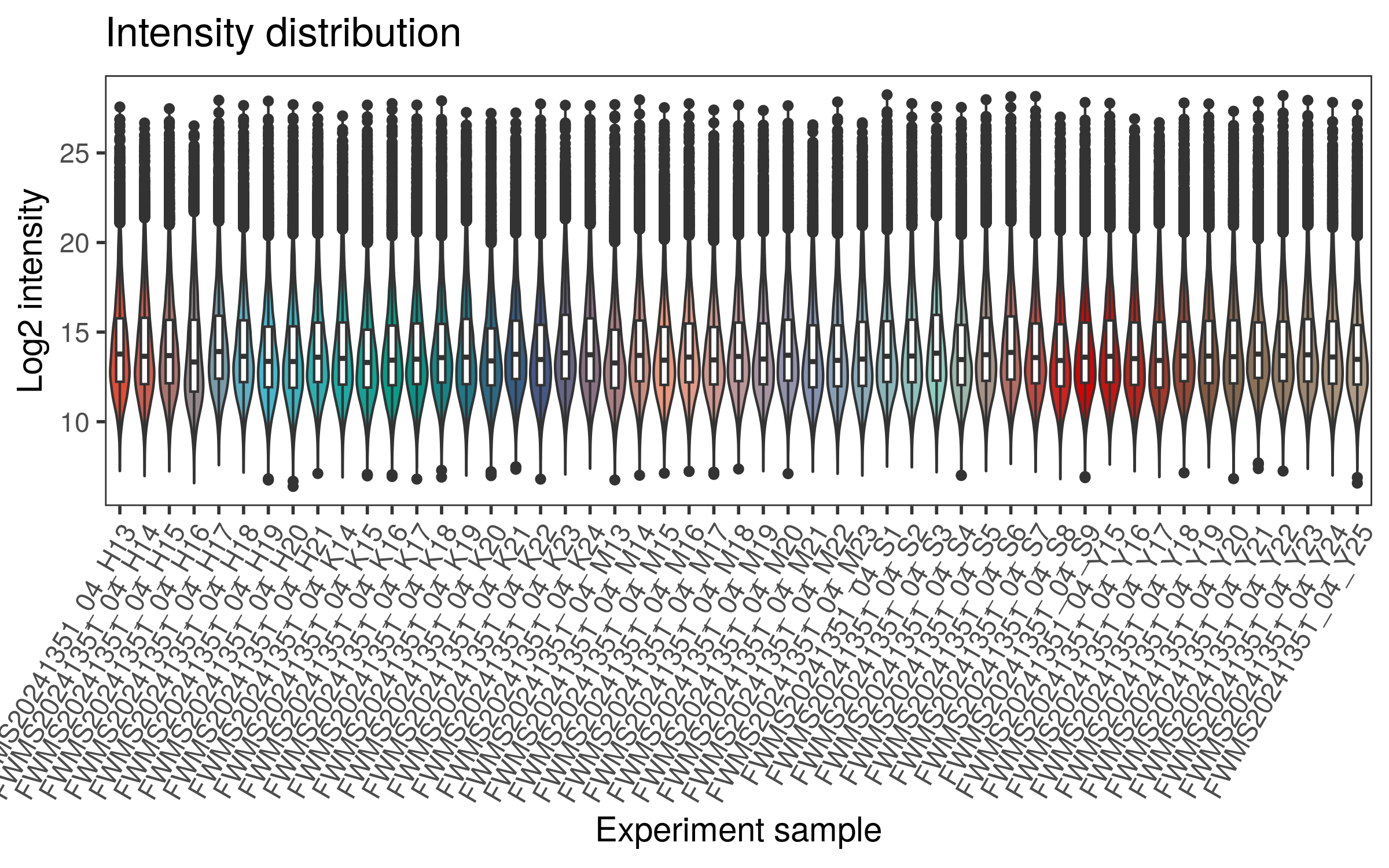

Supplement: Supplementary file 1 [file ijms-27-06236-s001.zip › Supplementary Materials/ijms-4276706_Proteomics_Dataset/1-MS_identified_summary/Identification_QC/Figure QC3 All.png]

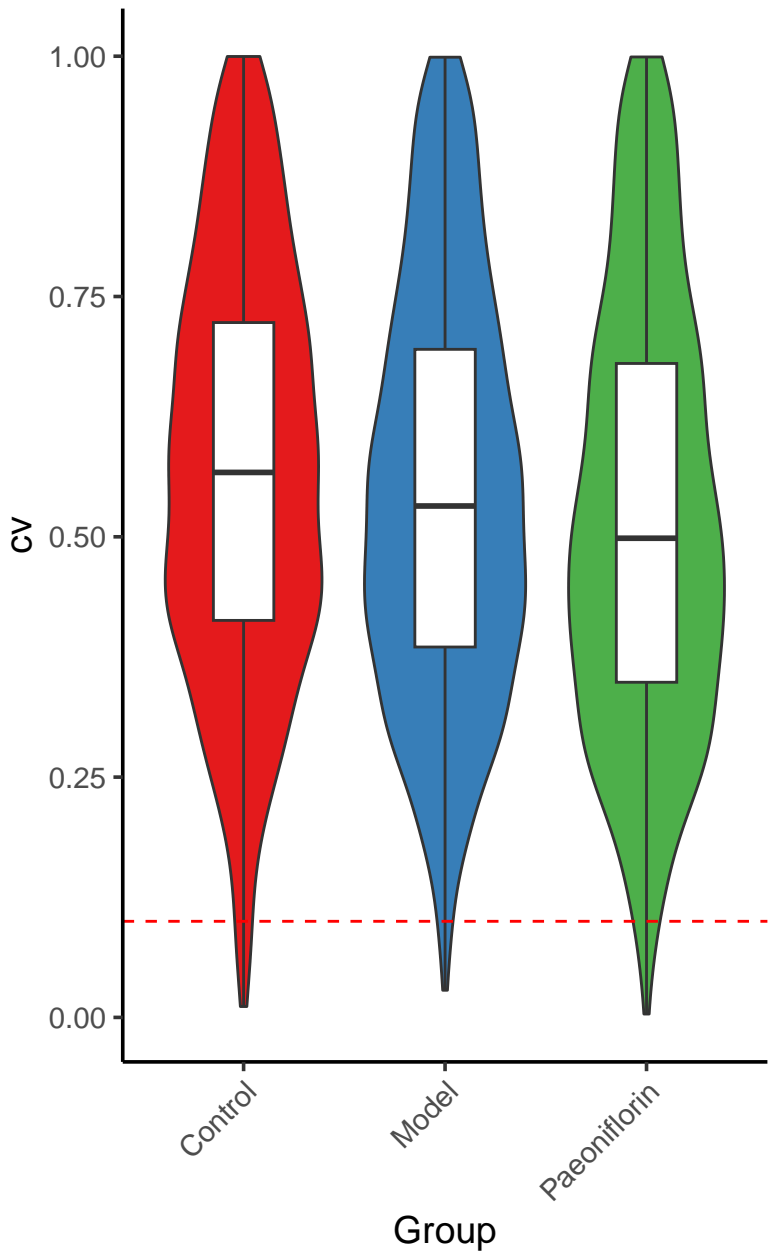

Supplement: Supplementary file 1 [file ijms-27-06236-s001.zip › Supplementary Materials/ijms-4276706_Proteomics_Dataset/1-MS_identified_summary/Quantification_QC/Figure 1b. Protein quantitation CV plot.pdf]

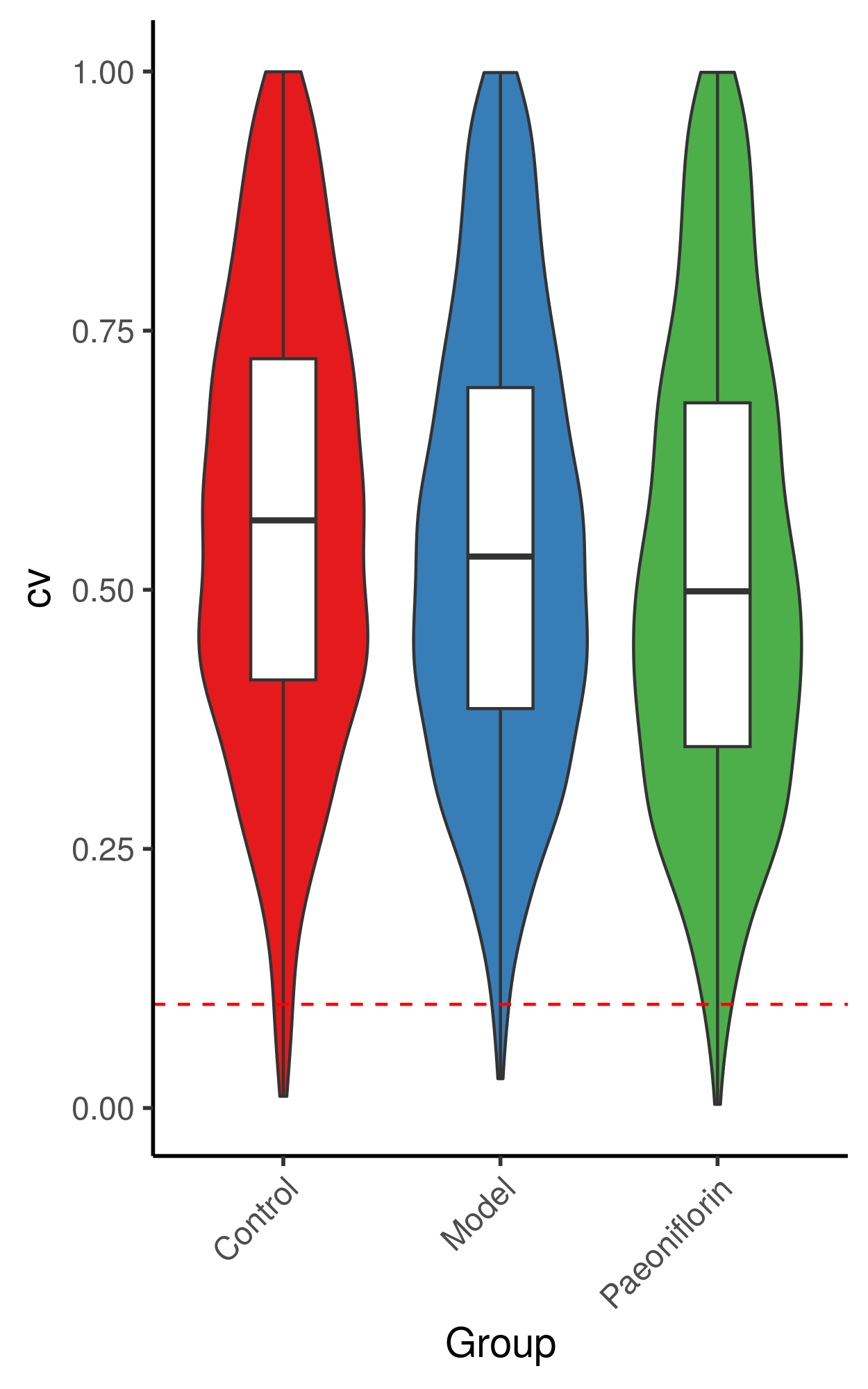

Supplement: Supplementary file 1 [file ijms-27-06236-s001.zip › Supplementary Materials/ijms-4276706_Proteomics_Dataset/1-MS_identified_summary/Quantification_QC/Figure 1b. Protein quantitation CV plot.png]

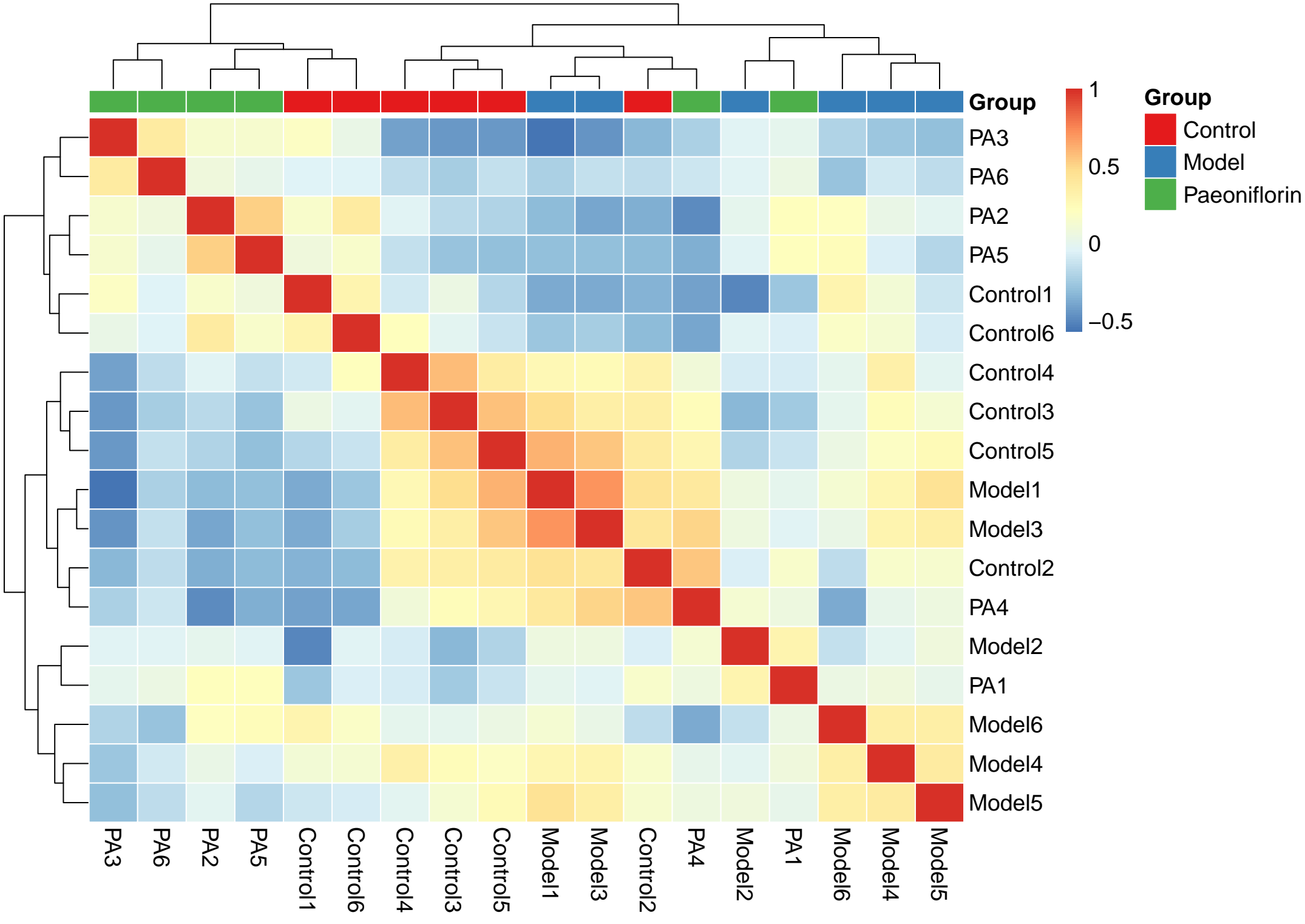

Supplement: Supplementary file 1 [file ijms-27-06236-s001.zip › Supplementary Materials/ijms-4276706_Proteomics_Dataset/1-MS_identified_summary/Quantification_QC/Figure 1d. Protein quantitation PCC plot.pdf]

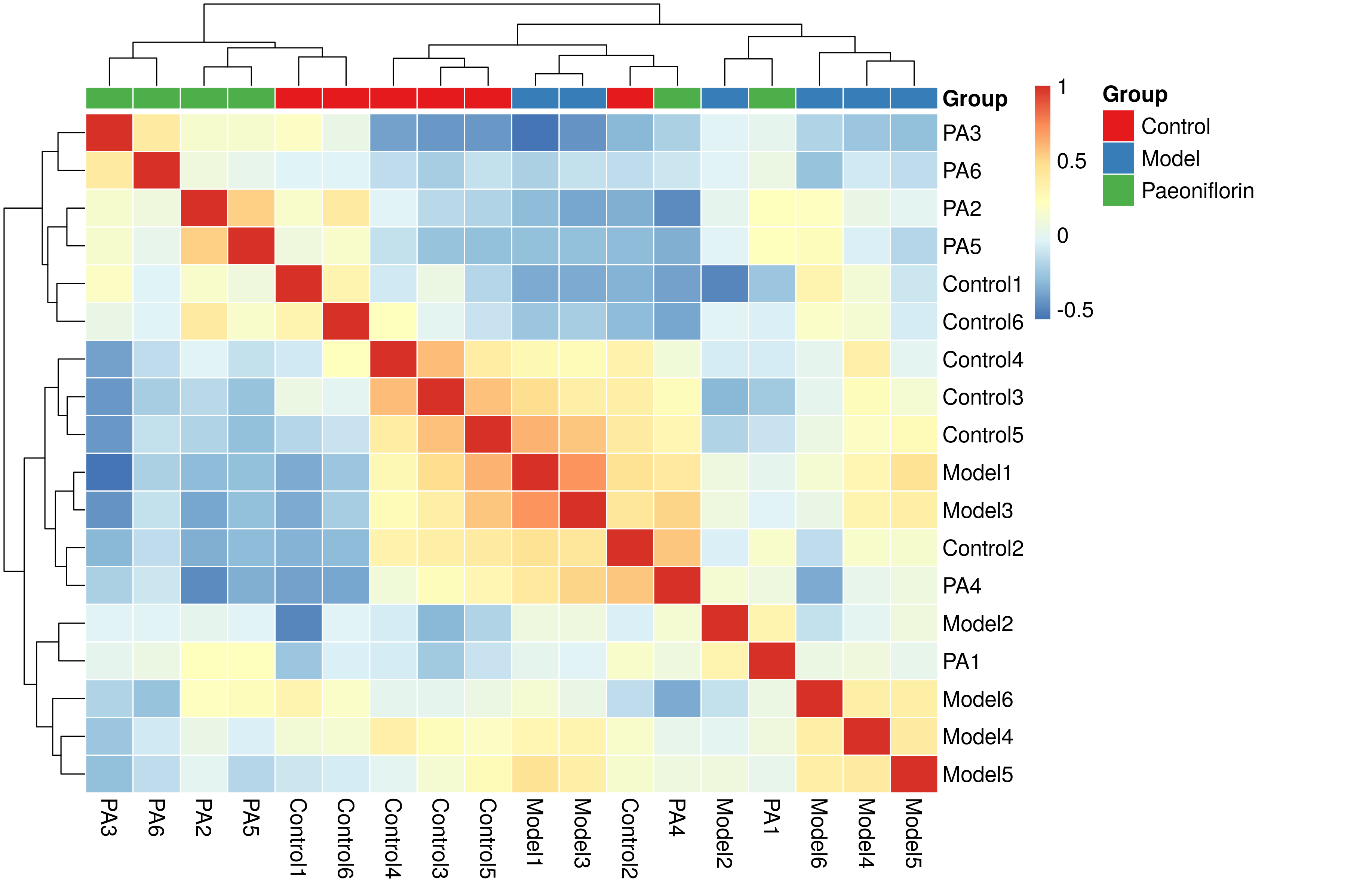

Supplement: Supplementary file 1 [file ijms-27-06236-s001.zip › Supplementary Materials/ijms-4276706_Proteomics_Dataset/1-MS_identified_summary/Quantification_QC/Figure 1d. Protein quantitation PCC plot.png]

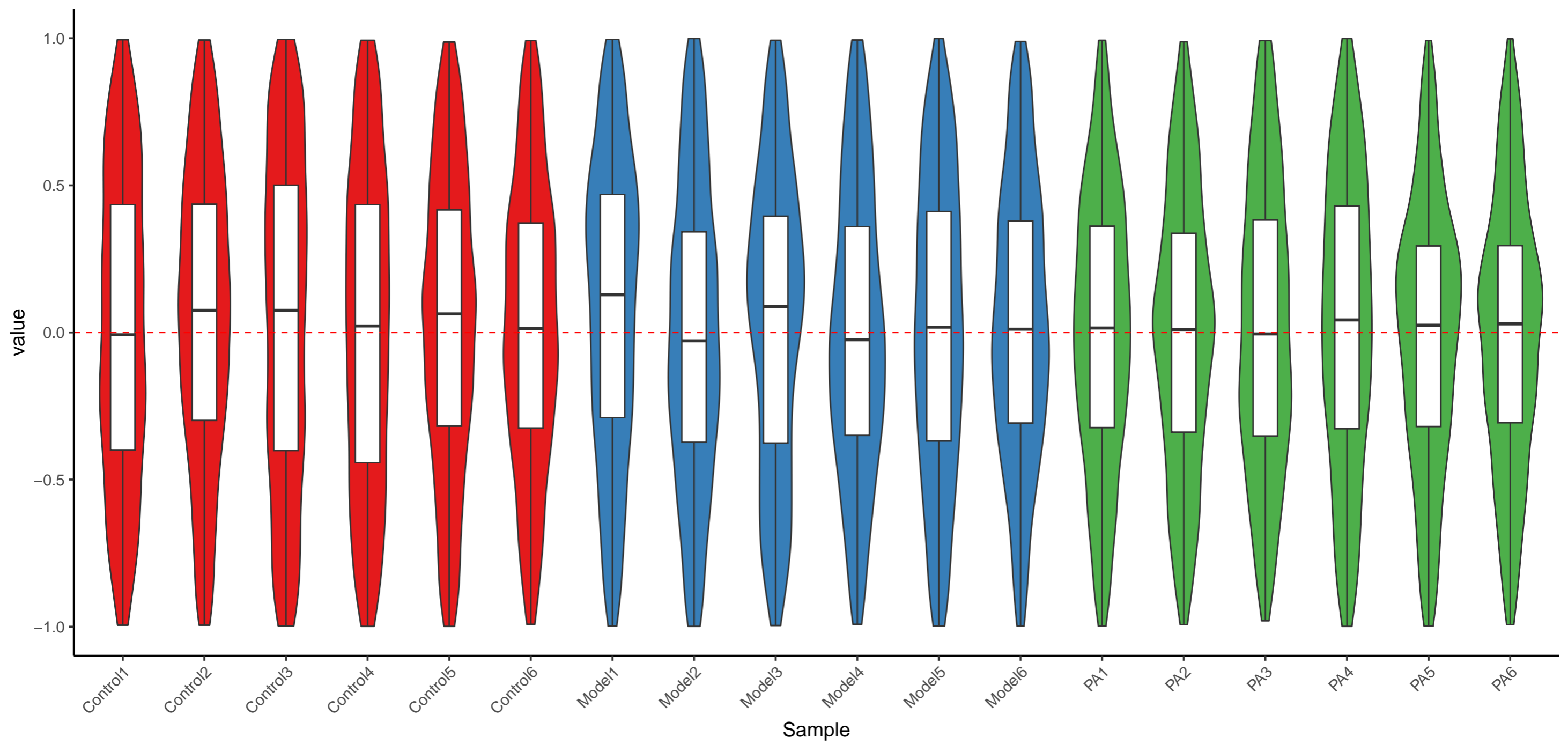

Supplement: Supplementary file 1 [file ijms-27-06236-s001.zip › Supplementary Materials/ijms-4276706_Proteomics_Dataset/1-MS_identified_summary/Quantification_QC/Figure 1a. Protein quantitation density plot.pdf]

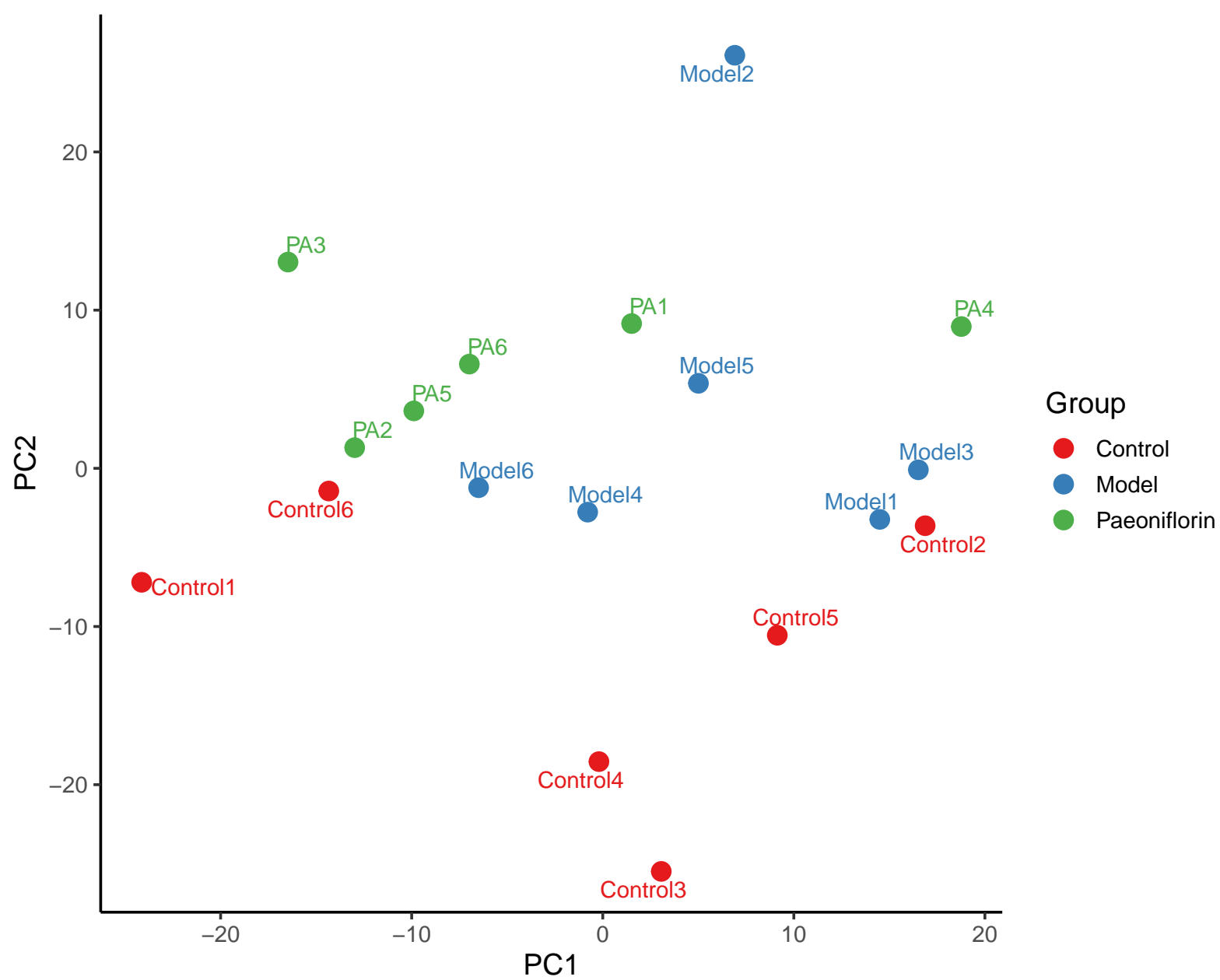

Supplement: Supplementary file 1 [file ijms-27-06236-s001.zip › Supplementary Materials/ijms-4276706_Proteomics_Dataset/1-MS_identified_summary/Quantification_QC/Figure 1c. Protein quantitation PCA plot.pdf]

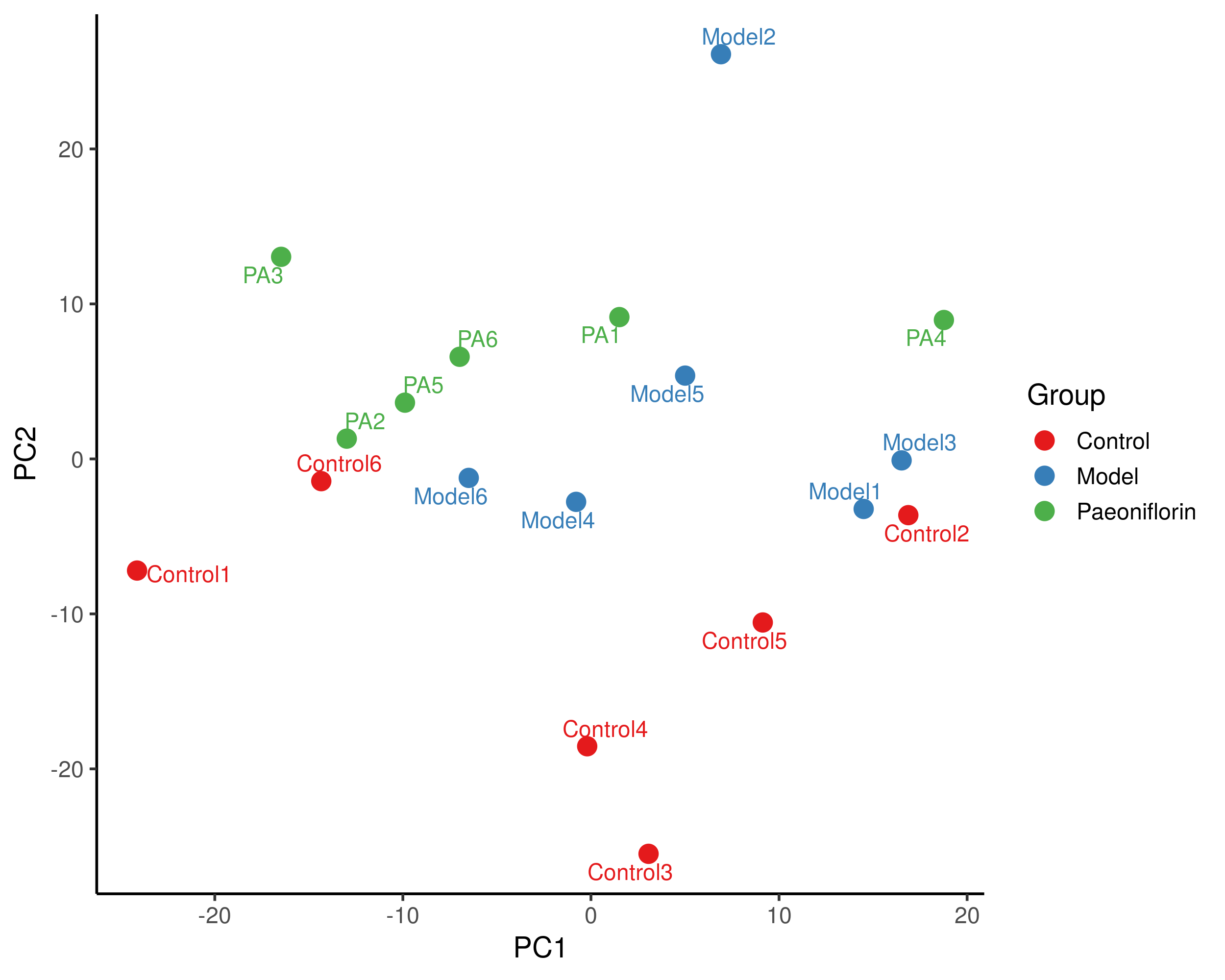

Supplement: Supplementary file 1 [file ijms-27-06236-s001.zip › Supplementary Materials/ijms-4276706_Proteomics_Dataset/1-MS_identified_summary/Quantification_QC/Figure 1c. Protein quantitation PCA plot.png]

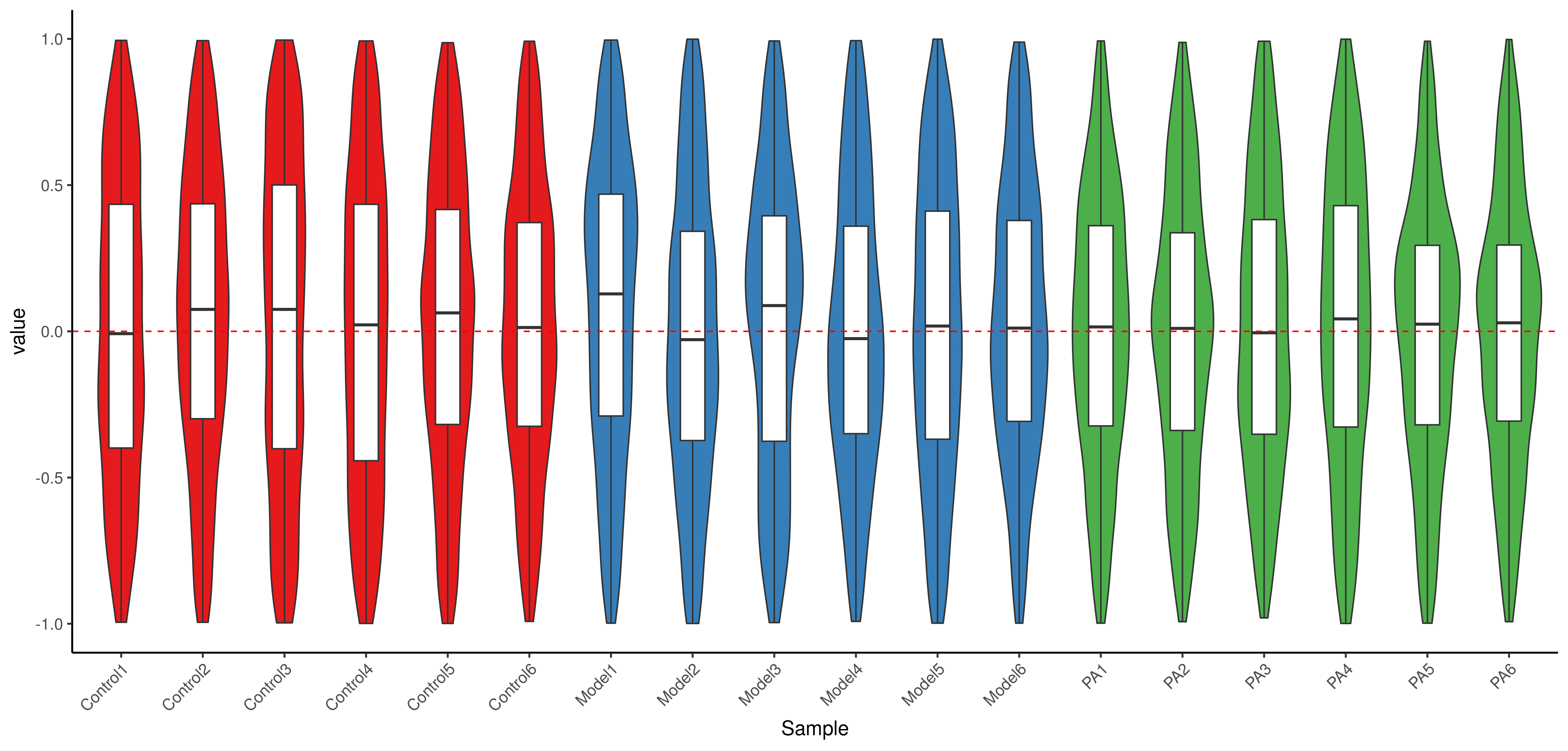

Supplement: Supplementary file 1 [file ijms-27-06236-s001.zip › Supplementary Materials/ijms-4276706_Proteomics_Dataset/1-MS_identified_summary/Quantification_QC/Figure 1a. Protein quantitation density plot.png]
