# Supplementary figures and images for: Multi-Omics and Experimental Validation Reveal the Protective Effect of Paeoniflorin Against Coronary Heart Disease in Mice via Inhibiting the C3-Cfd-C3aR Pathway (part 1 of 2)
Source: Int J Mol Sci. 2026 Jul 13;27(14):6236. doi: 10.3390/ijms27146236 (PMC13410309; doi:10.3390/ijms27146236)

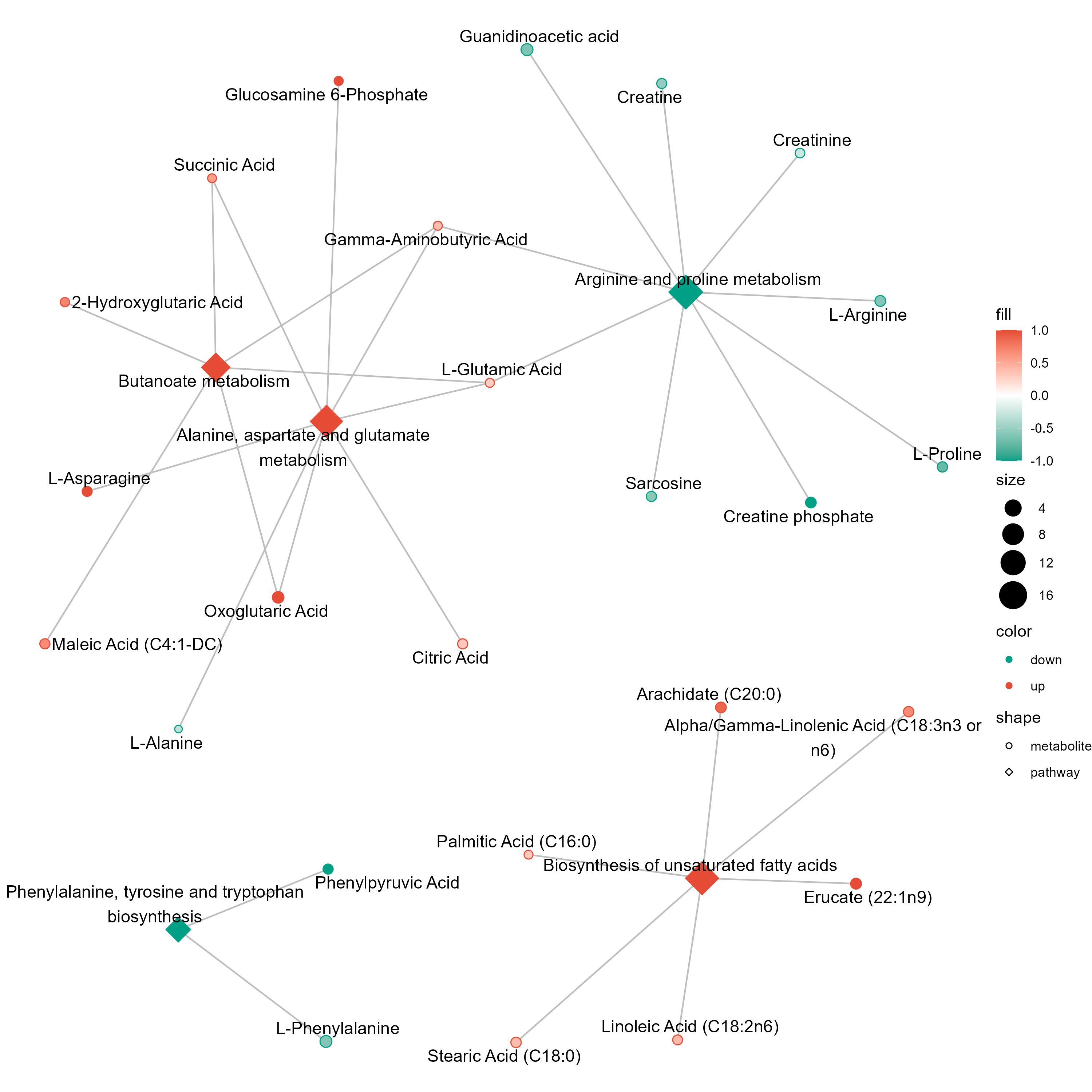

Supplement: Supplementary file 1 [file ijms-27-06236-s001.zip › Supplementary Materials/ijms-4276706_Metabolomics_Dataset/5-Pathway Network Analysis/Figure 5. Pathway network plot of Model-vs-Paeoniflorin.png]

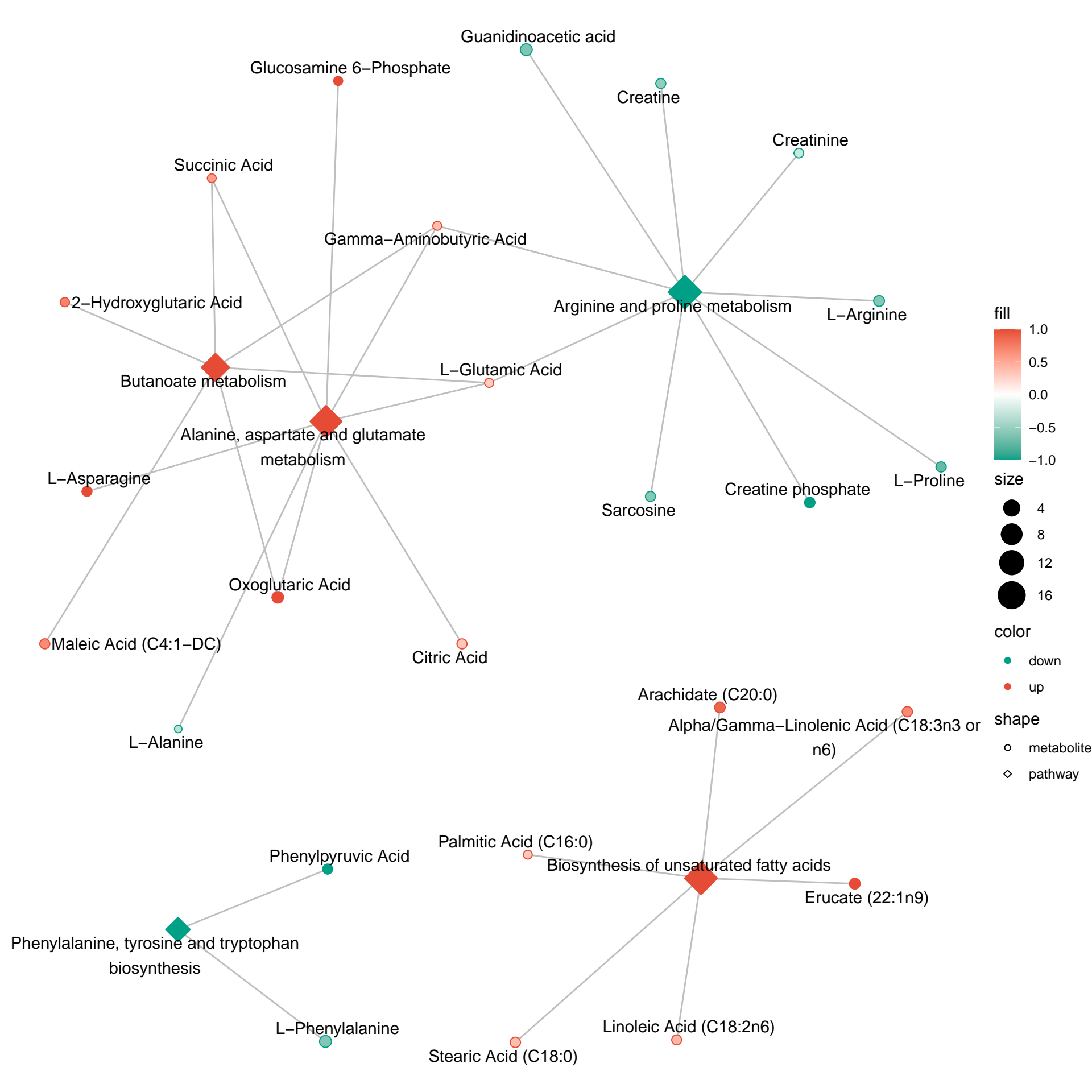

Supplement: Supplementary file 1 [file ijms-27-06236-s001.zip › Supplementary Materials/ijms-4276706_Metabolomics_Dataset/5-Pathway Network Analysis/Figure 5. Pathway network plot of Model-vs-Paeoniflorin.pdf]

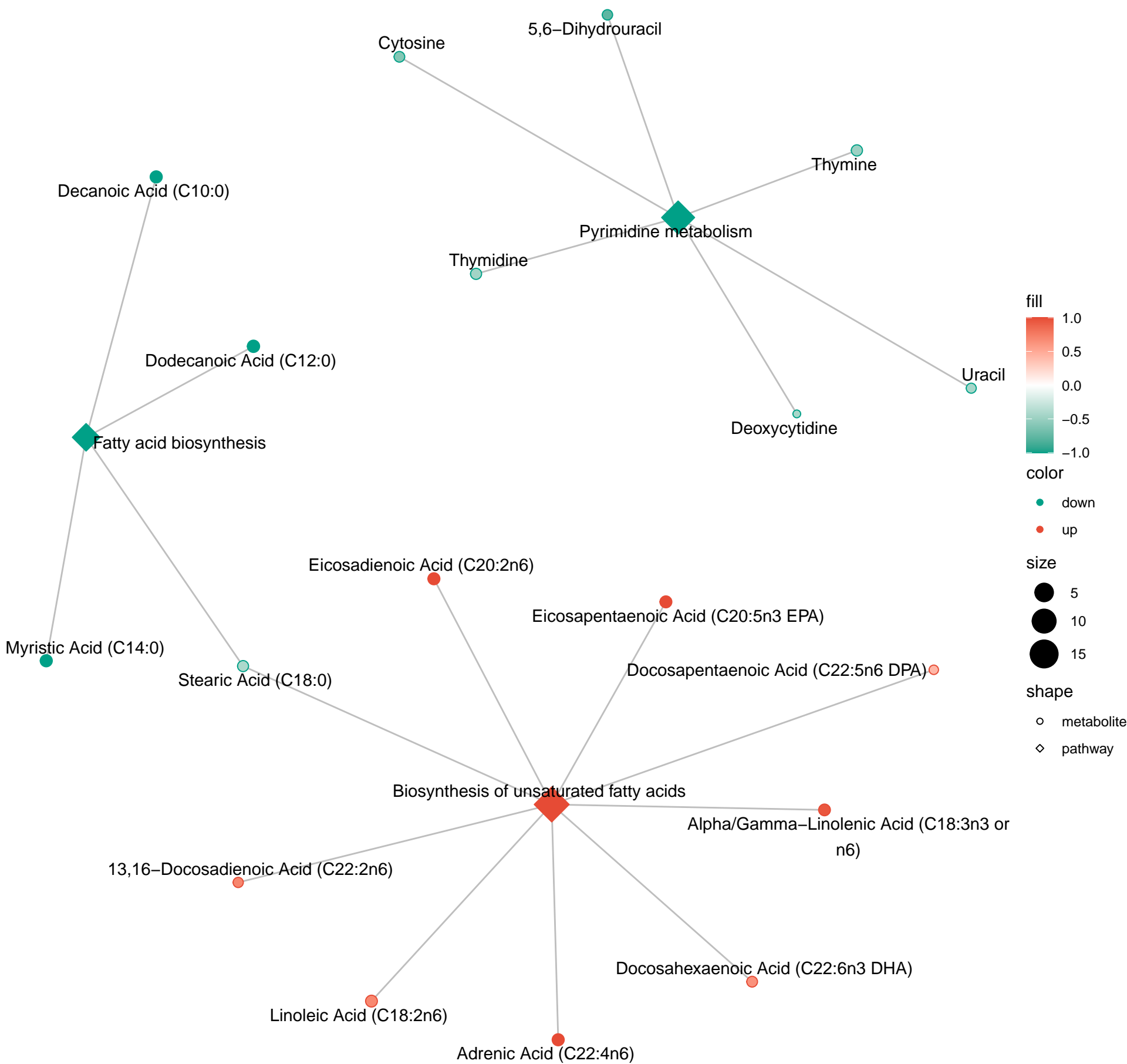

Supplement: Supplementary file 1 [file ijms-27-06236-s001.zip › Supplementary Materials/ijms-4276706_Metabolomics_Dataset/5-Pathway Network Analysis/Figure 5. Pathway network plot of Control-vs-Model.pdf]

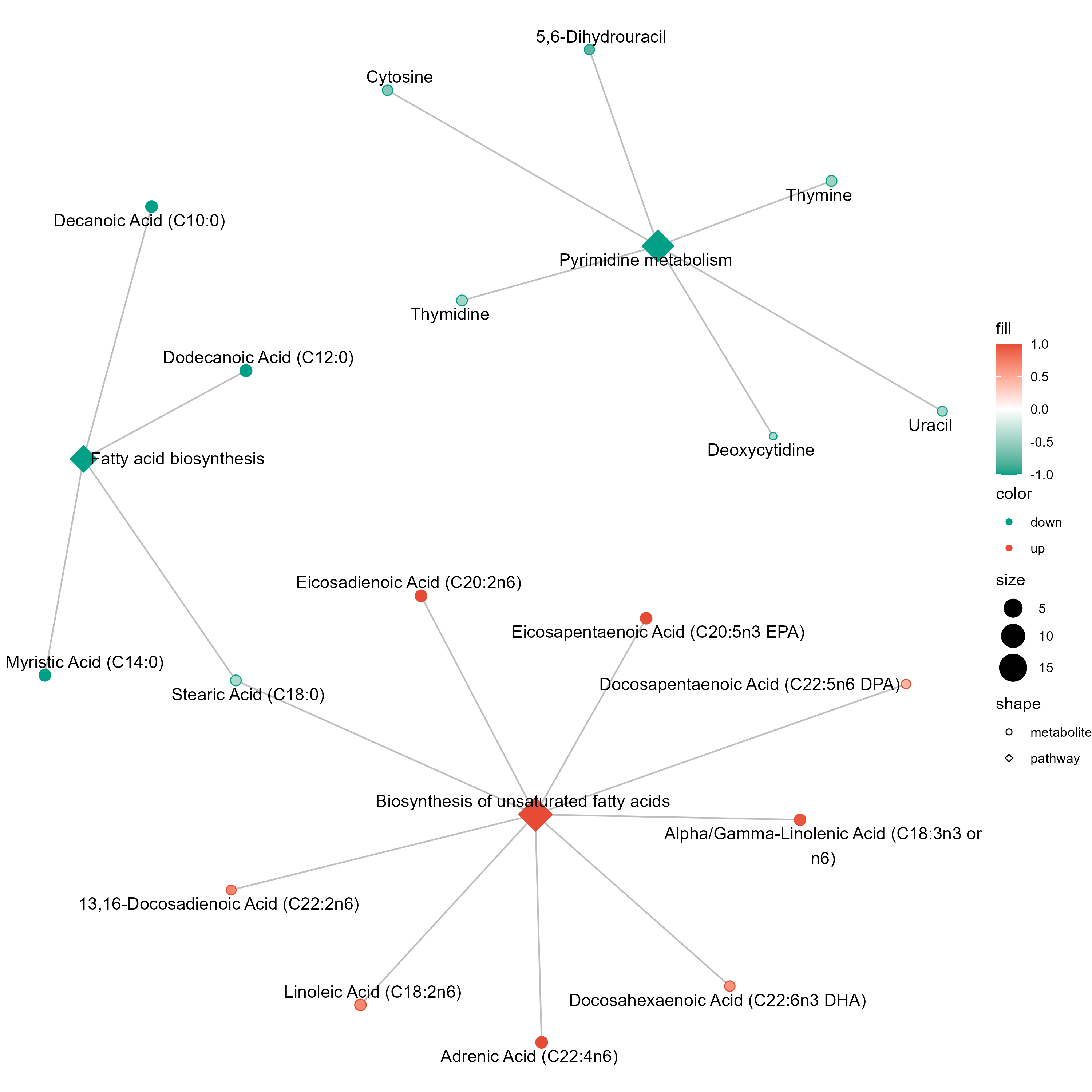

Supplement: Supplementary file 1 [file ijms-27-06236-s001.zip › Supplementary Materials/ijms-4276706_Metabolomics_Dataset/5-Pathway Network Analysis/Figure 5. Pathway network plot of Control-vs-Model.png]

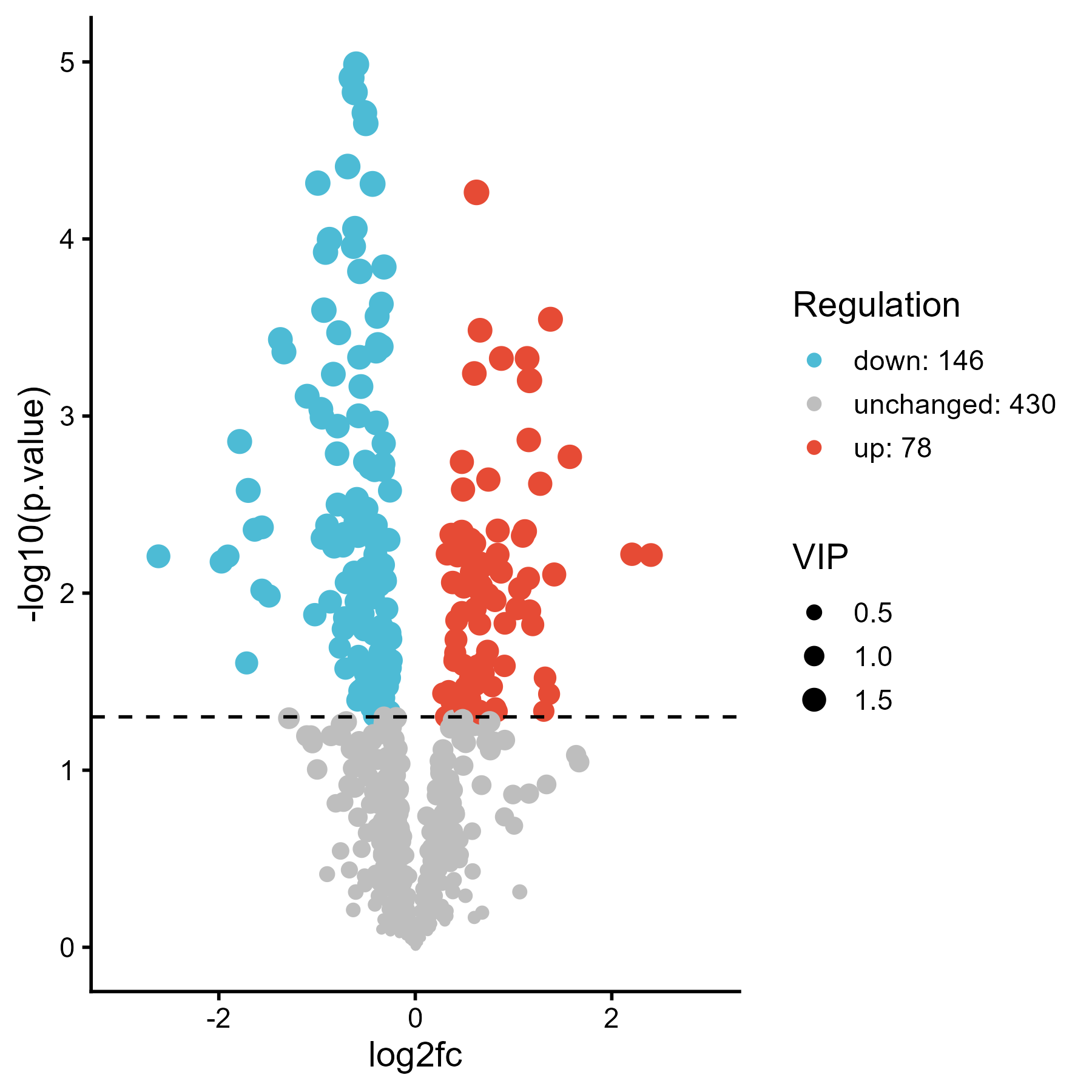

Supplement: Supplementary file 1 [file ijms-27-06236-s001.zip › Supplementary Materials/ijms-4276706_Metabolomics_Dataset/3-Differentially abundant metabolites/Figure 3b. Volcano plot of Model-vs-Paeoniflorin.png]

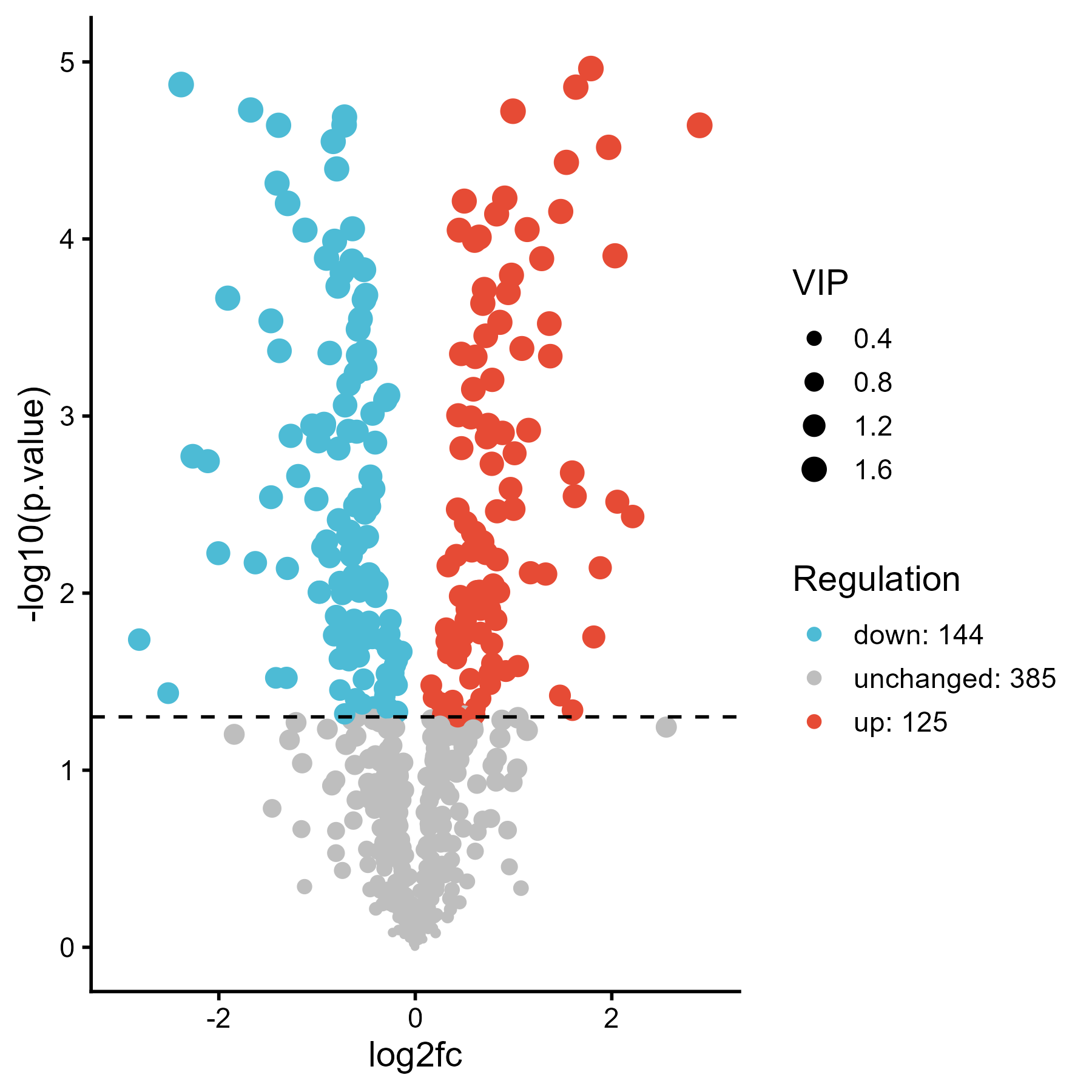

Supplement: Supplementary file 1 [file ijms-27-06236-s001.zip › Supplementary Materials/ijms-4276706_Metabolomics_Dataset/3-Differentially abundant metabolites/Figure 3b. Volcano plot of Control-vs-Model.png]

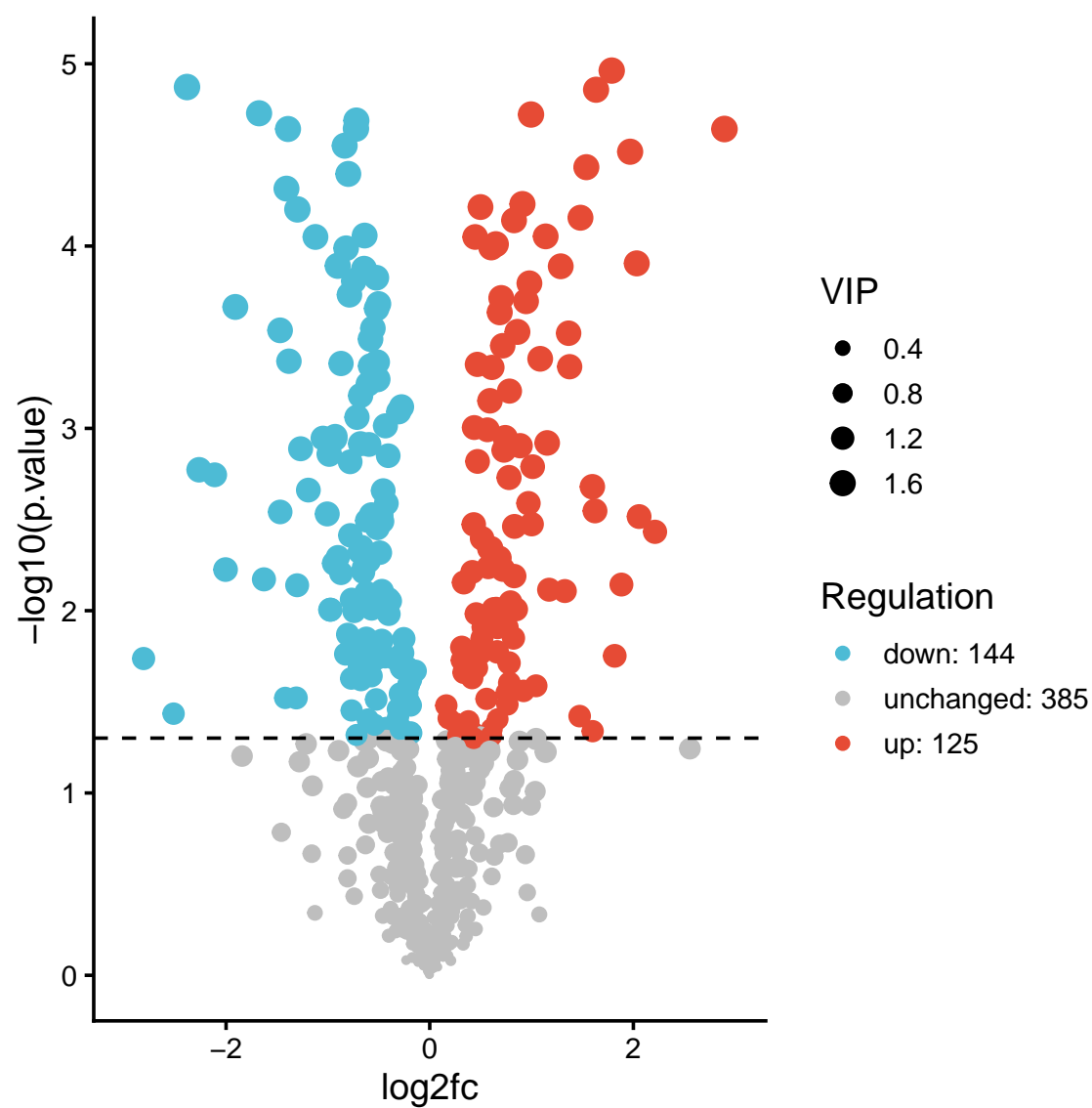

Supplement: Supplementary file 1 [file ijms-27-06236-s001.zip › Supplementary Materials/ijms-4276706_Metabolomics_Dataset/3-Differentially abundant metabolites/Figure 3b. Volcano plot of Control-vs-Model.pdf]

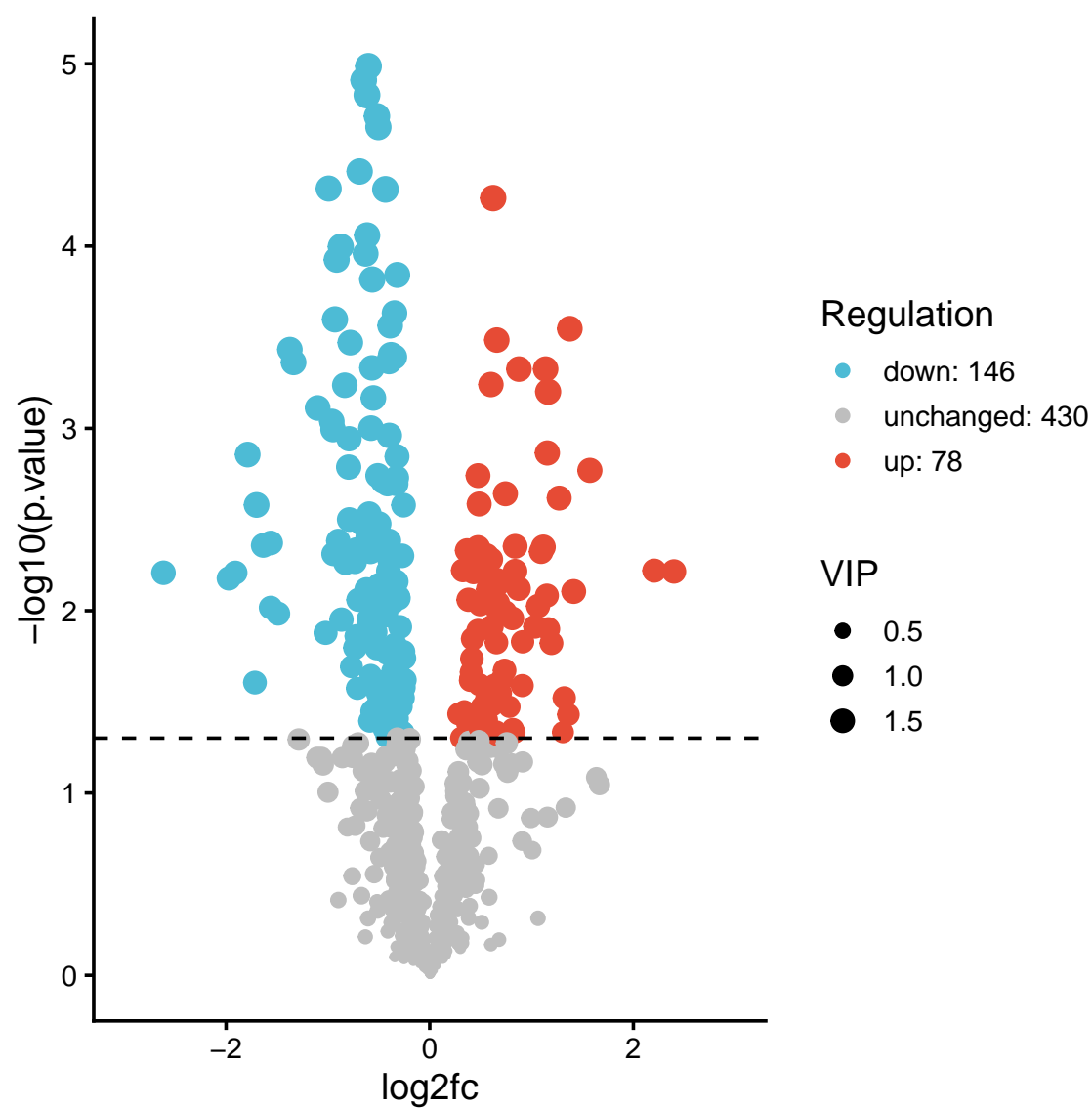

Supplement: Supplementary file 1 [file ijms-27-06236-s001.zip › Supplementary Materials/ijms-4276706_Metabolomics_Dataset/3-Differentially abundant metabolites/Figure 3b. Volcano plot of Model-vs-Paeoniflorin.pdf]

### Model Overview

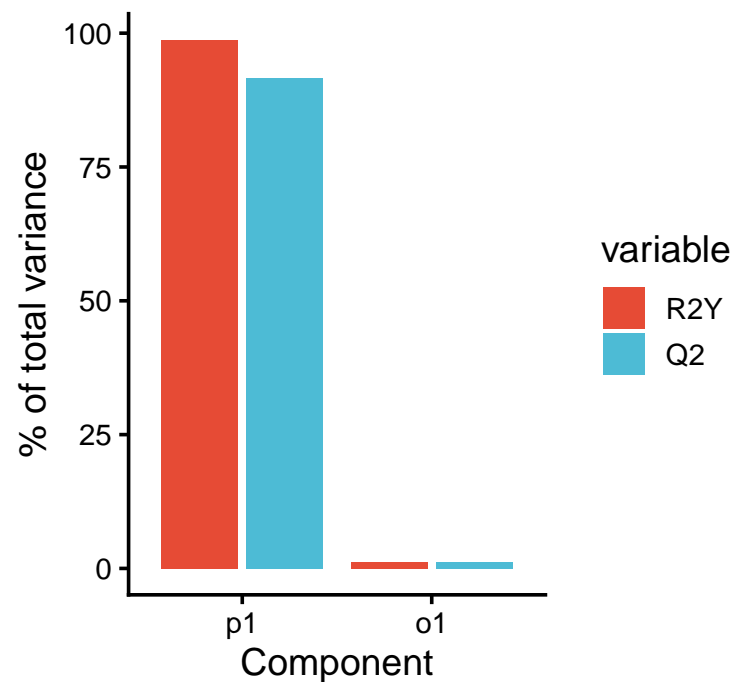

### Permutation testing

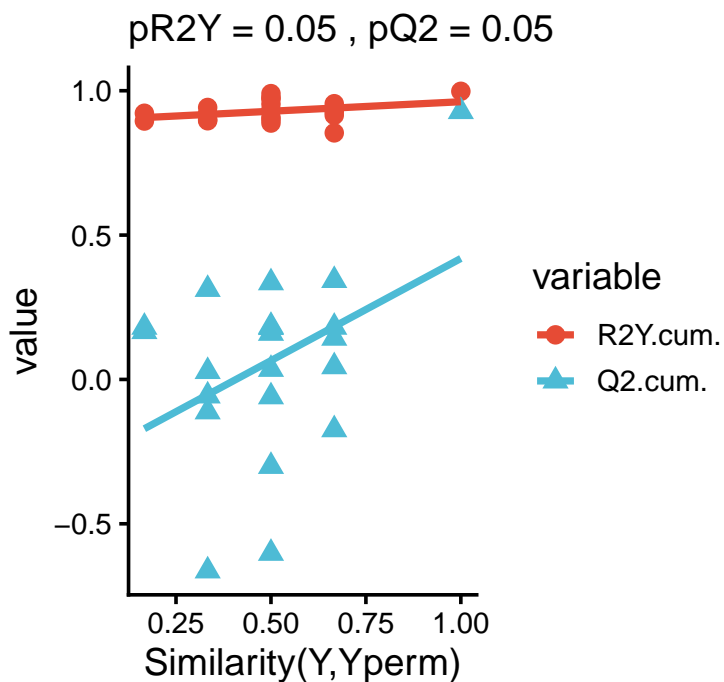

### Loading S-plot (OPLS-DA)

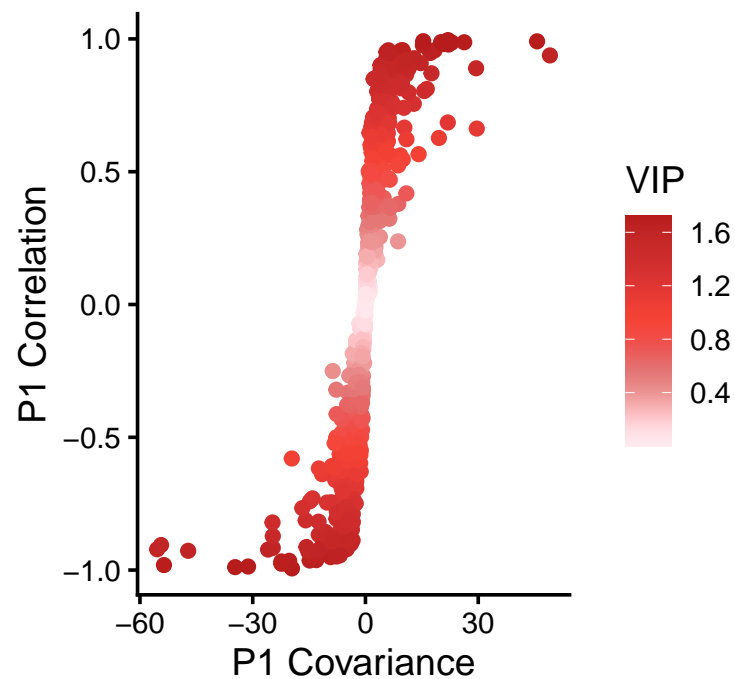

### Score (OPLS-DA) plot

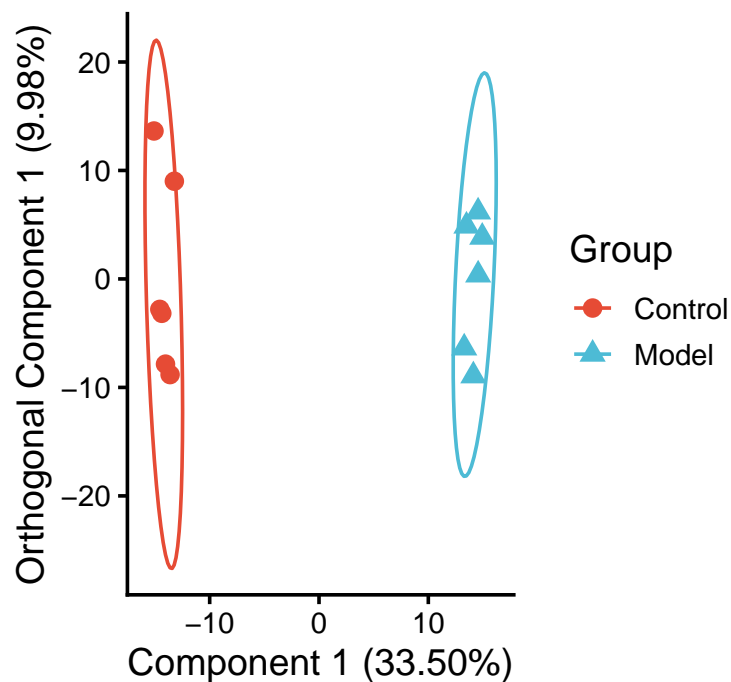

Supplement: Supplementary file 1 [file ijms-27-06236-s001.zip › Supplementary Materials/ijms-4276706_Metabolomics_Dataset/3-Differentially abundant metabolites/Figure 3a. OPLS-DA plot of Control-vs-Model.pdf]

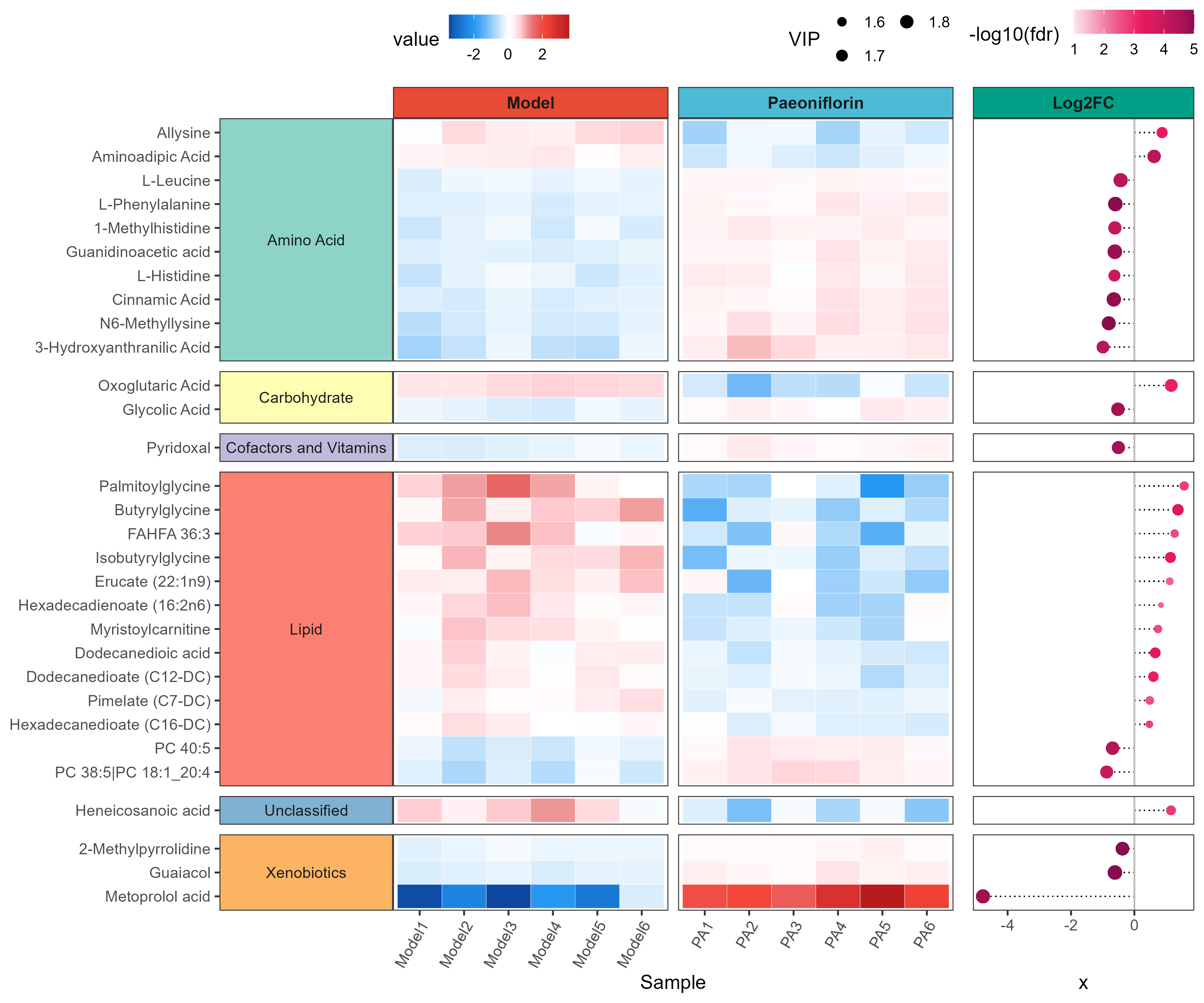

Supplement: Supplementary file 1 [file ijms-27-06236-s001.zip › Supplementary Materials/ijms-4276706_Metabolomics_Dataset/3-Differentially abundant metabolites/Figure 3c. Heatmap of Model-vs-Paeoniflorin.png]

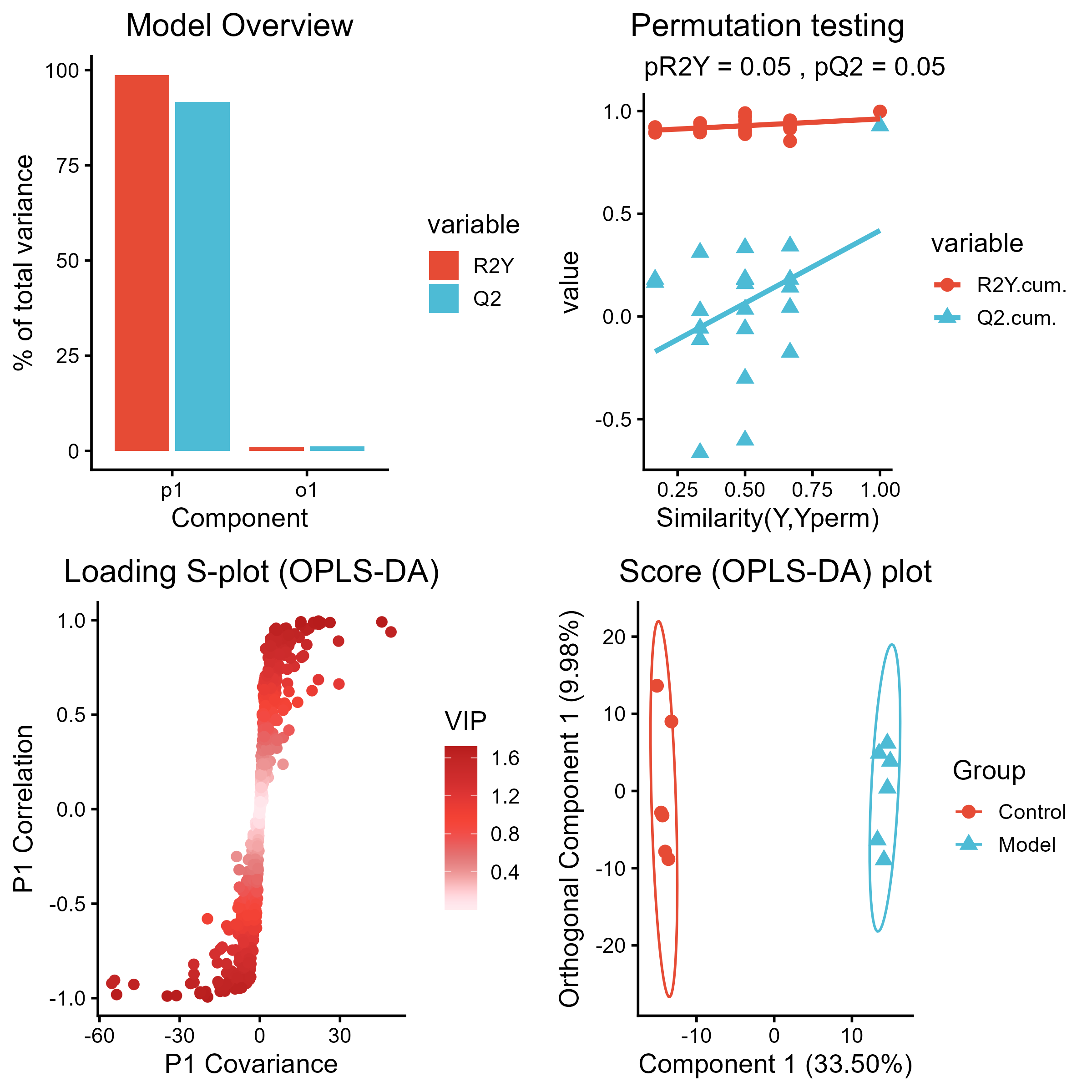

Supplement: Supplementary file 1 [file ijms-27-06236-s001.zip › Supplementary Materials/ijms-4276706_Metabolomics_Dataset/3-Differentially abundant metabolites/Figure 3a. OPLS-DA plot of Control-vs-Model.png]

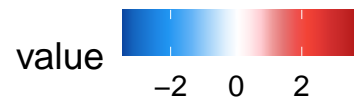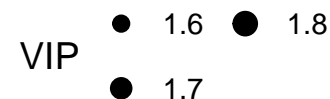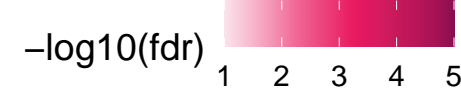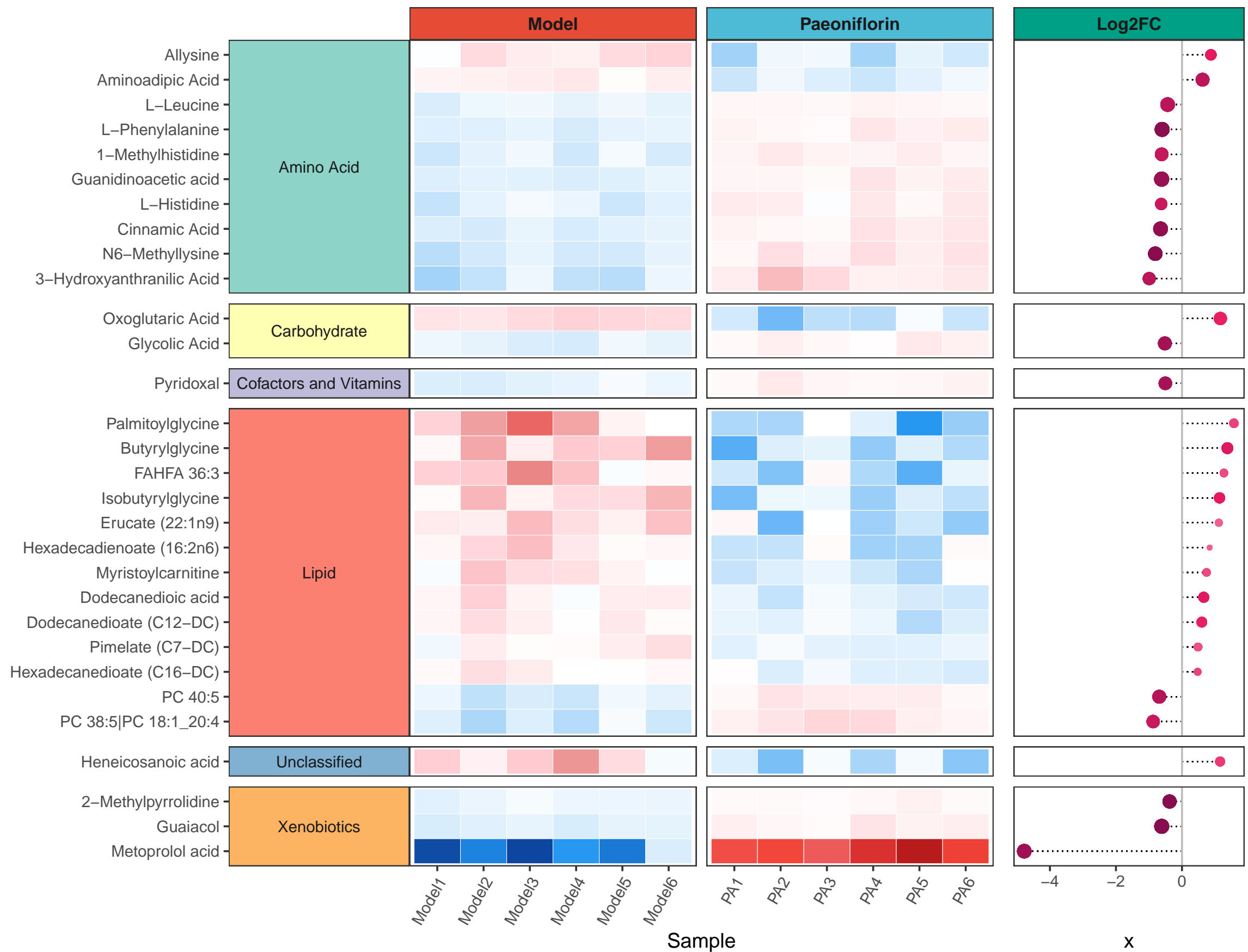

Supplement: Supplementary file 1 [file ijms-27-06236-s001.zip › Supplementary Materials/ijms-4276706_Metabolomics_Dataset/3-Differentially abundant metabolites/Figure 3c. Heatmap of Model-vs-Paeoniflorin.pdf]

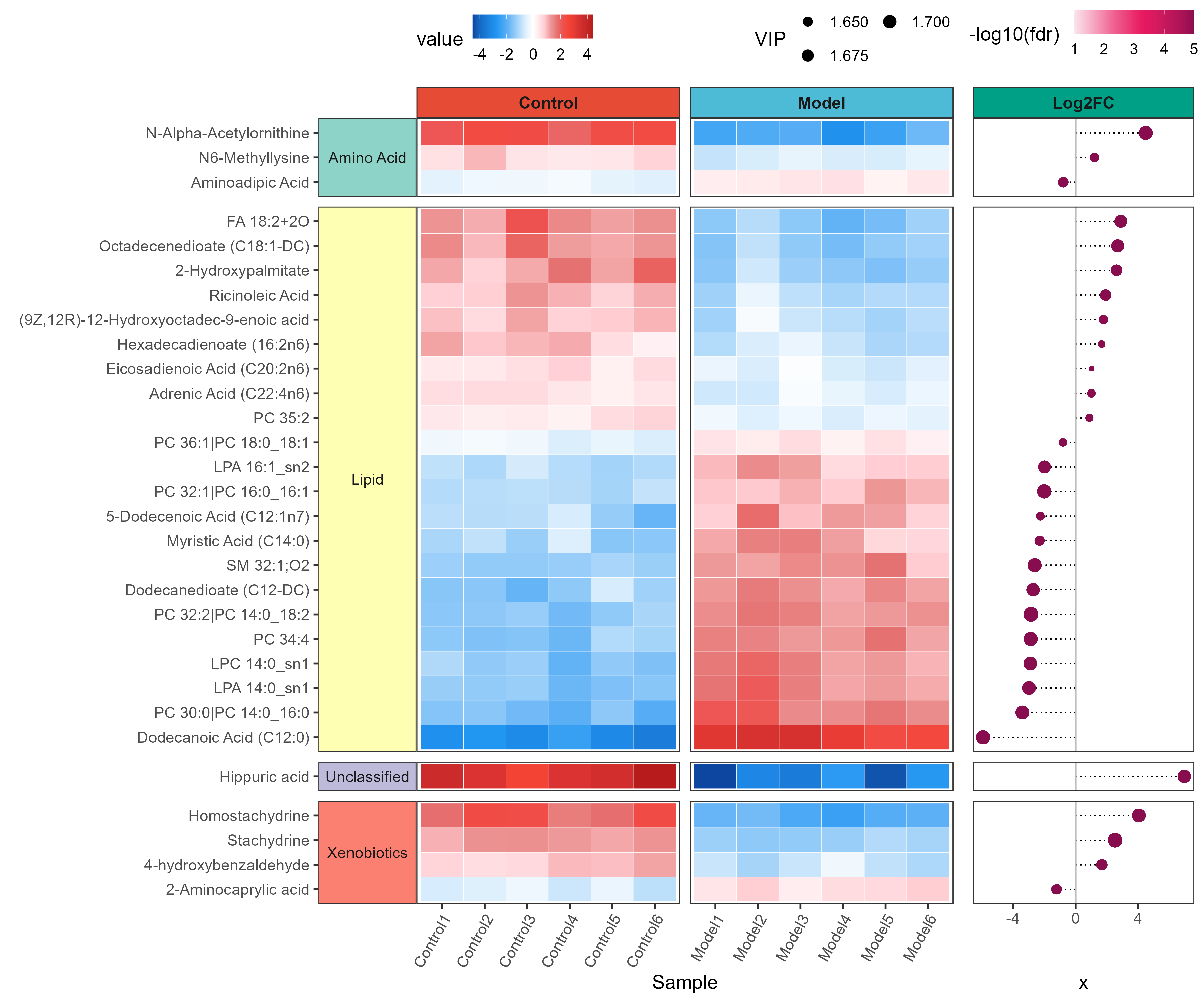

Supplement: Supplementary file 1 [file ijms-27-06236-s001.zip › Supplementary Materials/ijms-4276706_Metabolomics_Dataset/3-Differentially abundant metabolites/Figure 3c. Heatmap of Control-vs-Model.png]

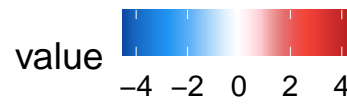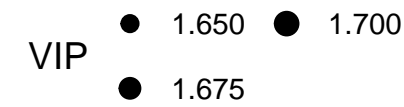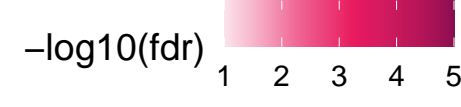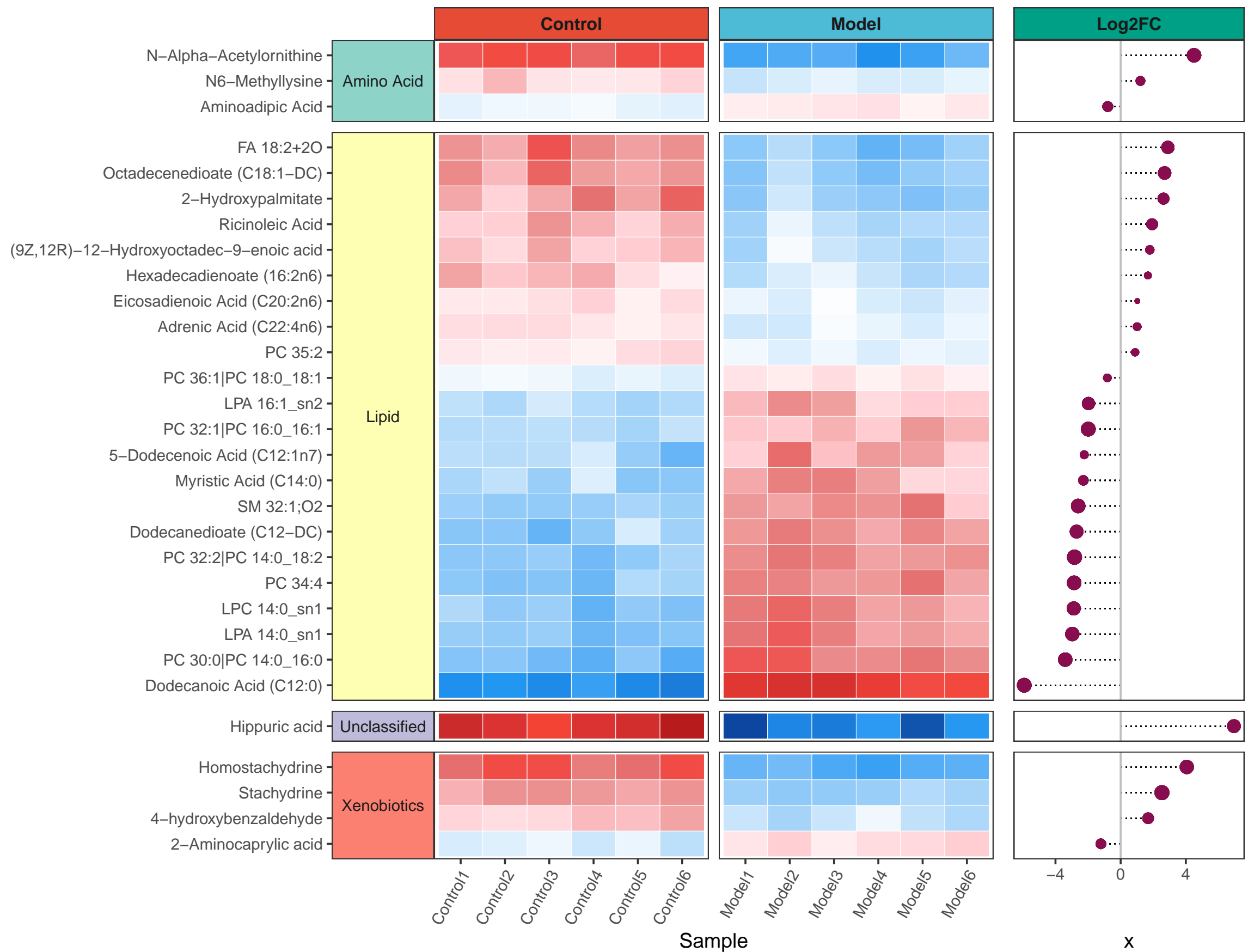

Supplement: Supplementary file 1 [file ijms-27-06236-s001.zip › Supplementary Materials/ijms-4276706_Metabolomics_Dataset/3-Differentially abundant metabolites/Figure 3c. Heatmap of Control-vs-Model.pdf]

### Model Overview

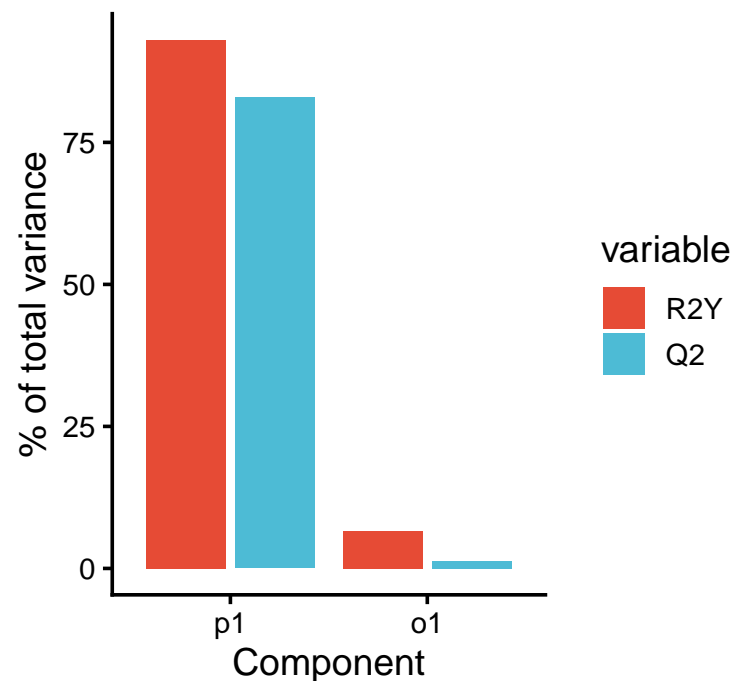

### Permutation testing

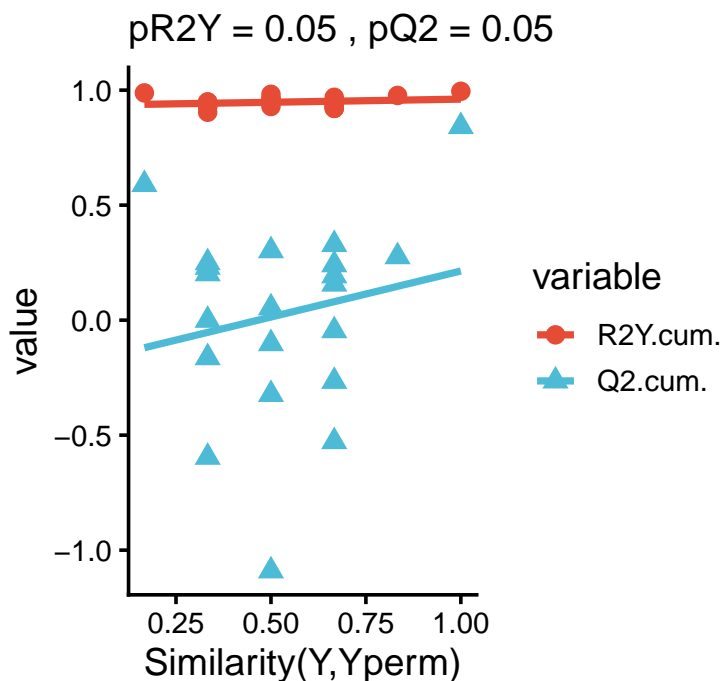

### Loading S-plot (OPLS-DA)

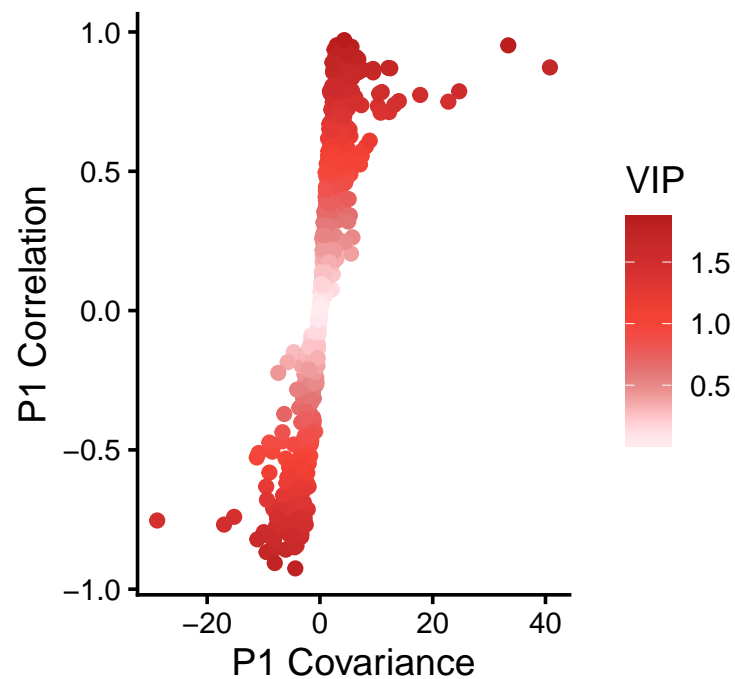

### Score (OPLS-DA) plot

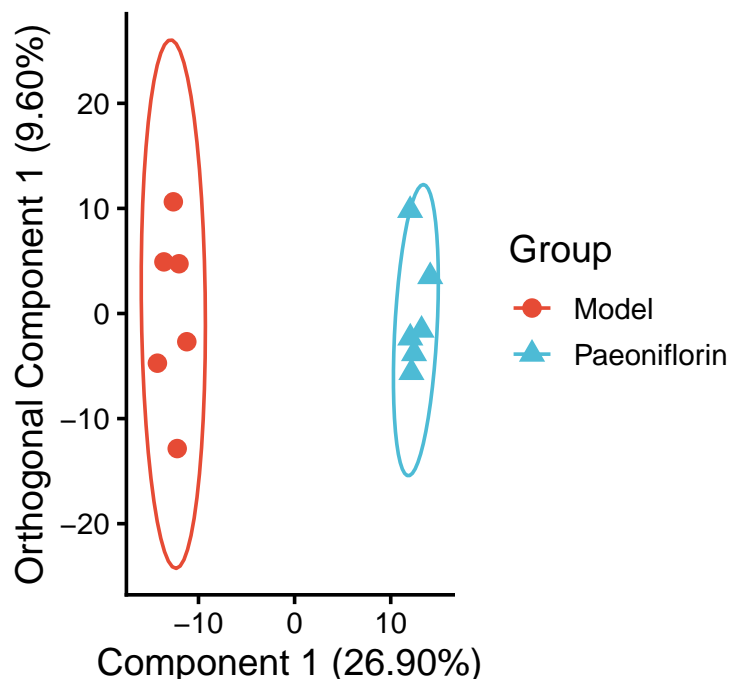

Supplement: Supplementary file 1 [file ijms-27-06236-s001.zip › Supplementary Materials/ijms-4276706_Metabolomics_Dataset/3-Differentially abundant metabolites/Figure 3a. OPLS-DA plot of Model-vs-Paeoniflorin.pdf]

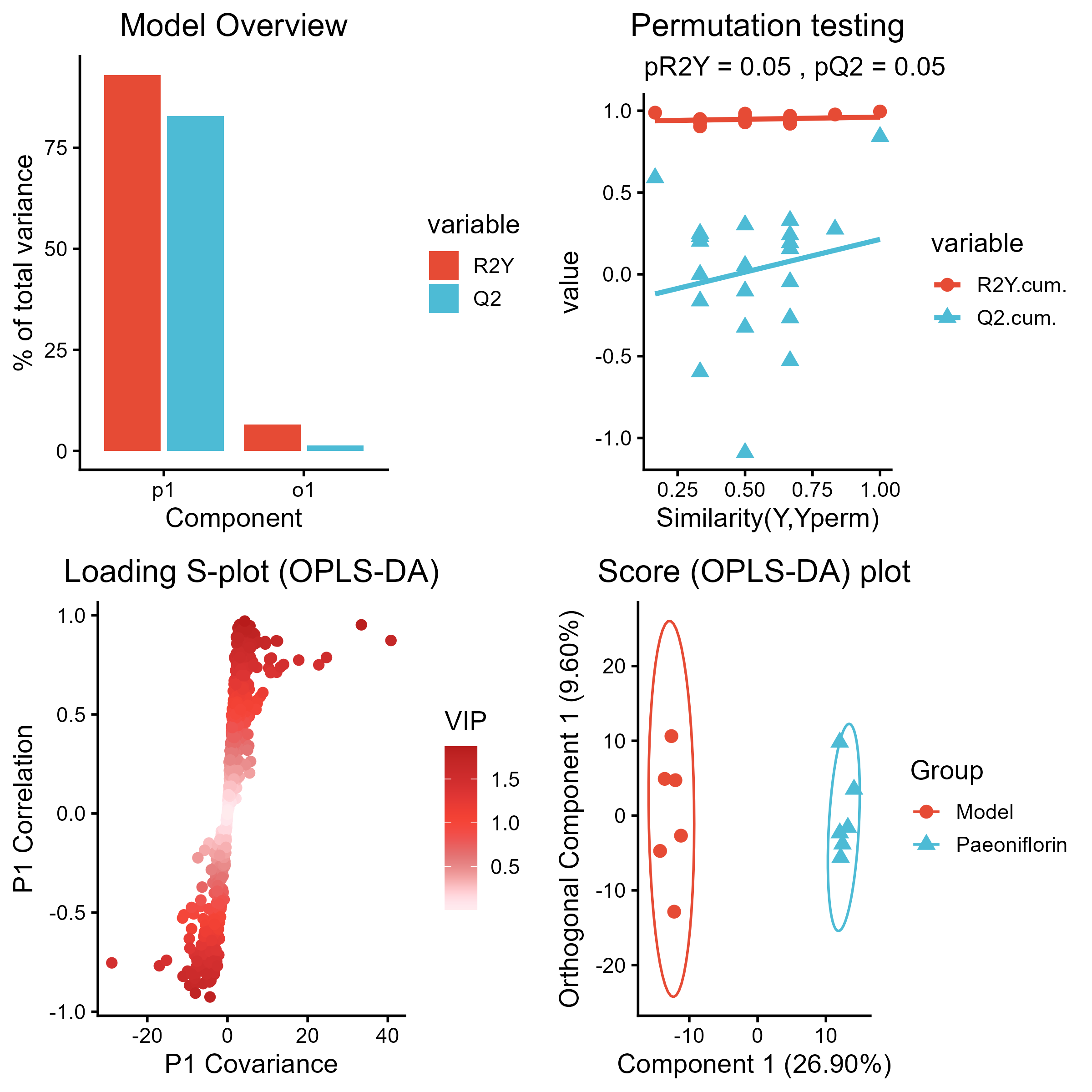

Supplement: Supplementary file 1 [file ijms-27-06236-s001.zip › Supplementary Materials/ijms-4276706_Metabolomics_Dataset/3-Differentially abundant metabolites/Figure 3a. OPLS-DA plot of Model-vs-Paeoniflorin.png]

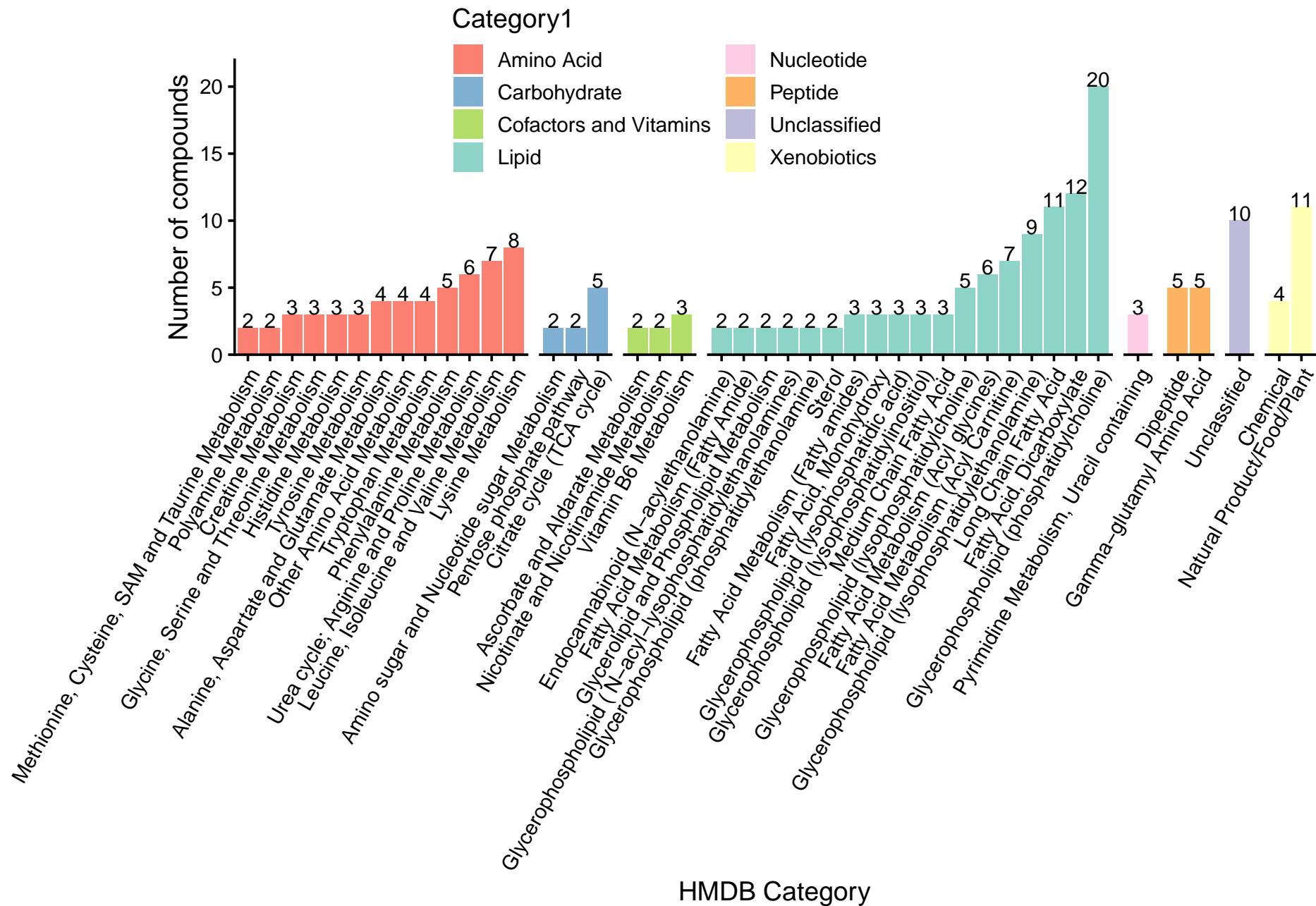

Supplement: Supplementary file 1 [file ijms-27-06236-s001.zip › Supplementary Materials/ijms-4276706_Metabolomics_Dataset/4-Functional Annotation and Enrichment/Figure 4a. Diff Metabolite Classification by Category of Model-vs-Paeoniflorin.pdf]

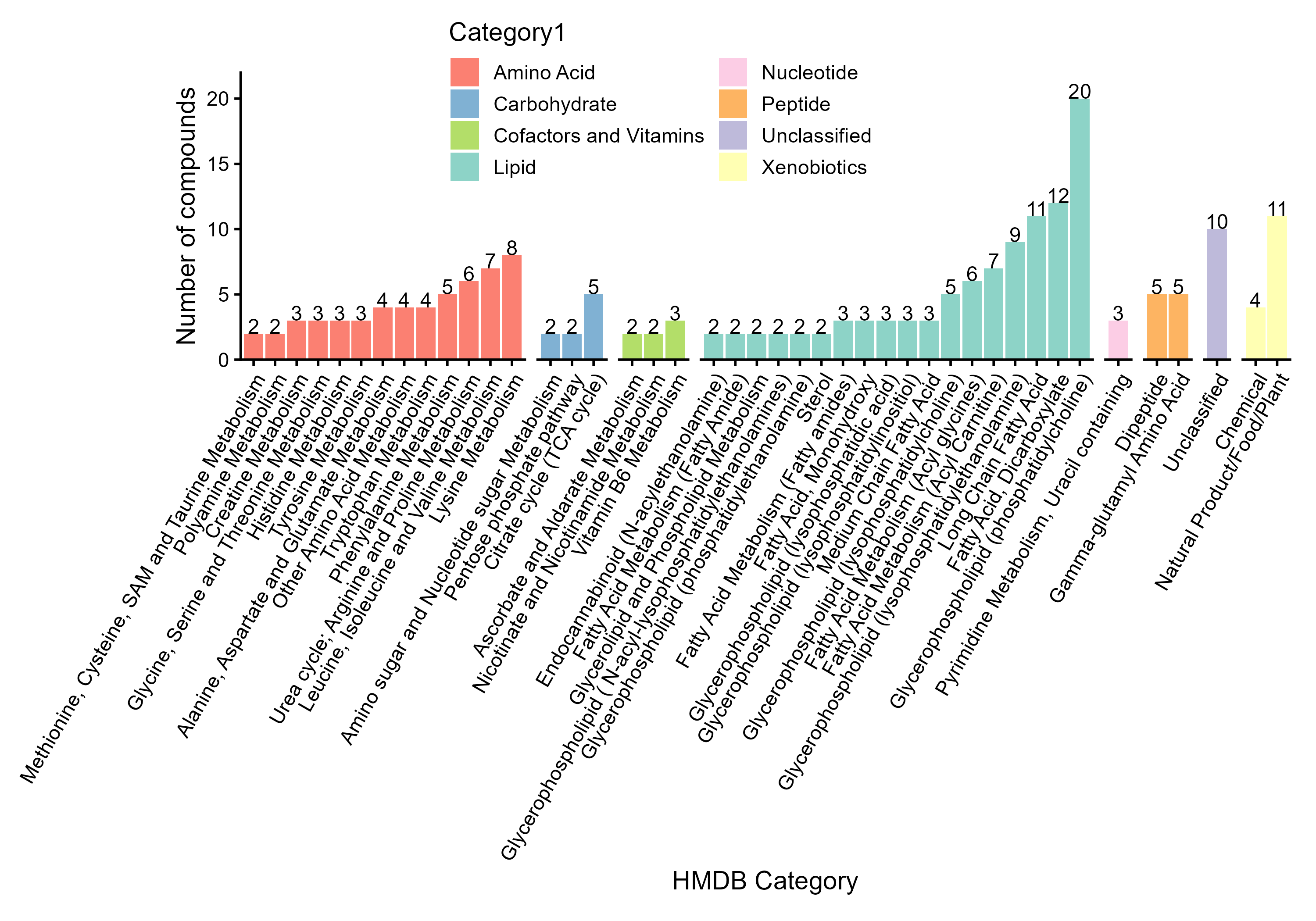

Supplement: Supplementary file 1 [file ijms-27-06236-s001.zip › Supplementary Materials/ijms-4276706_Metabolomics_Dataset/4-Functional Annotation and Enrichment/Figure 4a. Diff Metabolite Classification by Category of Model-vs-Paeoniflorin.png]

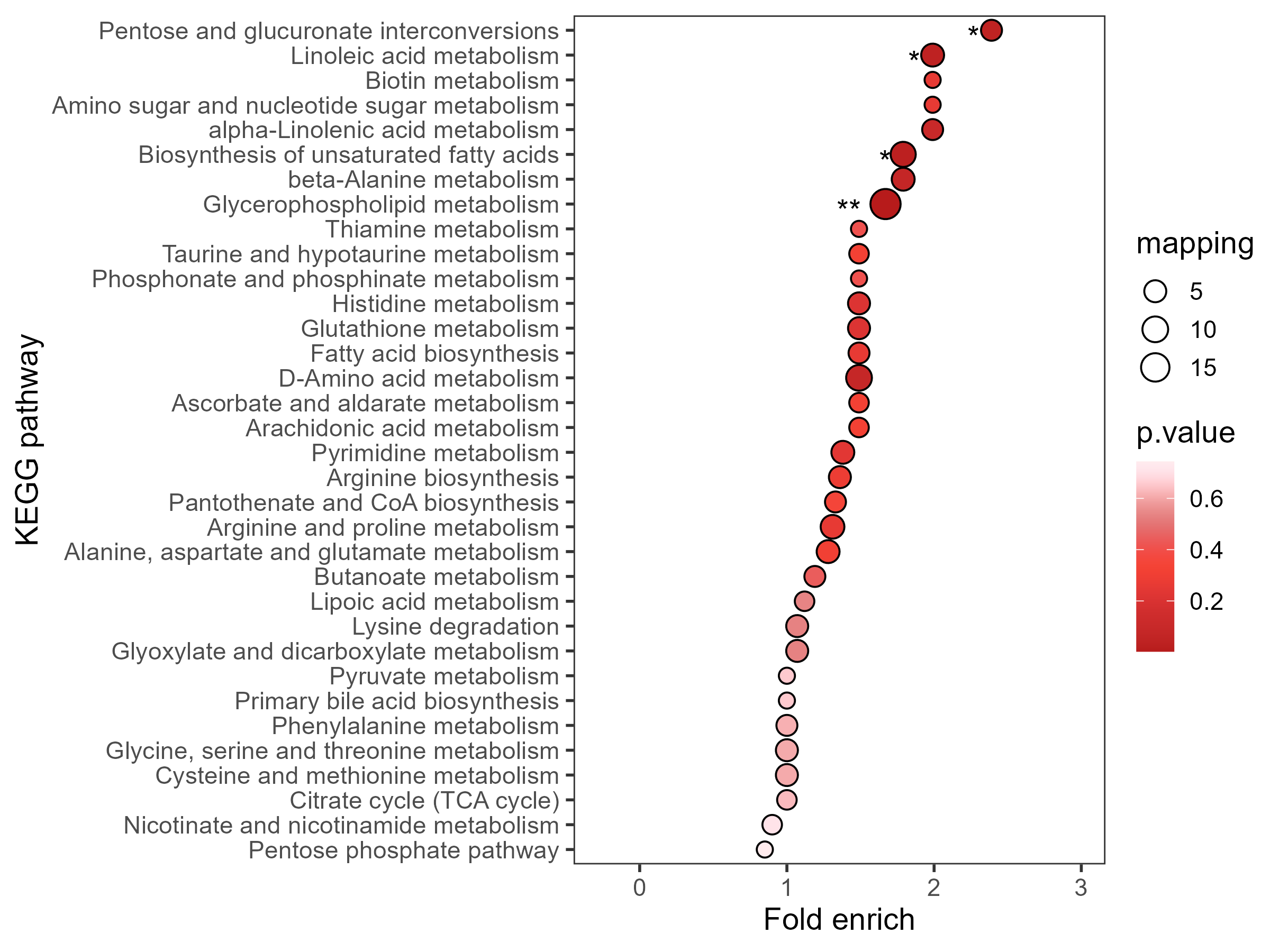

Supplement: Supplementary file 1 [file ijms-27-06236-s001.zip › Supplementary Materials/ijms-4276706_Metabolomics_Dataset/4-Functional Annotation and Enrichment/Figure 4c. KEGG enrichment of Control-vs-Model.png]

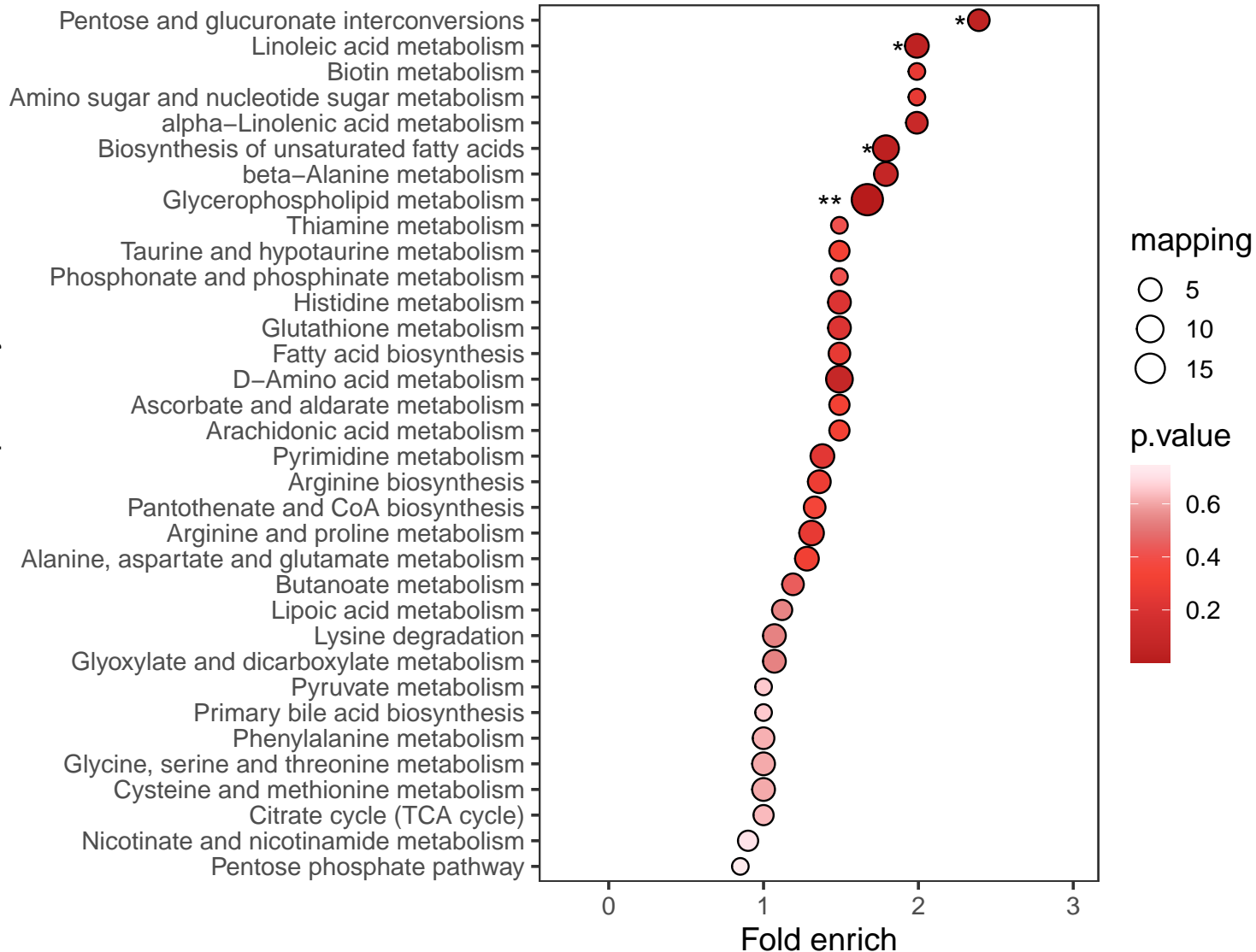

Supplement: Supplementary file 1 [file ijms-27-06236-s001.zip › Supplementary Materials/ijms-4276706_Metabolomics_Dataset/4-Functional Annotation and Enrichment/Figure 4c. KEGG enrichment of Control-vs-Model.pdf]

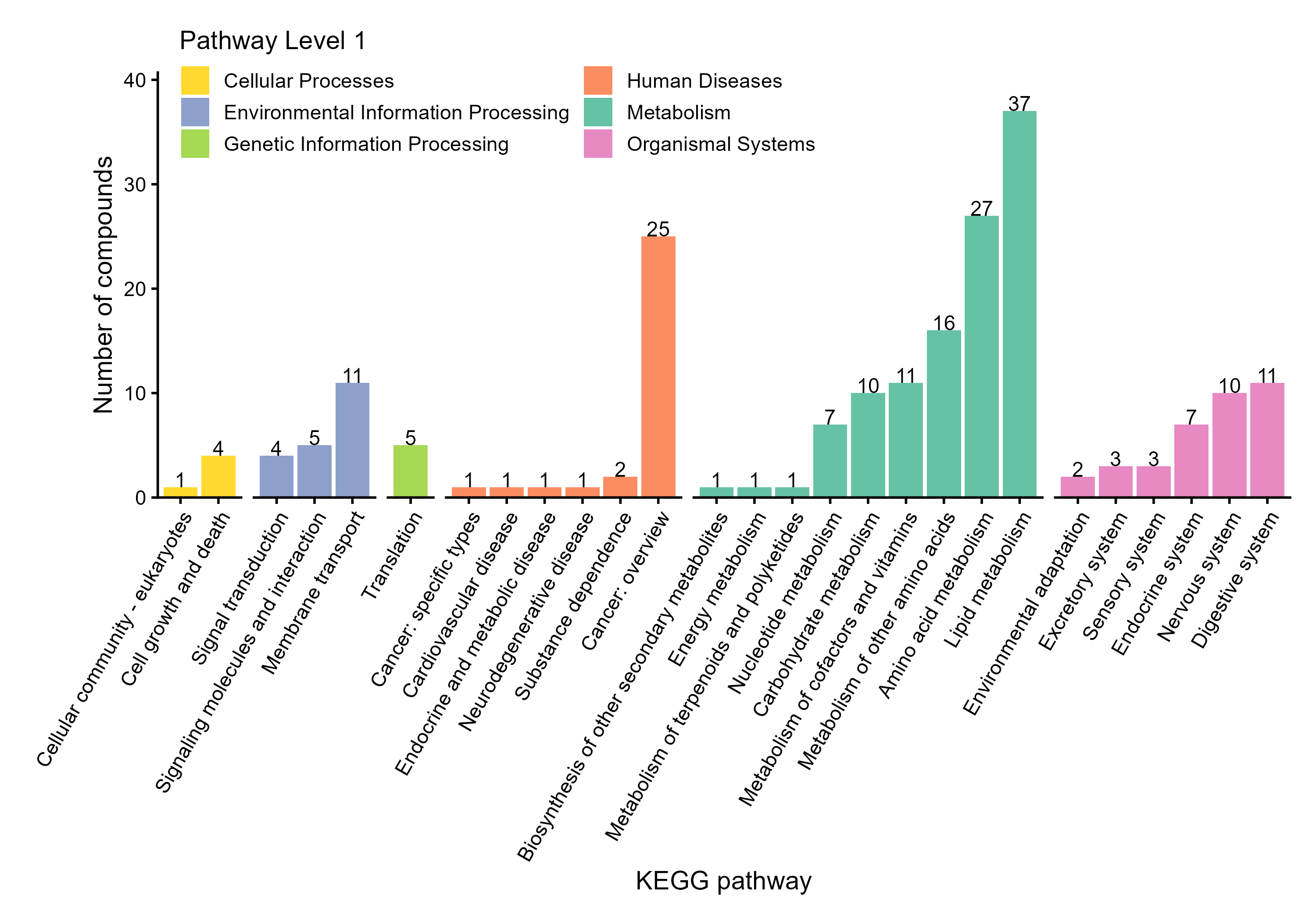

Supplement: Supplementary file 1 [file ijms-27-06236-s001.zip › Supplementary Materials/ijms-4276706_Metabolomics_Dataset/4-Functional Annotation and Enrichment/Figure 4b. Diff Metabolite Classification by Pathway of Control-vs-Model.png]

# Pathway Level 1

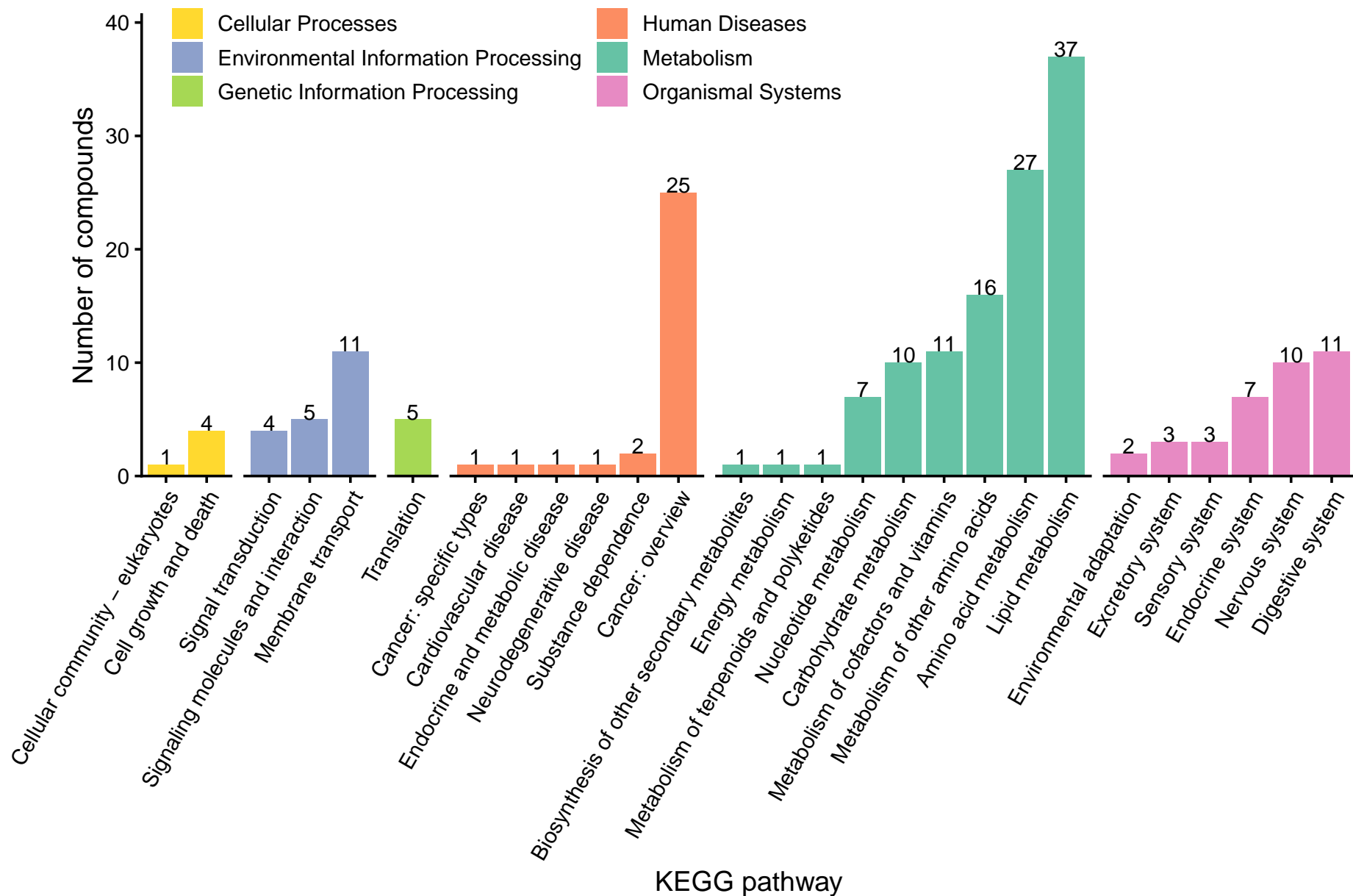

Supplement: Supplementary file 1 [file ijms-27-06236-s001.zip › Supplementary Materials/ijms-4276706_Metabolomics_Dataset/4-Functional Annotation and Enrichment/Figure 4b. Diff Metabolite Classification by Pathway of Control-vs-Model.pdf]

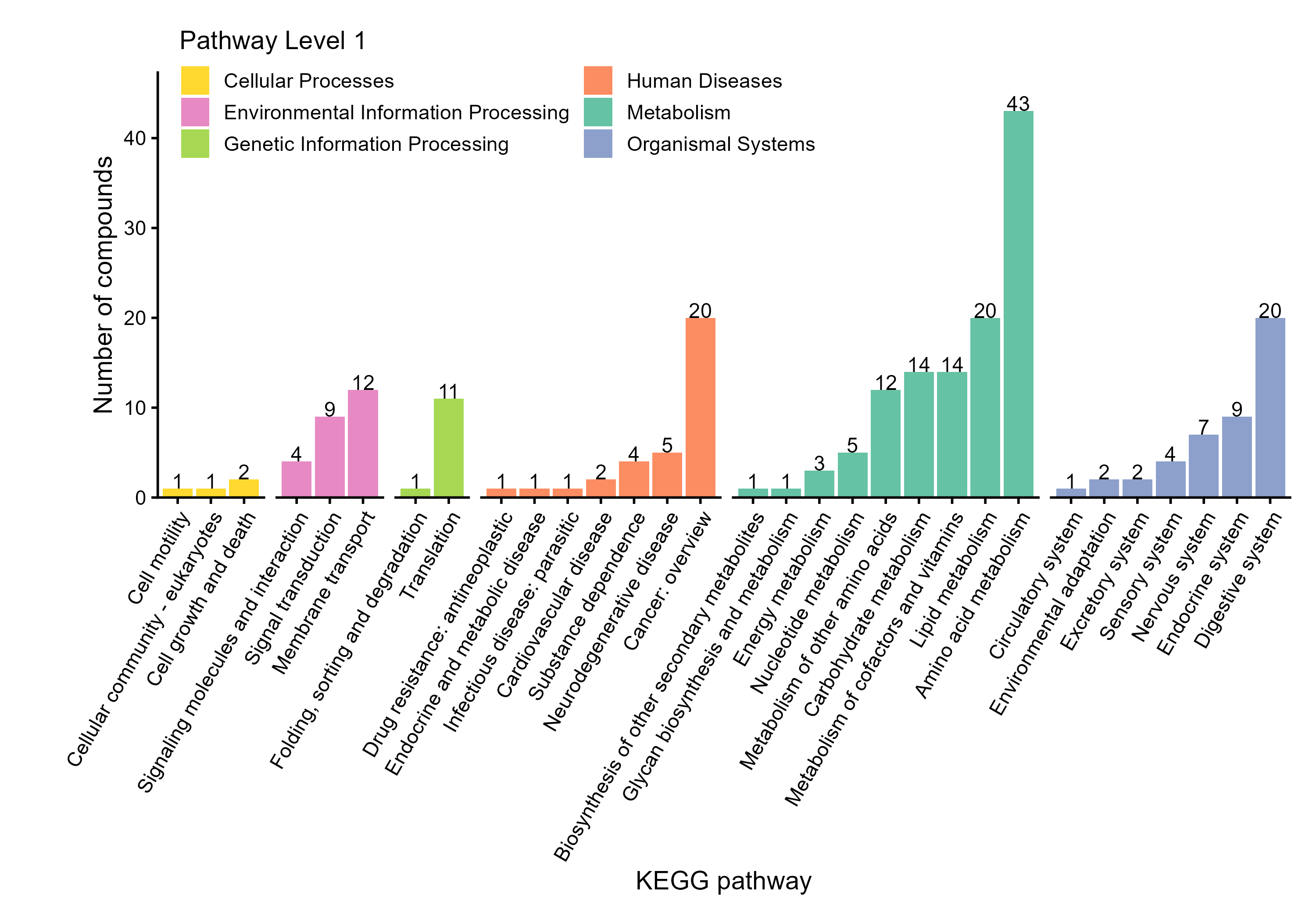

Supplement: Supplementary file 1 [file ijms-27-06236-s001.zip › Supplementary Materials/ijms-4276706_Metabolomics_Dataset/4-Functional Annotation and Enrichment/Figure 4b. Diff Metabolite Classification by Pathway of Model-vs-Paeoniflorin.png]

# Pathway Level 1

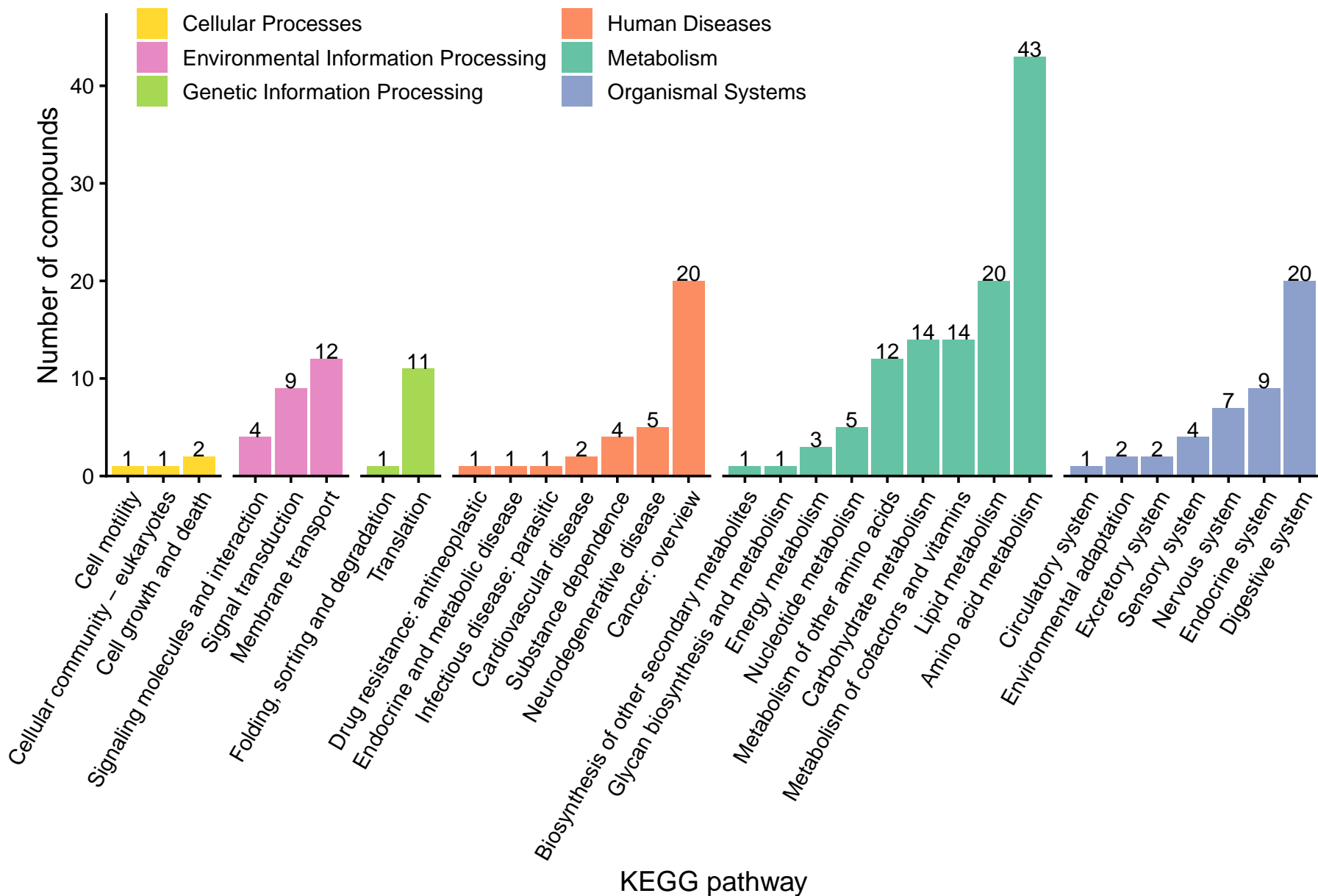

Supplement: Supplementary file 1 [file ijms-27-06236-s001.zip › Supplementary Materials/ijms-4276706_Metabolomics_Dataset/4-Functional Annotation and Enrichment/Figure 4b. Diff Metabolite Classification by Pathway of Model-vs-Paeoniflorin.pdf]

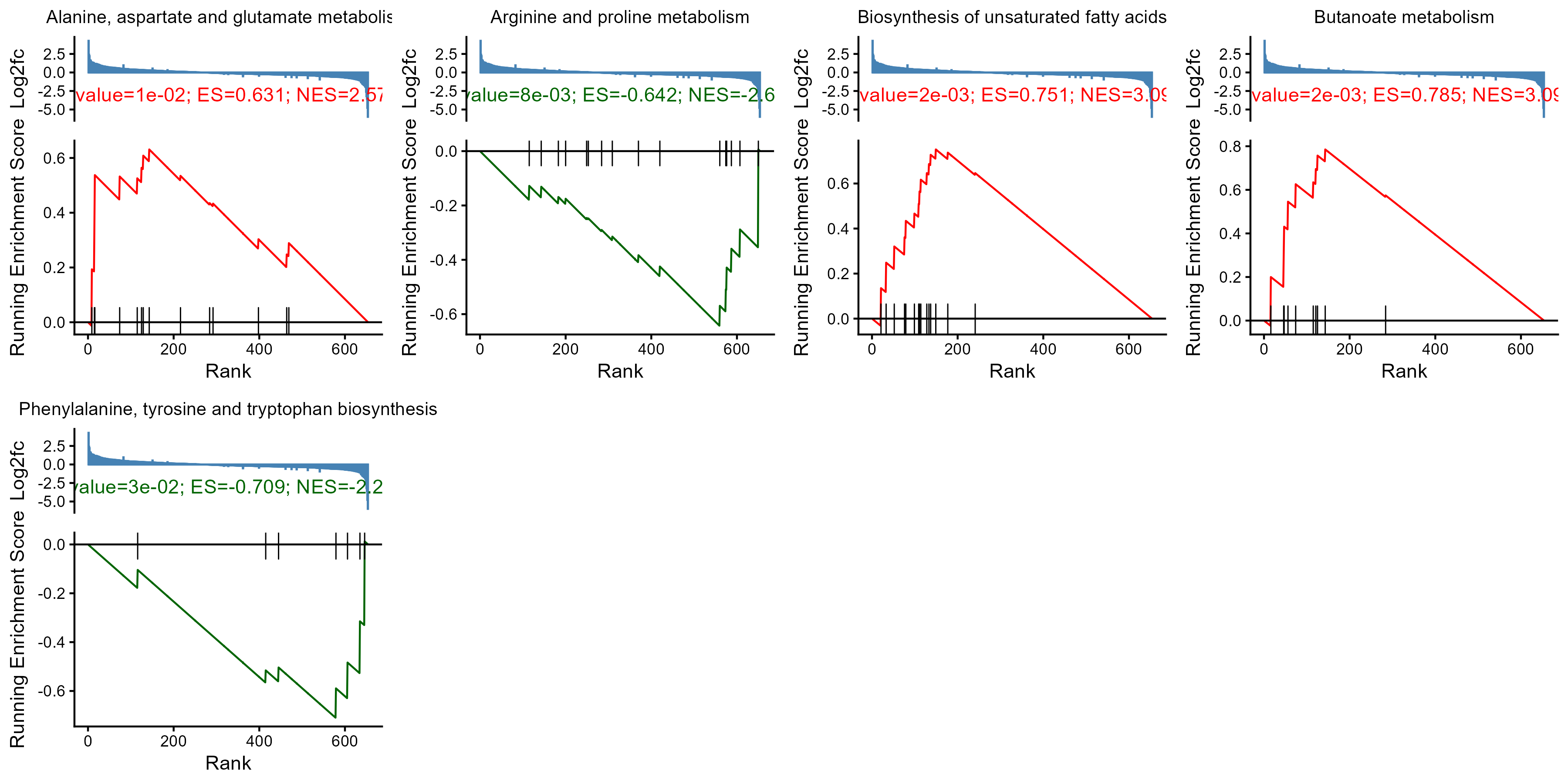

Supplement: Supplementary file 1 [file ijms-27-06236-s001.zip › Supplementary Materials/ijms-4276706_Metabolomics_Dataset/4-Functional Annotation and Enrichment/Figure 4e. MSEA enrichment2 of Model-vs-Paeoniflorin.png]

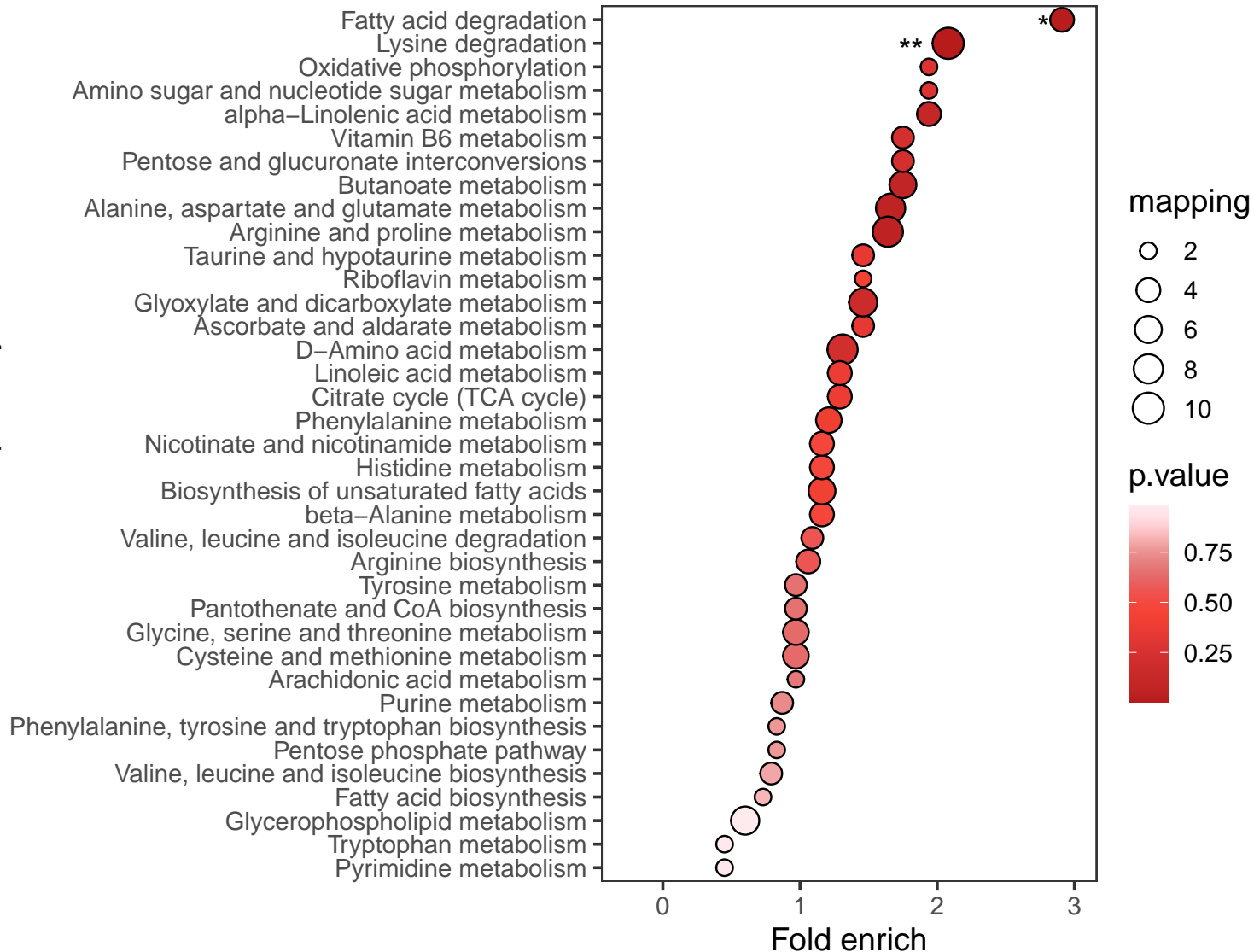

Supplement: Supplementary file 1 [file ijms-27-06236-s001.zip › Supplementary Materials/ijms-4276706_Metabolomics_Dataset/4-Functional Annotation and Enrichment/Figure 4c. KEGG enrichment of Model-vs-Paeoniflorin.pdf]

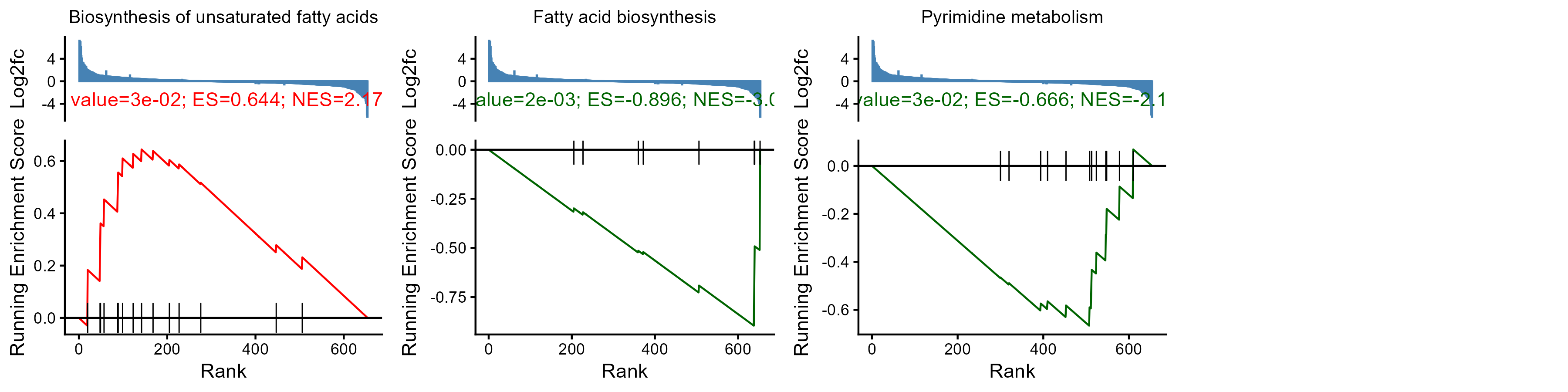

Supplement: Supplementary file 1 [file ijms-27-06236-s001.zip › Supplementary Materials/ijms-4276706_Metabolomics_Dataset/4-Functional Annotation and Enrichment/Figure 4e. MSEA enrichment2 of Control-vs-Model.png]

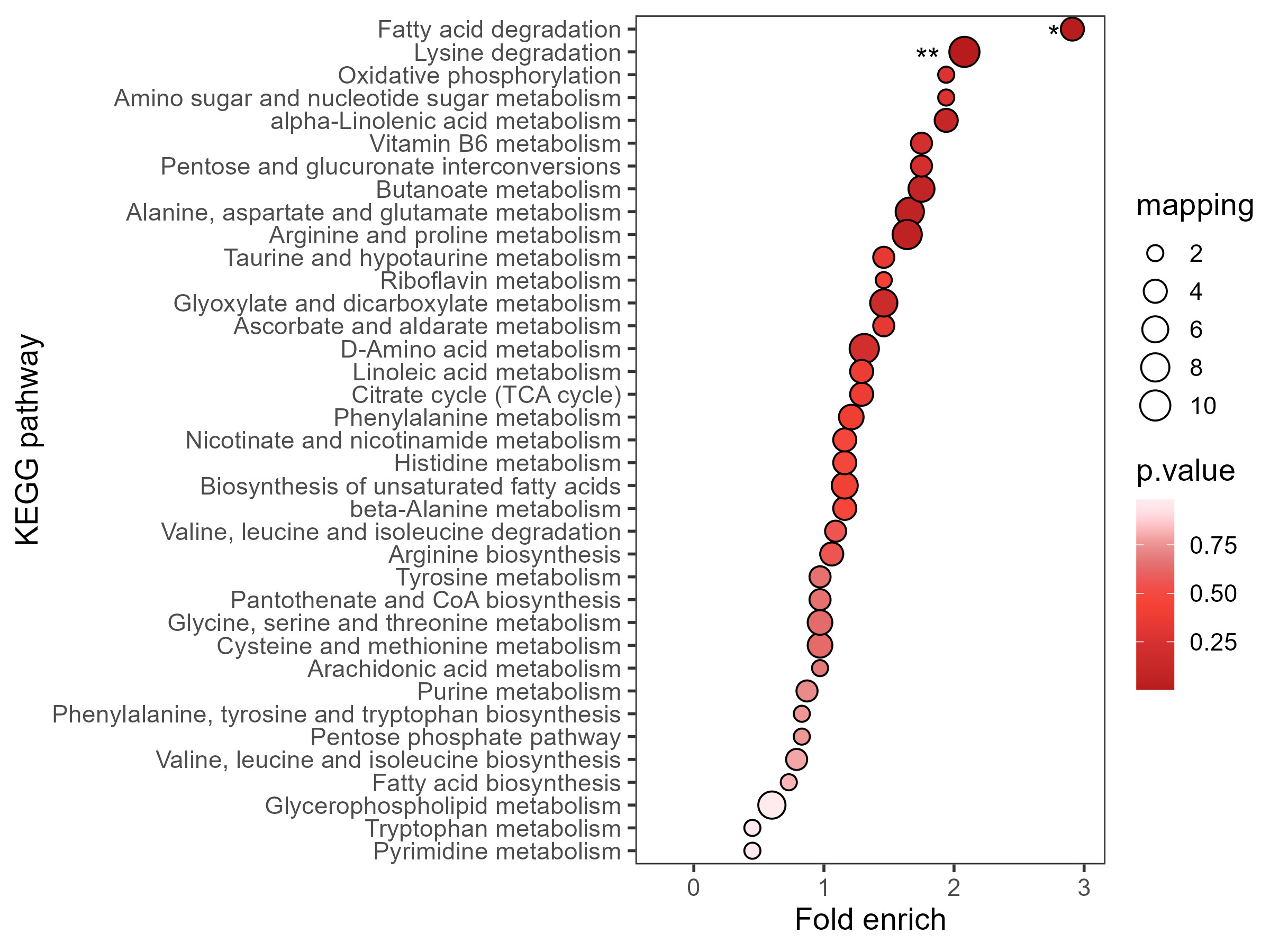

Supplement: Supplementary file 1 [file ijms-27-06236-s001.zip › Supplementary Materials/ijms-4276706_Metabolomics_Dataset/4-Functional Annotation and Enrichment/Figure 4c. KEGG enrichment of Model-vs-Paeoniflorin.png]

Biosynthesis of unsaturated fatty acids

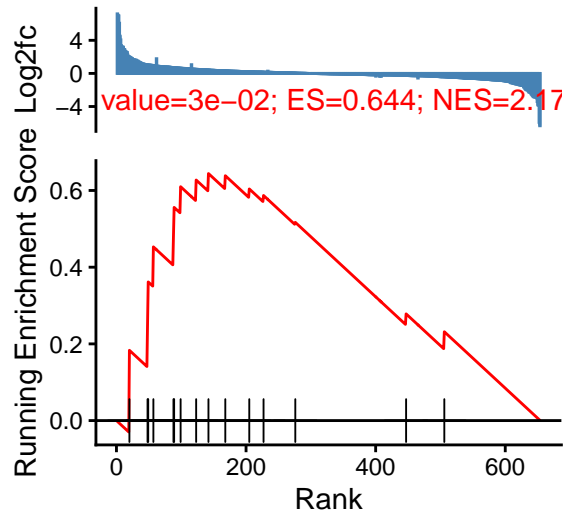

Fatty acid biosynthesis

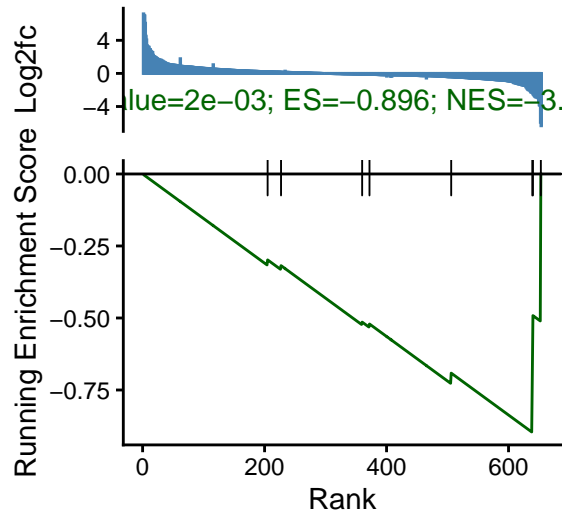

Pyrimidine metabolism

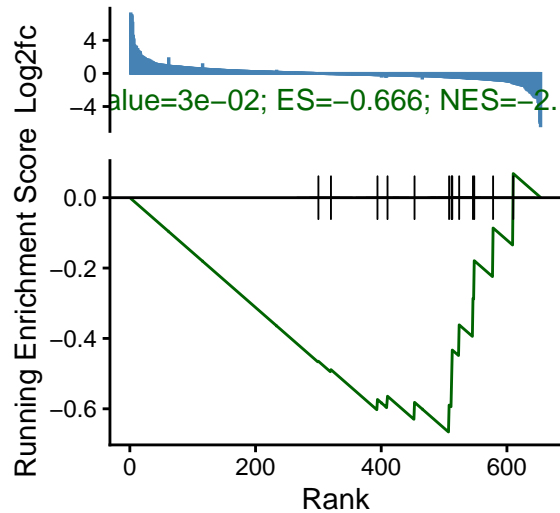

Supplement: Supplementary file 1 [file ijms-27-06236-s001.zip › Supplementary Materials/ijms-4276706_Metabolomics_Dataset/4-Functional Annotation and Enrichment/Figure 4e. MSEA enrichment2 of Control-vs-Model.pdf]

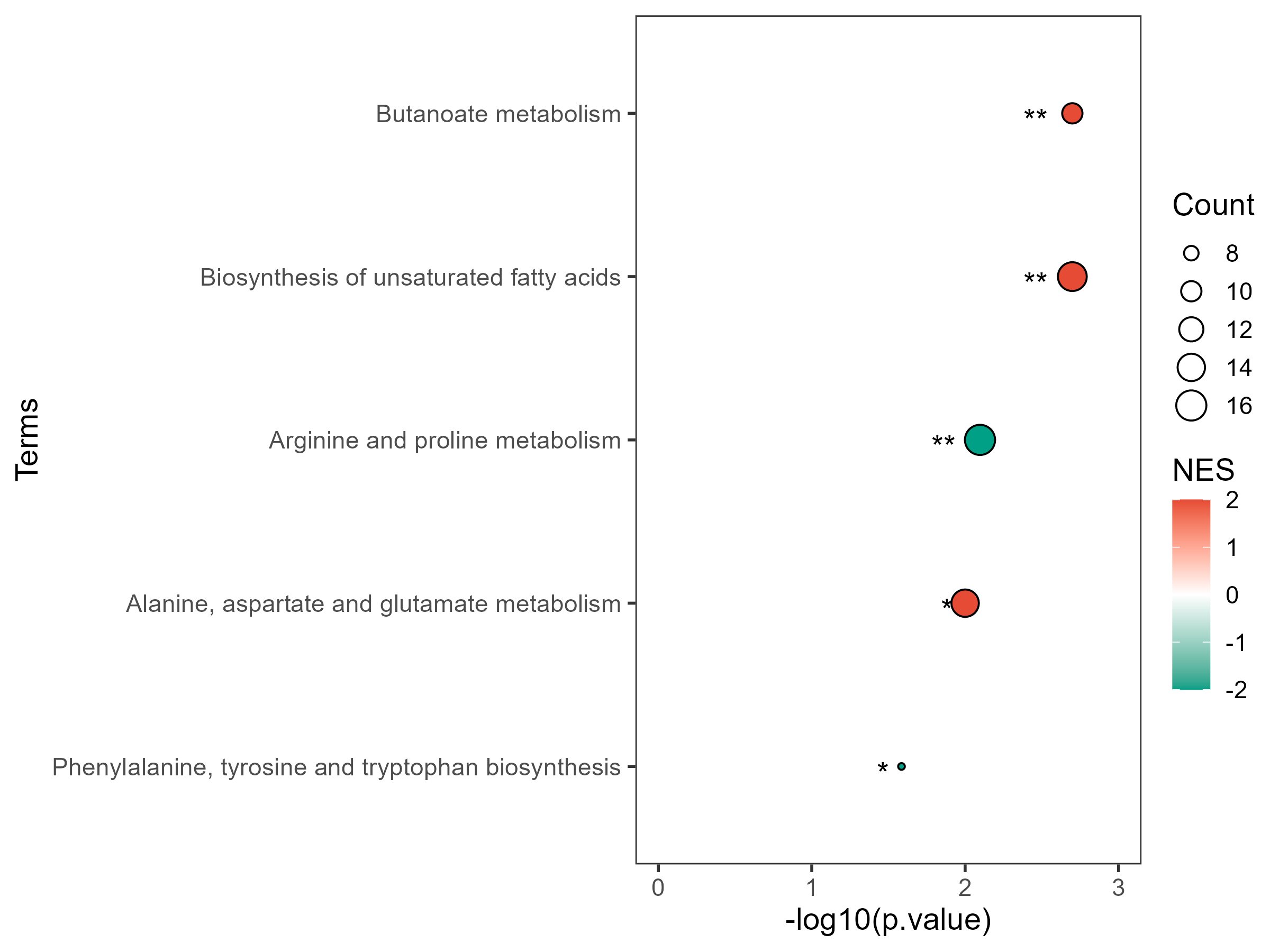

Supplement: Supplementary file 1 [file ijms-27-06236-s001.zip › Supplementary Materials/ijms-4276706_Metabolomics_Dataset/4-Functional Annotation and Enrichment/Figure 4d. MSEA enrichment1 of Model-vs-Paeoniflorin.png]

Terms

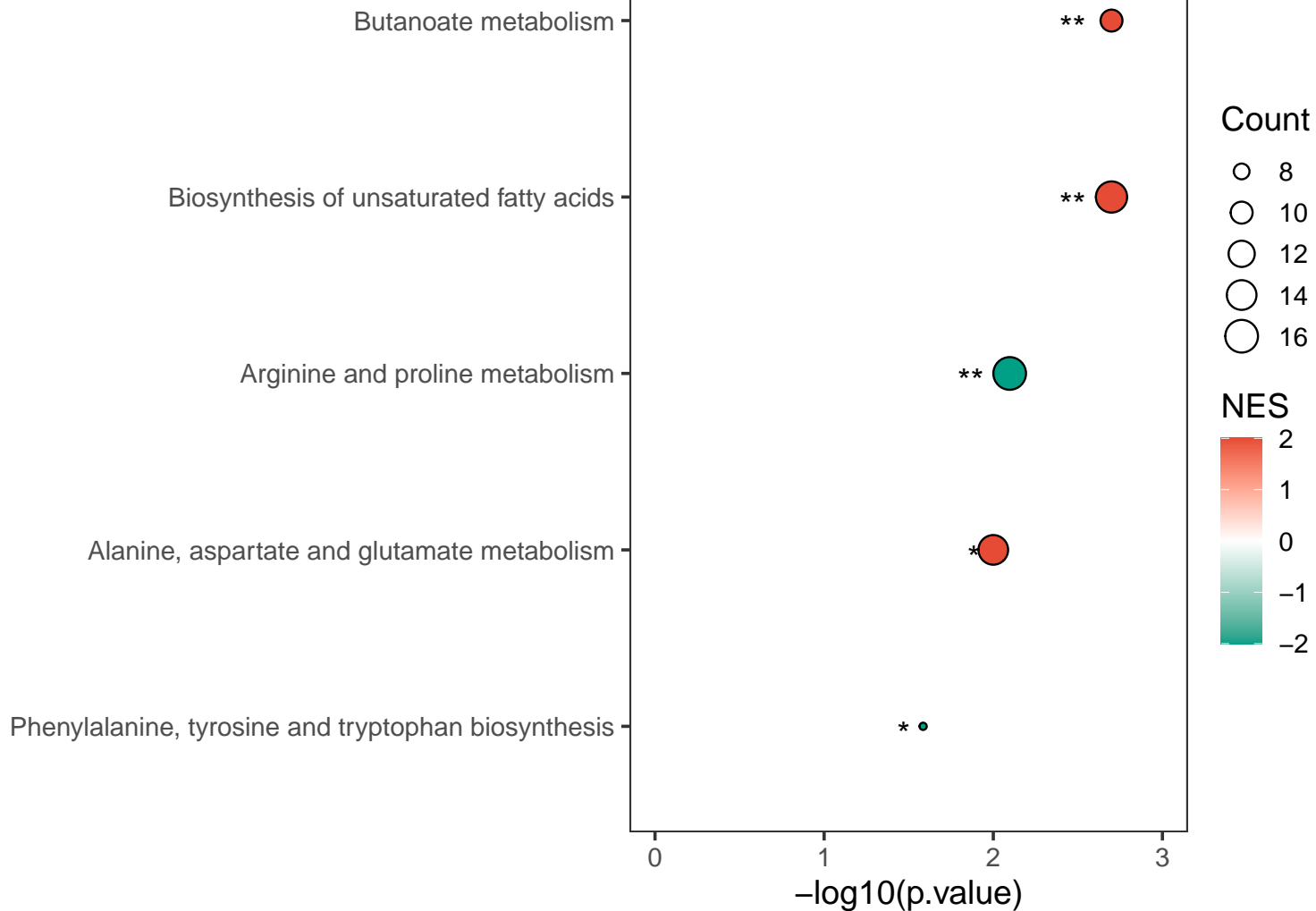

Supplement: Supplementary file 1 [file ijms-27-06236-s001.zip › Supplementary Materials/ijms-4276706_Metabolomics_Dataset/4-Functional Annotation and Enrichment/Figure 4d. MSEA enrichment1 of Model-vs-Paeoniflorin.pdf]

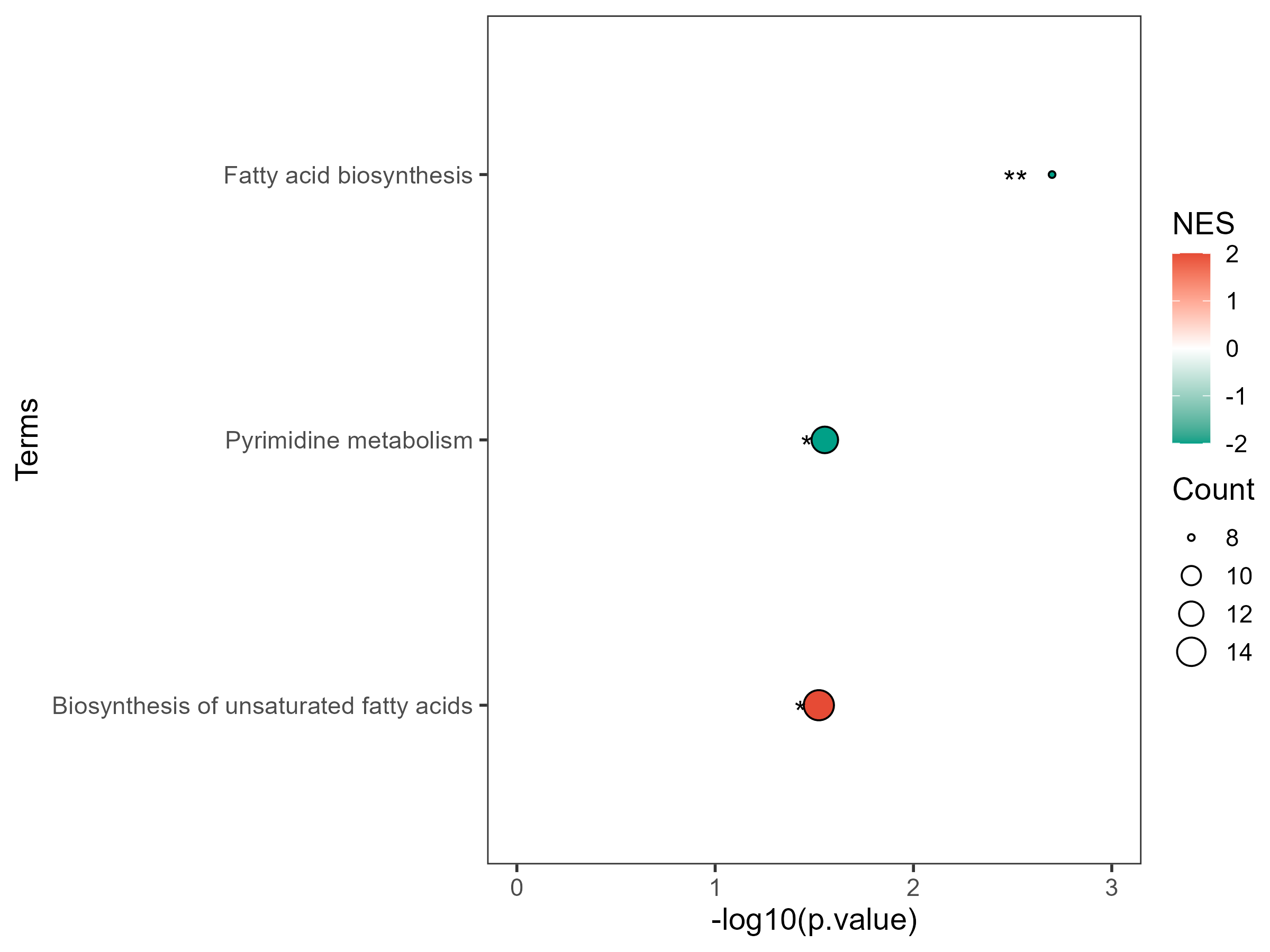

Supplement: Supplementary file 1 [file ijms-27-06236-s001.zip › Supplementary Materials/ijms-4276706_Metabolomics_Dataset/4-Functional Annotation and Enrichment/Figure 4d. MSEA enrichment1 of Control-vs-Model.png]

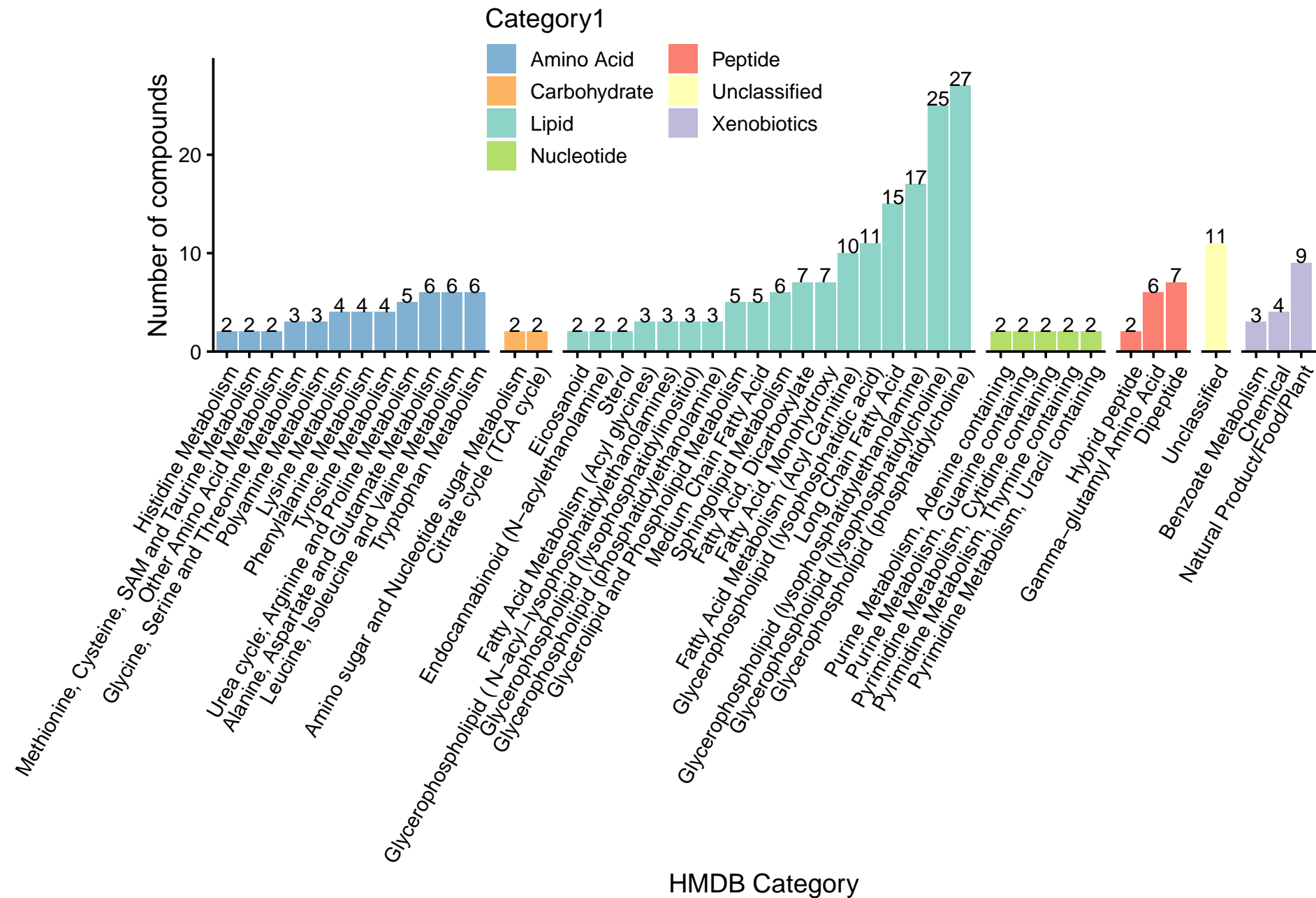

Supplement: Supplementary file 1 [file ijms-27-06236-s001.zip › Supplementary Materials/ijms-4276706_Metabolomics_Dataset/4-Functional Annotation and Enrichment/Figure 4a. Diff Metabolite Classification by Category of Control-vs-Model.pdf]

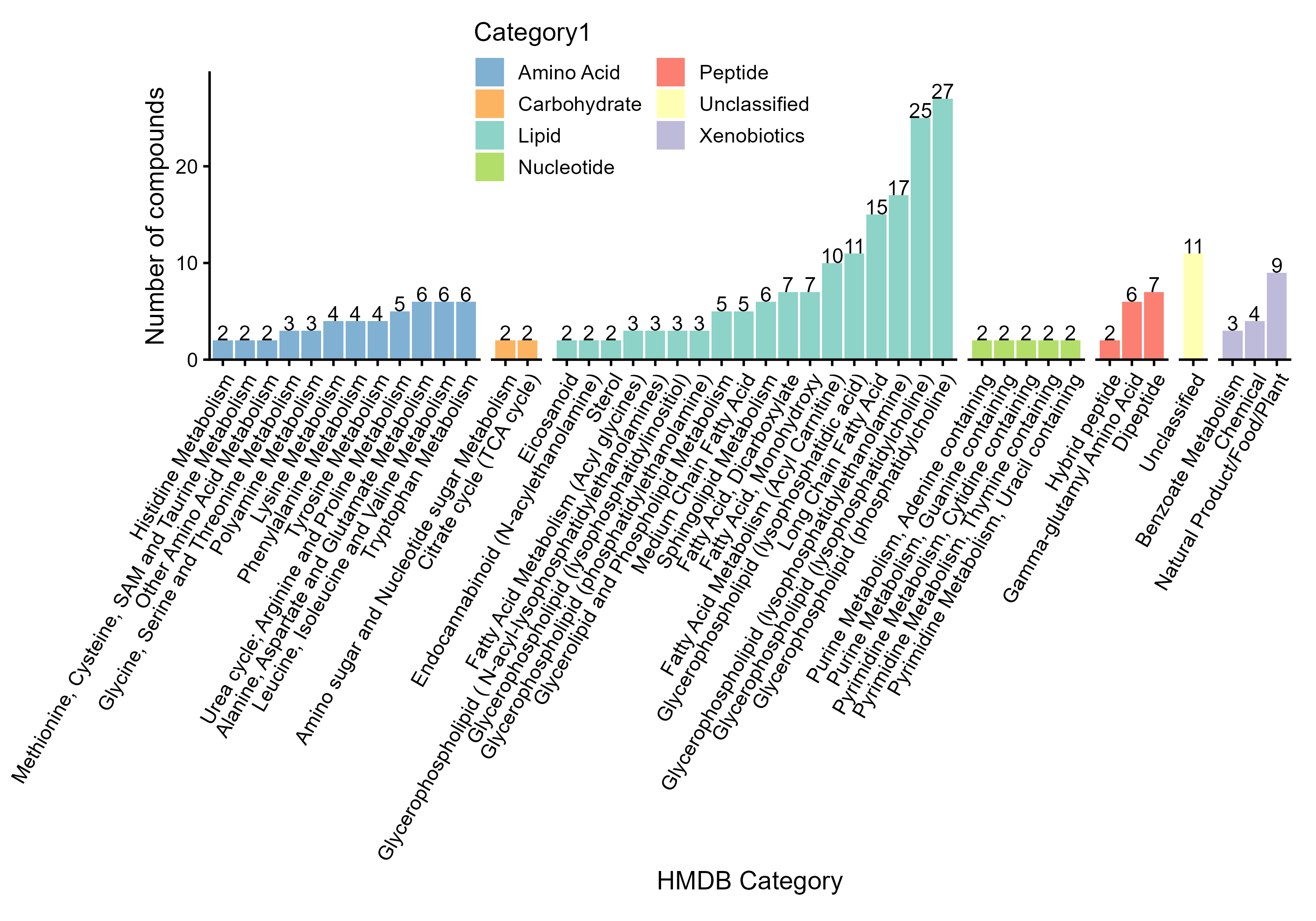

Supplement: Supplementary file 1 [file ijms-27-06236-s001.zip › Supplementary Materials/ijms-4276706_Metabolomics_Dataset/4-Functional Annotation and Enrichment/Figure 4a. Diff Metabolite Classification by Category of Control-vs-Model.png]

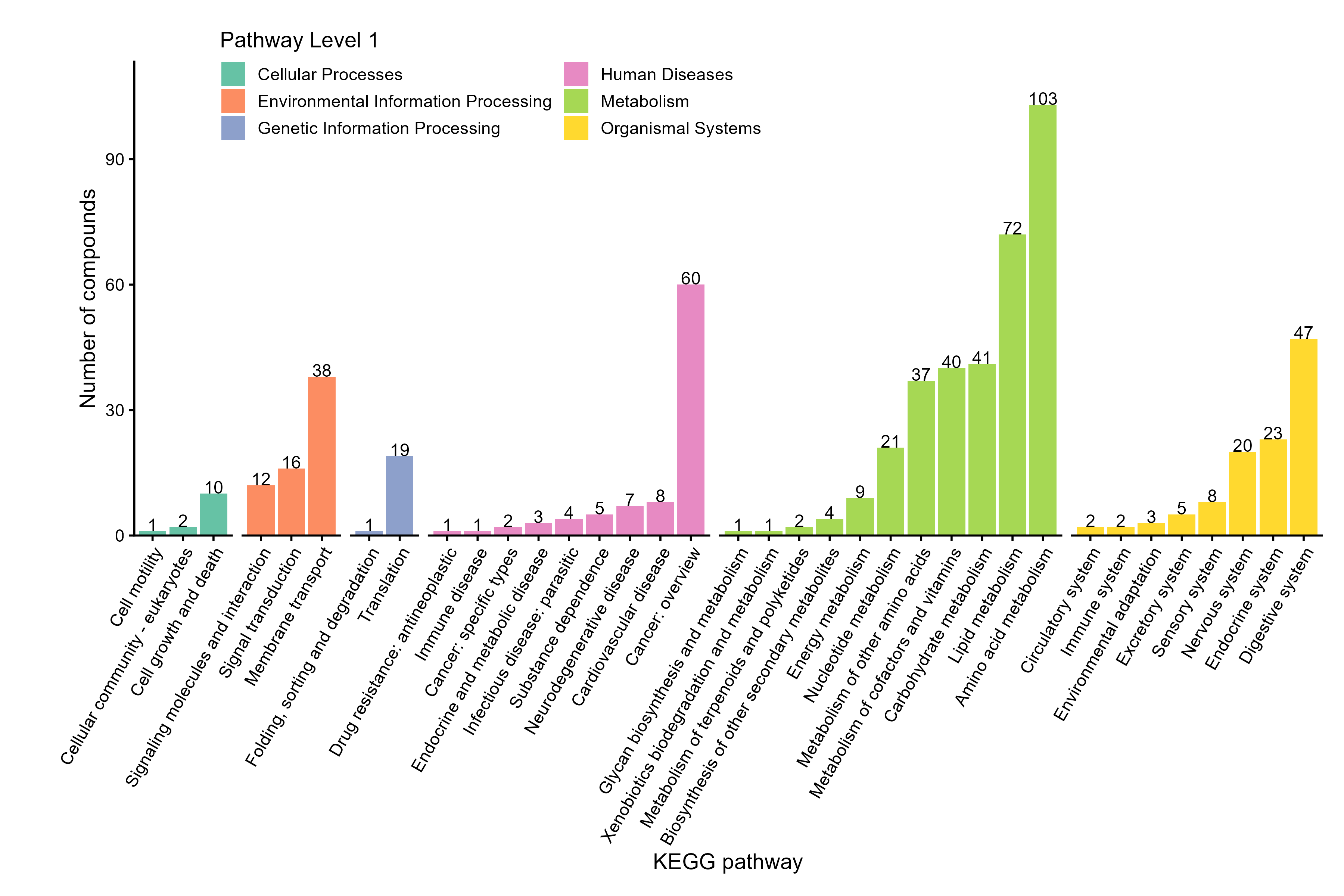

Supplement: Supplementary file 1 [file ijms-27-06236-s001.zip › Supplementary Materials/ijms-4276706_Metabolomics_Dataset/2-Metabolite Functional Classification/Figure 2b. Metabolites Classification by Pathway.png]

# Pathway Level 1

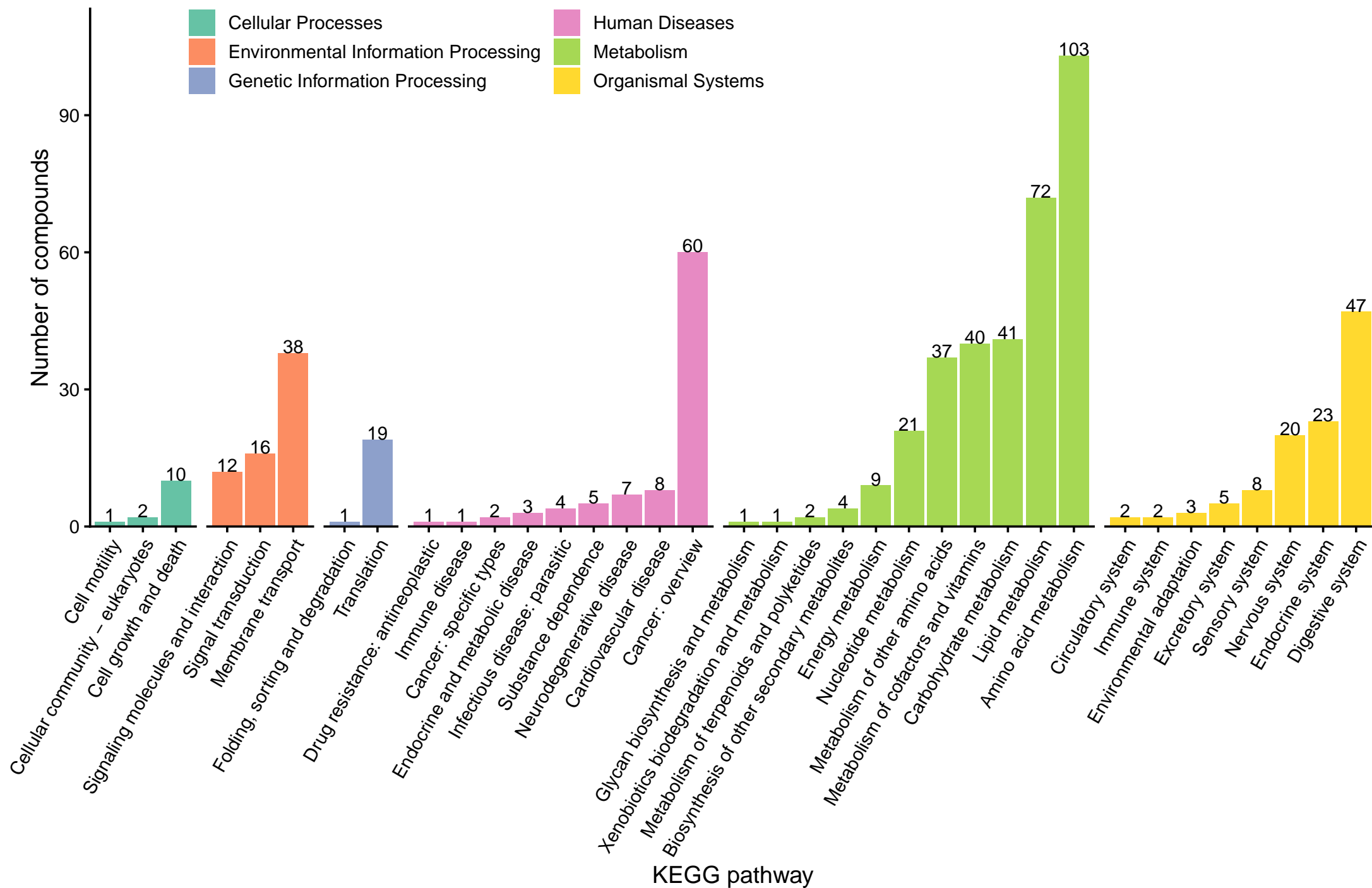

Supplement: Supplementary file 1 [file ijms-27-06236-s001.zip › Supplementary Materials/ijms-4276706_Metabolomics_Dataset/2-Metabolite Functional Classification/Figure 2b. Metabolites Classification by Pathway.pdf]

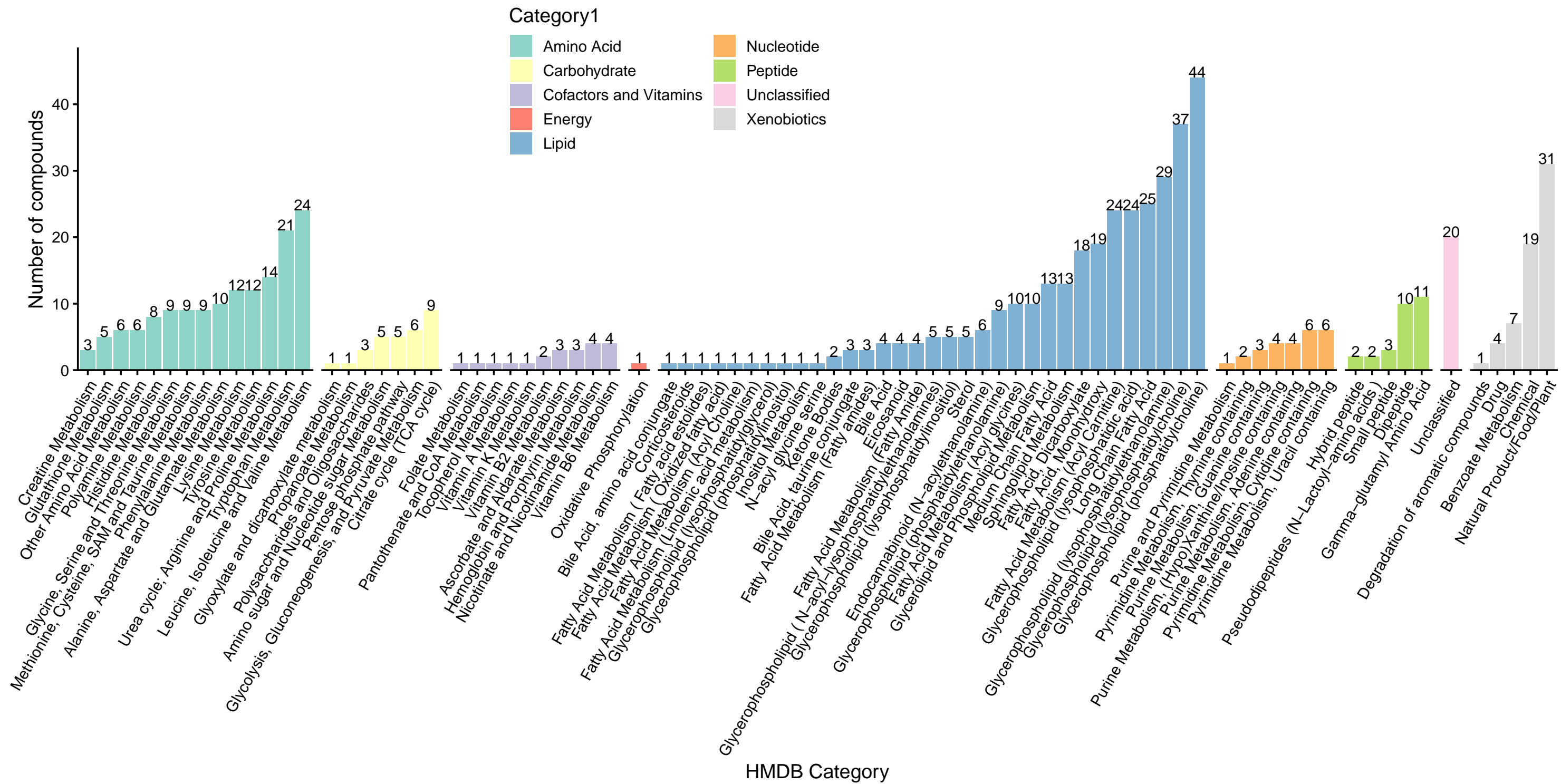

Supplement: Supplementary file 1 [file ijms-27-06236-s001.zip › Supplementary Materials/ijms-4276706_Metabolomics_Dataset/2-Metabolite Functional Classification/Figure 2a. Metabolites Classification by Category.pdf]

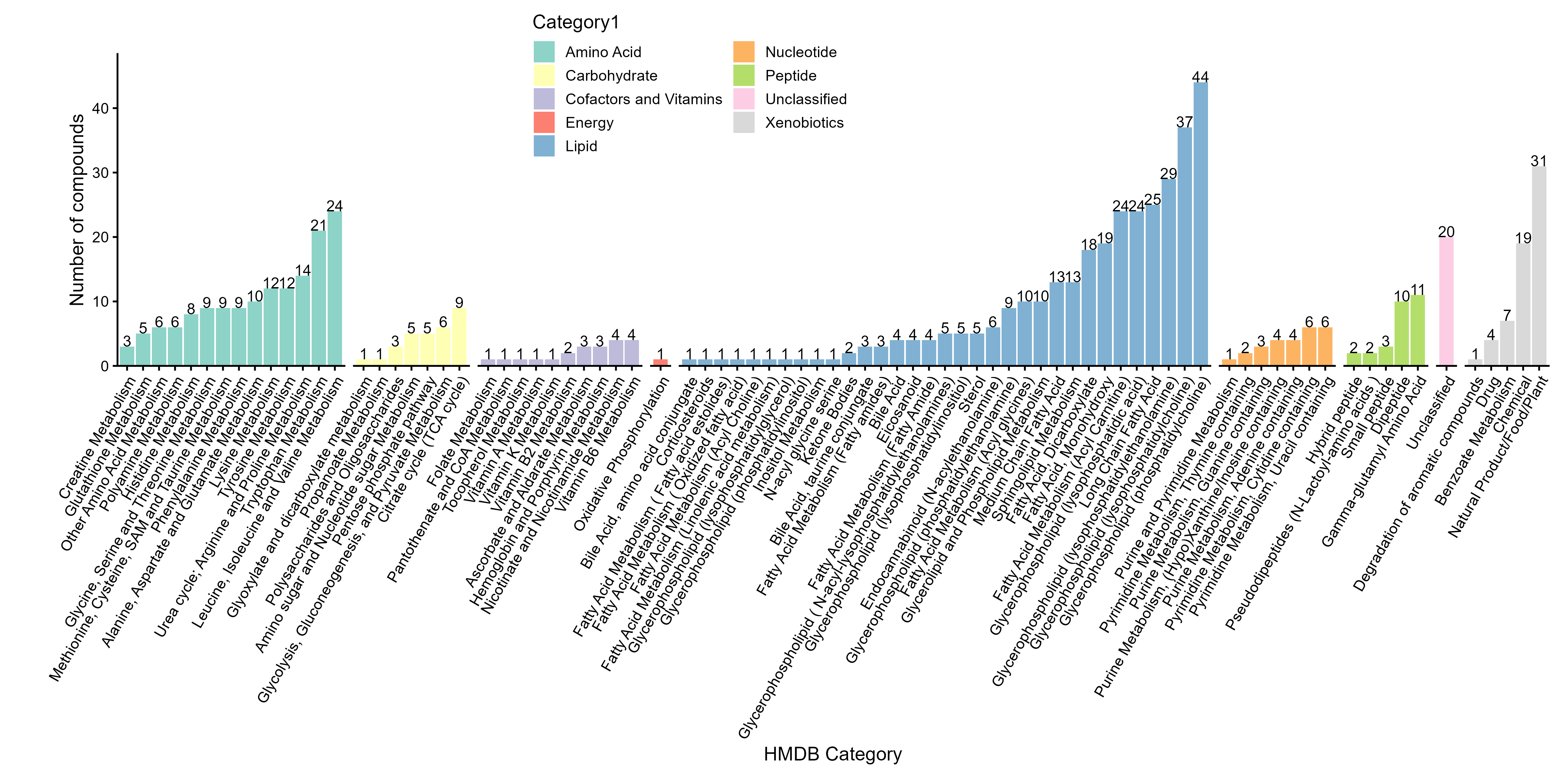

Supplement: Supplementary file 1 [file ijms-27-06236-s001.zip › Supplementary Materials/ijms-4276706_Metabolomics_Dataset/2-Metabolite Functional Classification/Figure 2a. Metabolites Classification by Category.png]

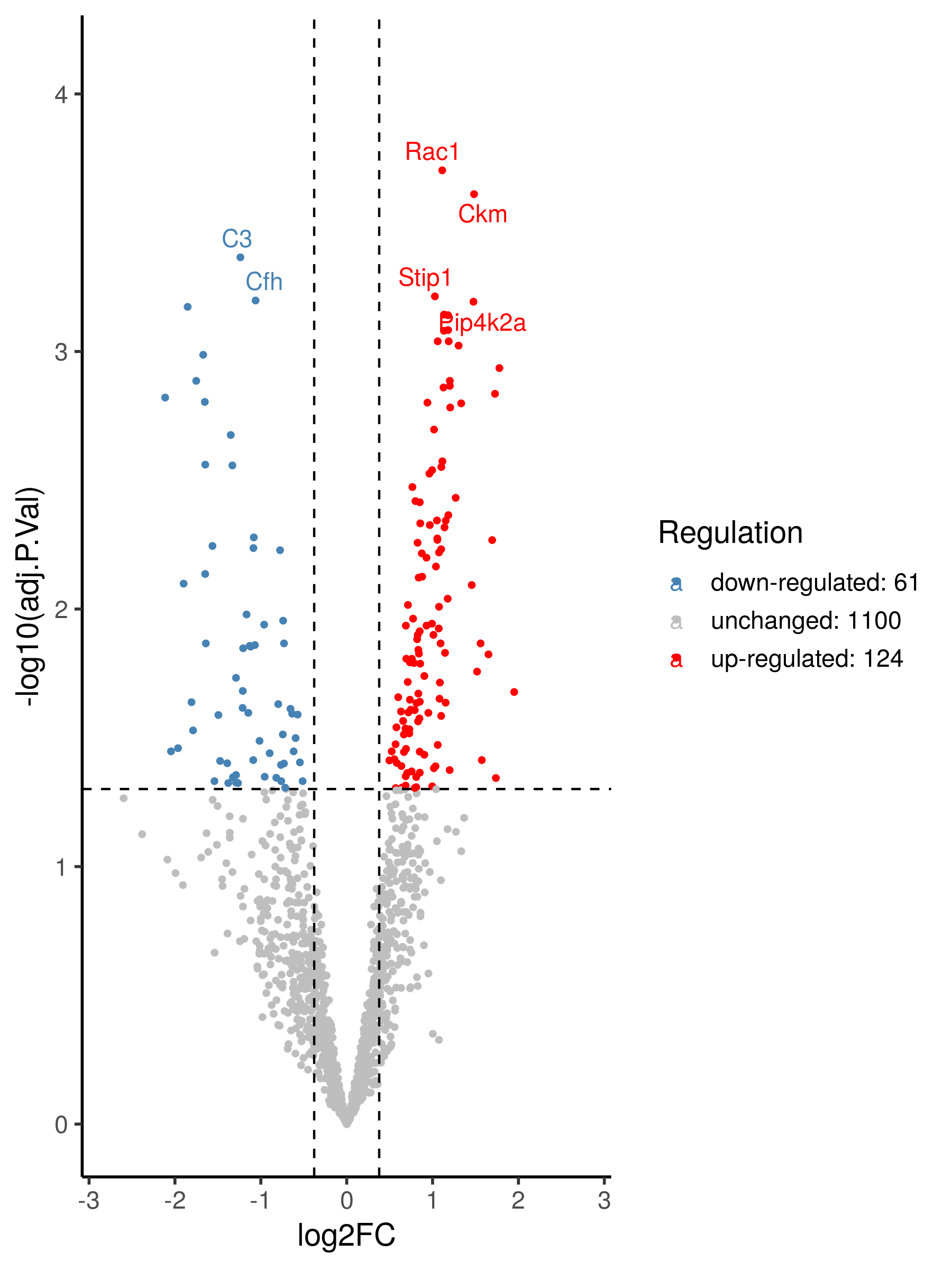

Supplement: Supplementary file 1 [file ijms-27-06236-s001.zip › Supplementary Materials/ijms-4276706_Proteomics_Dataset/2-Differentially_expressed_protein/Figure 2a. DEPs volcano of Model-vs-Paeoniflorin.png]

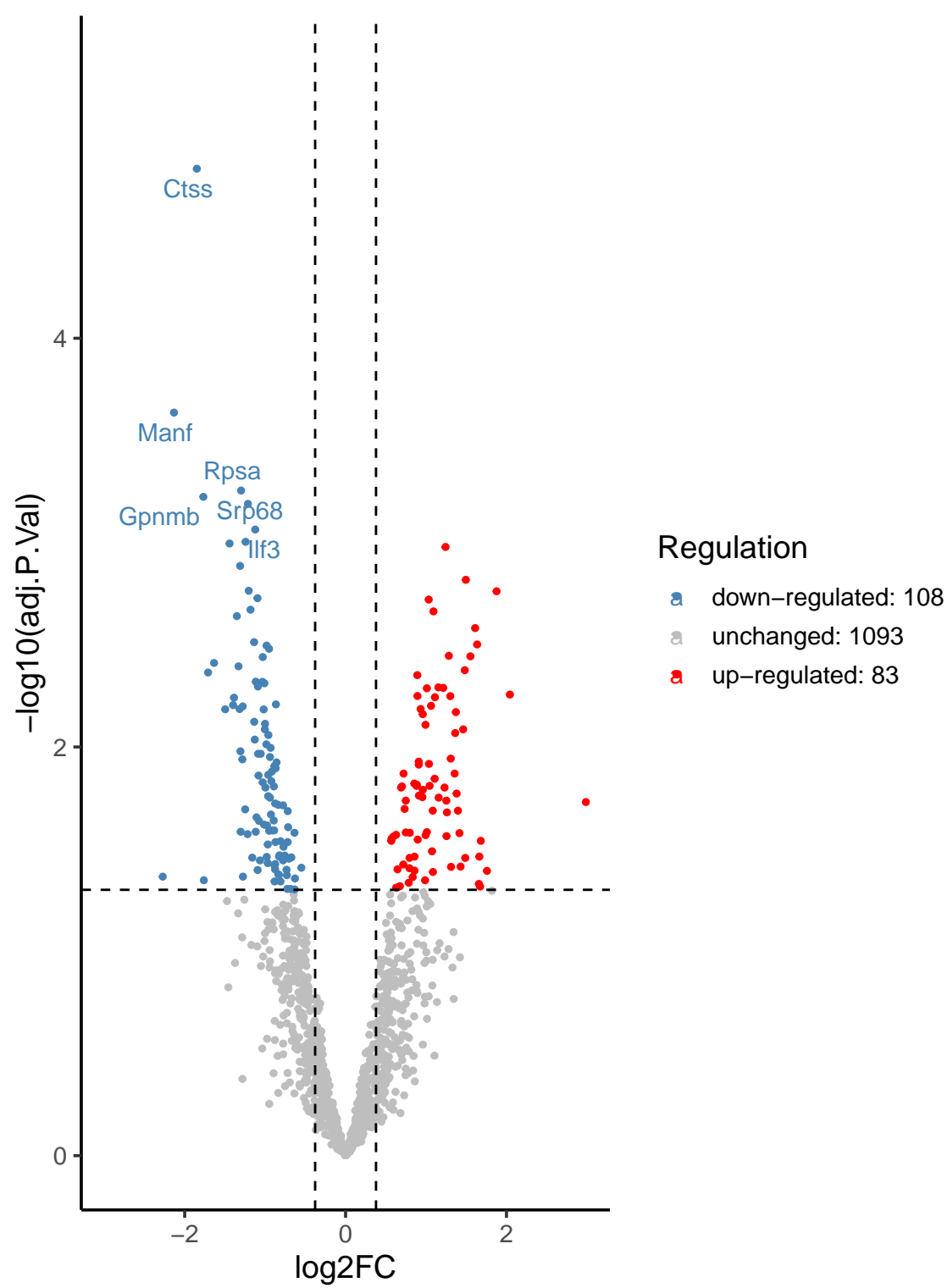

Supplement: Supplementary file 1 [file ijms-27-06236-s001.zip › Supplementary Materials/ijms-4276706_Proteomics_Dataset/2-Differentially_expressed_protein/Figure 2a. DEPs volcano of Control-vs-Model.pdf]

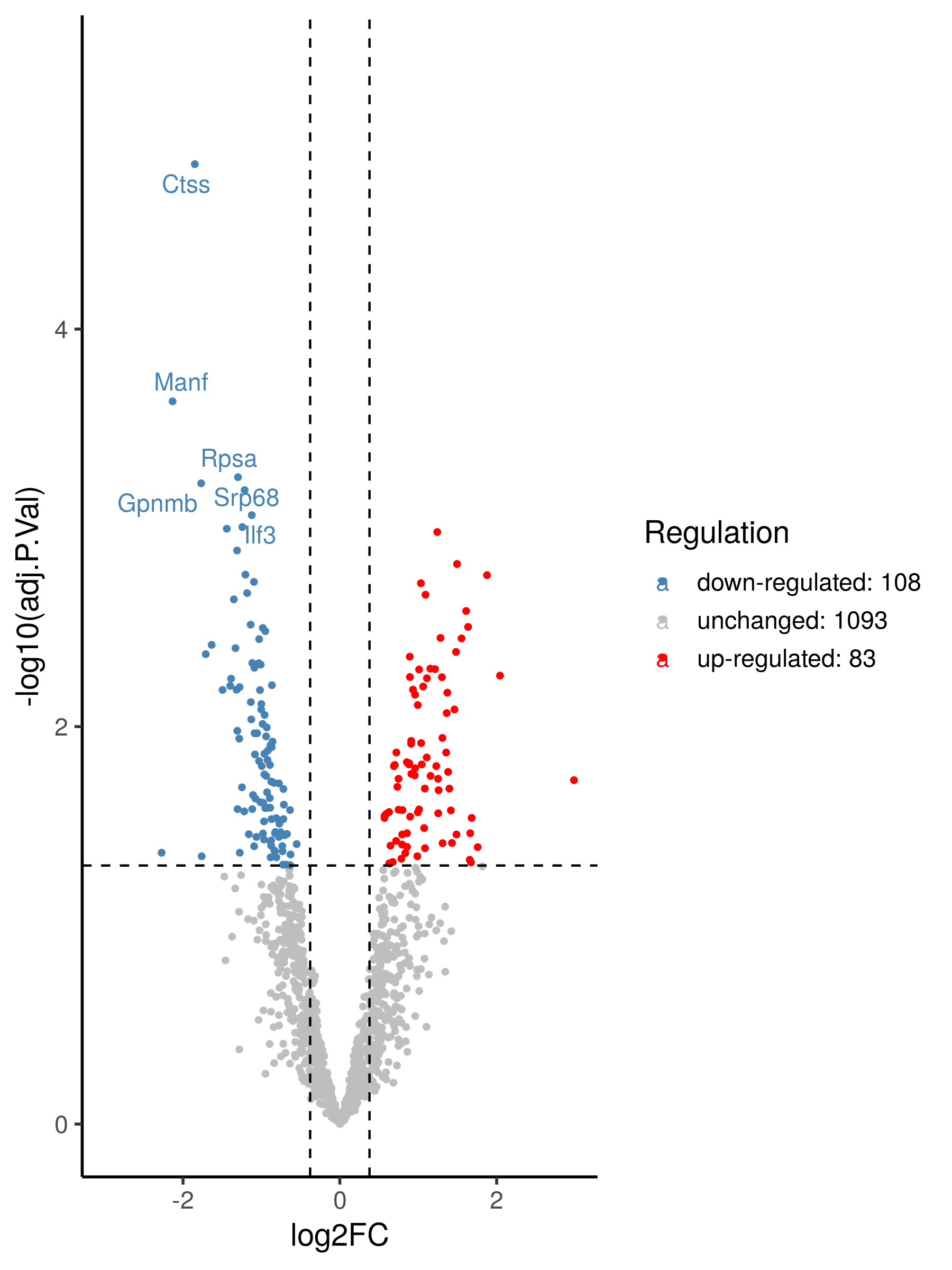

Supplement: Supplementary file 1 [file ijms-27-06236-s001.zip › Supplementary Materials/ijms-4276706_Proteomics_Dataset/2-Differentially_expressed_protein/Figure 2a. DEPs volcano of Control-vs-Model.png]

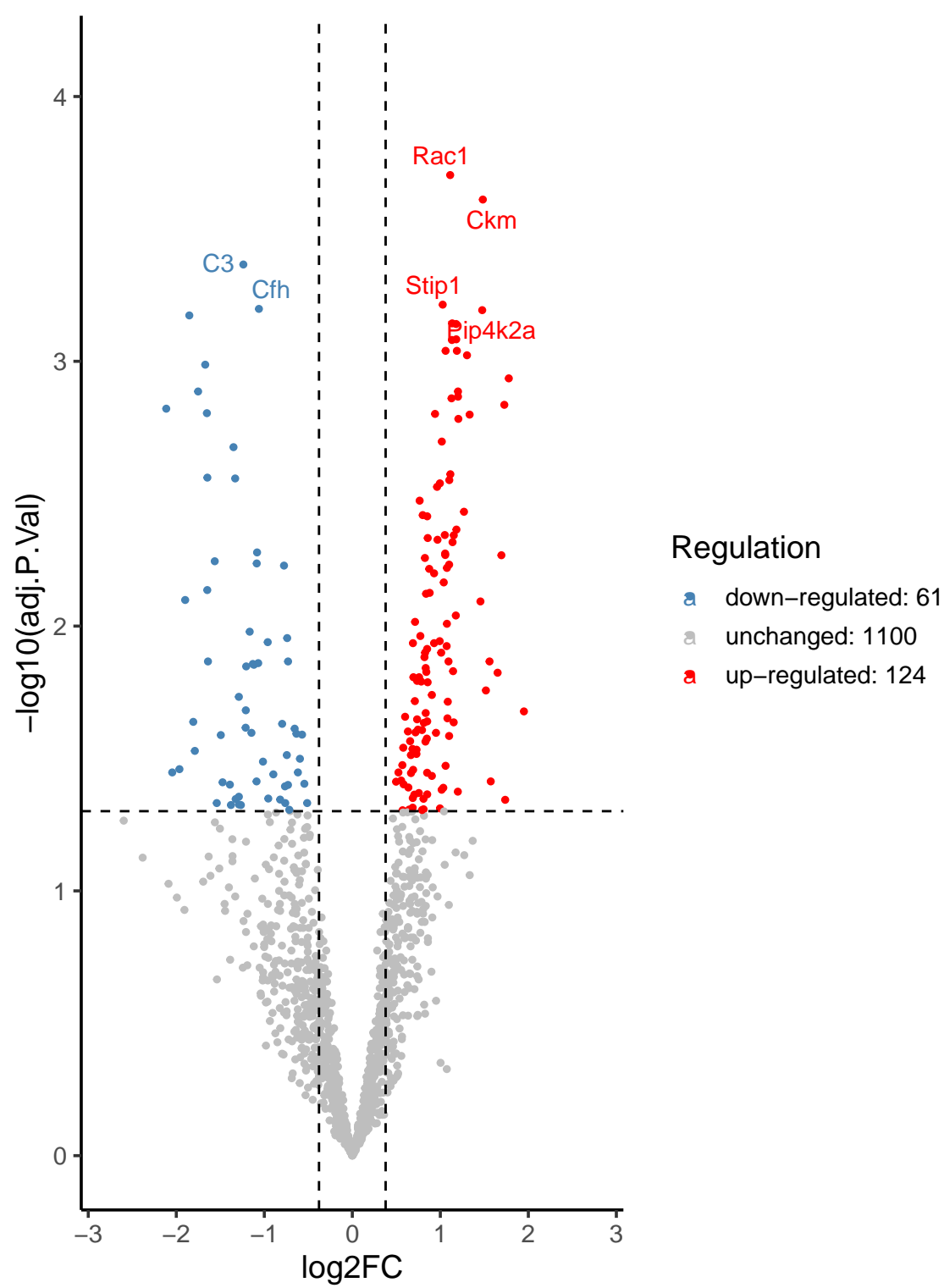

Supplement: Supplementary file 1 [file ijms-27-06236-s001.zip › Supplementary Materials/ijms-4276706_Proteomics_Dataset/2-Differentially_expressed_protein/Figure 2a. DEPs volcano of Model-vs-Paeoniflorin.pdf]

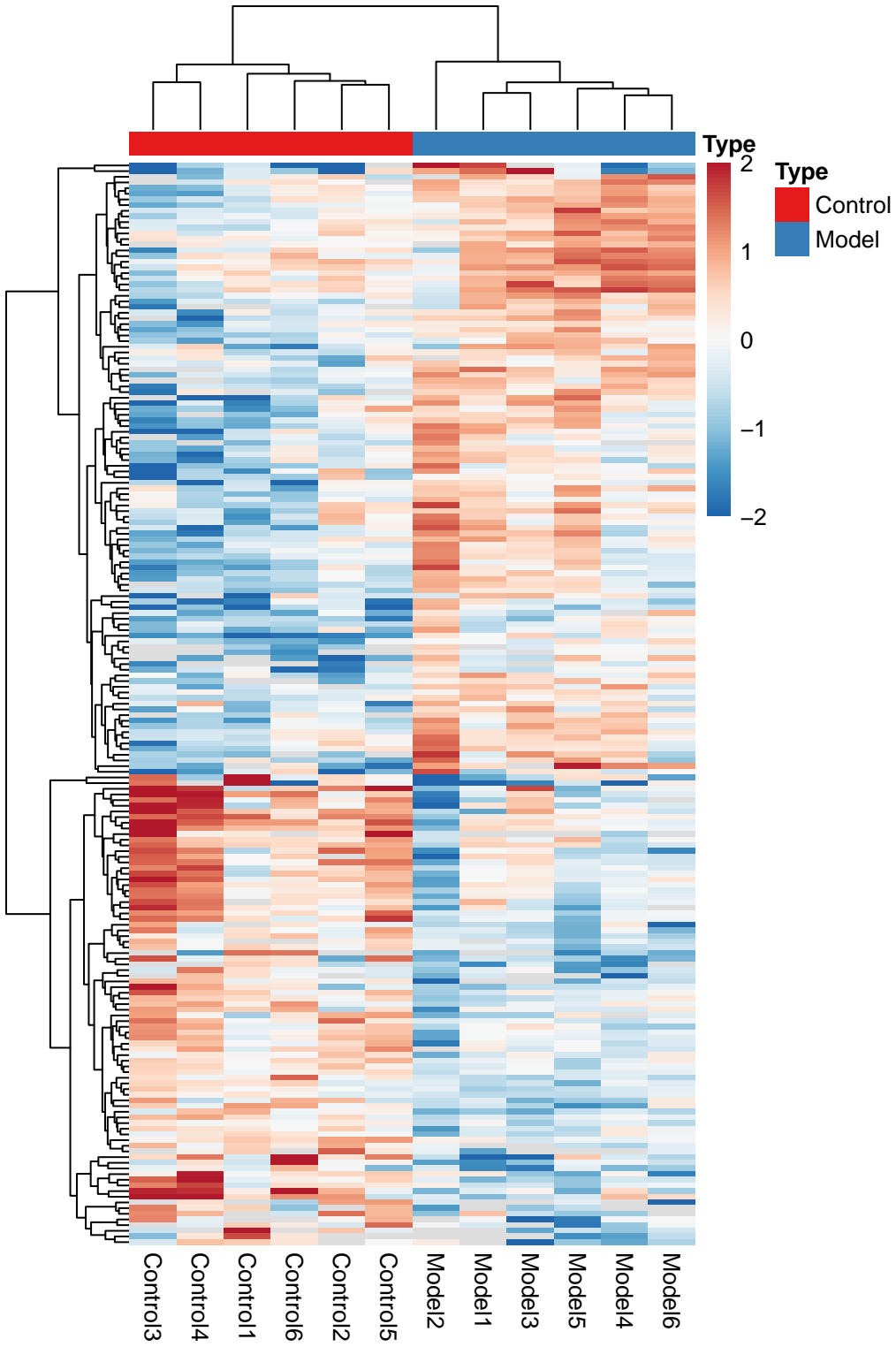

Supplement: Supplementary file 1 [file ijms-27-06236-s001.zip › Supplementary Materials/ijms-4276706_Proteomics_Dataset/2-Differentially_expressed_protein/Figure 2b. DEPs heatmap of Control-vs-Model.pdf]

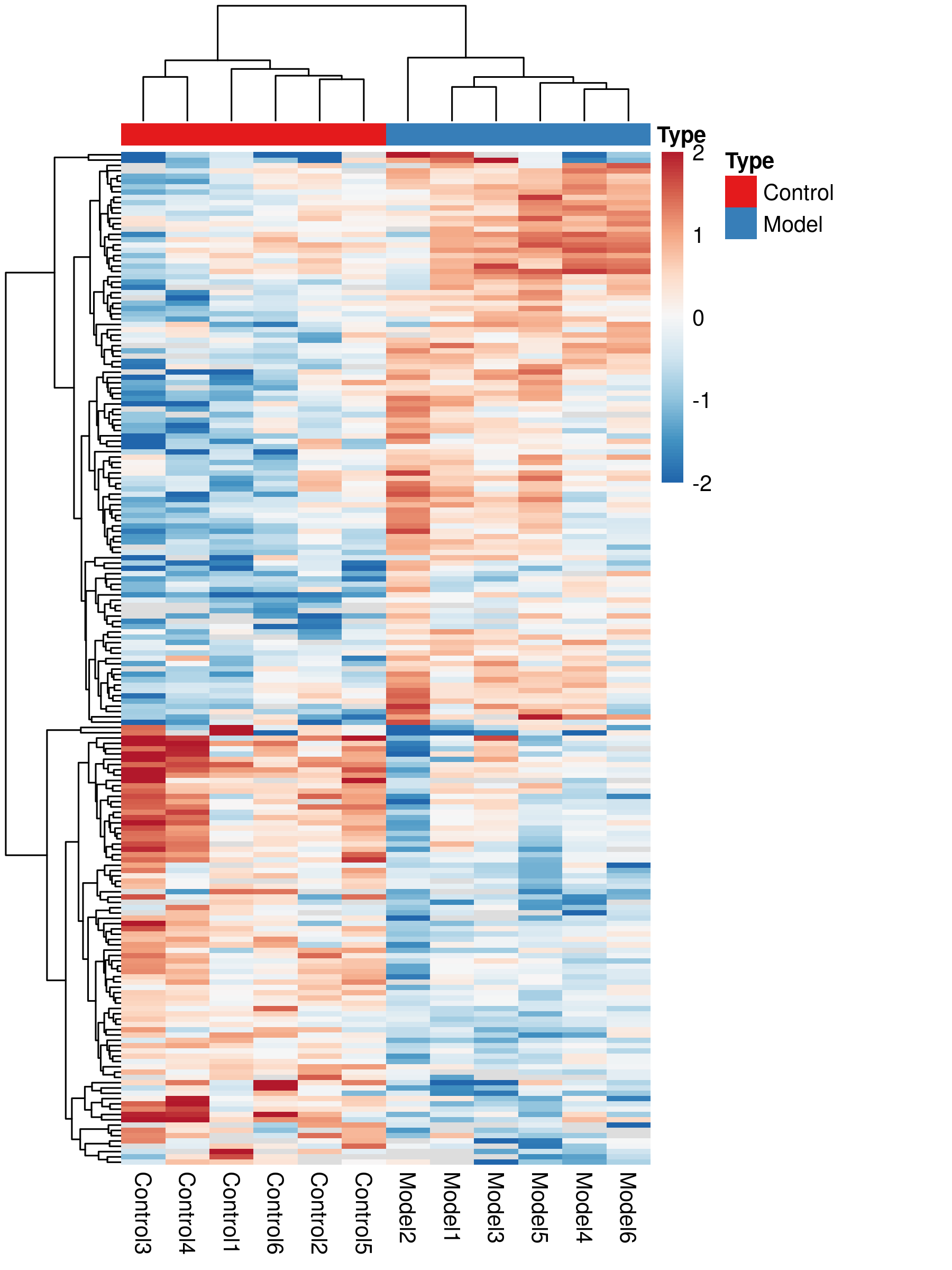

Supplement: Supplementary file 1 [file ijms-27-06236-s001.zip › Supplementary Materials/ijms-4276706_Proteomics_Dataset/2-Differentially_expressed_protein/Figure 2b. DEPs heatmap of Control-vs-Model.png]

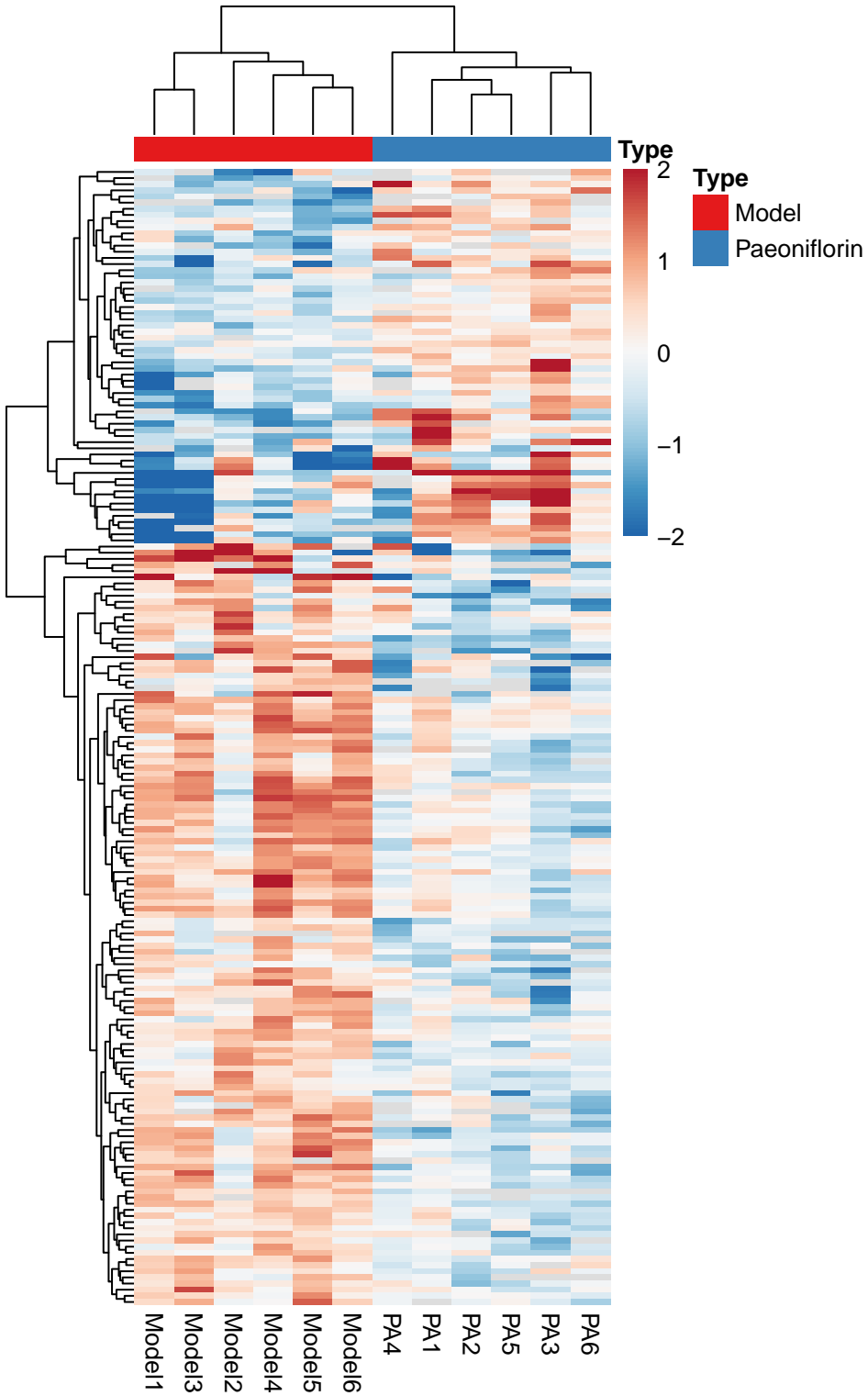

Supplement: Supplementary file 1 [file ijms-27-06236-s001.zip › Supplementary Materials/ijms-4276706_Proteomics_Dataset/2-Differentially_expressed_protein/Figure 2b. DEPs heatmap of Model-vs-Paeoniflorin.pdf]

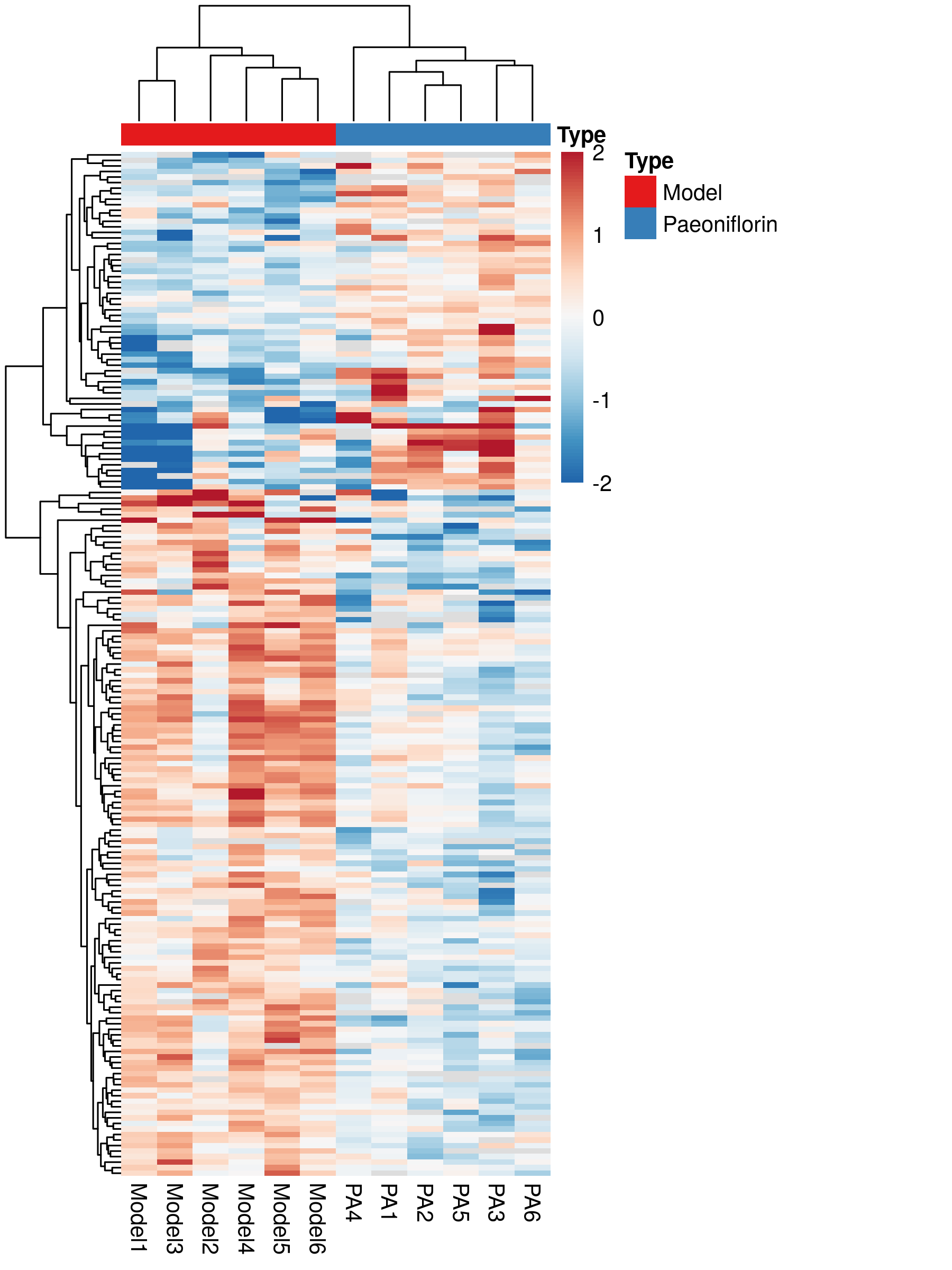

Supplement: Supplementary file 1 [file ijms-27-06236-s001.zip › Supplementary Materials/ijms-4276706_Proteomics_Dataset/2-Differentially_expressed_protein/Figure 2b. DEPs heatmap of Model-vs-Paeoniflorin.png]

Category ■ Biological process ■ Cellular component ■ Molecular function

Terms

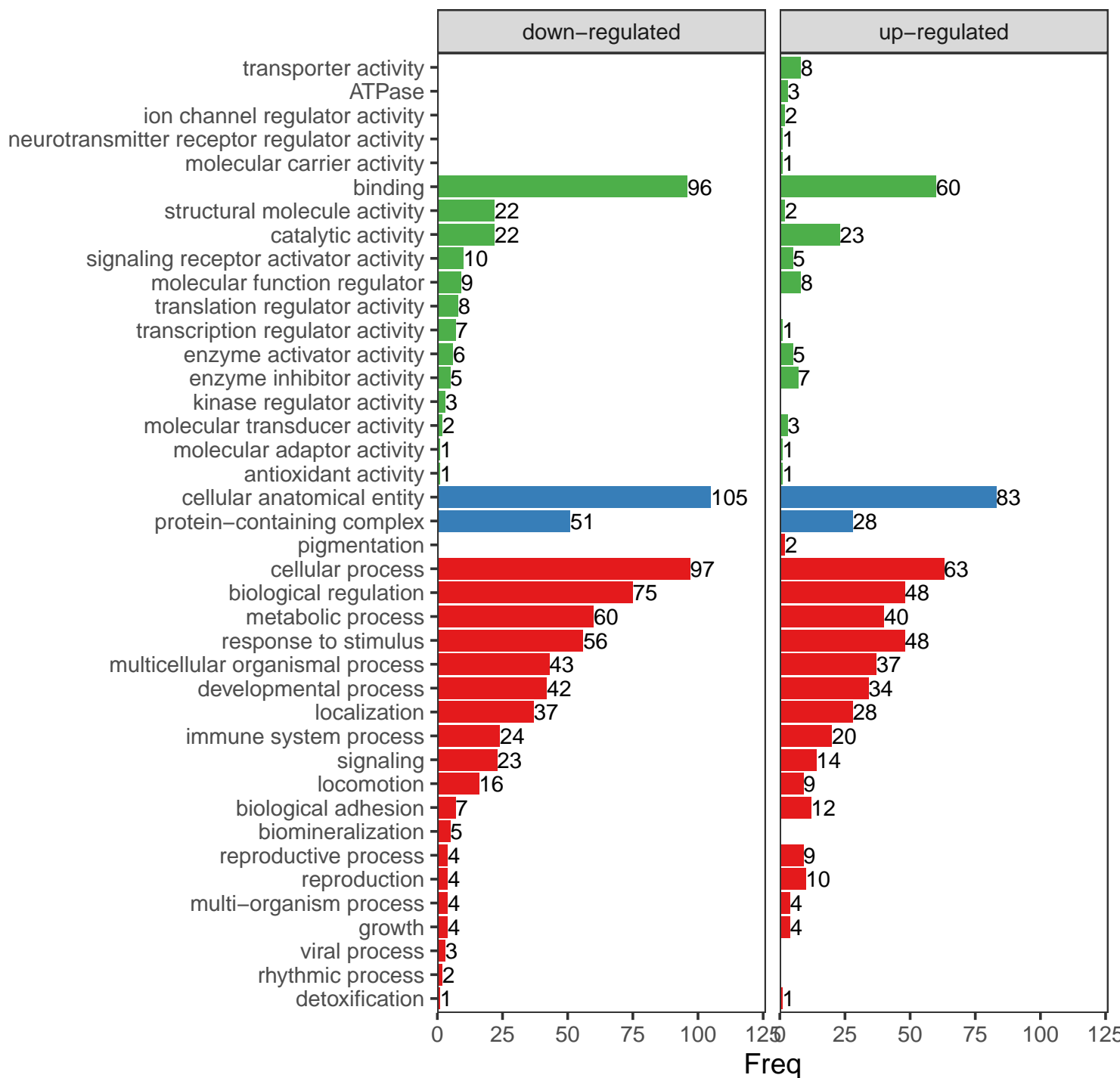

Supplement: Supplementary file 1 [file ijms-27-06236-s001.zip › Supplementary Materials/ijms-4276706_Proteomics_Dataset/4-Functional_classification/Figure 3a. GO classify of Control-vs-Model.pdf]

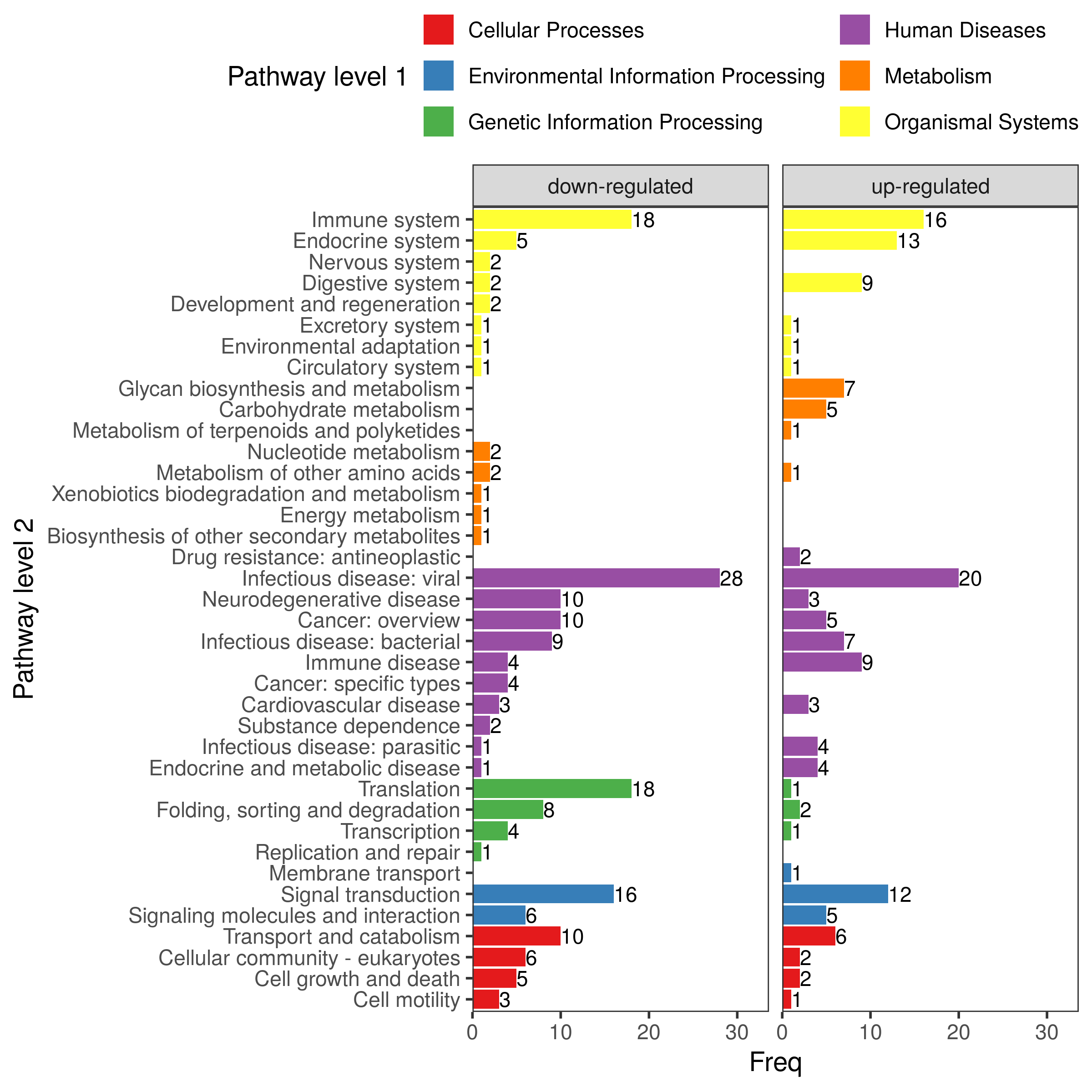

Supplement: Supplementary file 1 [file ijms-27-06236-s001.zip › Supplementary Materials/ijms-4276706_Proteomics_Dataset/4-Functional_classification/Figure 3d. KEGG classify of Control-vs-Model.png]

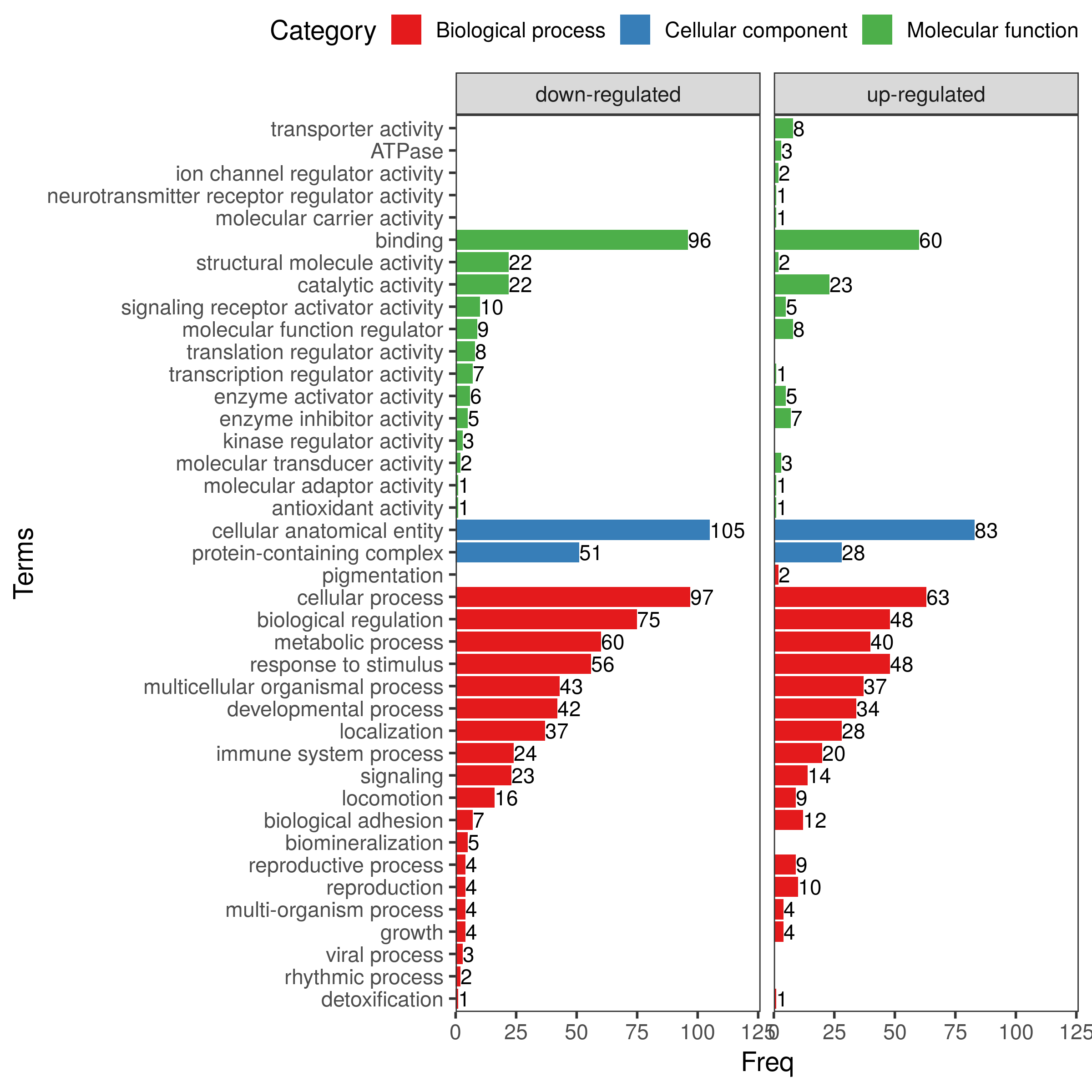

Supplement: Supplementary file 1 [file ijms-27-06236-s001.zip › Supplementary Materials/ijms-4276706_Proteomics_Dataset/4-Functional_classification/Figure 3a. GO classify of Control-vs-Model.png]

Pathway level 1

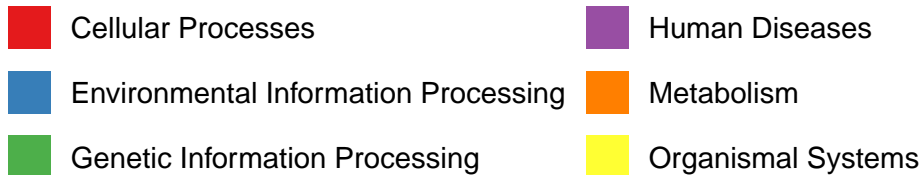

Pathway level 2

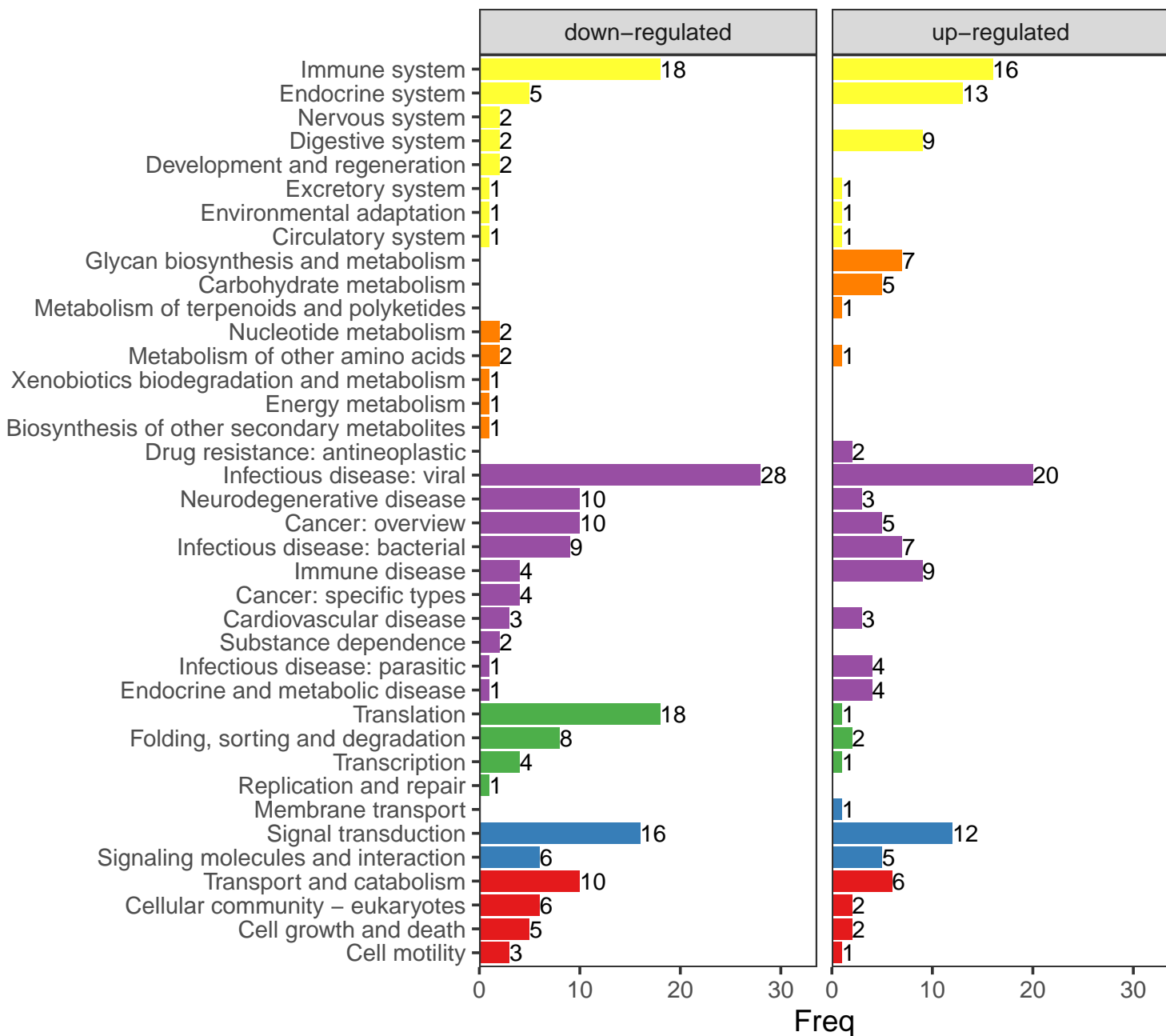

Supplement: Supplementary file 1 [file ijms-27-06236-s001.zip › Supplementary Materials/ijms-4276706_Proteomics_Dataset/4-Functional_classification/Figure 3d. KEGG classify of Control-vs-Model.pdf]

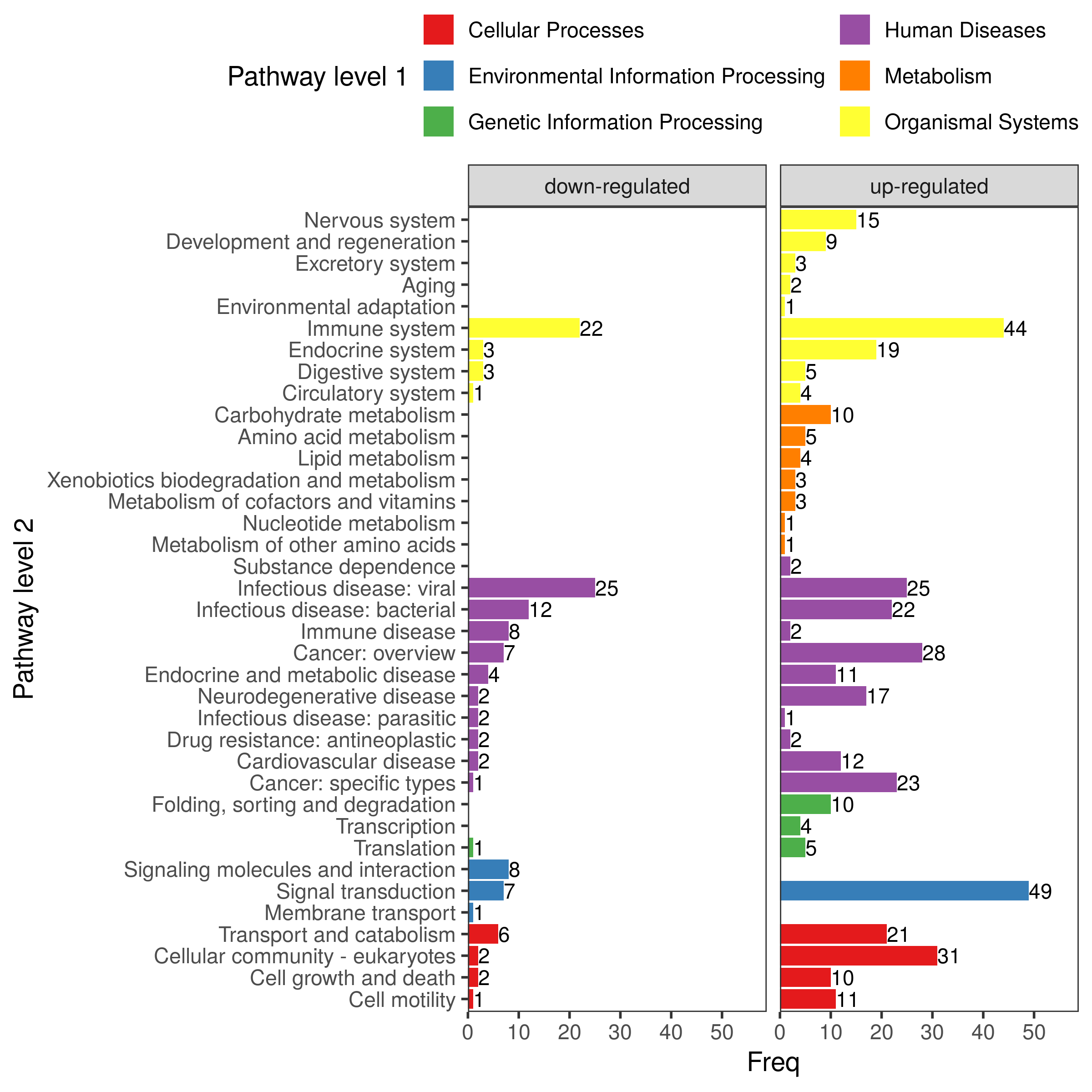

Supplement: Supplementary file 1 [file ijms-27-06236-s001.zip › Supplementary Materials/ijms-4276706_Proteomics_Dataset/4-Functional_classification/Figure 3d. KEGG classify of Model-vs-Paeoniflorin.png]

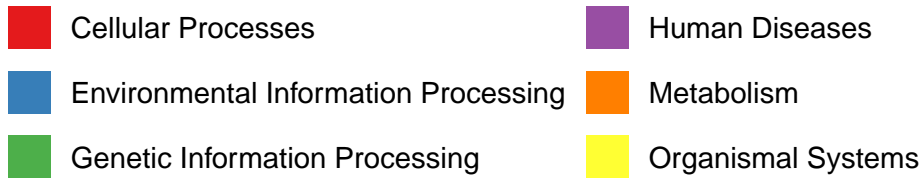

Pathway level 2

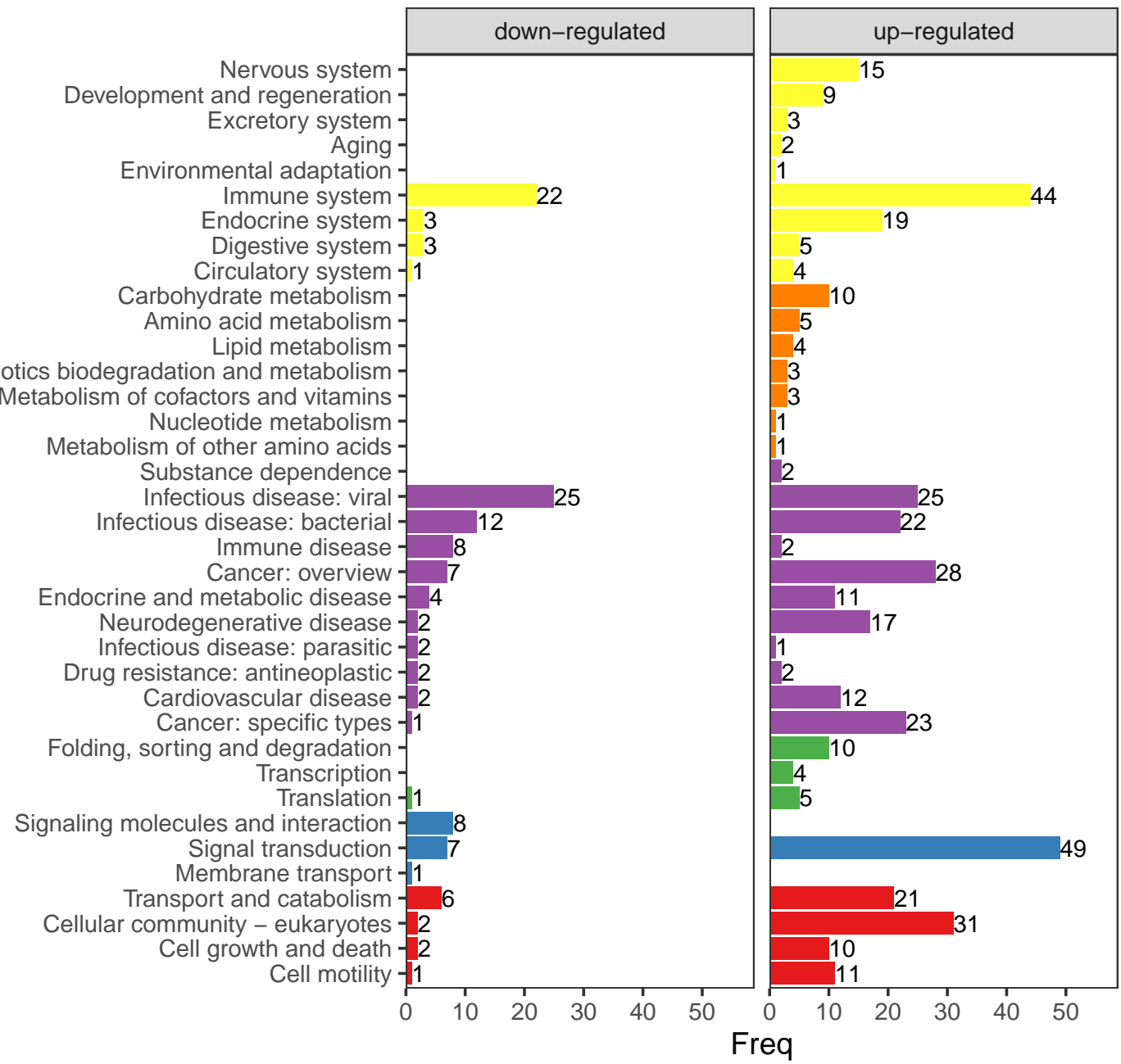

Supplement: Supplementary file 1 [file ijms-27-06236-s001.zip › Supplementary Materials/ijms-4276706_Proteomics_Dataset/4-Functional_classification/Figure 3d. KEGG classify of Model-vs-Paeoniflorin.pdf]

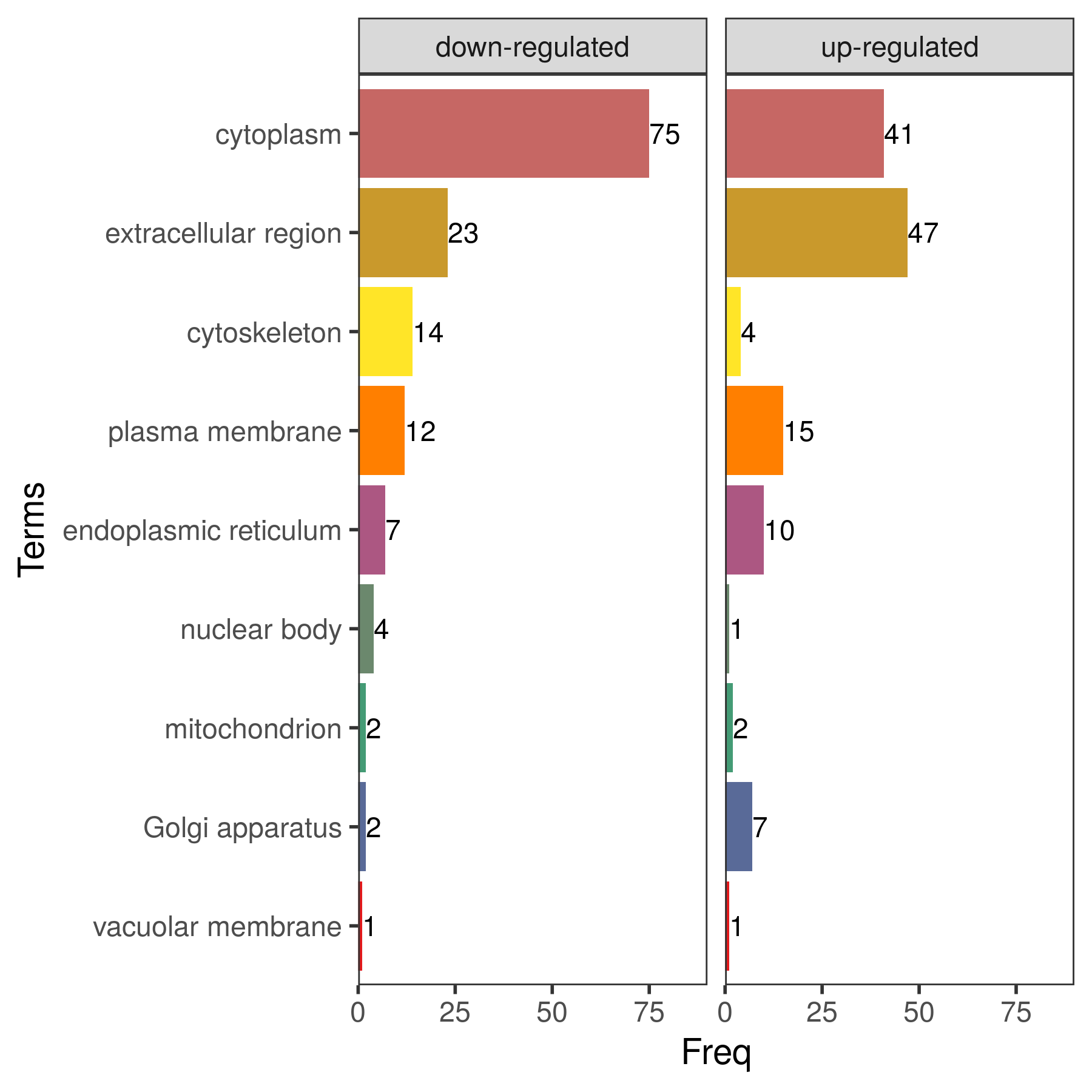

Supplement: Supplementary file 1 [file ijms-27-06236-s001.zip › Supplementary Materials/ijms-4276706_Proteomics_Dataset/4-Functional_classification/Figure 3c. Subcell classify of Control-vs-Model.png]

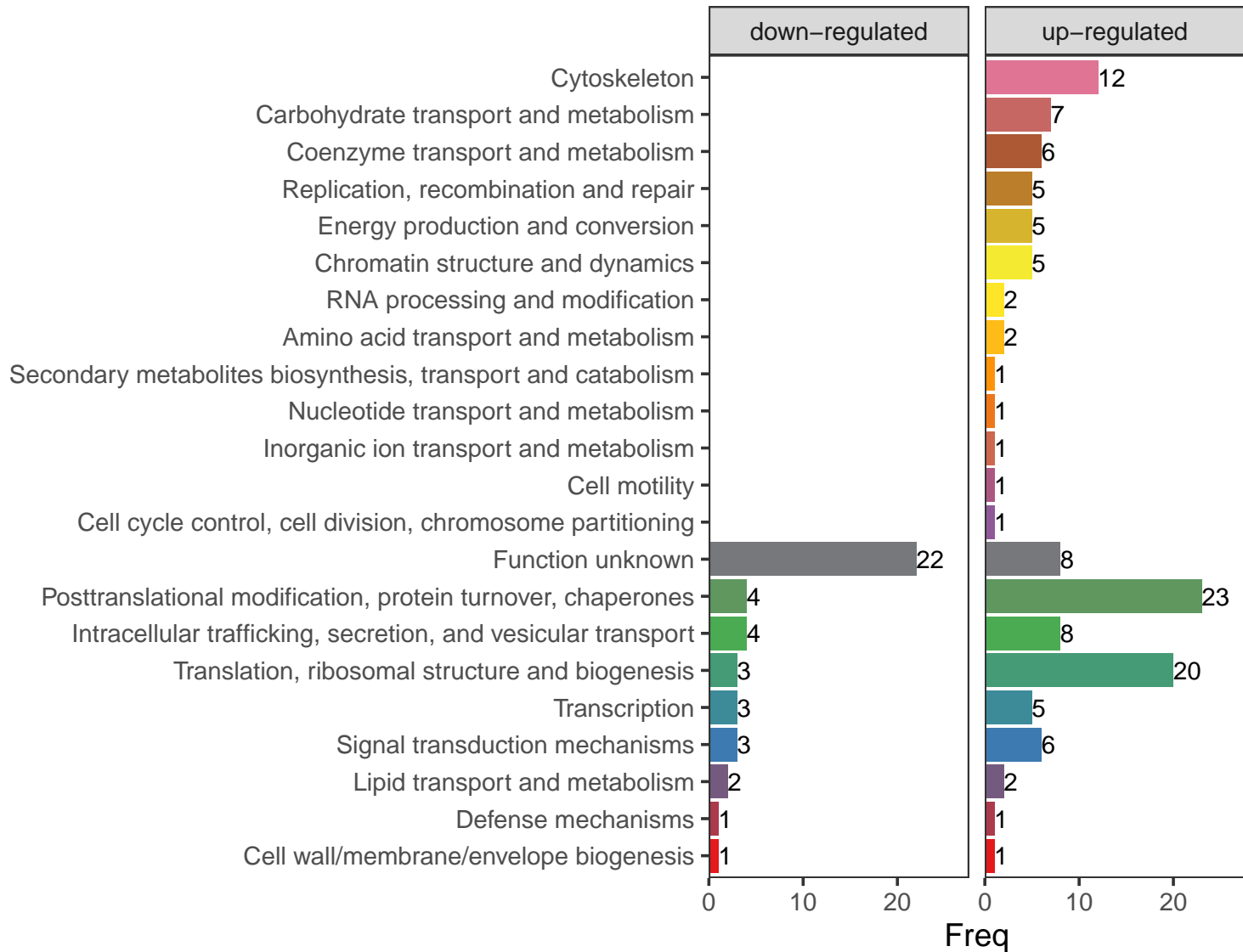

Supplement: Supplementary file 1 [file ijms-27-06236-s001.zip › Supplementary Materials/ijms-4276706_Proteomics_Dataset/4-Functional_classification/Figure 3b. KOG classify of Model-vs-Paeoniflorin.pdf]

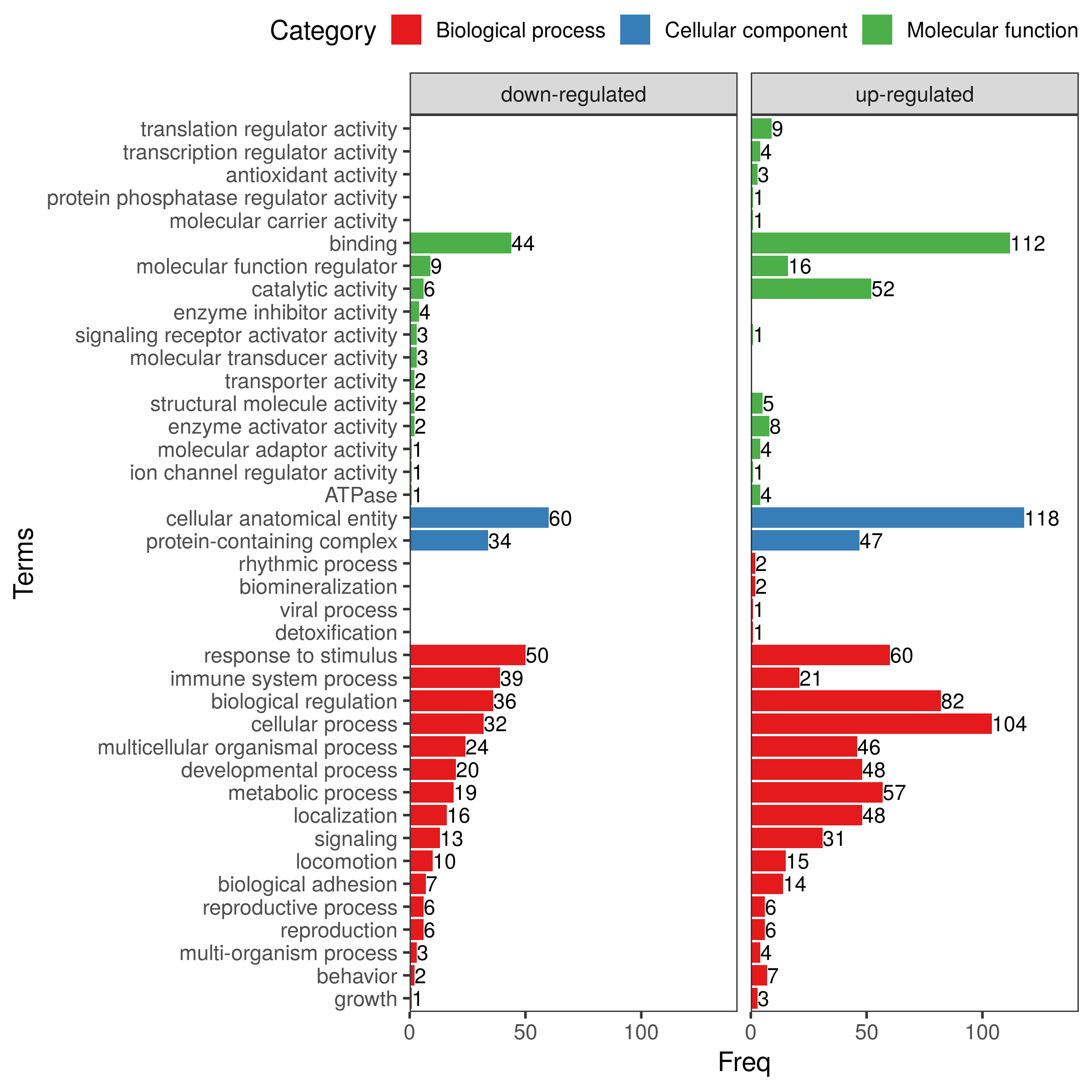

Supplement: Supplementary file 1 [file ijms-27-06236-s001.zip › Supplementary Materials/ijms-4276706_Proteomics_Dataset/4-Functional_classification/Figure 3a. GO classify of Model-vs-Paeoniflorin.png]

# Category

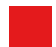

Biological process

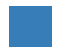

Cellular component

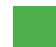

Molecular function

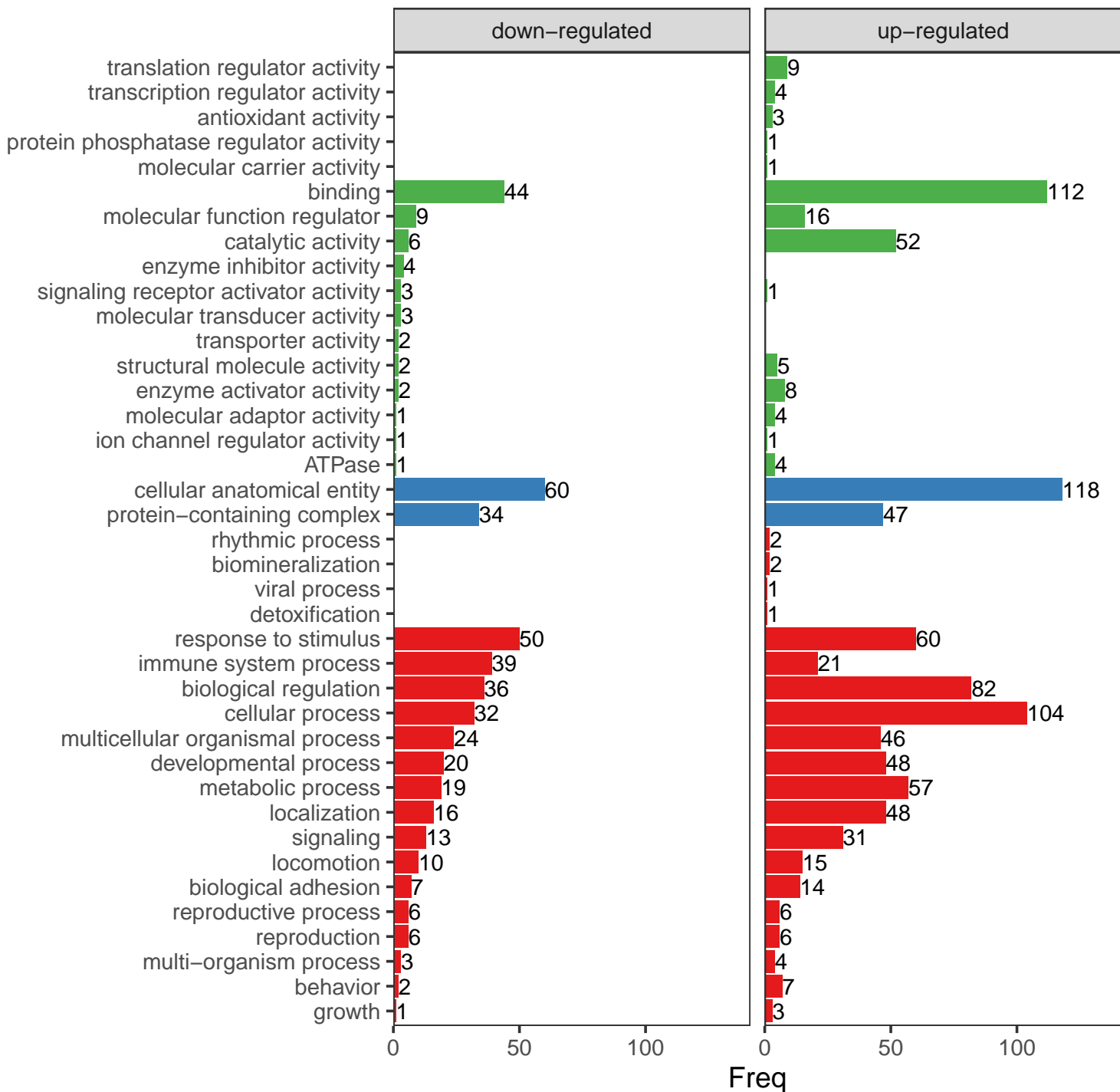

Supplement: Supplementary file 1 [file ijms-27-06236-s001.zip › Supplementary Materials/ijms-4276706_Proteomics_Dataset/4-Functional_classification/Figure 3a. GO classify of Model-vs-Paeoniflorin.pdf]

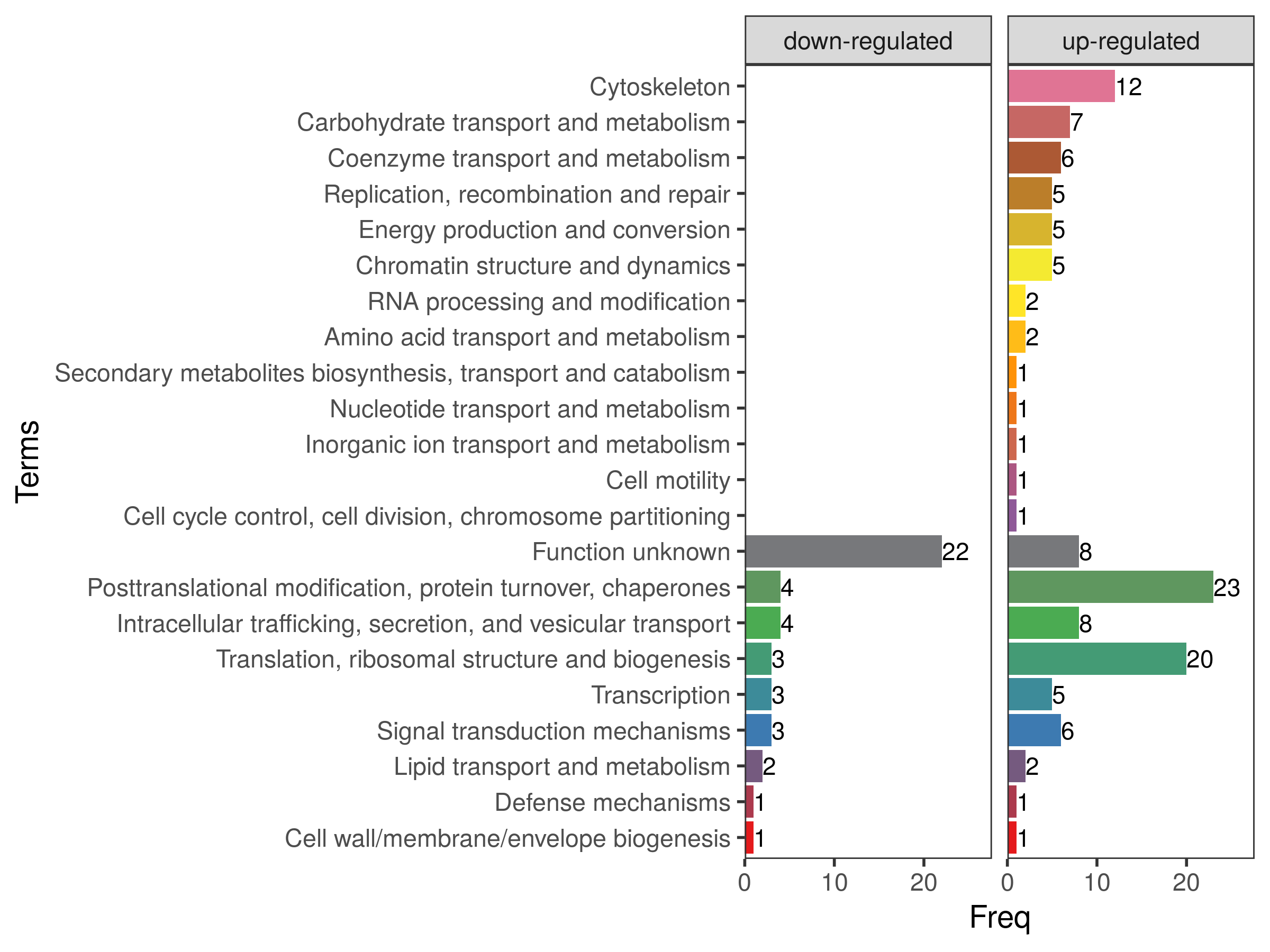

Supplement: Supplementary file 1 [file ijms-27-06236-s001.zip › Supplementary Materials/ijms-4276706_Proteomics_Dataset/4-Functional_classification/Figure 3b. KOG classify of Model-vs-Paeoniflorin.png]

Terms

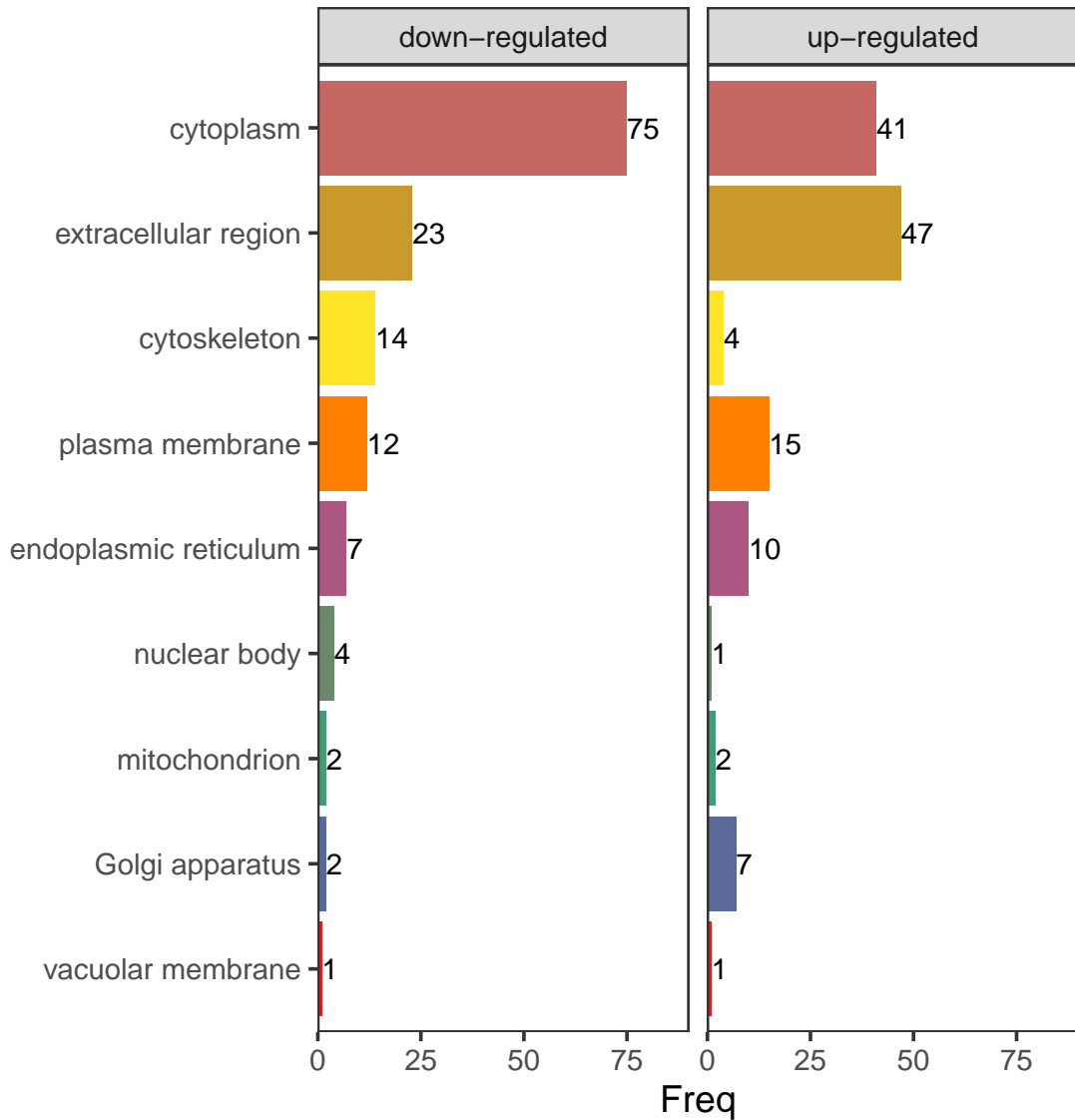

Supplement: Supplementary file 1 [file ijms-27-06236-s001.zip › Supplementary Materials/ijms-4276706_Proteomics_Dataset/4-Functional_classification/Figure 3c. Subcell classify of Control-vs-Model.pdf]

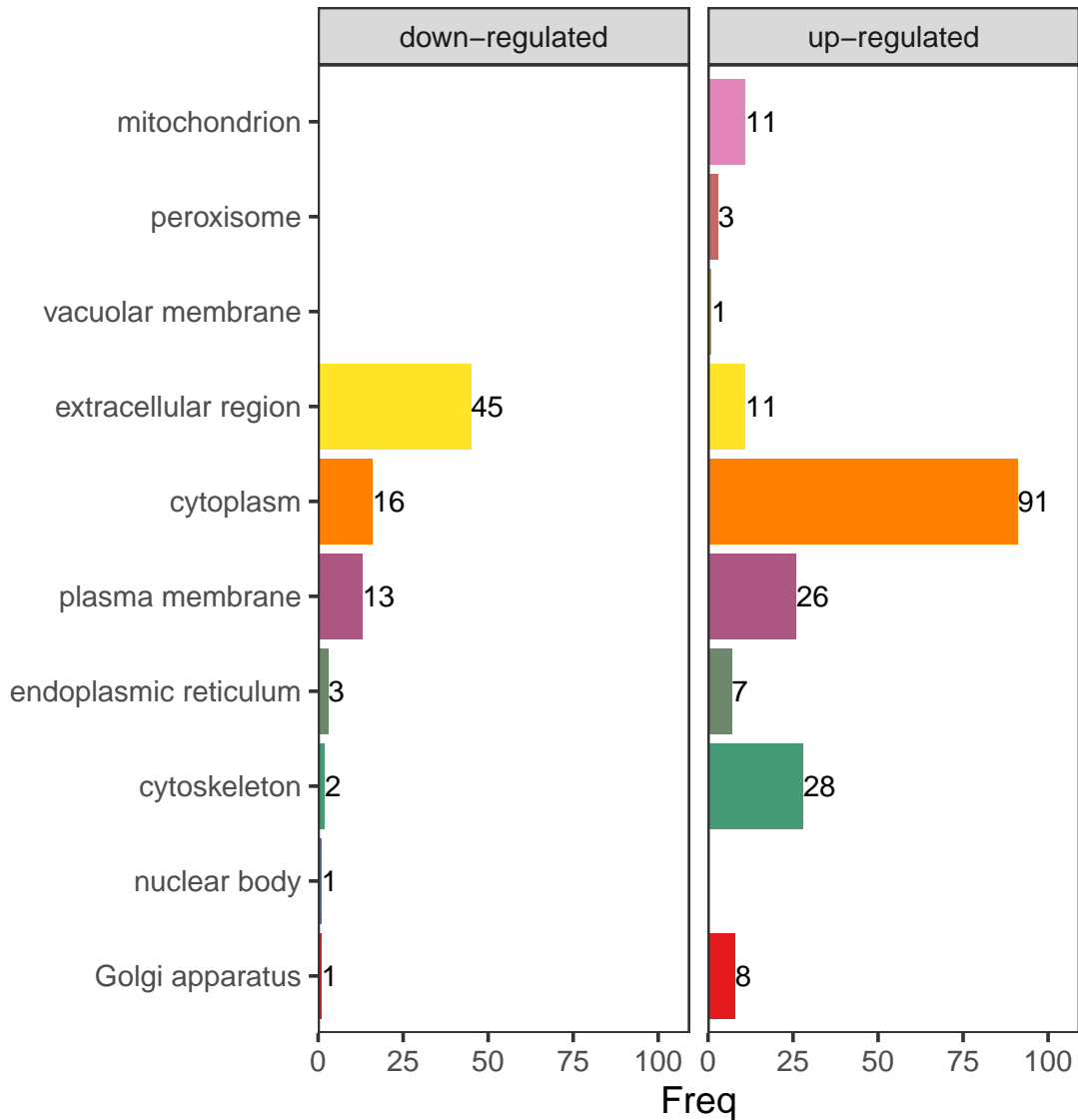

Supplement: Supplementary file 1 [file ijms-27-06236-s001.zip › Supplementary Materials/ijms-4276706_Proteomics_Dataset/4-Functional_classification/Figure 3c. Subcell classify of Model-vs-Paeoniflorin.pdf]

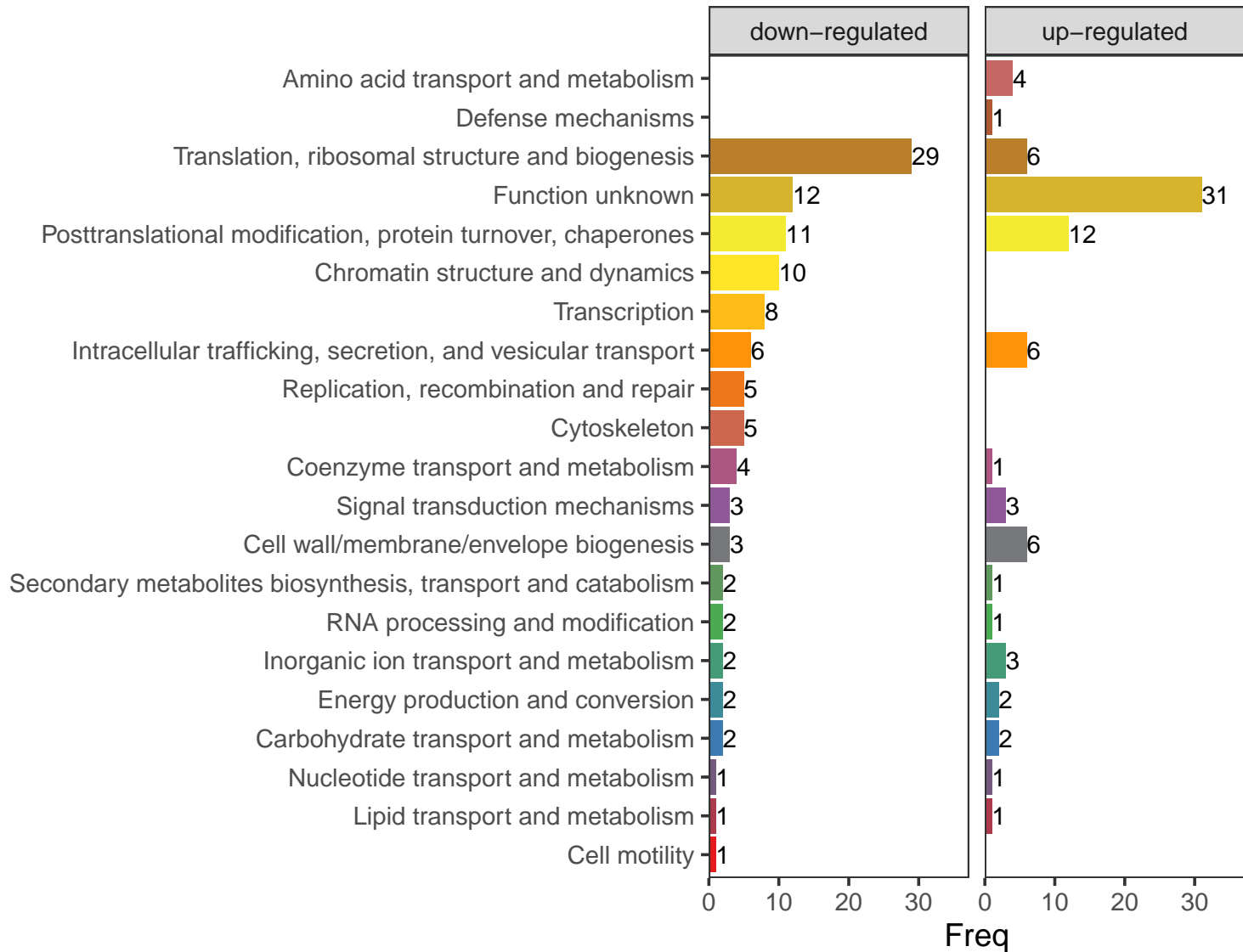

Supplement: Supplementary file 1 [file ijms-27-06236-s001.zip › Supplementary Materials/ijms-4276706_Proteomics_Dataset/4-Functional_classification/Figure 3b. KOG classify of Control-vs-Model.pdf]

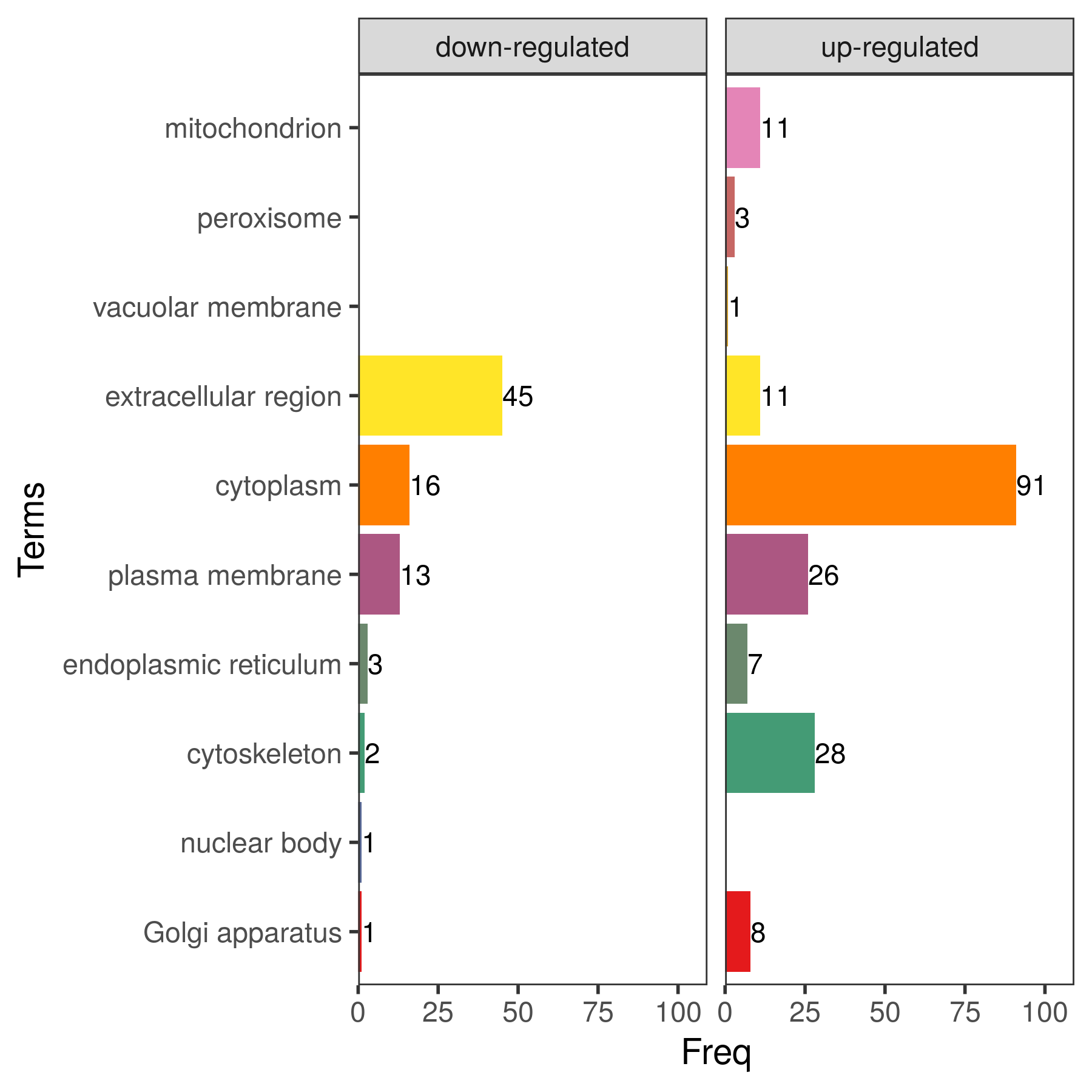

Supplement: Supplementary file 1 [file ijms-27-06236-s001.zip › Supplementary Materials/ijms-4276706_Proteomics_Dataset/4-Functional_classification/Figure 3c. Subcell classify of Model-vs-Paeoniflorin.png]

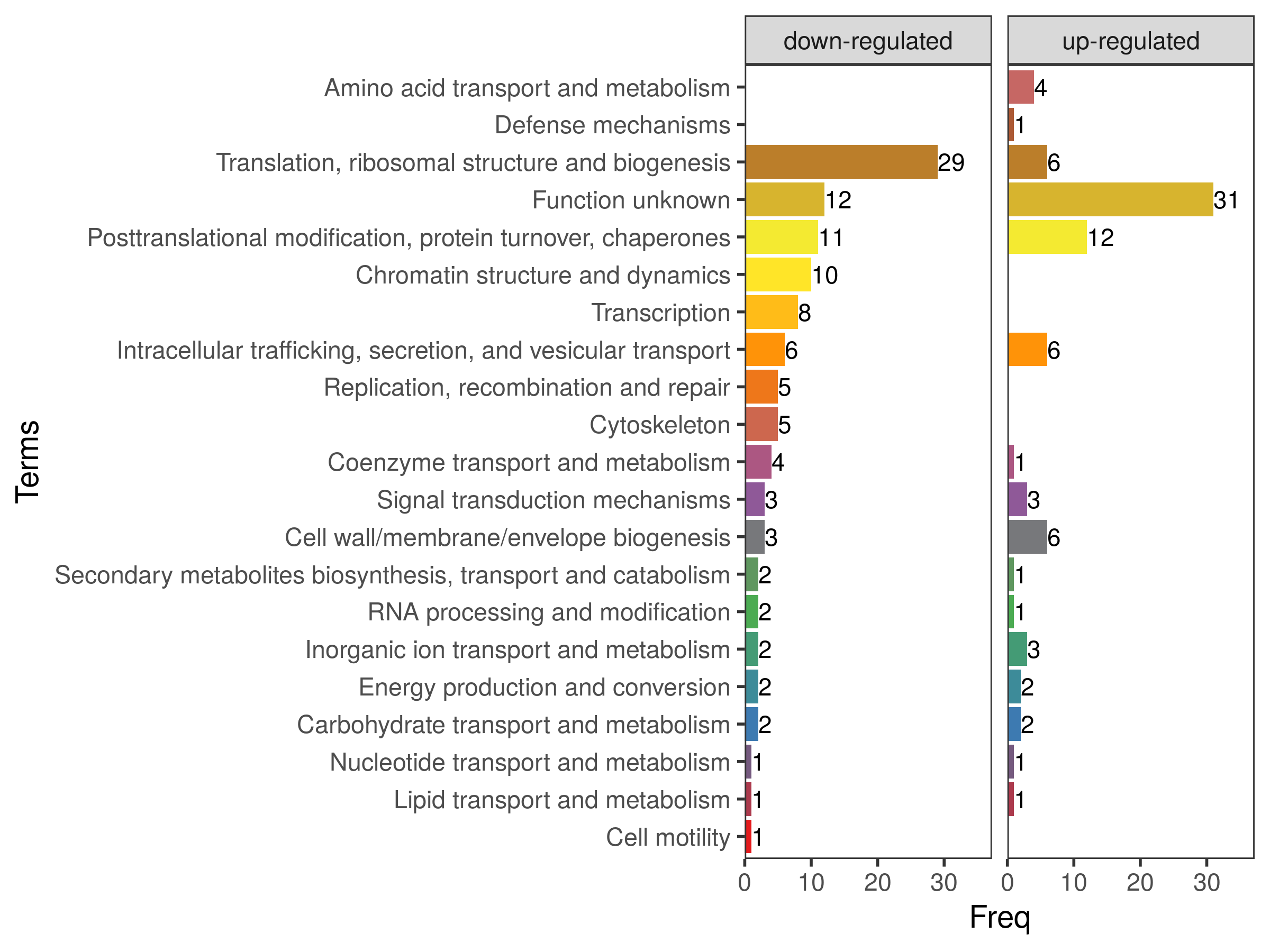

Supplement: Supplementary file 1 [file ijms-27-06236-s001.zip › Supplementary Materials/ijms-4276706_Proteomics_Dataset/4-Functional_classification/Figure 3b. KOG classify of Control-vs-Model.png]

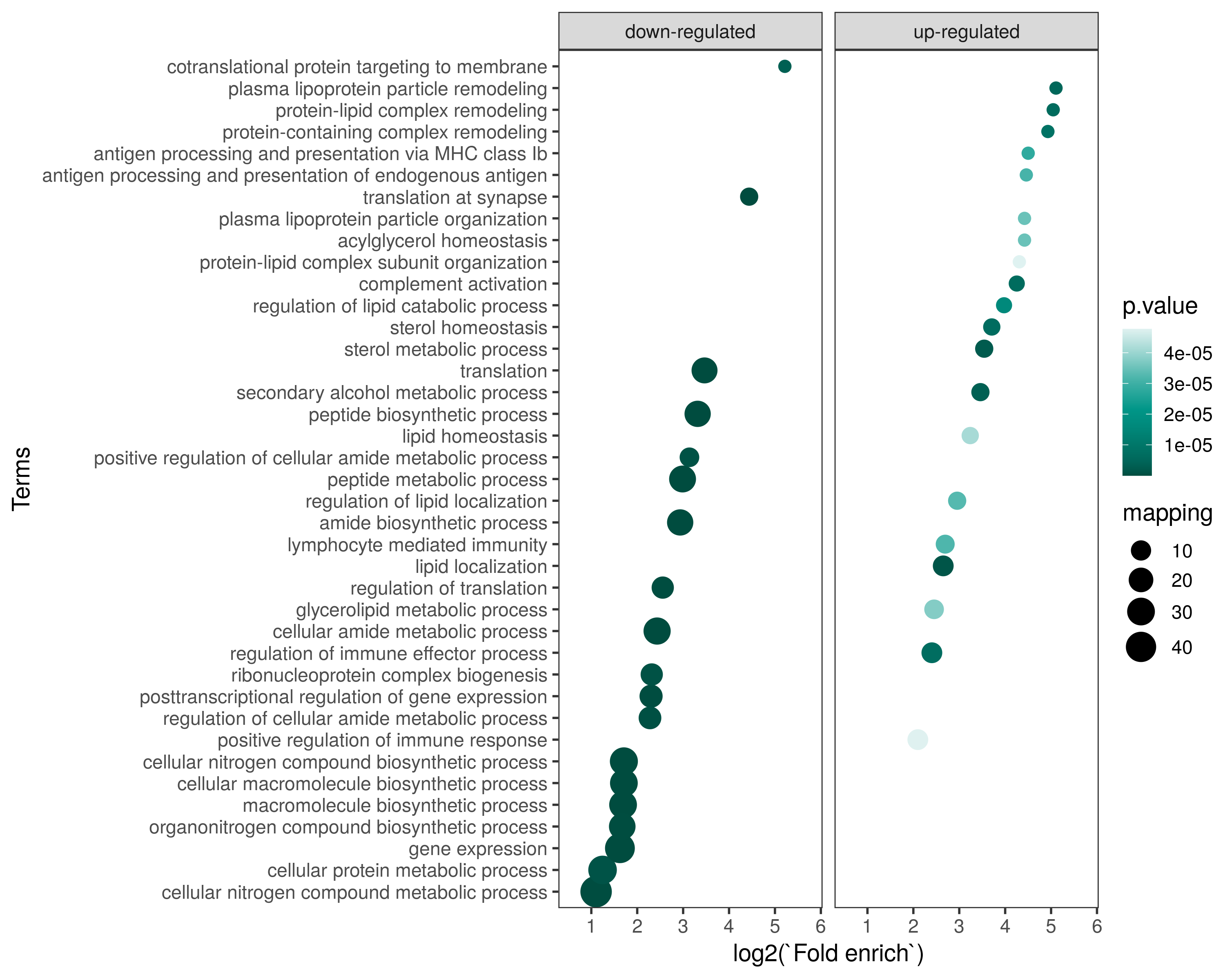

Supplement: Supplementary file 1 [file ijms-27-06236-s001.zip › Supplementary Materials/ijms-4276706_Proteomics_Dataset/5-Functional_enrichment/Figure 4. Biological process enrichment of Control-vs-Model.png]

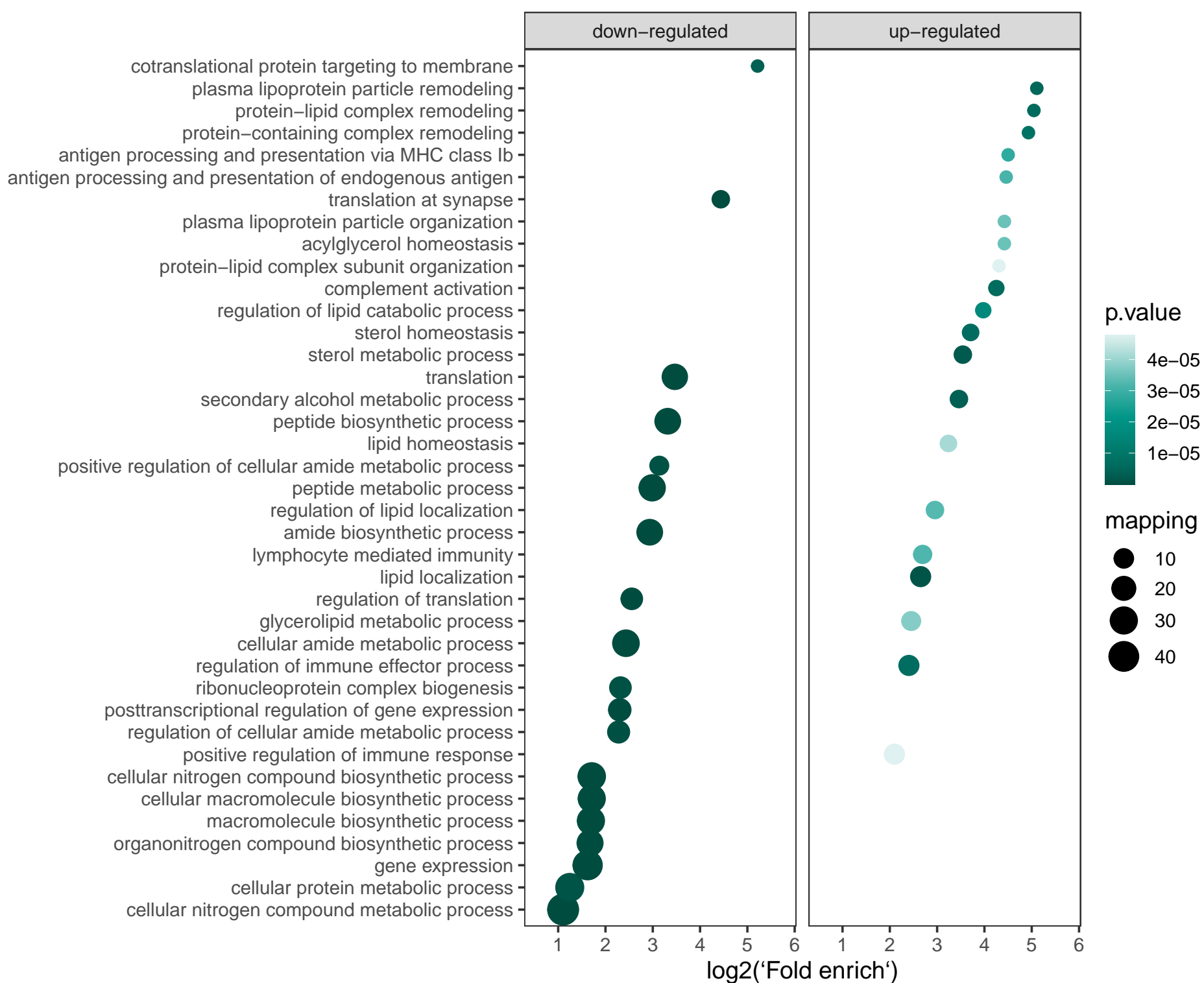

Supplement: Supplementary file 1 [file ijms-27-06236-s001.zip › Supplementary Materials/ijms-4276706_Proteomics_Dataset/5-Functional_enrichment/Figure 4. Biological process enrichment of Control-vs-Model.pdf]

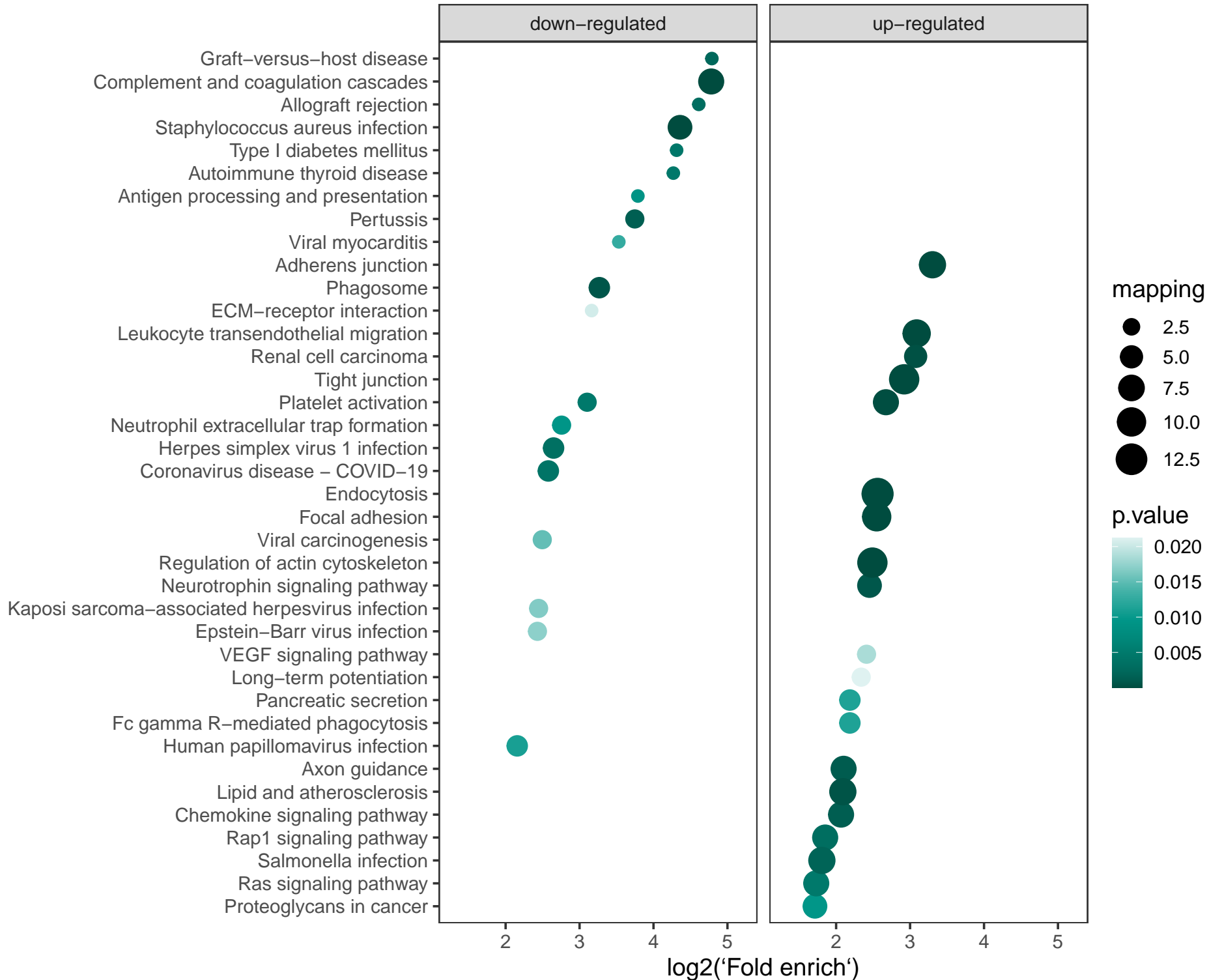

Supplement: Supplementary file 1 [file ijms-27-06236-s001.zip › Supplementary Materials/ijms-4276706_Proteomics_Dataset/5-Functional_enrichment/Figure 4. KEGG enrichment of Model-vs-Paeoniflorin.pdf]

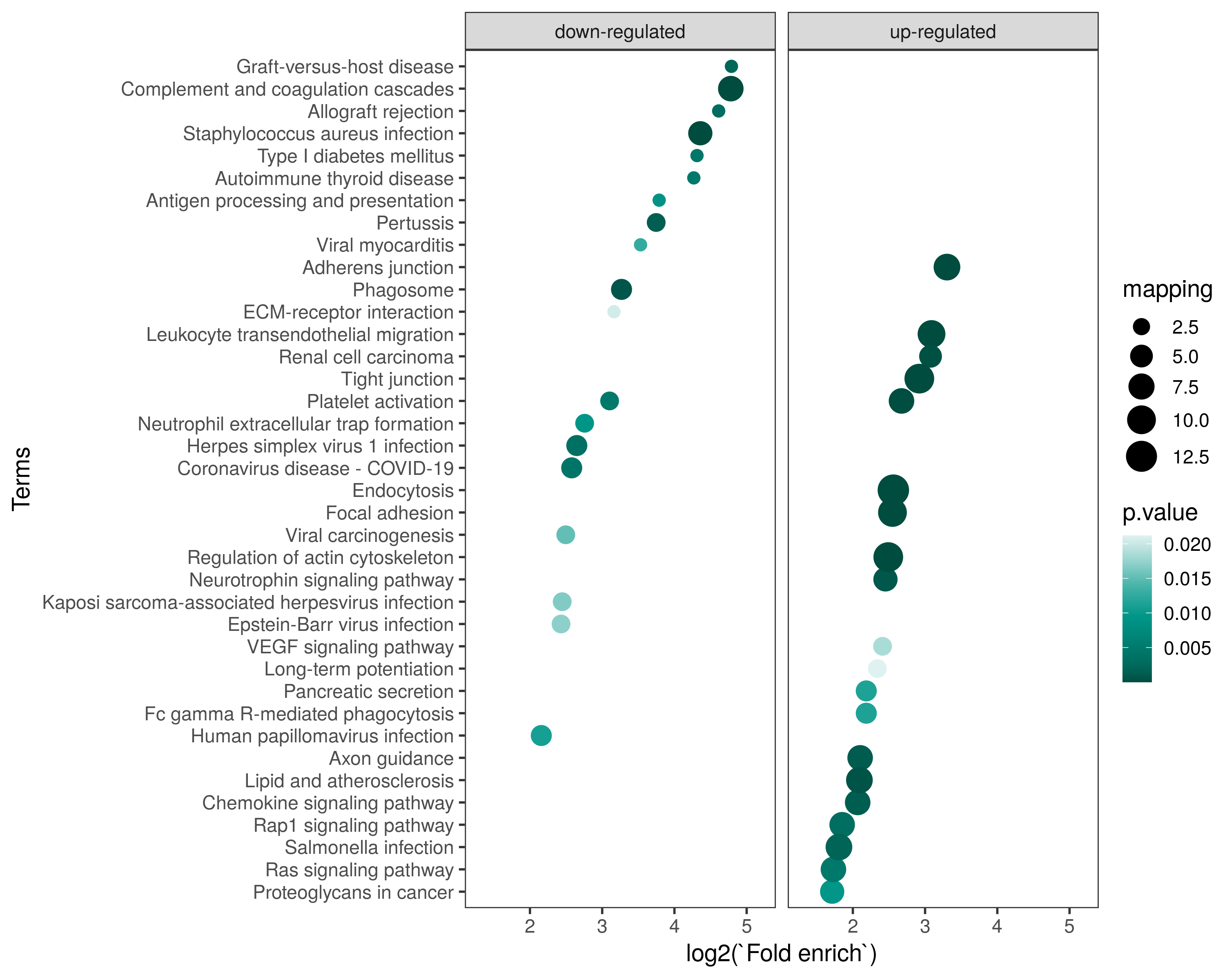

Supplement: Supplementary file 1 [file ijms-27-06236-s001.zip › Supplementary Materials/ijms-4276706_Proteomics_Dataset/5-Functional_enrichment/Figure 4. KEGG enrichment of Model-vs-Paeoniflorin.png]

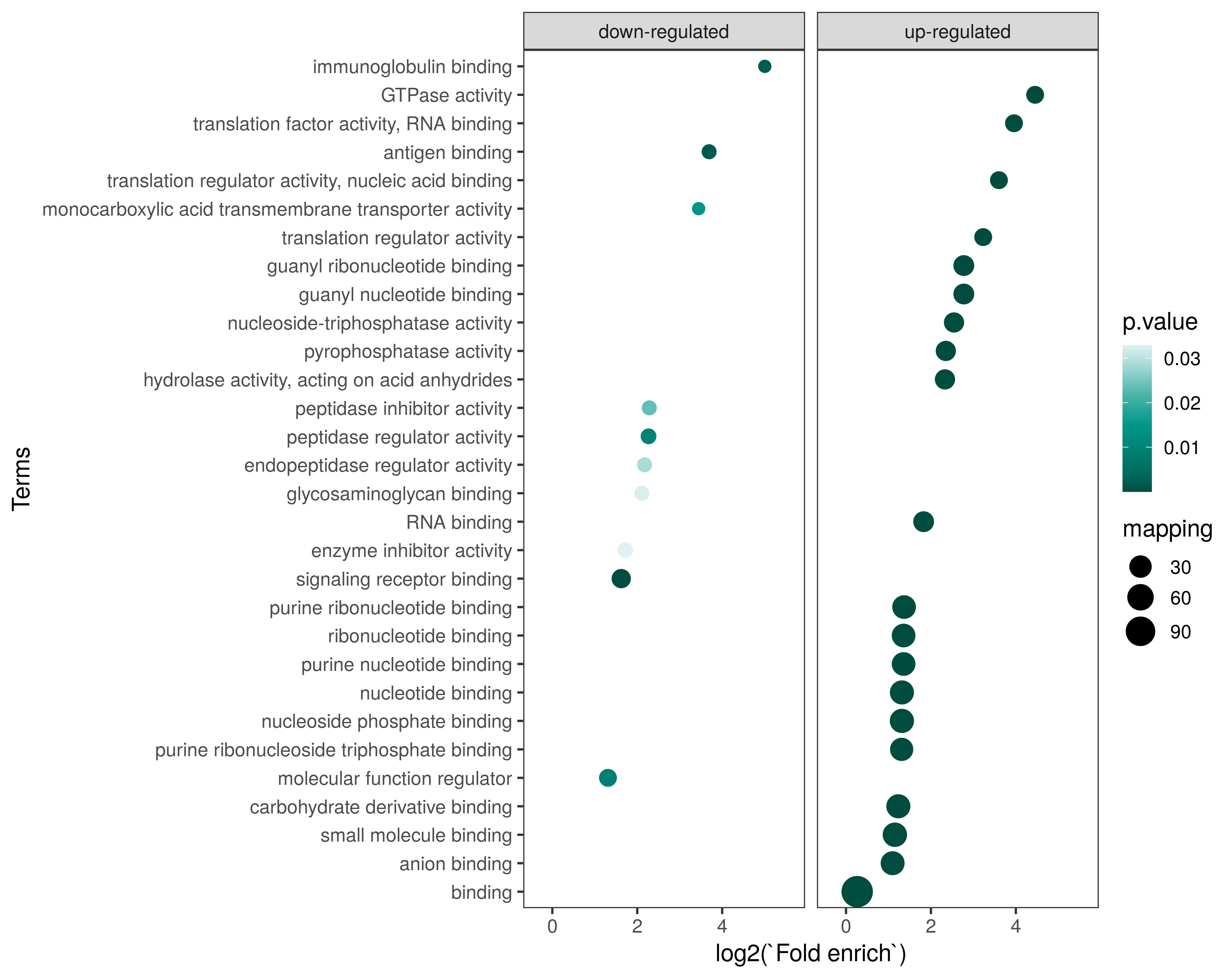

Supplement: Supplementary file 1 [file ijms-27-06236-s001.zip › Supplementary Materials/ijms-4276706_Proteomics_Dataset/5-Functional_enrichment/Figure 4. Molecular function enrichment of Model-vs-Paeoniflorin.png]

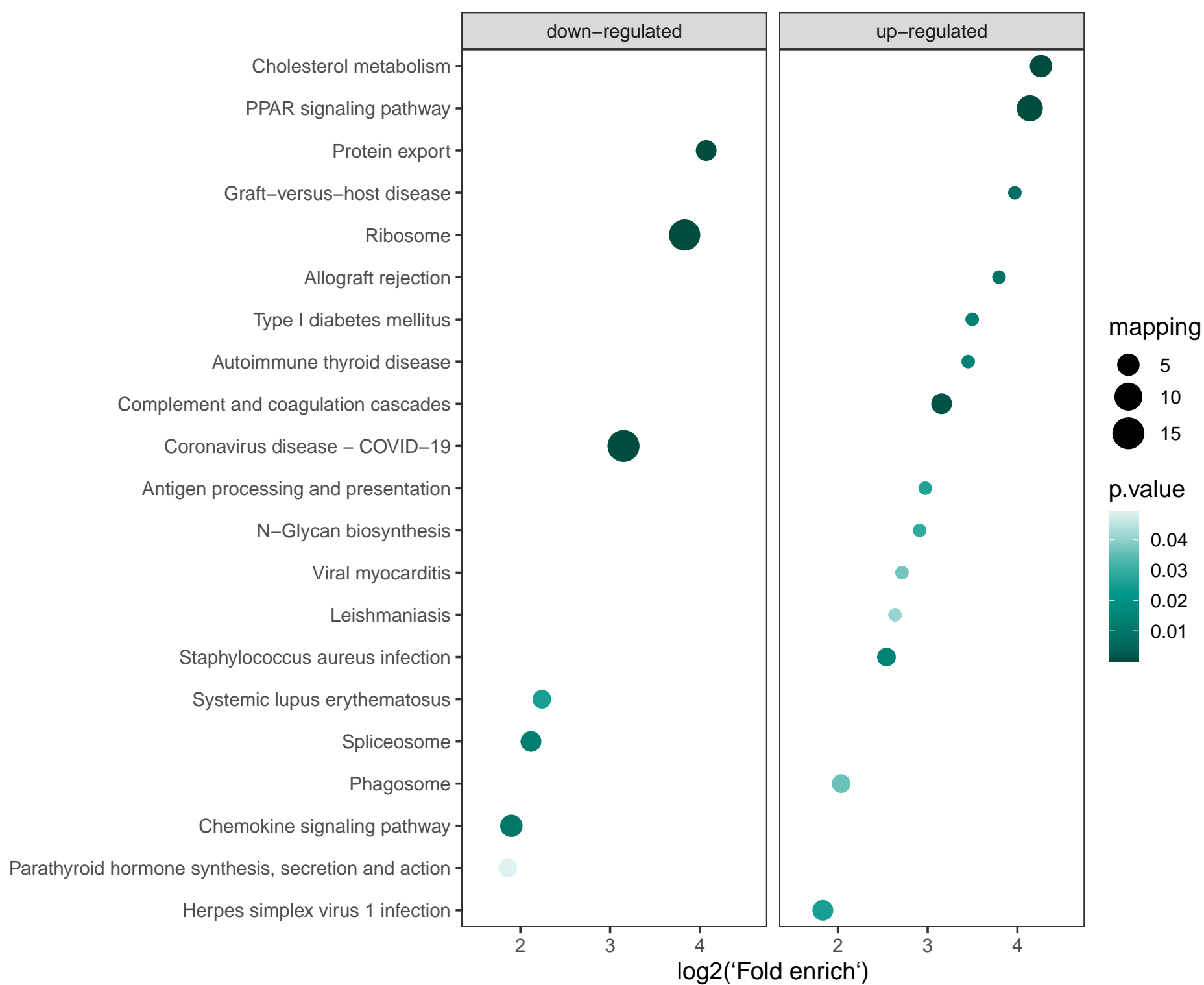

Supplement: Supplementary file 1 [file ijms-27-06236-s001.zip › Supplementary Materials/ijms-4276706_Proteomics_Dataset/5-Functional_enrichment/Figure 4. KEGG enrichment of Control-vs-Model.pdf]

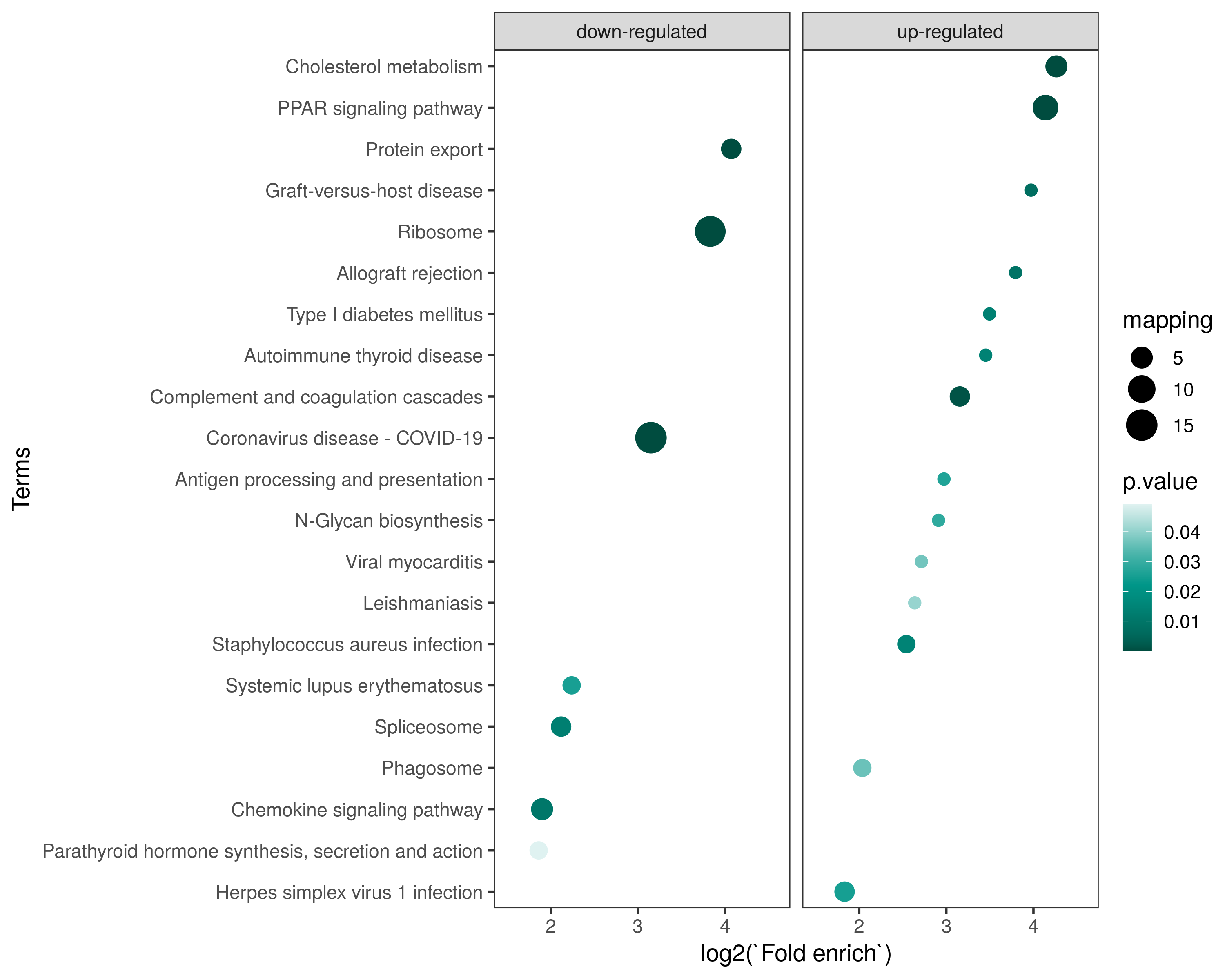

Supplement: Supplementary file 1 [file ijms-27-06236-s001.zip › Supplementary Materials/ijms-4276706_Proteomics_Dataset/5-Functional_enrichment/Figure 4. KEGG enrichment of Control-vs-Model.png]

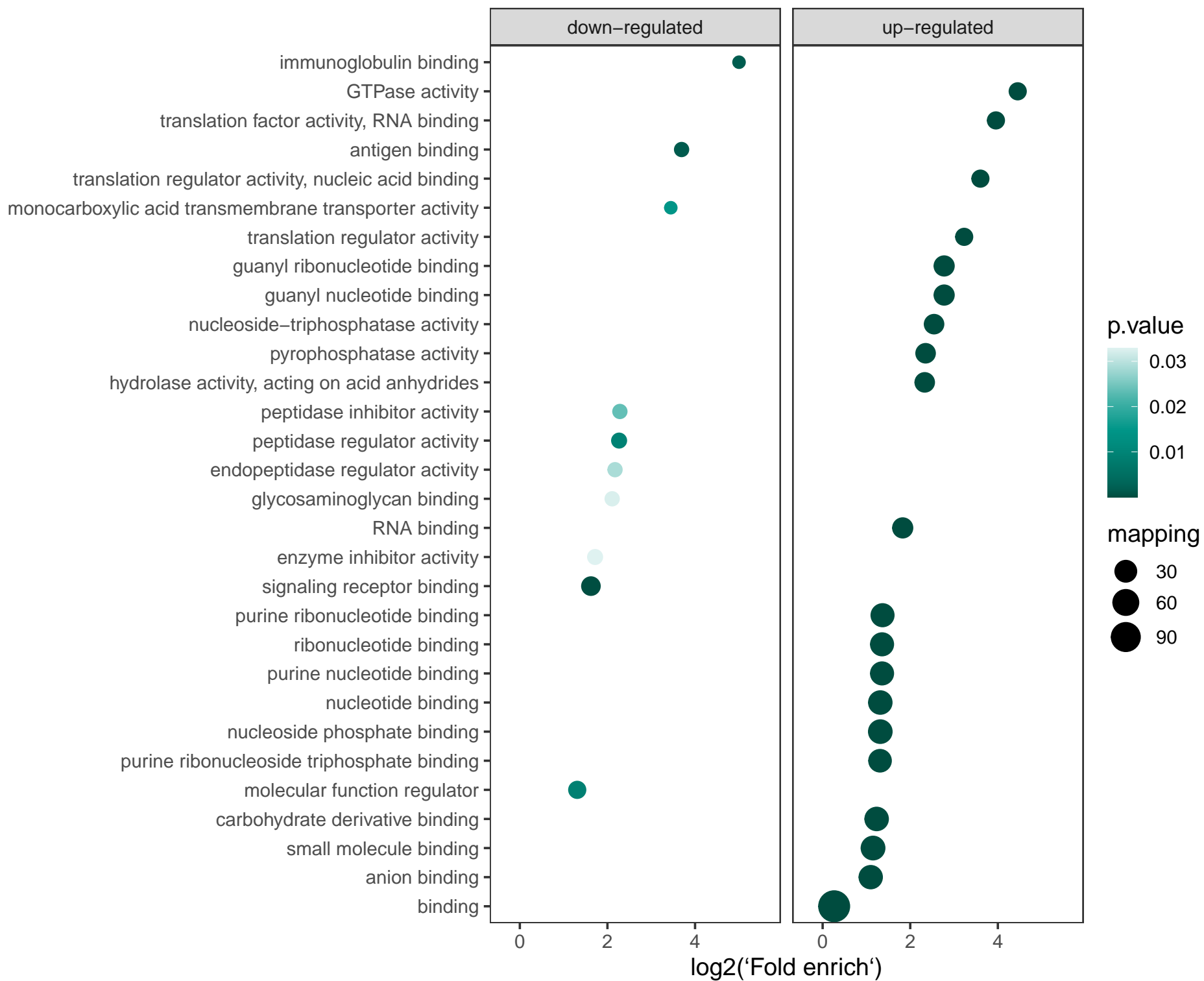

Supplement: Supplementary file 1 [file ijms-27-06236-s001.zip › Supplementary Materials/ijms-4276706_Proteomics_Dataset/5-Functional_enrichment/Figure 4. Molecular function enrichment of Model-vs-Paeoniflorin.pdf]

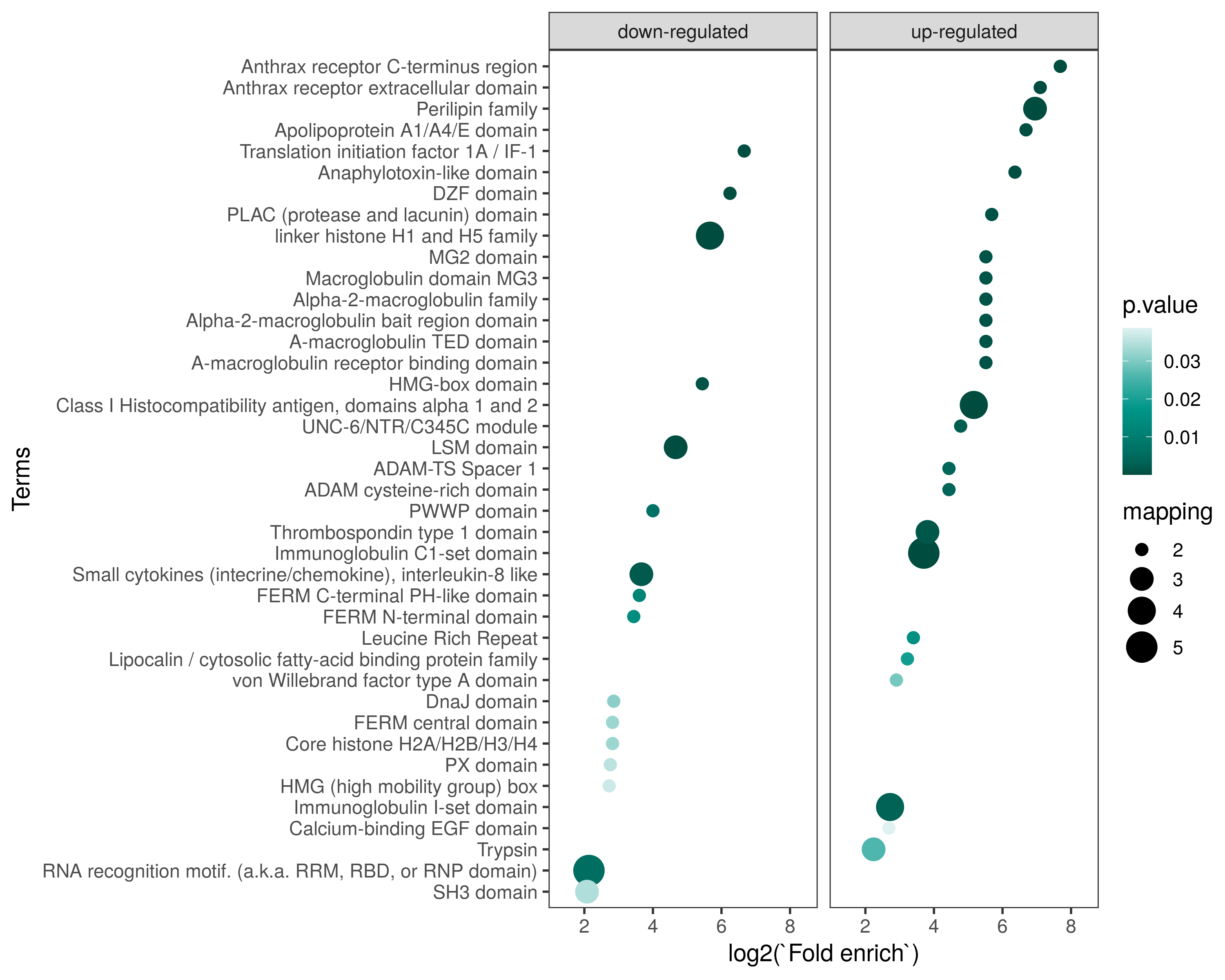

Supplement: Supplementary file 1 [file ijms-27-06236-s001.zip › Supplementary Materials/ijms-4276706_Proteomics_Dataset/5-Functional_enrichment/Figure 4. Pfam enrichment of Control-vs-Model.png]

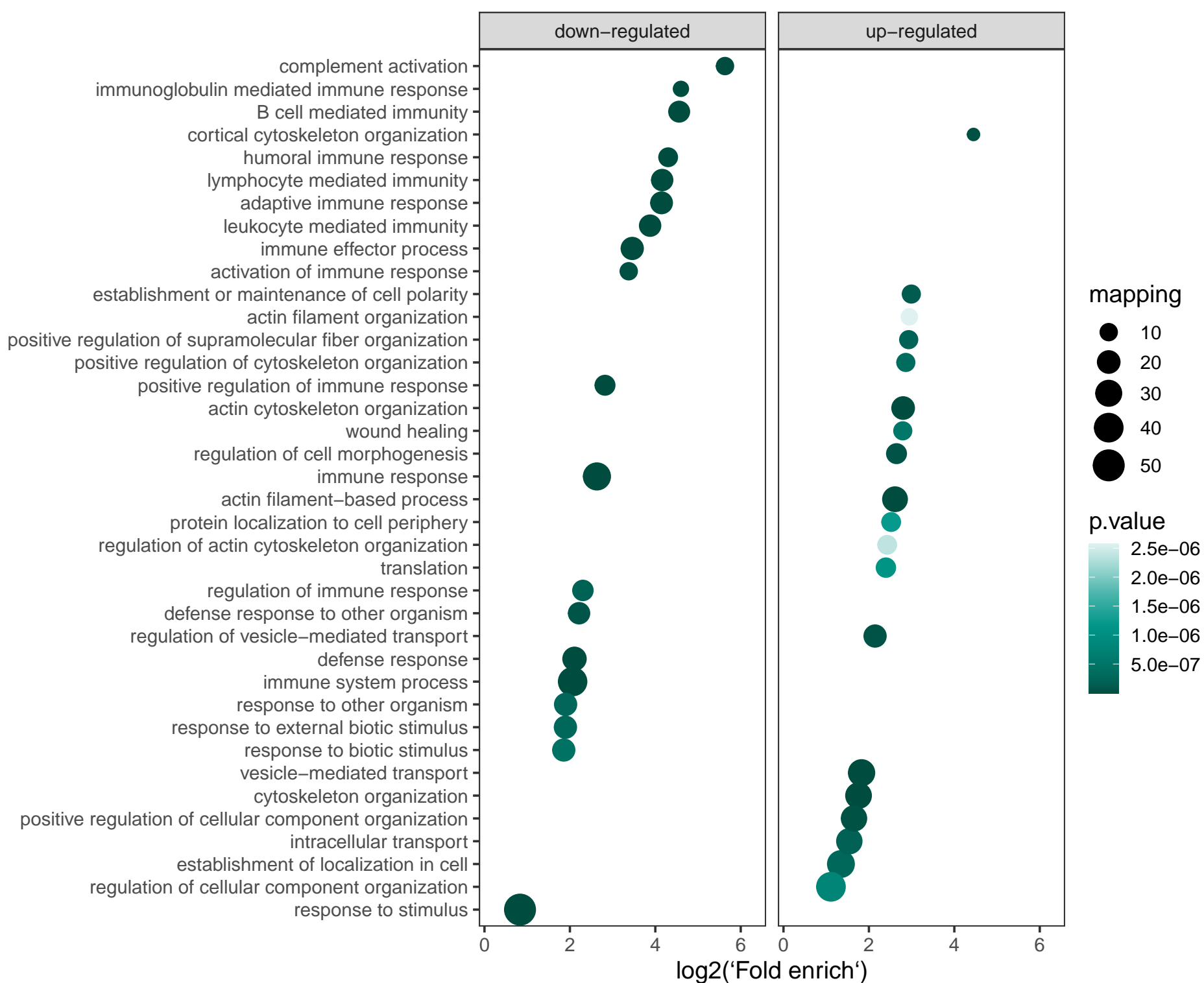

Supplement: Supplementary file 1 [file ijms-27-06236-s001.zip › Supplementary Materials/ijms-4276706_Proteomics_Dataset/5-Functional_enrichment/Figure 4. Biological process enrichment of Model-vs-Paeoniflorin.pdf]

Terms

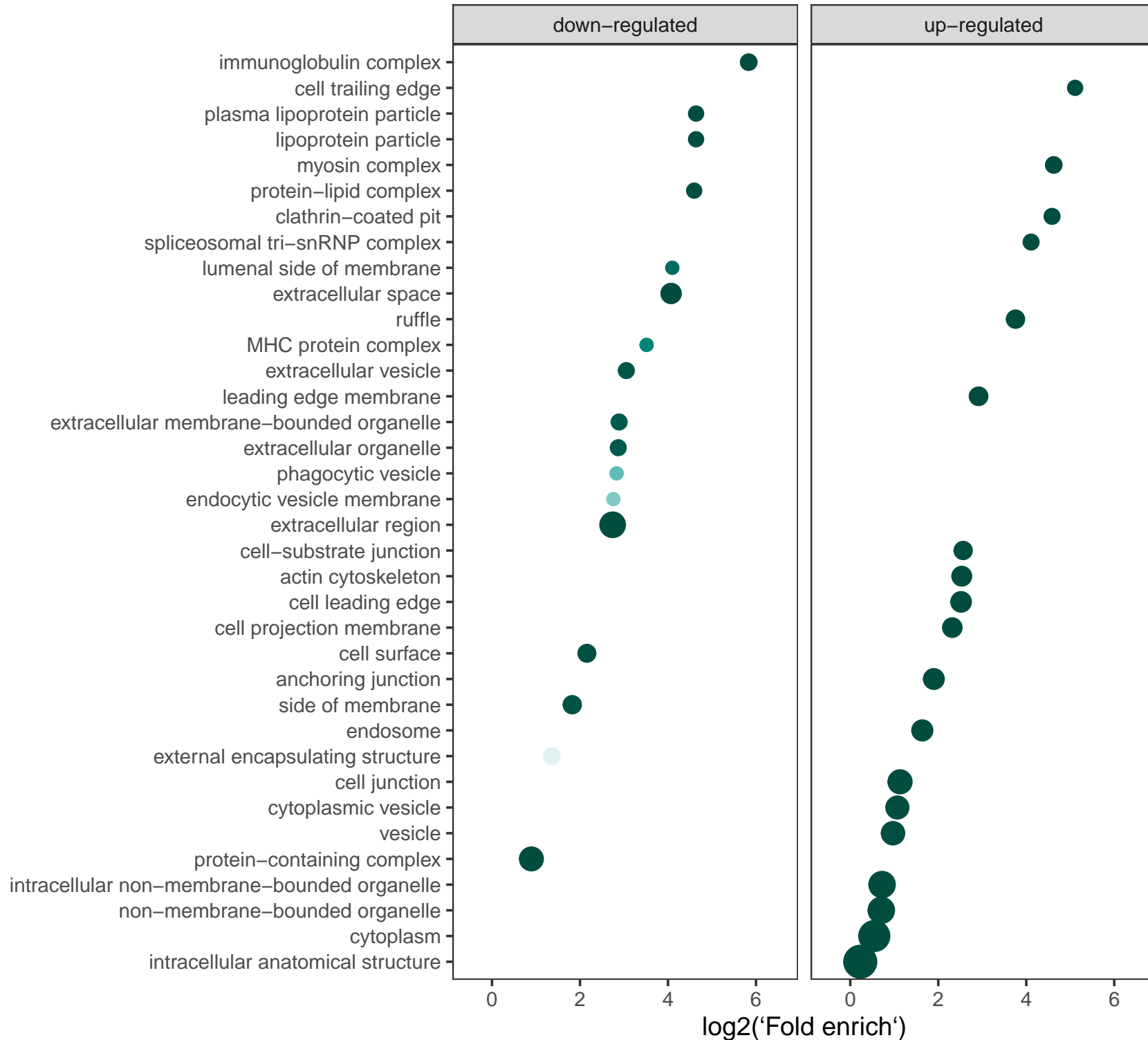

Supplement: Supplementary file 1 [file ijms-27-06236-s001.zip › Supplementary Materials/ijms-4276706_Proteomics_Dataset/5-Functional_enrichment/Figure 4. Cellular component enrichment of Model-vs-Paeoniflorin.pdf]

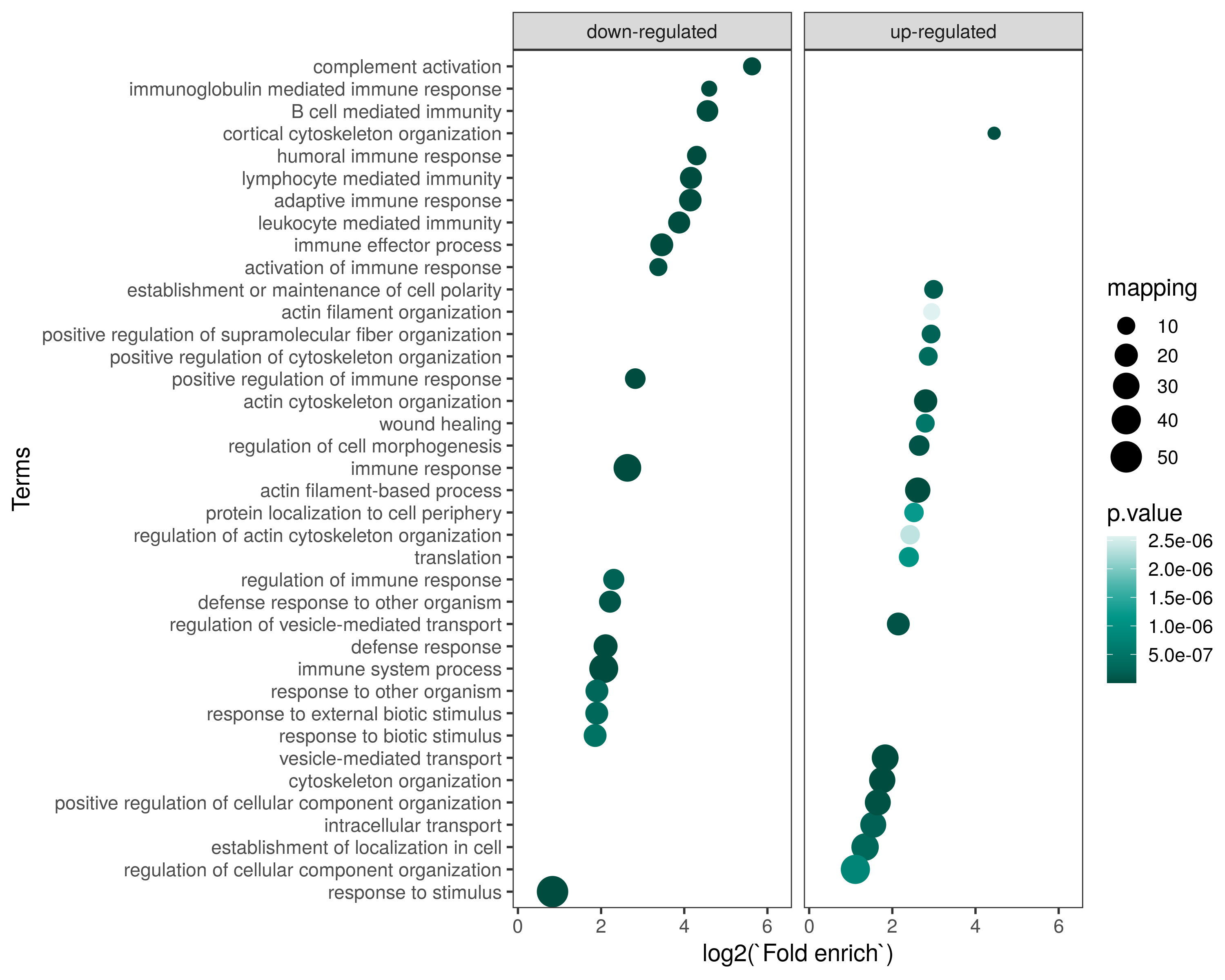

Supplement: Supplementary file 1 [file ijms-27-06236-s001.zip › Supplementary Materials/ijms-4276706_Proteomics_Dataset/5-Functional_enrichment/Figure 4. Biological process enrichment of Model-vs-Paeoniflorin.png]

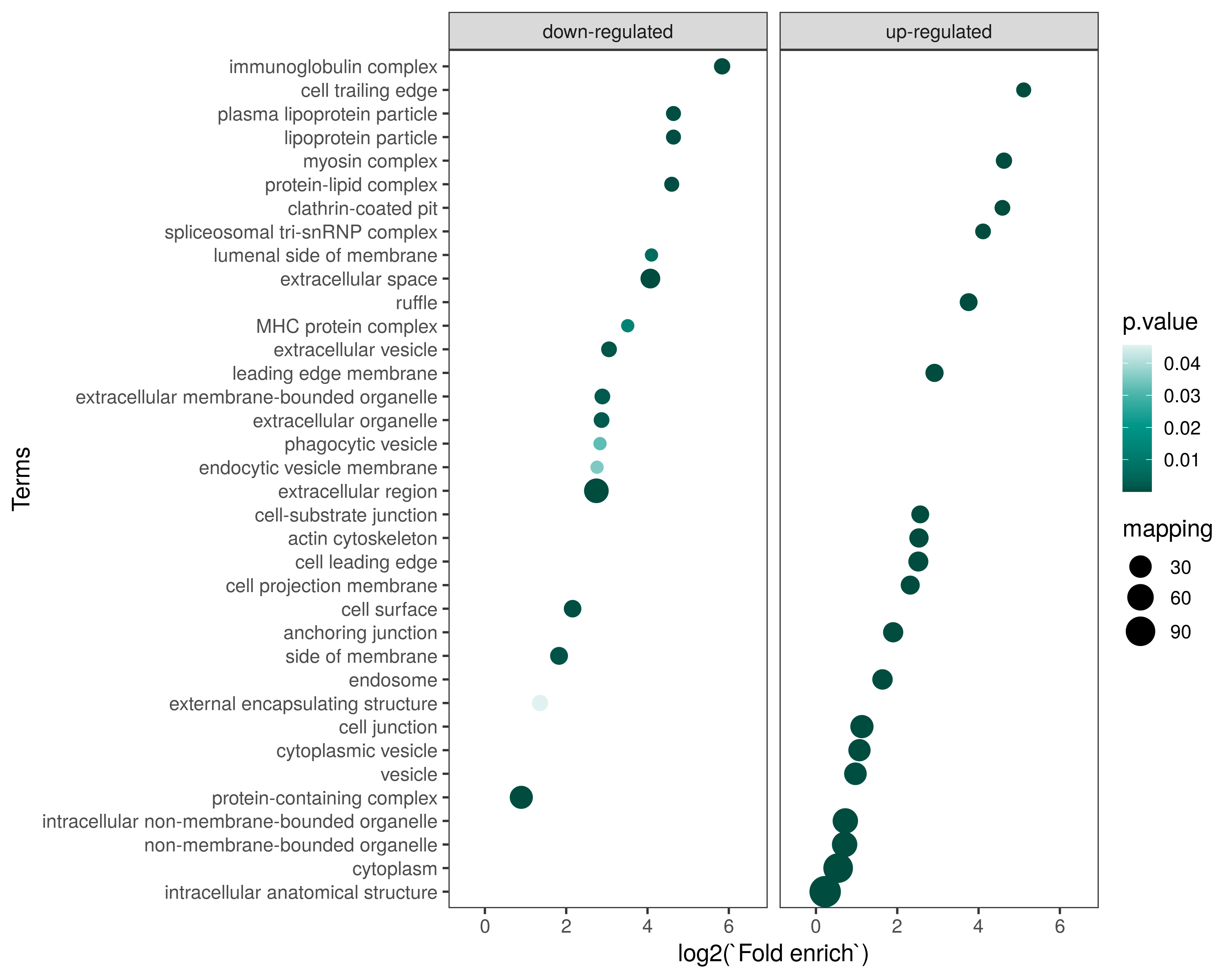

Supplement: Supplementary file 1 [file ijms-27-06236-s001.zip › Supplementary Materials/ijms-4276706_Proteomics_Dataset/5-Functional_enrichment/Figure 4. Cellular component enrichment of Model-vs-Paeoniflorin.png]

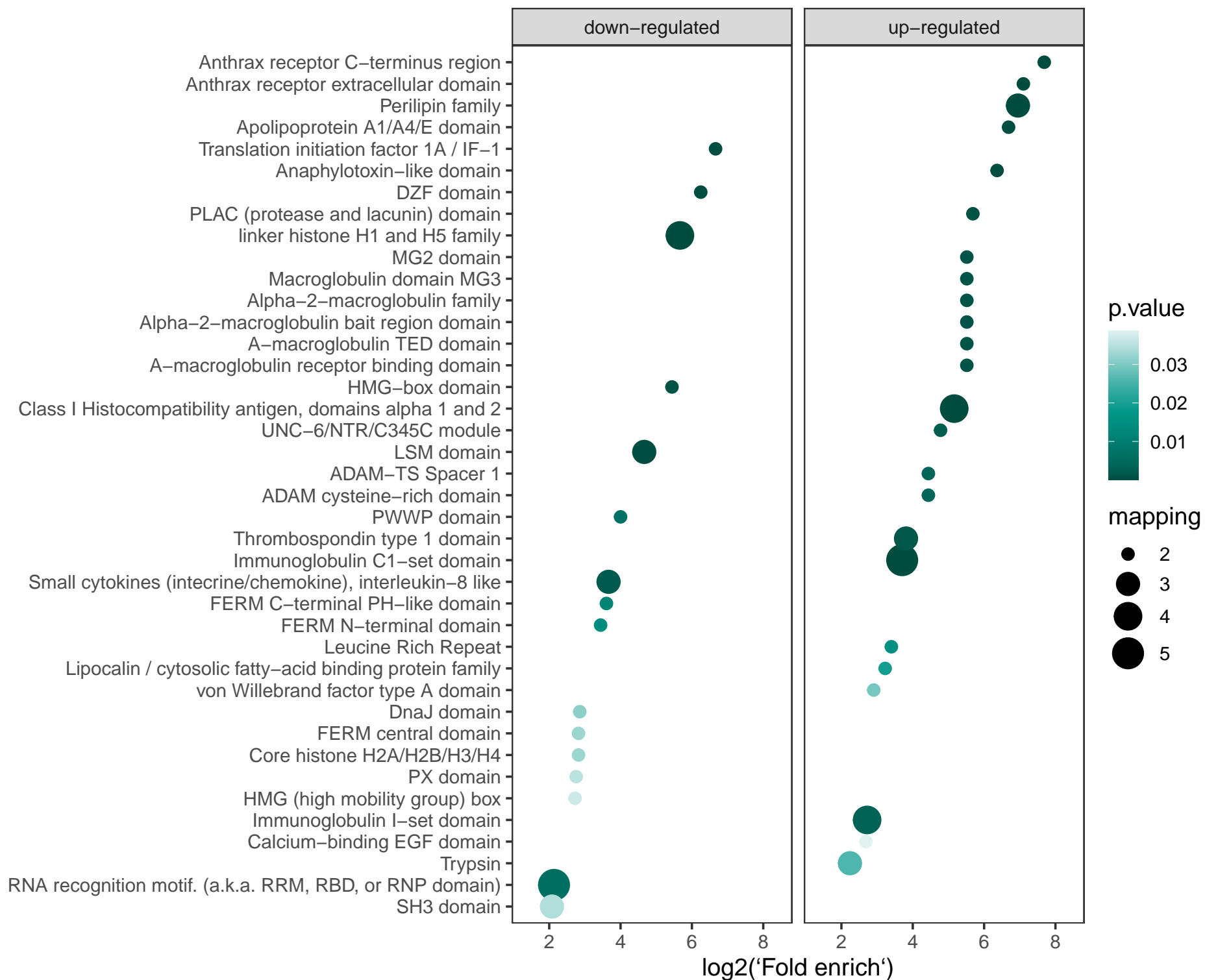

Supplement: Supplementary file 1 [file ijms-27-06236-s001.zip › Supplementary Materials/ijms-4276706_Proteomics_Dataset/5-Functional_enrichment/Figure 4. Pfam enrichment of Control-vs-Model.pdf]

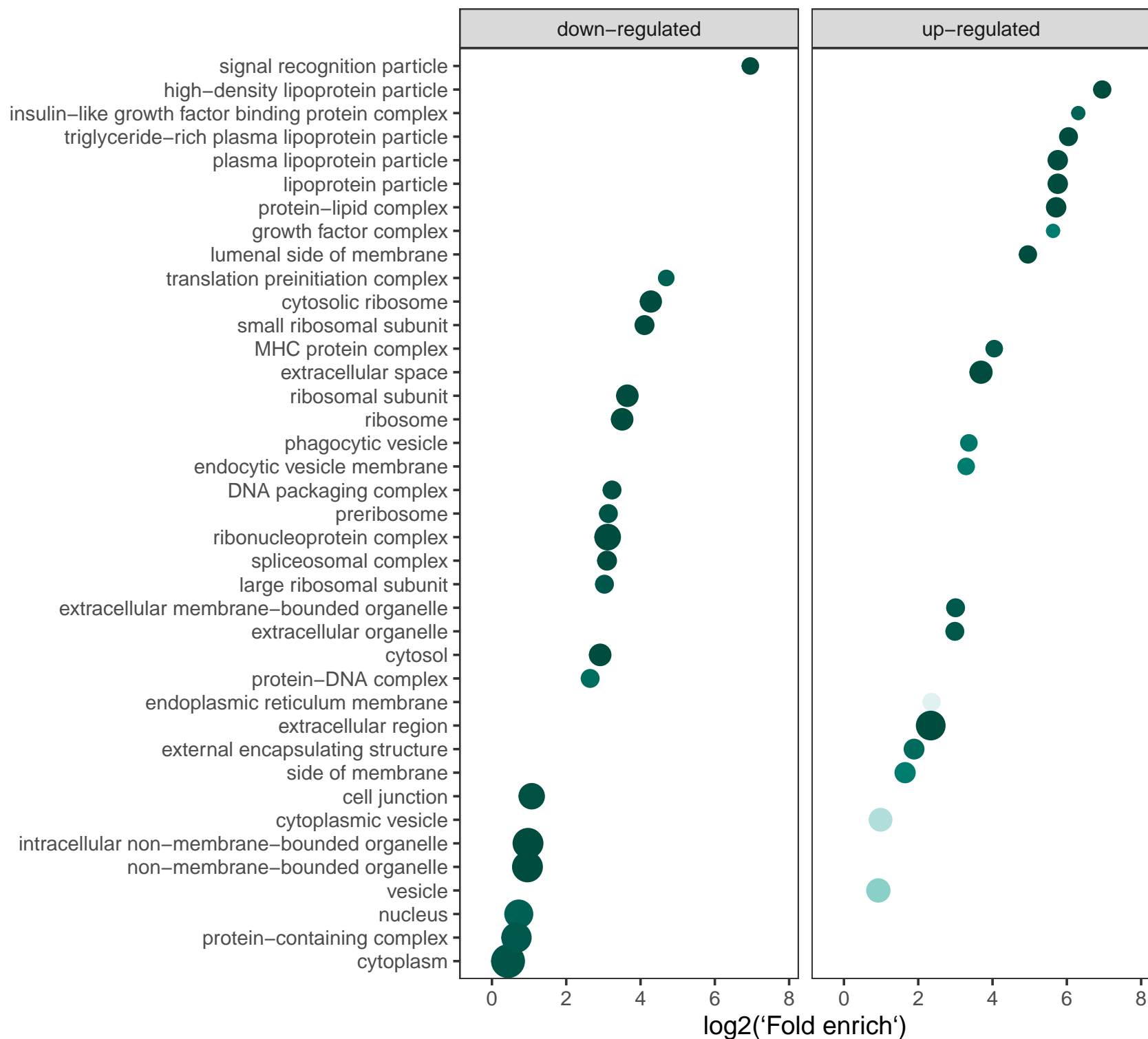

Supplement: Supplementary file 1 [file ijms-27-06236-s001.zip › Supplementary Materials/ijms-4276706_Proteomics_Dataset/5-Functional_enrichment/Figure 4. Cellular component enrichment of Control-vs-Model.pdf]

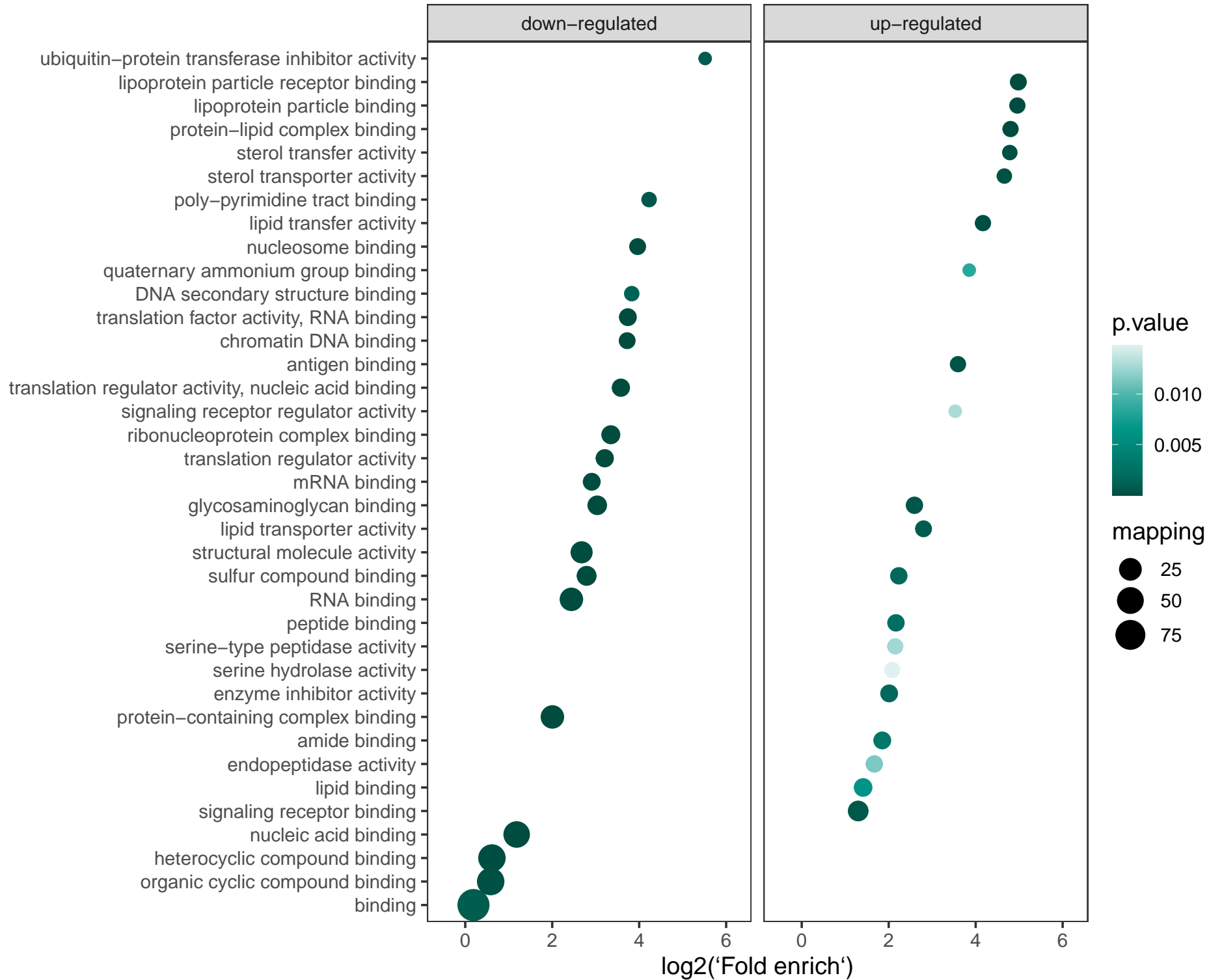

Supplement: Supplementary file 1 [file ijms-27-06236-s001.zip › Supplementary Materials/ijms-4276706_Proteomics_Dataset/5-Functional_enrichment/Figure 4. Molecular function enrichment of Control-vs-Model.pdf]

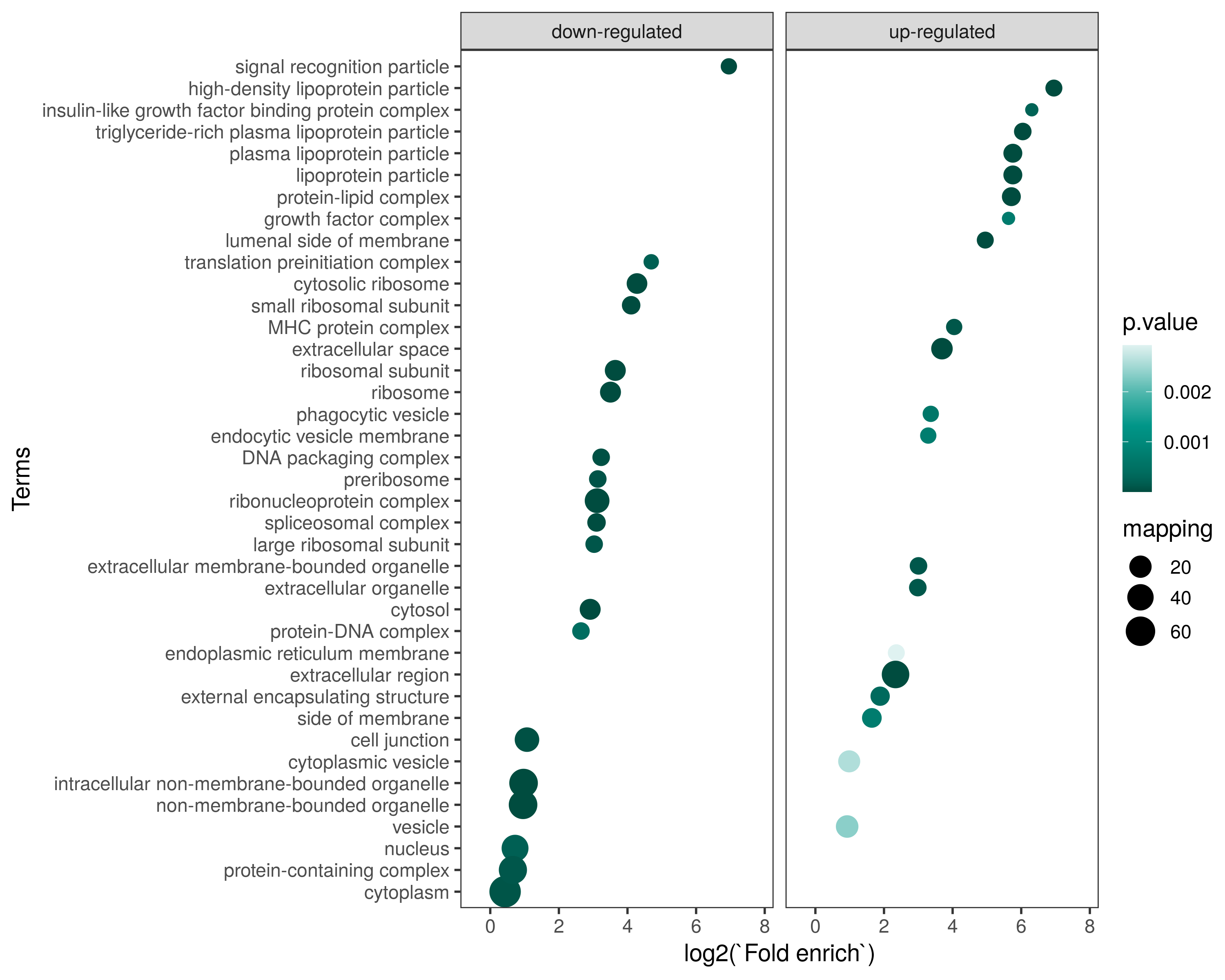

Supplement: Supplementary file 1 [file ijms-27-06236-s001.zip › Supplementary Materials/ijms-4276706_Proteomics_Dataset/5-Functional_enrichment/Figure 4. Cellular component enrichment of Control-vs-Model.png]

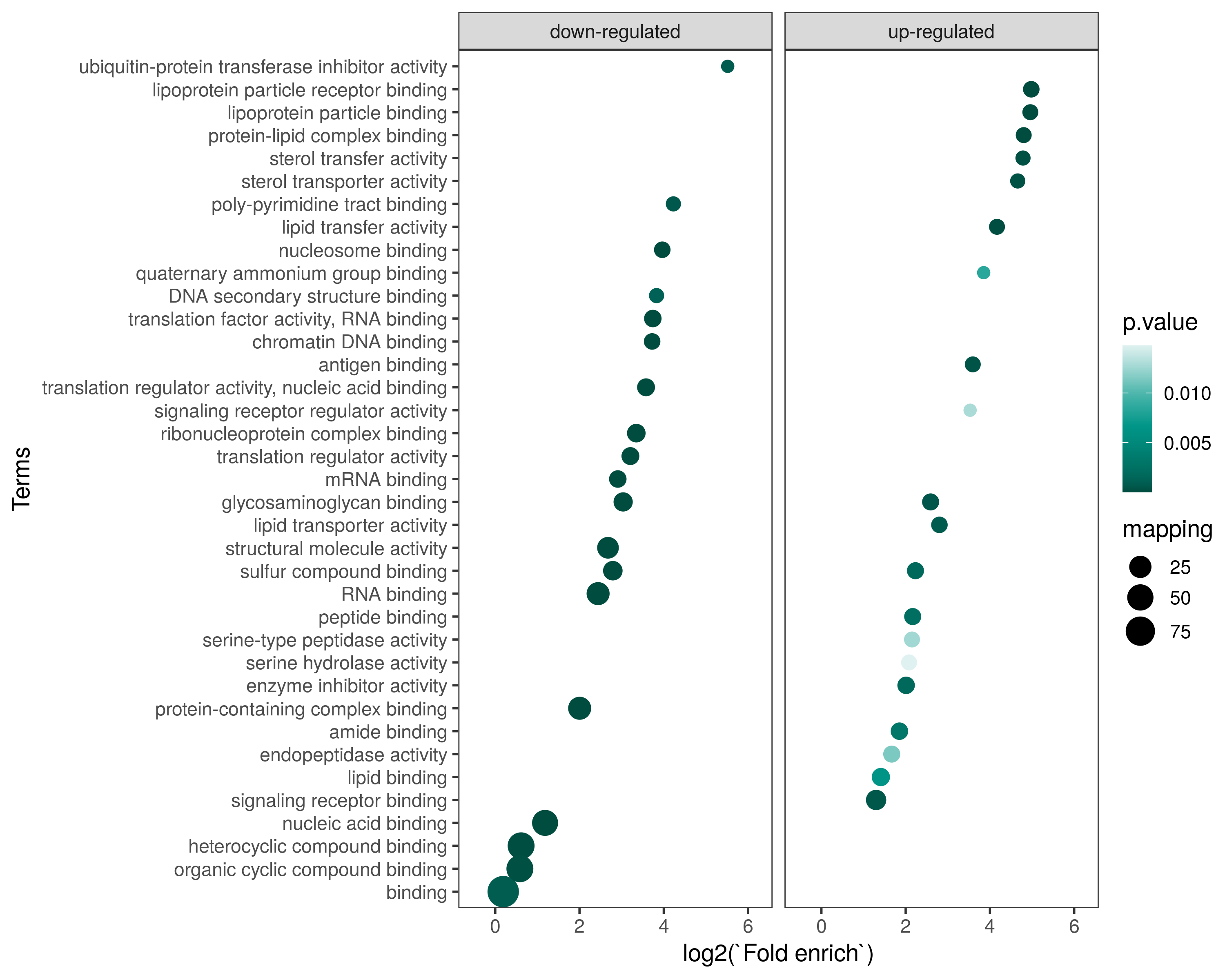

Supplement: Supplementary file 1 [file ijms-27-06236-s001.zip › Supplementary Materials/ijms-4276706_Proteomics_Dataset/5-Functional_enrichment/Figure 4. Molecular function enrichment of Control-vs-Model.png]

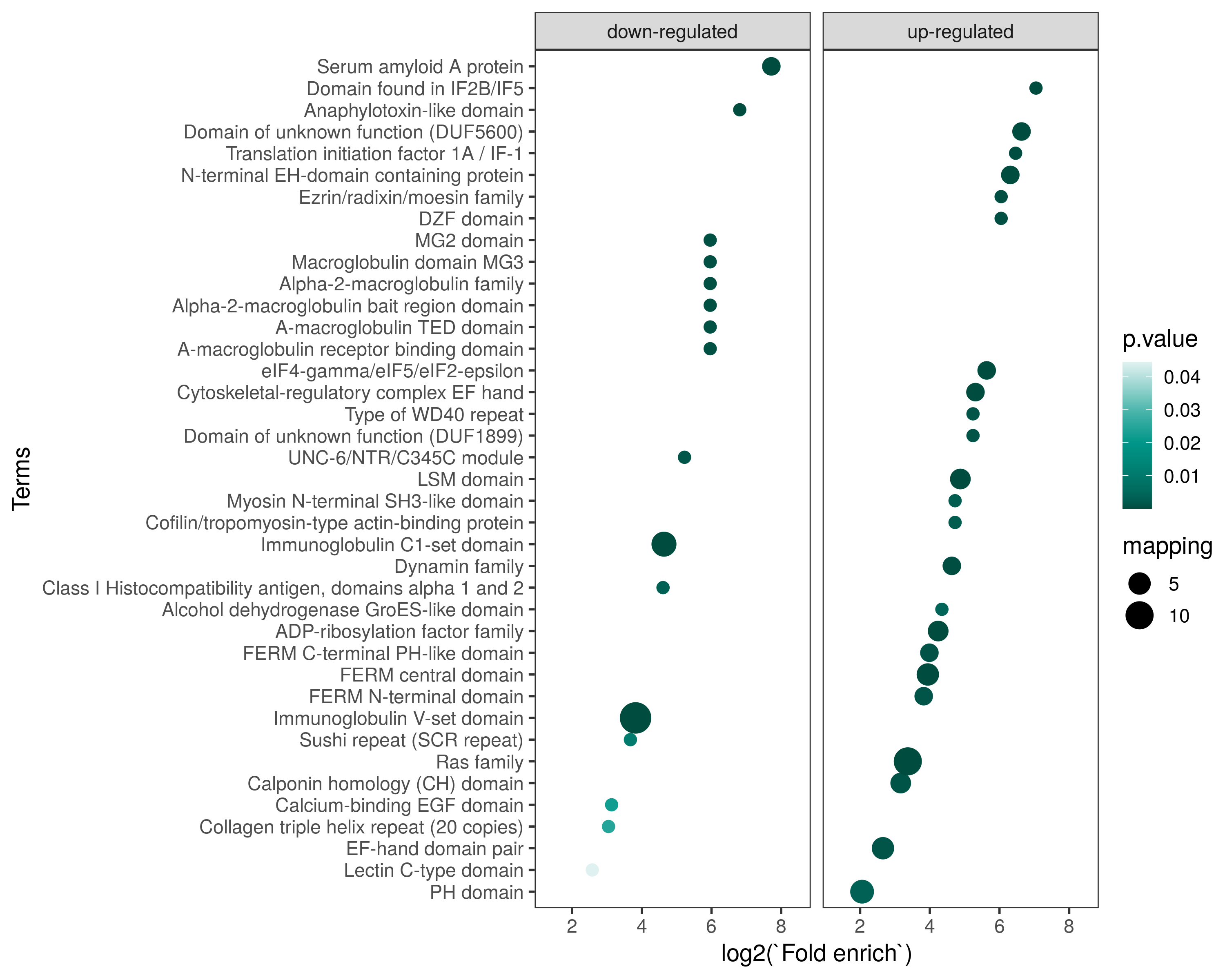

Supplement: Supplementary file 1 [file ijms-27-06236-s001.zip › Supplementary Materials/ijms-4276706_Proteomics_Dataset/5-Functional_enrichment/Figure 4. Pfam enrichment of Model-vs-Paeoniflorin.png]

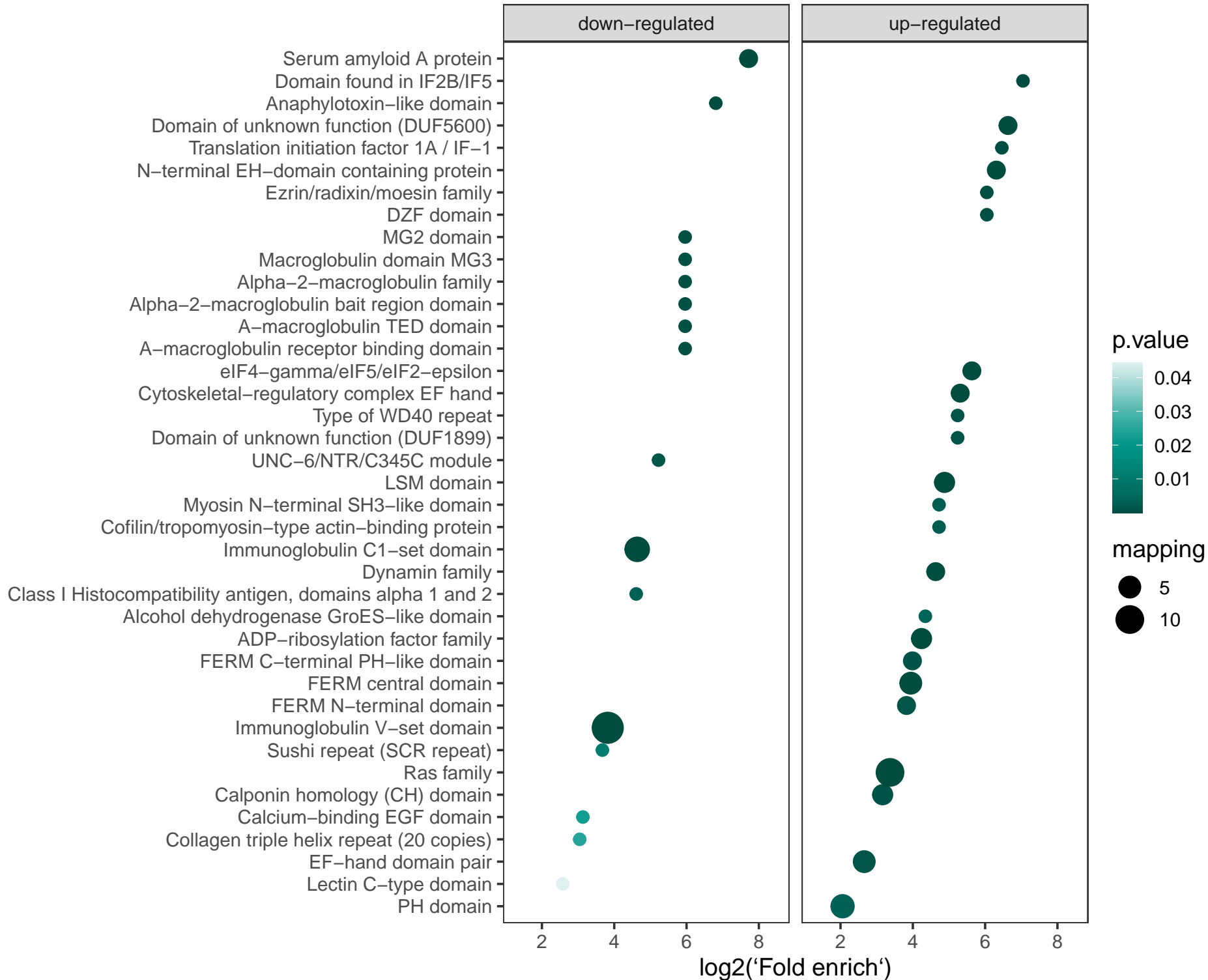

Supplement: Supplementary file 1 [file ijms-27-06236-s001.zip › Supplementary Materials/ijms-4276706_Proteomics_Dataset/5-Functional_enrichment/Figure 4. Pfam enrichment of Model-vs-Paeoniflorin.pdf]

# Cellular component

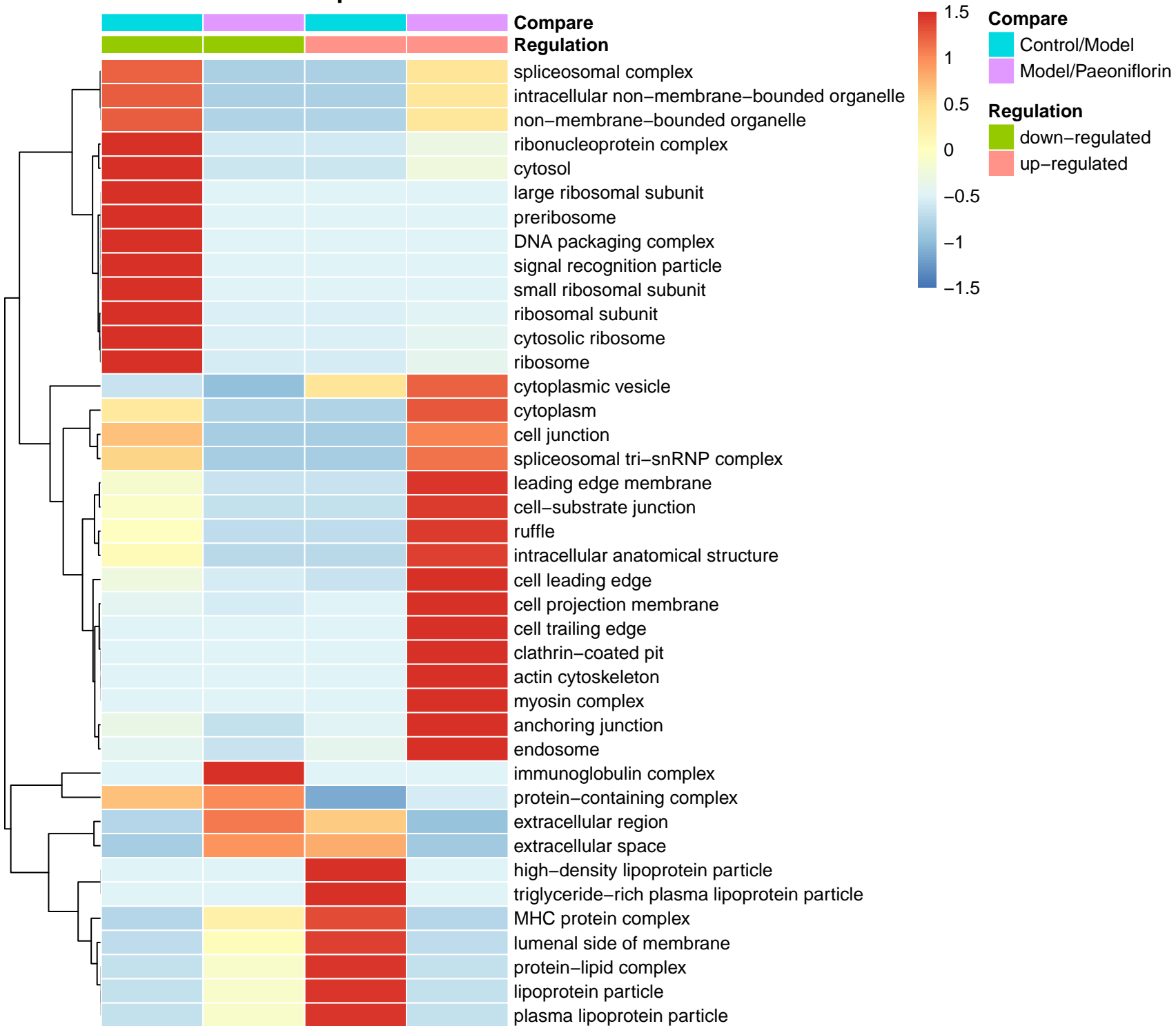

Supplement: Supplementary file 1 [file ijms-27-06236-s001.zip › Supplementary Materials/ijms-4276706_Proteomics_Dataset/6-Functional_enrichment_cluster/Figure 5. Enrichment cluster of Cellular component.pdf]

# KEGG

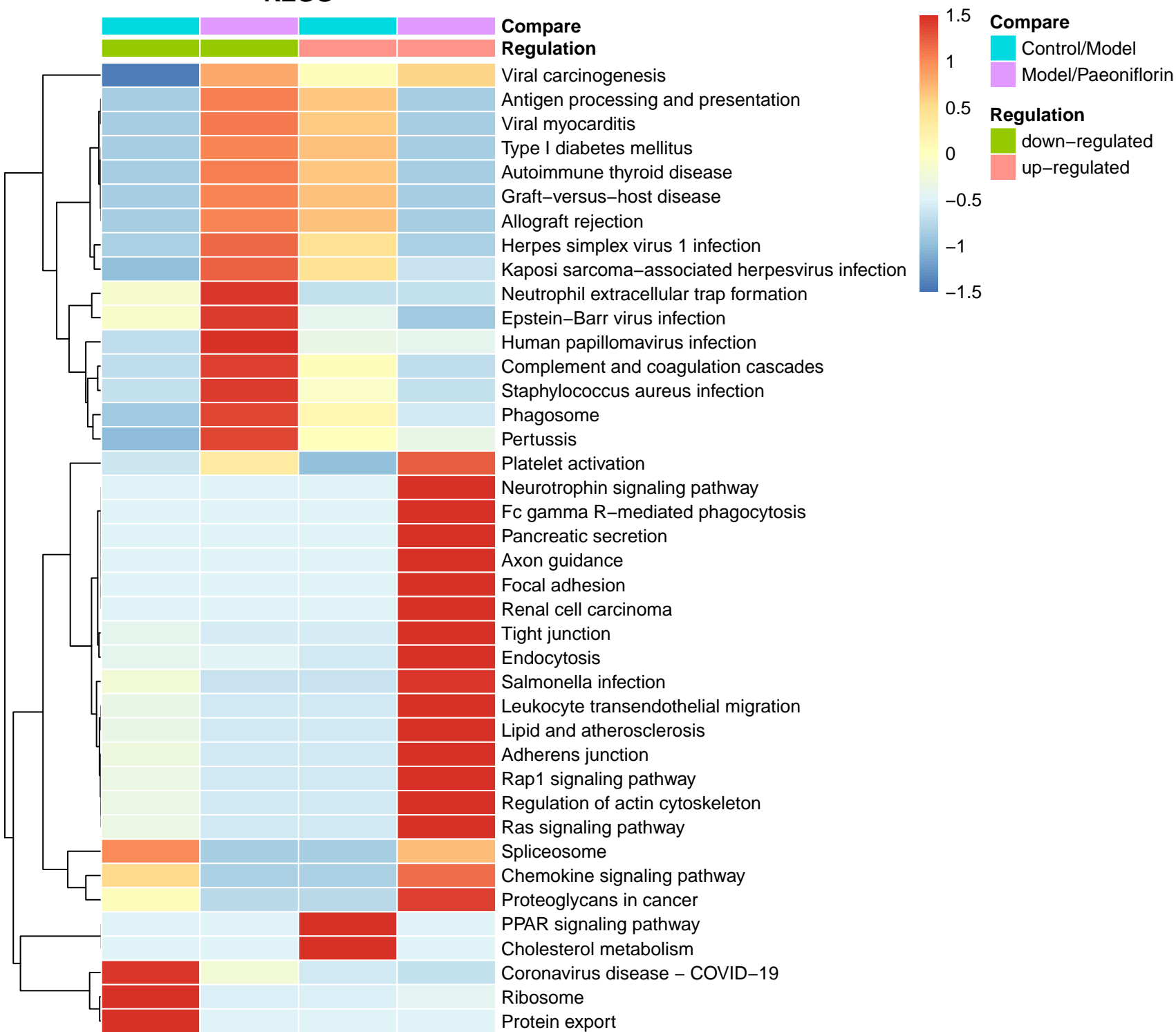

Supplement: Supplementary file 1 [file ijms-27-06236-s001.zip › Supplementary Materials/ijms-4276706_Proteomics_Dataset/6-Functional_enrichment_cluster/Figure 5. Enrichment cluster of KEGG.pdf]

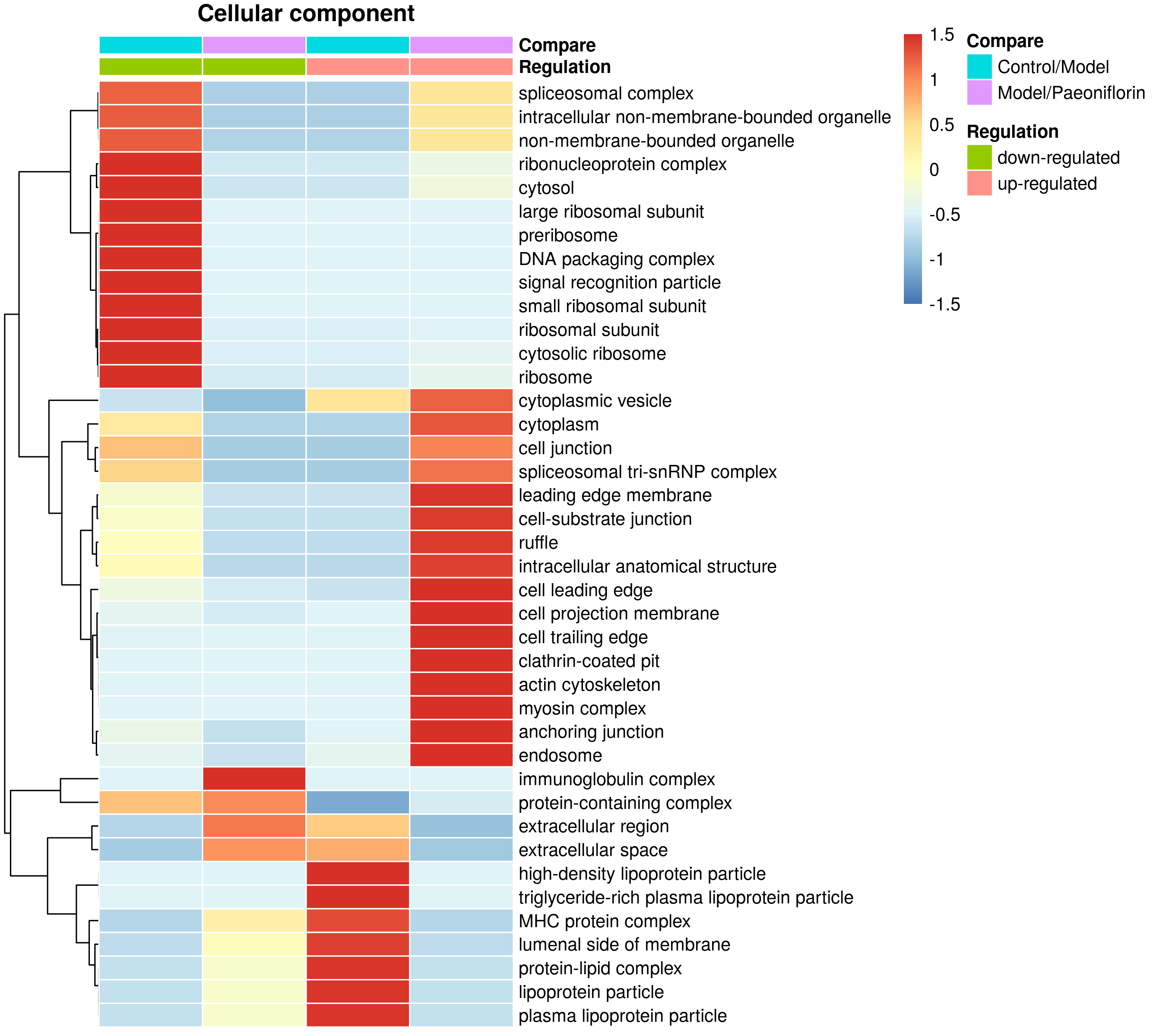

Supplement: Supplementary file 1 [file ijms-27-06236-s001.zip › Supplementary Materials/ijms-4276706_Proteomics_Dataset/6-Functional_enrichment_cluster/Figure 5. Enrichment cluster of Cellular component.png]

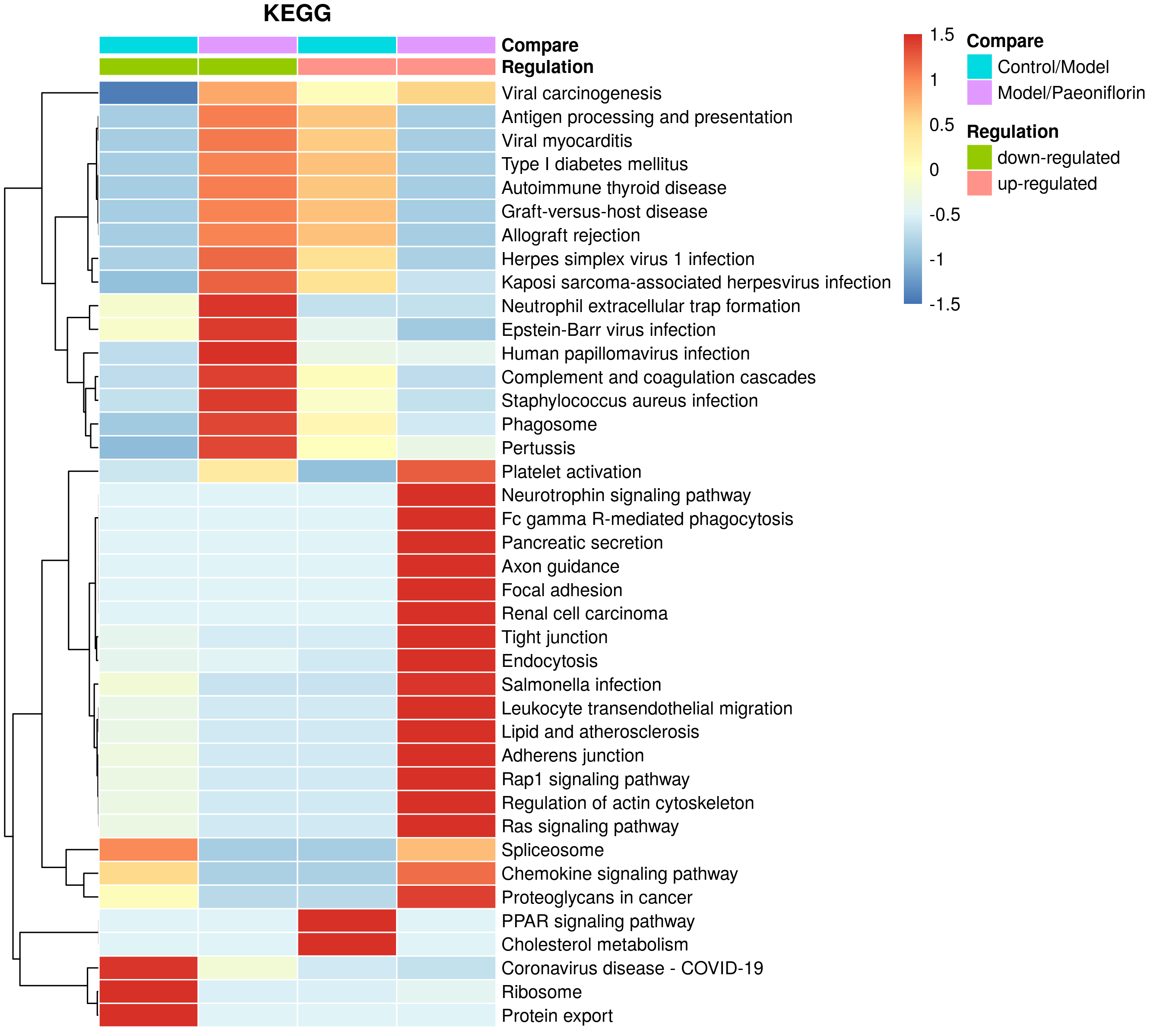

Supplement: Supplementary file 1 [file ijms-27-06236-s001.zip › Supplementary Materials/ijms-4276706_Proteomics_Dataset/6-Functional_enrichment_cluster/Figure 5. Enrichment cluster of KEGG.png]

# Molecular function

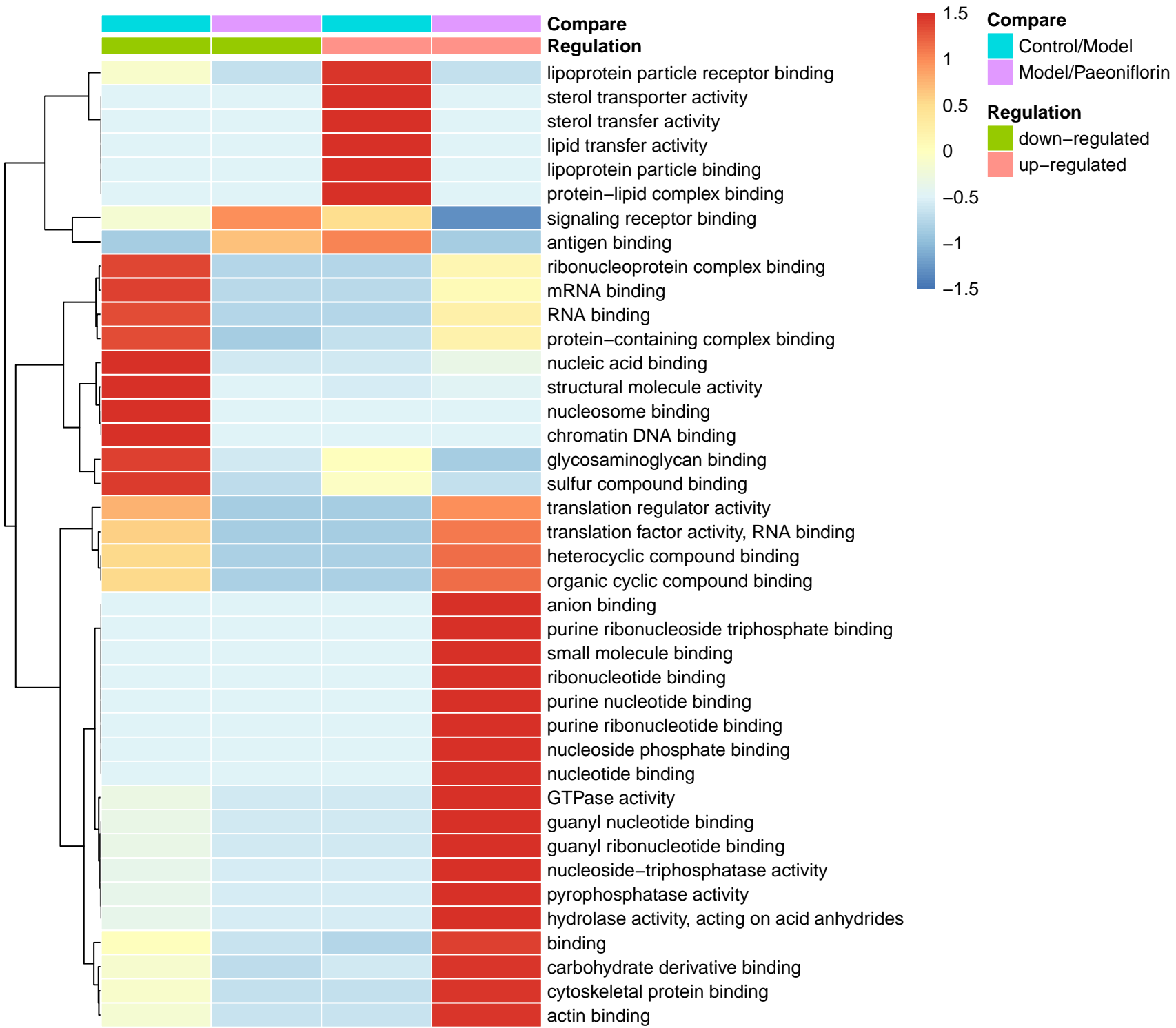

Supplement: Supplementary file 1 [file ijms-27-06236-s001.zip › Supplementary Materials/ijms-4276706_Proteomics_Dataset/6-Functional_enrichment_cluster/Figure 5. Enrichment cluster of Molecular function.pdf]

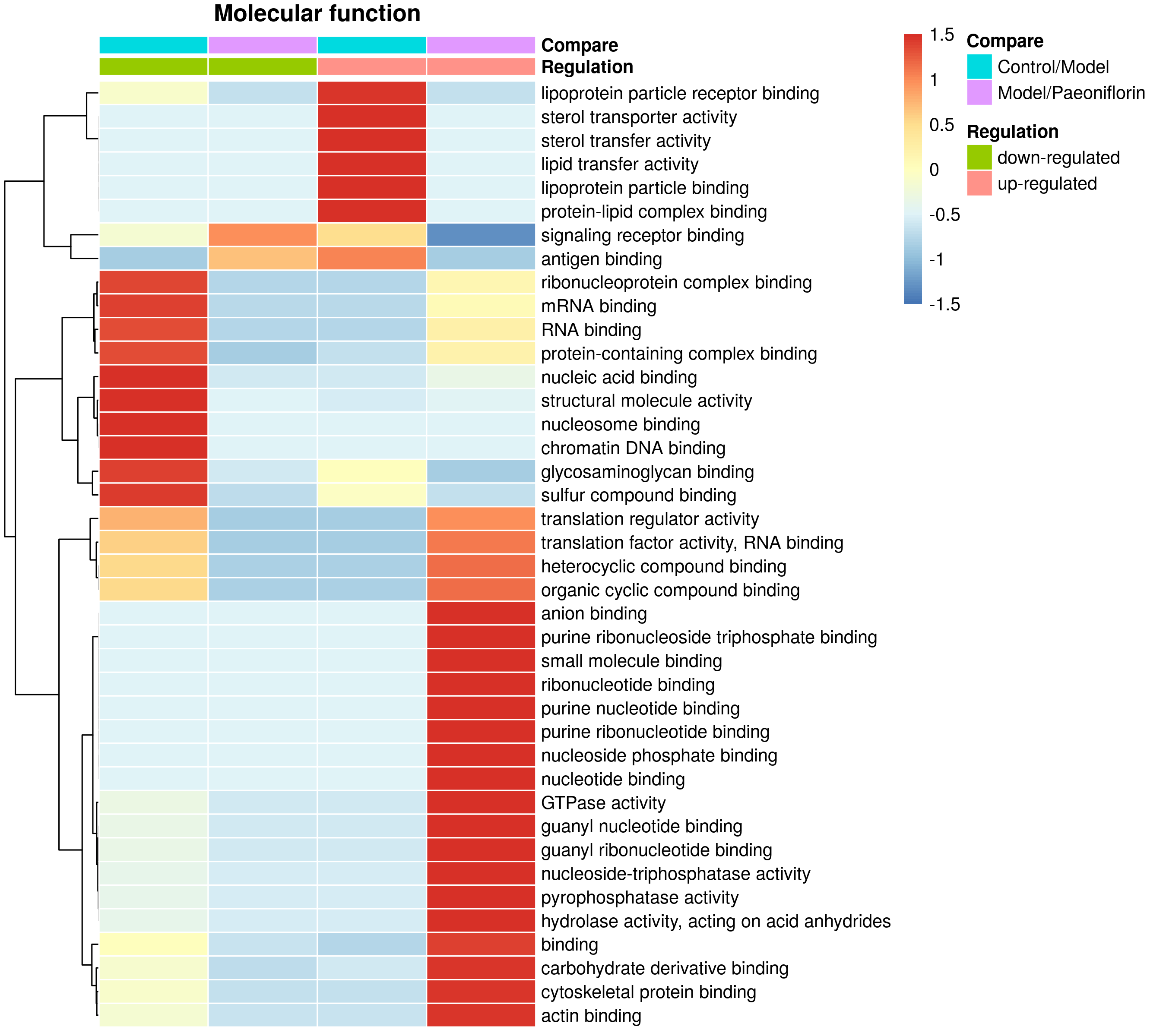

Supplement: Supplementary file 1 [file ijms-27-06236-s001.zip › Supplementary Materials/ijms-4276706_Proteomics_Dataset/6-Functional_enrichment_cluster/Figure 5. Enrichment cluster of Molecular function.png]

# Biological process

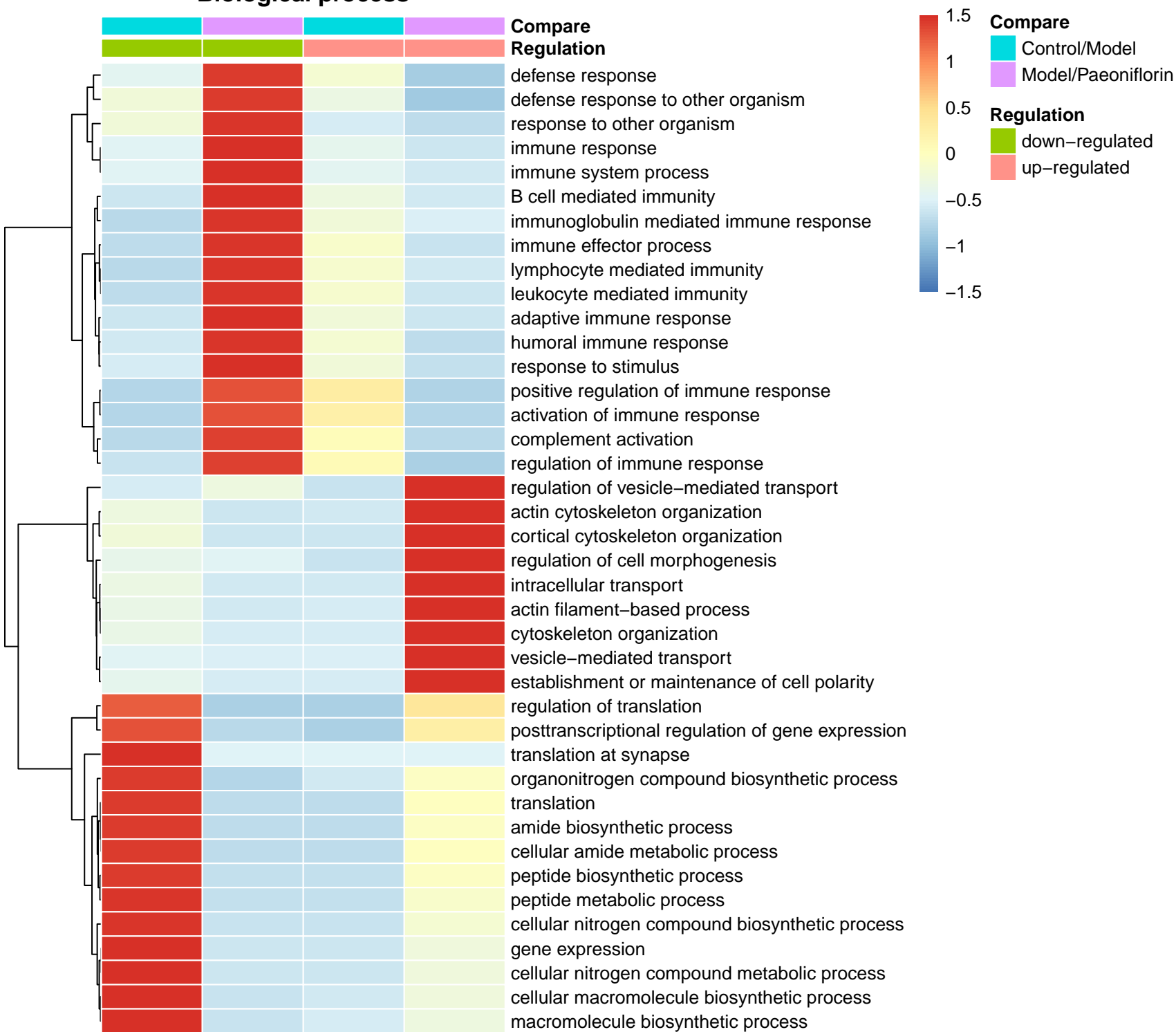

Supplement: Supplementary file 1 [file ijms-27-06236-s001.zip › Supplementary Materials/ijms-4276706_Proteomics_Dataset/6-Functional_enrichment_cluster/Figure 5. Enrichment cluster of Biological process.pdf]

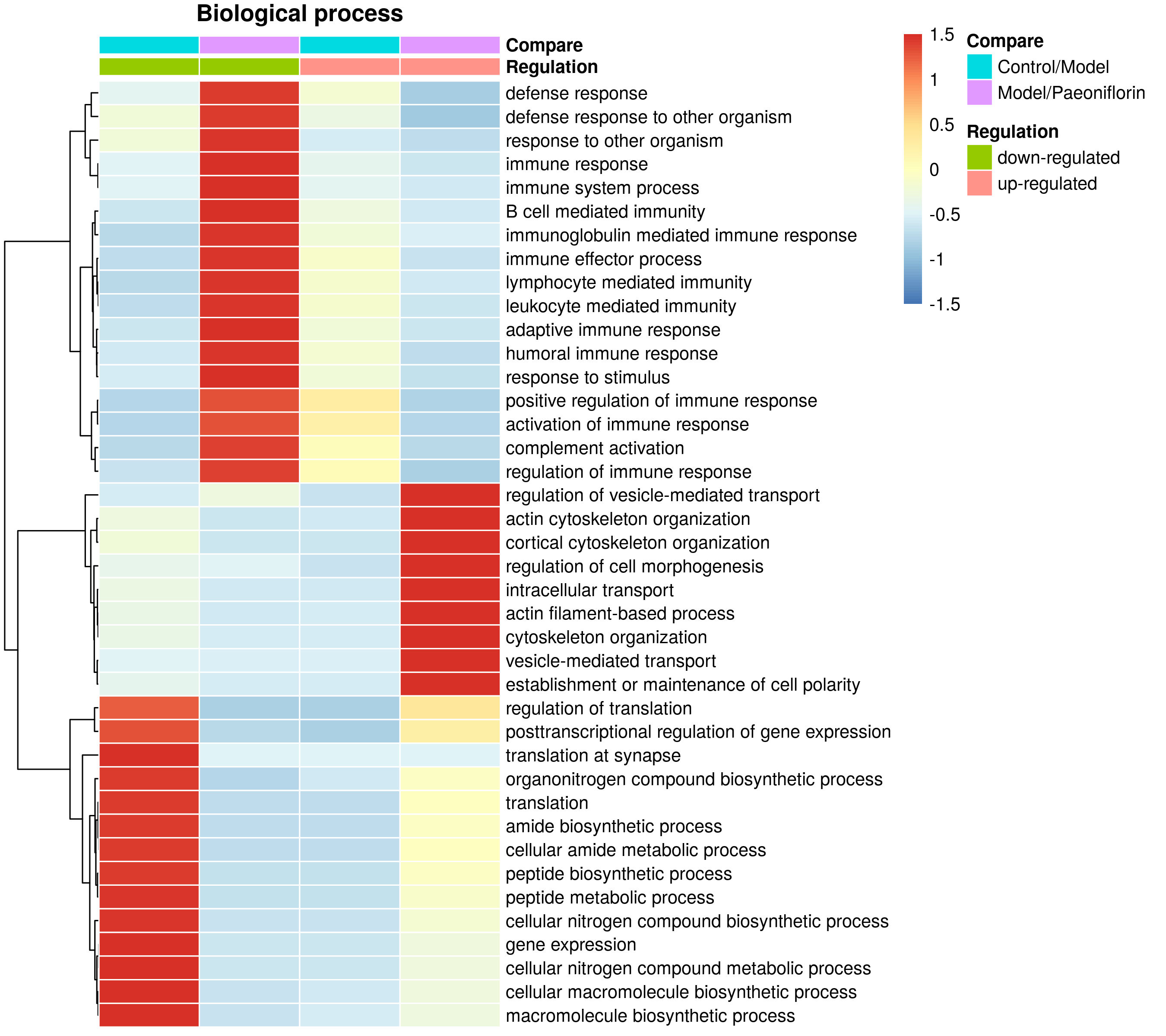

Supplement: Supplementary file 1 [file ijms-27-06236-s001.zip › Supplementary Materials/ijms-4276706_Proteomics_Dataset/6-Functional_enrichment_cluster/Figure 5. Enrichment cluster of Biological process.png]

# Pfam

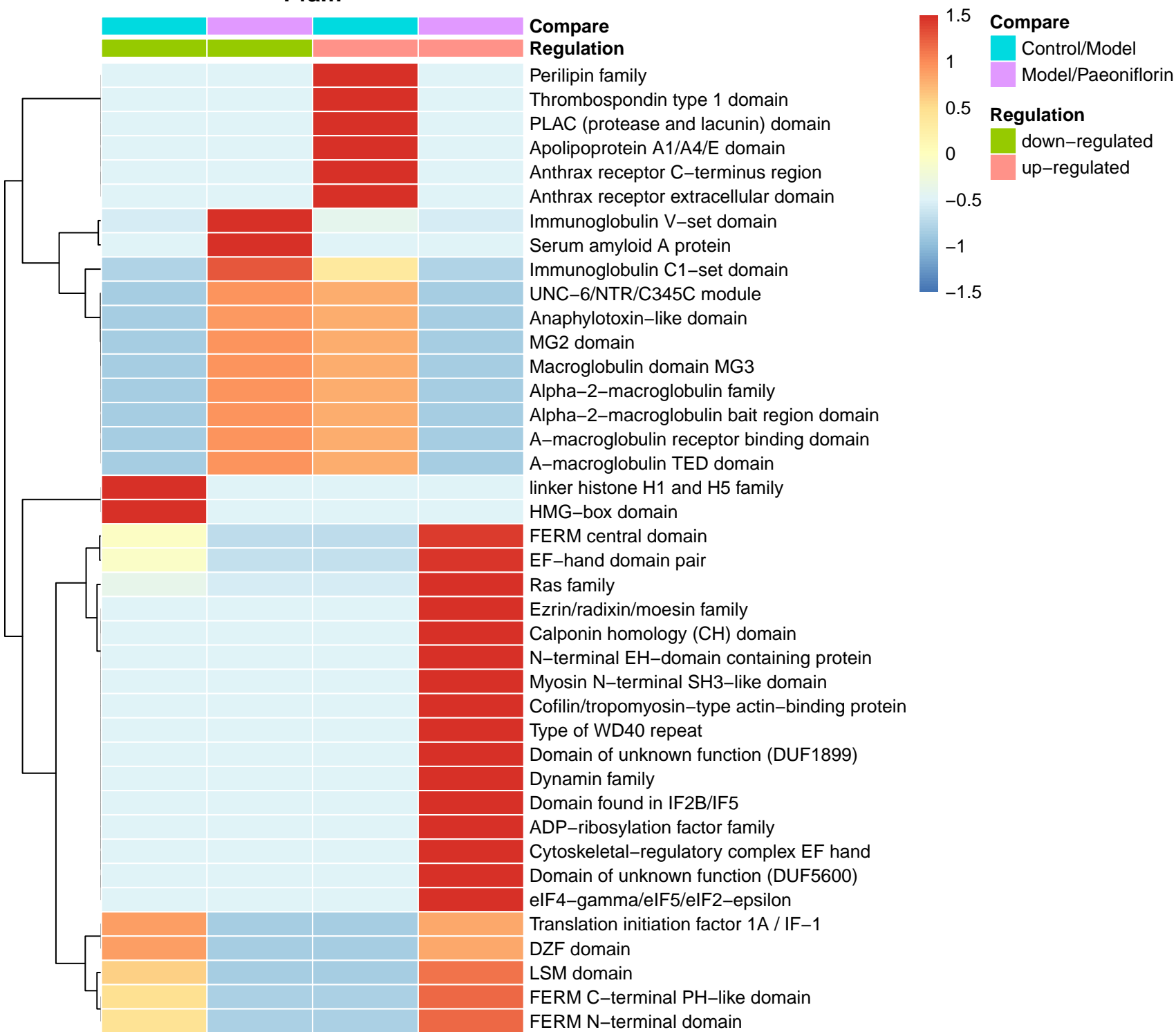

Supplement: Supplementary file 1 [file ijms-27-06236-s001.zip › Supplementary Materials/ijms-4276706_Proteomics_Dataset/6-Functional_enrichment_cluster/Figure 5. Enrichment cluster of Pfam.pdf]

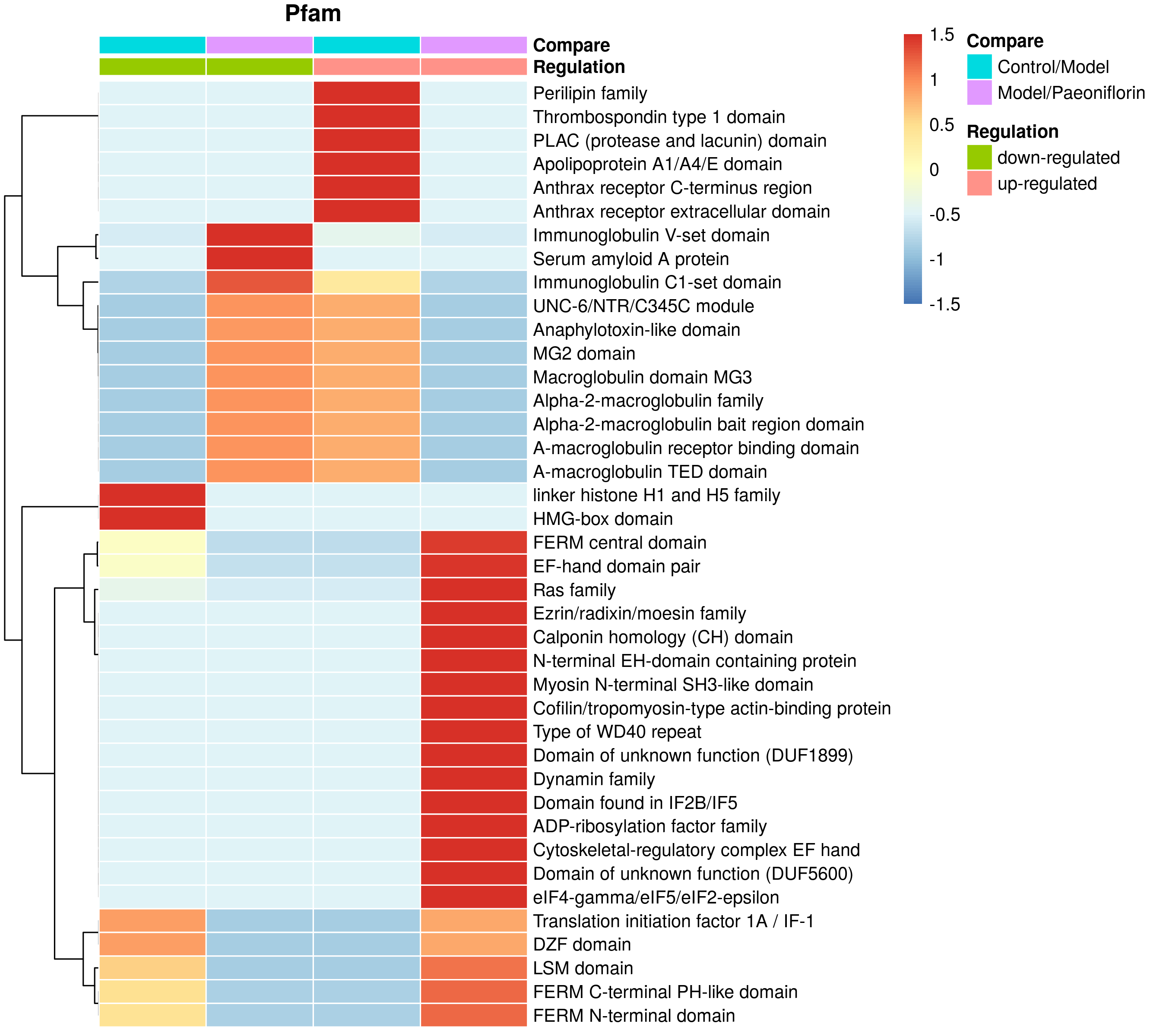

Supplement: Supplementary file 1 [file ijms-27-06236-s001.zip › Supplementary Materials/ijms-4276706_Proteomics_Dataset/6-Functional_enrichment_cluster/Figure 5. Enrichment cluster of Pfam.png]

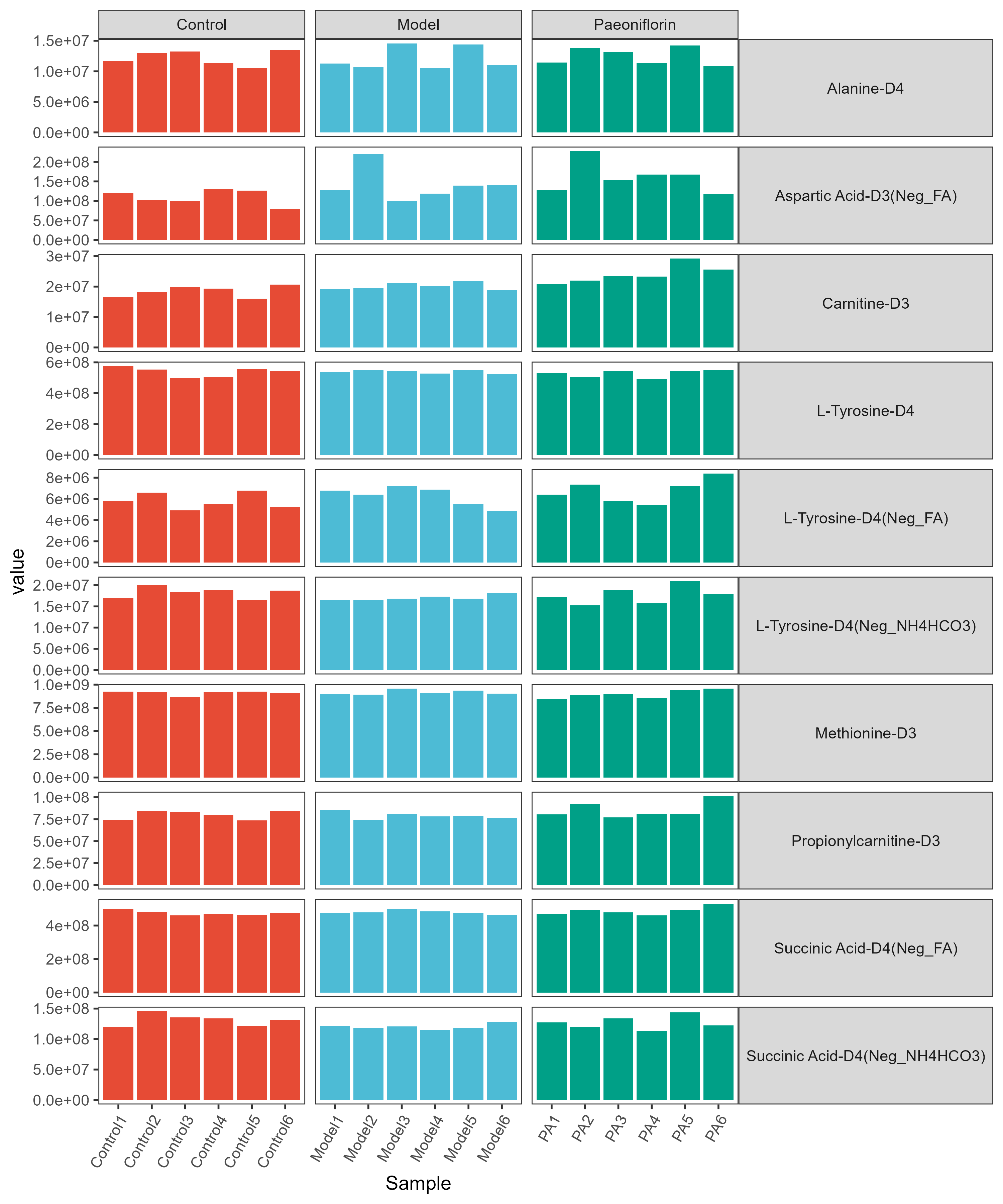

Supplement: Supplementary file 1 [file ijms-27-06236-s001.zip › Supplementary Materials/ijms-4276706_Metabolomics_Dataset/1-MS_identified_summary/Identification_QC/QC plot.png]

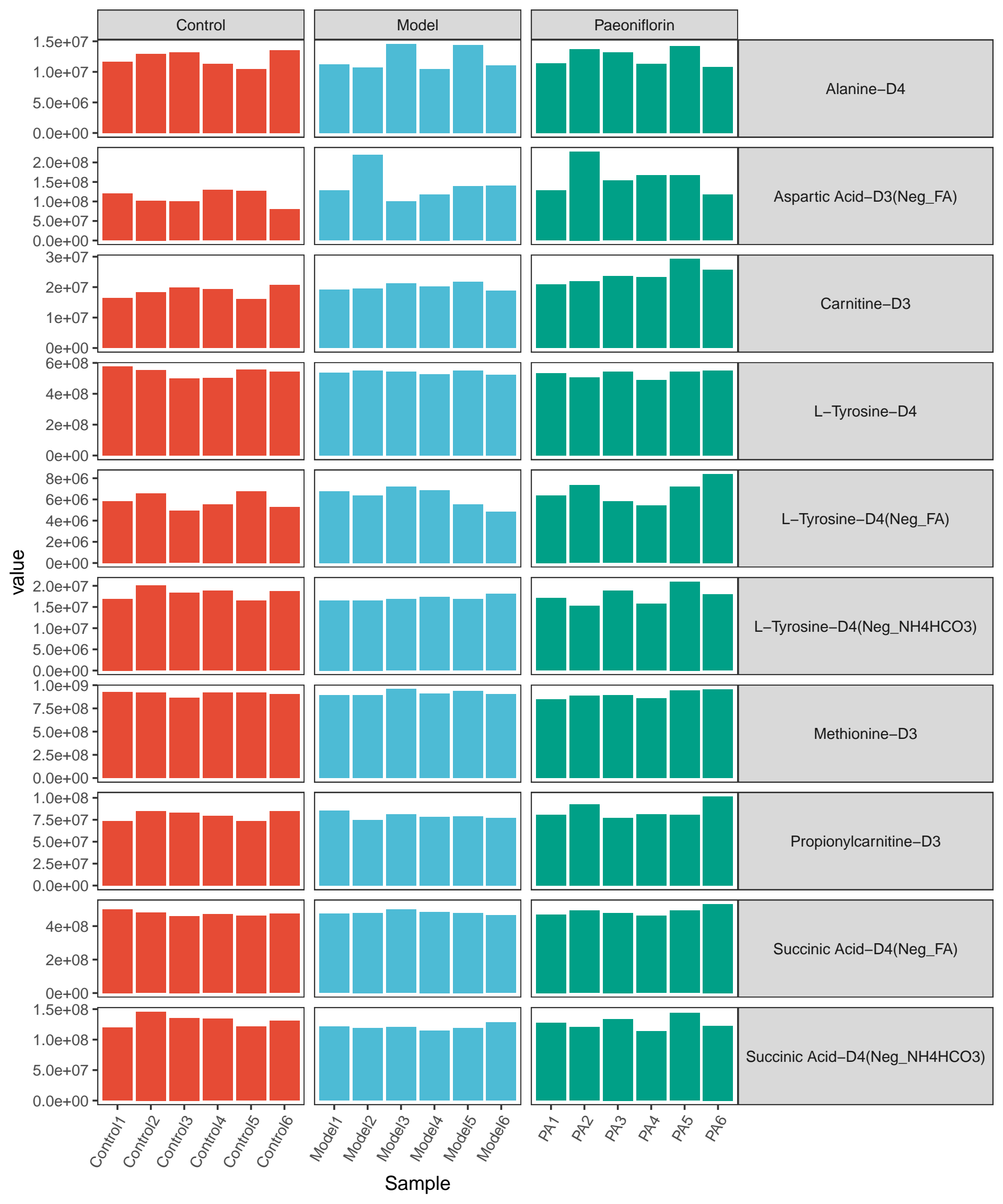

Supplement: Supplementary file 1 [file ijms-27-06236-s001.zip › Supplementary Materials/ijms-4276706_Metabolomics_Dataset/1-MS_identified_summary/Identification_QC/QC plot.pdf]

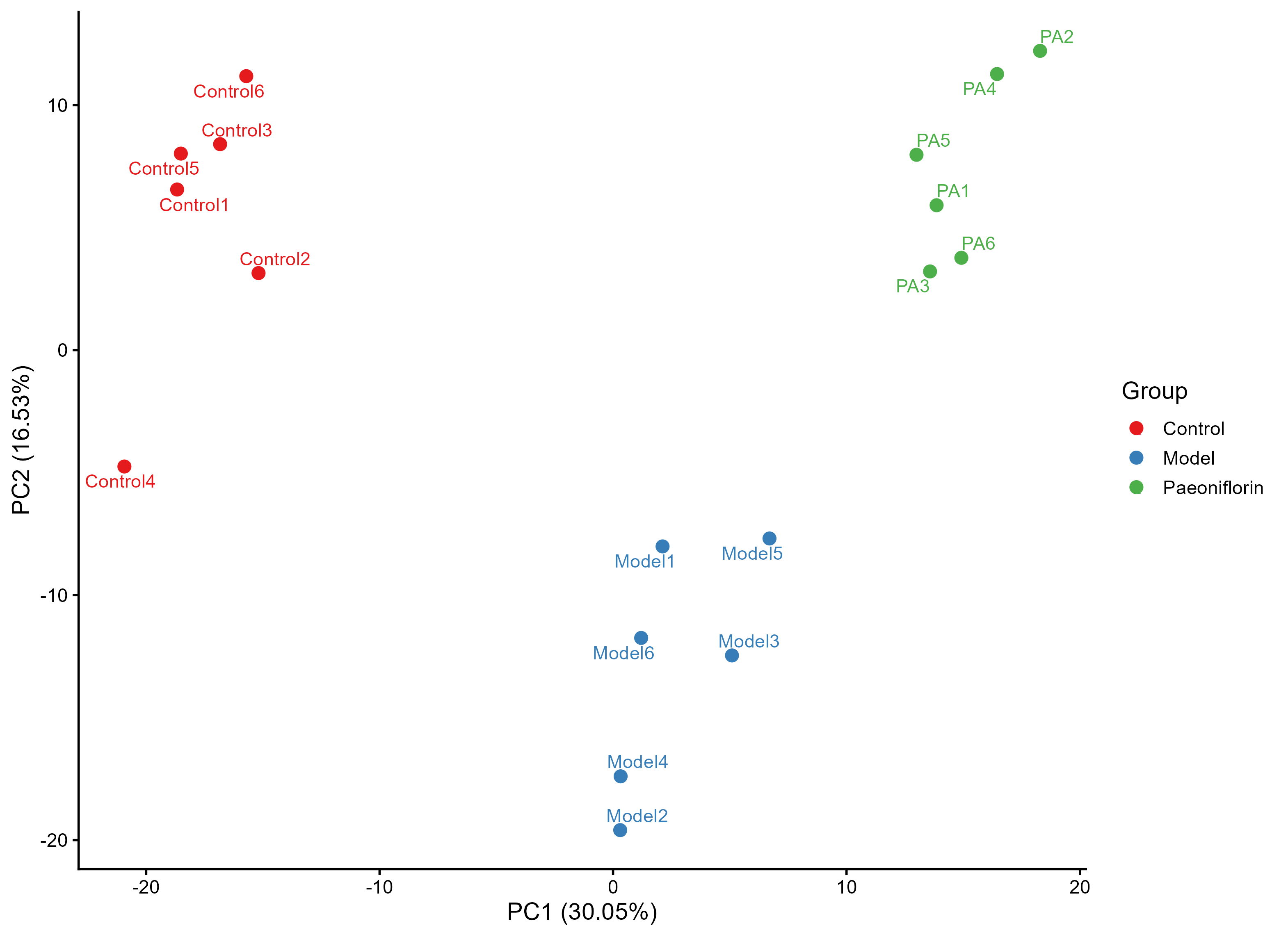

Supplement: Supplementary file 1 [file ijms-27-06236-s001.zip › Supplementary Materials/ijms-4276706_Metabolomics_Dataset/1-MS_identified_summary/Quantification_QC/Figure 1c. Metabolites quantitation PCA plot.png]

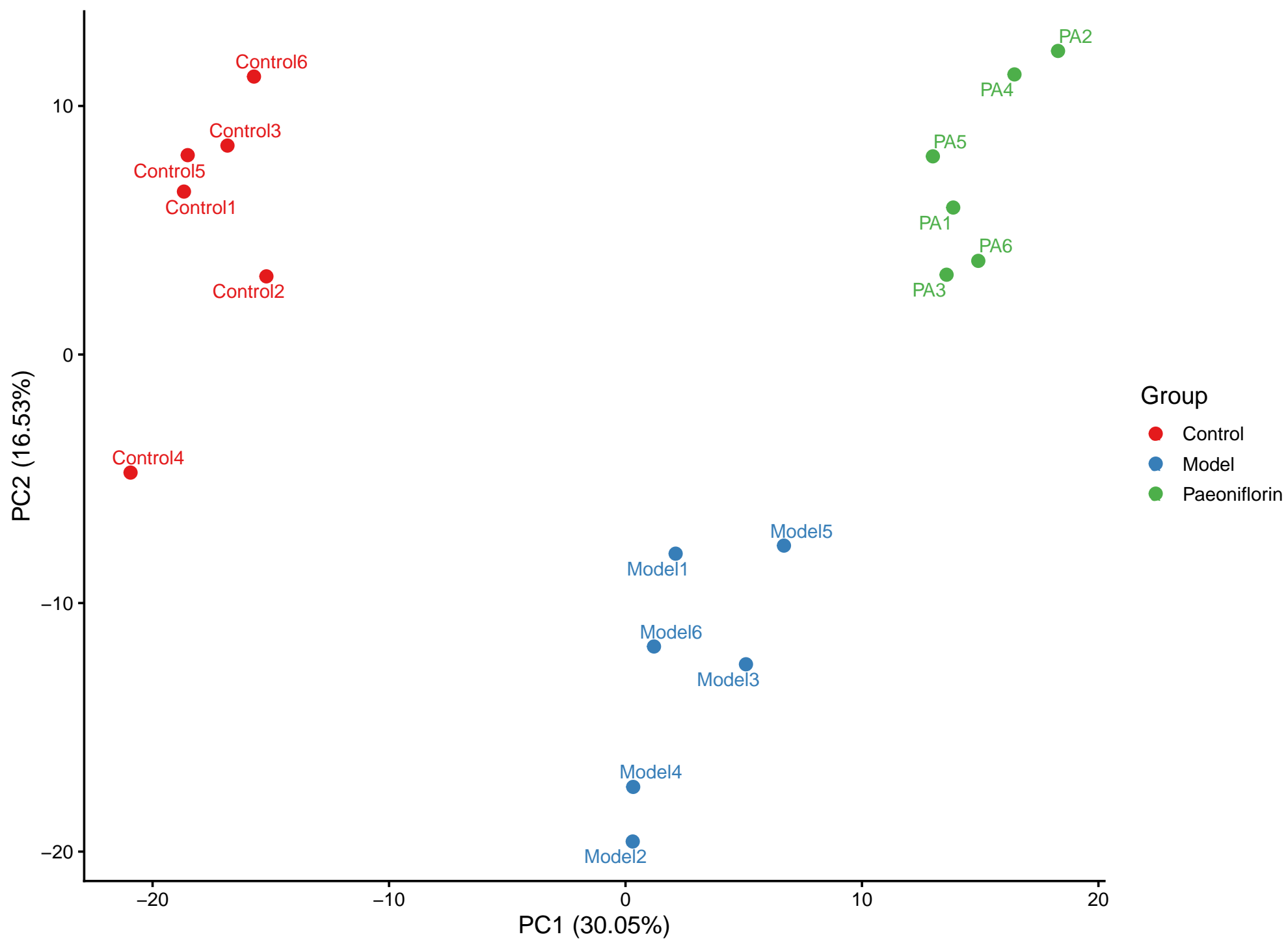

Supplement: Supplementary file 1 [file ijms-27-06236-s001.zip › Supplementary Materials/ijms-4276706_Metabolomics_Dataset/1-MS_identified_summary/Quantification_QC/Figure 1c. Metabolites quantitation PCA plot.pdf]

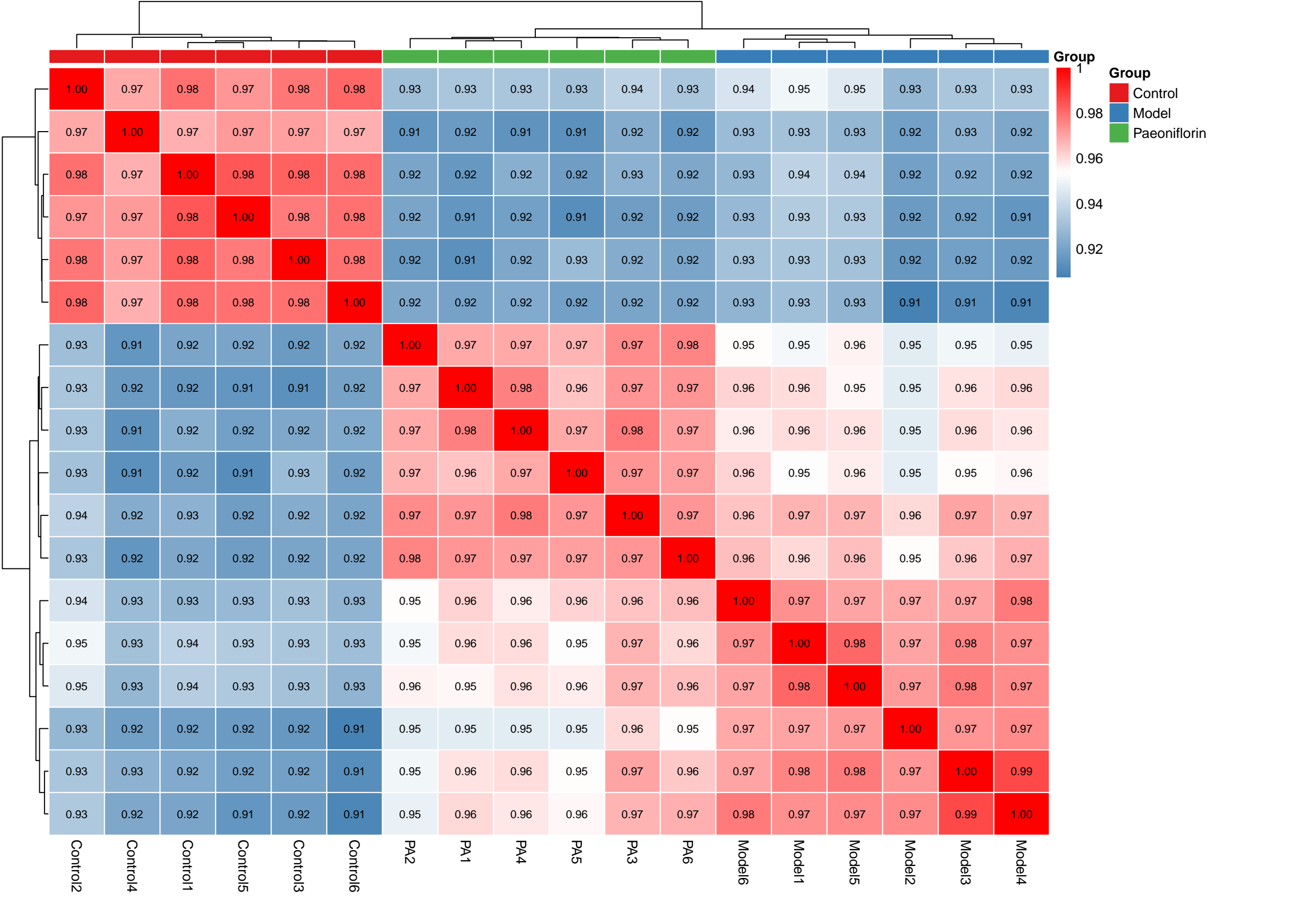

Supplement: Supplementary file 1 [file ijms-27-06236-s001.zip › Supplementary Materials/ijms-4276706_Metabolomics_Dataset/1-MS_identified_summary/Quantification_QC/Figure 1d. Metabolites quantitation PCC plot.pdf]

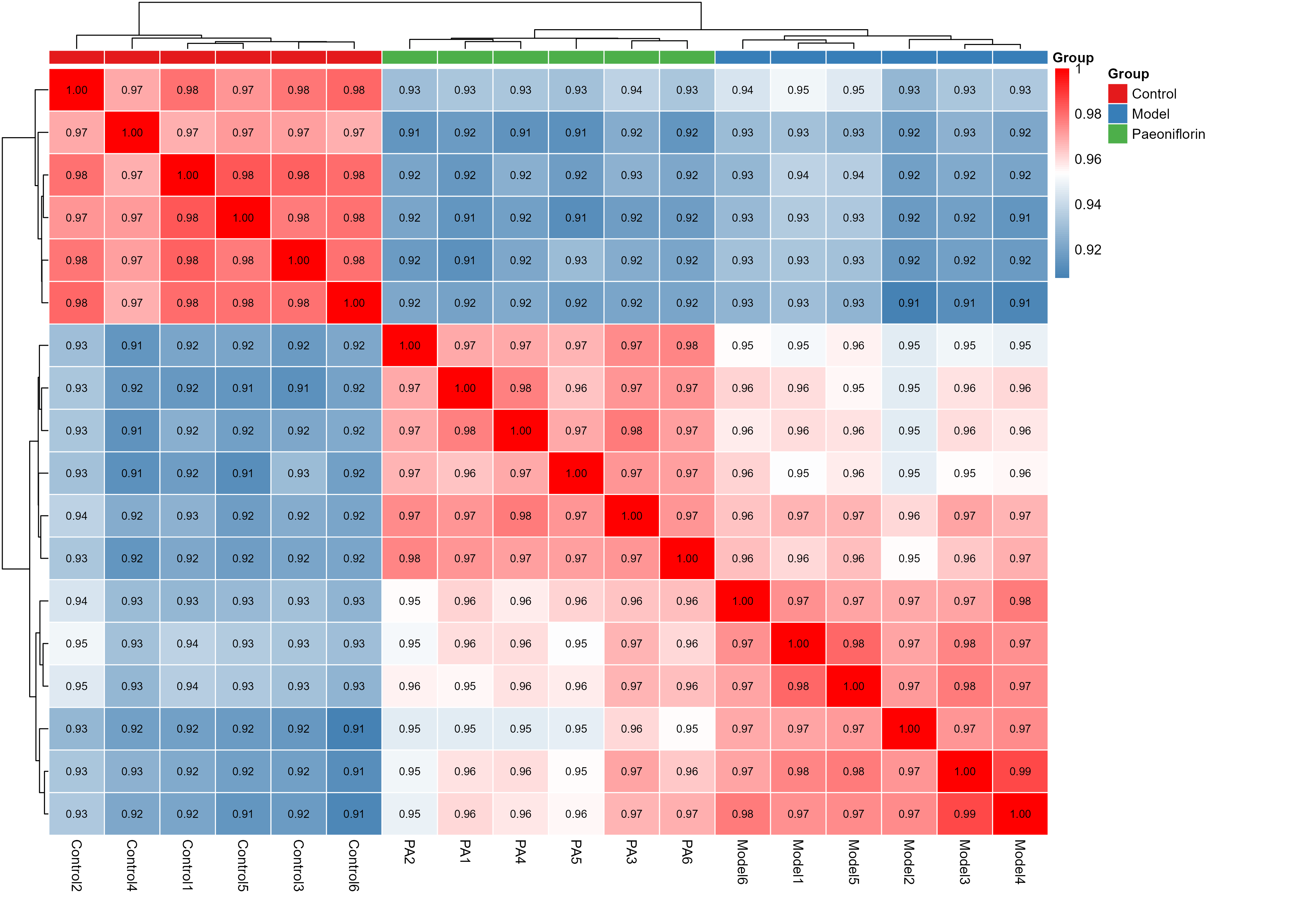

Supplement: Supplementary file 1 [file ijms-27-06236-s001.zip › Supplementary Materials/ijms-4276706_Metabolomics_Dataset/1-MS_identified_summary/Quantification_QC/Figure 1d. Metabolites quantitation PCC plot.png]

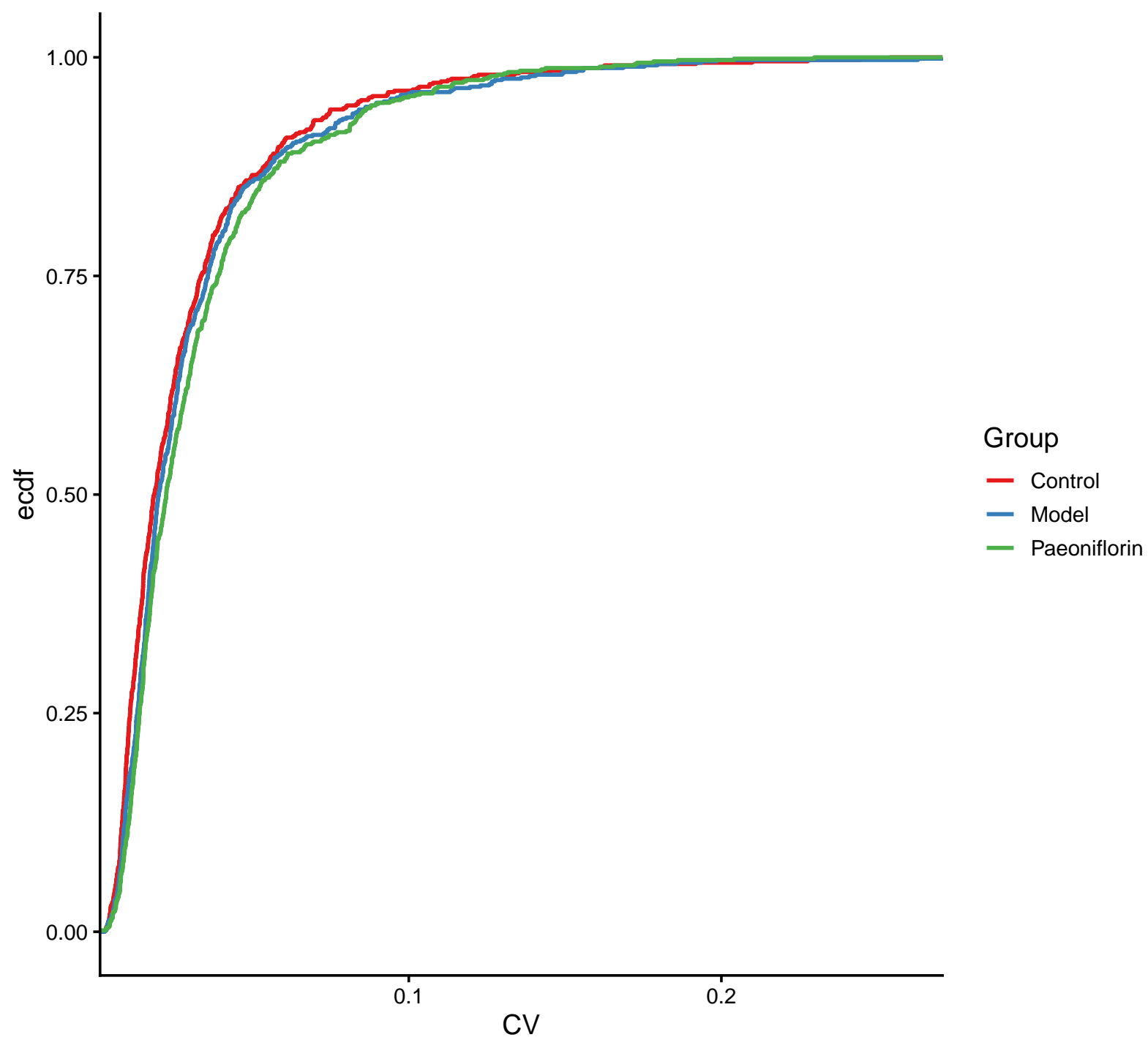

Supplement: Supplementary file 1 [file ijms-27-06236-s001.zip › Supplementary Materials/ijms-4276706_Metabolomics_Dataset/1-MS_identified_summary/Quantification_QC/Figure 1b. Metabolites quantitation CV plot.pdf]

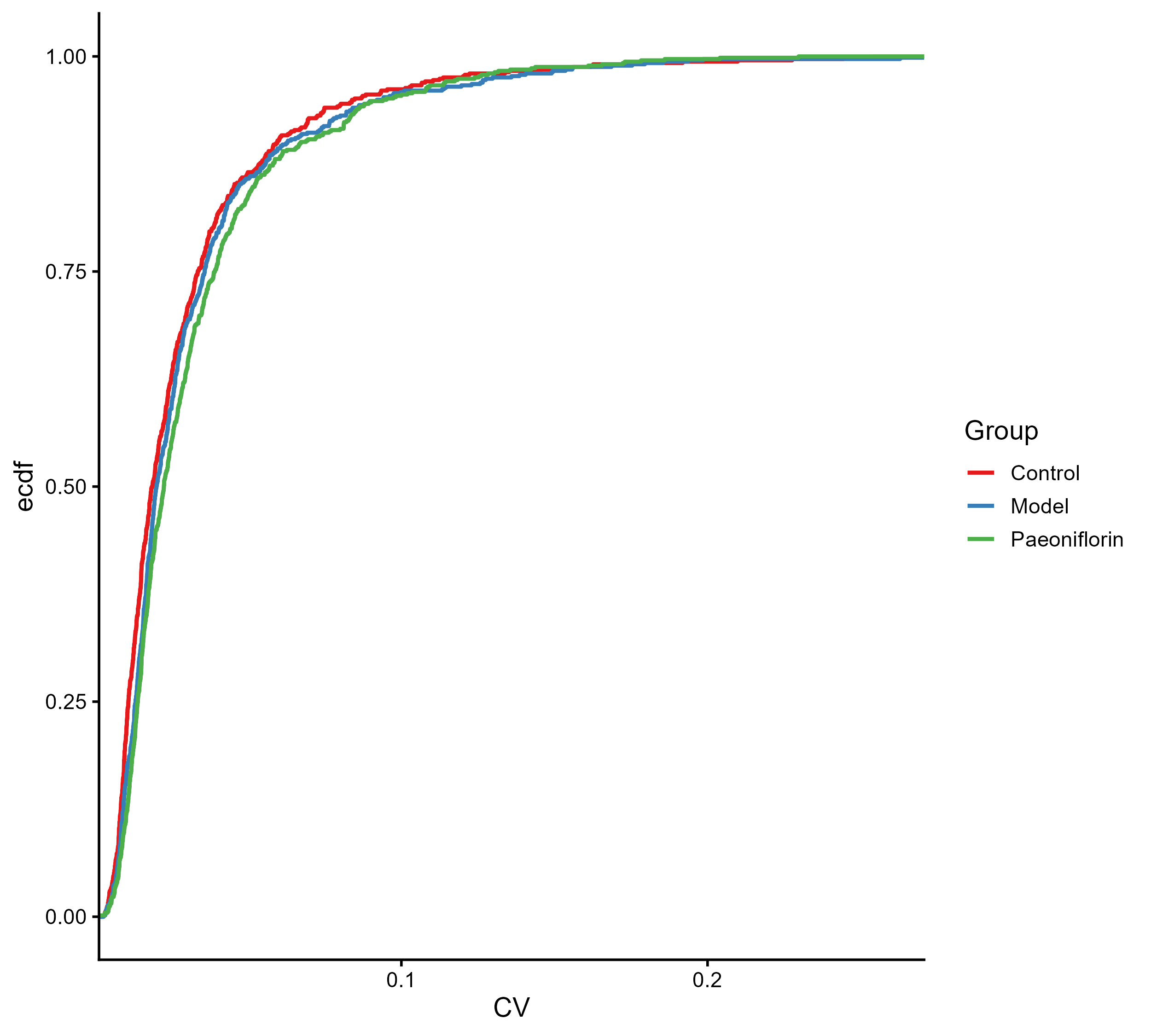

Supplement: Supplementary file 1 [file ijms-27-06236-s001.zip › Supplementary Materials/ijms-4276706_Metabolomics_Dataset/1-MS_identified_summary/Quantification_QC/Figure 1b. Metabolites quantitation CV plot.png]
