# Supplementary material for: Multi-Omics and Experimental Validation Reveal the Protective Effect of Paeoniflorin Against Coronary Heart Disease in Mice via Inhibiting the C3-Cfd-C3aR Pathway
Source: Int J Mol Sci. 2026 Jul 13;27(14):6236. doi: 10.3390/ijms27146236 (PMC13410309; doi:10.3390/ijms27146236)
Supplement: Supplementary file 1 [file ijms-27-06236-s001.zip › Supplementary Materials/ijms-4276706_Proteomics_Dataset/8-KEGG_pathway_image/Model-vs-Paeoniflorin/mmu04972.html]

KEGG PATHWAY: Pancreatic secretion - Mus musculus (house mouse)


# Pancreatic secretion - Mus musculus (house mouse)


[
Pathway menu
| Organism menu
| Pathway entry
| Show description
| Download
| Help
]

The pancreas performs both exocrine and endocrine functions. The exocrine pancreas consists of two parts, the acinar and duct cells. The primary functions of pancreatic acinar cells are to synthesize and secrete digestive enzymes. Stimulation of the cell by secretagogues such as acetylcholine (ACh) and cholecystokinin (CCK) causes the generation of an intracellular Ca2+ signal. This signal, in turn, triggers the fusion of the zymogen granules with the apical plasma membrane, leading to the polarised secretion of the enzymes. The major task of pancreatic duct cells is the secretion of fluid and bicarbonate ions (HCO3-), which neutralize the acidity of gastric contents that enter the duodenum. An increase in intracellular cAMP by secretin is one of the major signals of pancreatic HCO3- secretion. Activation of the CFTR Cl- channel and the CFTR-dependent Cl-/HCO3- exchange activities is responsible for cAMP-induced HCO3- secretion.


##### Option

Scale:


100%

Image resolution:


 High

##### Background color

Organism

##### Search

##### ID search

##### Color


KGML

Image (png) file 1x

Image (png) file 2x
