# Supplementary material for: Multi-Omics and Experimental Validation Reveal the Protective Effect of Paeoniflorin Against Coronary Heart Disease in Mice via Inhibiting the C3-Cfd-C3aR Pathway
Source: Int J Mol Sci. 2026 Jul 13;27(14):6236. doi: 10.3390/ijms27146236 (PMC13410309; doi:10.3390/ijms27146236)
Supplement: Supplementary file 1 [file ijms-27-06236-s001.zip › Supplementary Materials/ijms-4276706_Proteomics_Dataset/8-KEGG_pathway_image/Model-vs-Paeoniflorin/mmu05322.html]

KEGG PATHWAY: Systemic lupus erythematosus - Mus musculus (house mouse)


# Systemic lupus erythematosus - Mus musculus (house mouse)


[
Pathway menu
| Organism menu
| Pathway entry
| Show description
| Download
| Help
]

Systemic lupus erythematosus (SLE) is a prototypic autoimmune disease characterised by the production of IgG autoantibodies that are specific for self-antigens, such as DNA, nuclear proteins and certain cytoplasmic components, in association with a diverse array of clinical manifestations. The primary pathological findings in patients with SLE are those of inflammation, vasculitis, immune complex deposition, and vasculopathy. Immune complexes comprising autoantibody and self-antigen is deposited particulary in the renal glomeruli and mediate a systemic inflammatory response by activating complement or via Fc{gamma}R-mediated neutrophil and macrophage activation. Activation of complement (C5) leads to injury both through formation of the membrane attack complex (C5b-9) or by generation of the anaphylatoxin and cell activator C5a. Neutrophils and macrophages cause tissue injury by the release of oxidants and proteases.


##### Option

Scale:


100%

Image resolution:


 High

##### Background color

Organism

##### Search

##### ID search

##### Color


KGML

Image (png) file 1x

Image (png) file 2x
