# Supplementary material for: Multi-Omics and Experimental Validation Reveal the Protective Effect of Paeoniflorin Against Coronary Heart Disease in Mice via Inhibiting the C3-Cfd-C3aR Pathway
Source: Int J Mol Sci. 2026 Jul 13;27(14):6236. doi: 10.3390/ijms27146236 (PMC13410309; doi:10.3390/ijms27146236)
Supplement: Supplementary file 1 [file ijms-27-06236-s001.zip › Supplementary Materials/ijms-4276706_Proteomics_Dataset/8-KEGG_pathway_image/Model-vs-Paeoniflorin/mmu05135.html]

KEGG PATHWAY: Yersinia infection - Mus musculus (house mouse)


# Yersinia infection - Mus musculus (house mouse)


[
Pathway menu
| Organism menu
| Pathway entry
| Show description
| Download
| Help
]

Pathogenic Yersinia injects virulent Yersinia outer proteins (Yops) into the host cell through a type three secretion system (T3SS) to express its pathogenicity. Through inactivation of small GTPases by YopT protease, YopE GTPase-activating protein, and YpkA/YopO sequestration of GDP-bound small GTPases, Yersinia prevents its uptake by phagocytic cells and disrupts the actin cytoskeleton. YopH phosphatase shuts down phagocytosis by dephosphorylating key host proteins as well as suppresses T cell activation. Yersinia induces macrophage pyroptosis via YopB/YopD activation of canonical and noncanonical inflammasomes and YopT/YopE-mediated activation of pyrin. These effects are counteracted by YopK and YopM. YopP/J is a member of an ubiquitin-like protein cysteine protease family and simultaneously targets signaling components of the MAPK and NF-kappaB signaling pathways following activation of Toll-like receptor4 to inhibit production of proinflammatory cytokines.


##### Option

Scale:


100%

Image resolution:


 High

##### Background color

Organism

##### Search

##### ID search

##### Color


KGML

Image (png) file 1x

Image (png) file 2x
