# Supplementary material for: Multi-Omics and Experimental Validation Reveal the Protective Effect of Paeoniflorin Against Coronary Heart Disease in Mice via Inhibiting the C3-Cfd-C3aR Pathway
Source: Int J Mol Sci. 2026 Jul 13;27(14):6236. doi: 10.3390/ijms27146236 (PMC13410309; doi:10.3390/ijms27146236)
Supplement: Supplementary file 1 [file ijms-27-06236-s001.zip › Supplementary Materials/ijms-4276706_Proteomics_Dataset/8-KEGG_pathway_image/Model-vs-Paeoniflorin/mmu04270.html]

KEGG PATHWAY: Vascular smooth muscle contraction - Mus musculus (house mouse)


# Vascular smooth muscle contraction - Mus musculus (house mouse)


[
Pathway menu
| Organism menu
| Pathway entry
| Show description
| Download
| Help
]

The vascular smooth muscle cell (VSMC) is a highly specialized cell whose principal function is contraction. On contraction, VSMCs shorten, thereby decreasing the diameter of a blood vessel to regulate the blood flow and pressure.
The principal mechanisms that regulate the contractile state of VSMCs are changes in cytosolic Ca2+ concentration ([Ca2+]c). In response to vasoconstrictor stimuli, Ca2+ is mobilized from intracellular stores and/or the extracellular space to increase [Ca2+]c in VSMCs. The increase in [Ca2+]c, in turn, activates the Ca2+-CaM-MLCK pathway and stimulates MLC20 phosphorylation, leading to myosin-actin interactions and, hence, the development of contractile force. The sensitivity of contractile myofilaments or MLC20 phosphorylation to Ca2+ can be secondarily modulated by other signaling pathways. During receptor stimulation, the contractile force is greatly enhanced by the inhibition of myosin phosphatase. Rho/Rho kinase, PKC, and arachidonic acid have been proposed to play a pivotal role in this enhancement.
The signaling events that mediate relaxation include the removal of a contractile agonist (passive relaxation) and activation of cyclic nucleotide-dependent signaling pathways in the continued presence of a contractile agonist (active relaxation). Active relaxation occurs through the inhibition of both Ca2+ mobilization and myofilament Ca2+ sensitivity in VSMCs.


##### Option

Scale:


100%

Image resolution:


 High

##### Background color

Organism

##### Search

##### ID search

##### Color


KGML

Image (png) file 1x

Image (png) file 2x
