# Supplementary material for: Multi-Omics and Experimental Validation Reveal the Protective Effect of Paeoniflorin Against Coronary Heart Disease in Mice via Inhibiting the C3-Cfd-C3aR Pathway
Source: Int J Mol Sci. 2026 Jul 13;27(14):6236. doi: 10.3390/ijms27146236 (PMC13410309; doi:10.3390/ijms27146236)
Supplement: Supplementary file 1 [file ijms-27-06236-s001.zip › Supplementary Materials/ijms-4276706_Proteomics_Dataset/8-KEGG_pathway_image/Model-vs-Paeoniflorin/mmu03018.html]

KEGG PATHWAY: RNA degradation - Mus musculus (house mouse)


# RNA degradation - Mus musculus (house mouse)


[
Pathway menu
| Organism menu
| Pathway entry
| Show description
| Download
| Help
]

The correct processing, quality control and turnover of cellular RNA molecules are critical to many aspects in the expression of genetic information. In eukaryotes, two major pathways of mRNA decay exist and both pathways are initiated by poly(A) shortening of the mRNA. In the 5' to 3' pathway, this is followed by decapping which then permits the 5' to 3' exonucleolytic degradation of transcripts. In the 3' to 5' pathway, the exosome, a large multisubunit complex, plays a key role. The exosome exists in archaeal cells, too. In bacteria, endoribonuclease E, a key enzyme involved in RNA decay and processing, organizes a protein complex called degradosome. RNase E or R interacts with the phosphate-dependent exoribonuclease polynucleotide phosphorylase, DEAD-box helicases, and additional factors in the RNA-degrading complex.


##### Option

Scale:


100%

Image resolution:


 High

##### Background color

Organism

##### Search

##### ID search

##### Color

##### Related Brite

- 03019  Messenger RNA biogenesis


KGML

Image (png) file 1x

Image (png) file 2x
