# Supplementary material for: Multi-Omics and Experimental Validation Reveal the Protective Effect of Paeoniflorin Against Coronary Heart Disease in Mice via Inhibiting the C3-Cfd-C3aR Pathway
Source: Int J Mol Sci. 2026 Jul 13;27(14):6236. doi: 10.3390/ijms27146236 (PMC13410309; doi:10.3390/ijms27146236)
Supplement: Supplementary file 1 [file ijms-27-06236-s001.zip › Supplementary Materials/ijms-4276706_Proteomics_Dataset/8-KEGG_pathway_image/Model-vs-Paeoniflorin/mmu04512.html]

KEGG PATHWAY: ECM-receptor interaction - Mus musculus (house mouse)


# ECM-receptor interaction - Mus musculus (house mouse)


[
Pathway menu
| Organism menu
| Pathway entry
| Show description
| Download
| Help
]

The extracellular matrix (ECM) consists of a complex mixture of structural and functional macromolecules and serves an important role in tissue and organ morphogenesis and in the maintenance of cell and tissue structure and function. Specific interactions between cells and the ECM are mediated by transmembrane molecules, mainly integrins and perhaps also proteoglycans, CD36, or other cell-surface-associated components. These interactions lead to a direct or indirect control of cellular activities such as adhesion, migration, differentiation, proliferation, and apoptosis. In addition, integrins function as mechanoreceptors and provide a force-transmitting physical link between the ECM and the cytoskeleton. Integrins are a family of glycosylated, heterodimeric transmembrane adhesion receptors that consist of noncovalently bound alpha- and beta-subunits.


##### Option

Scale:


100%

Image resolution:


 High

##### Background color

Organism

##### Search

##### ID search

##### Color


KGML

Image (png) file 1x

Image (png) file 2x
