# Supplementary material for: Multi-Omics and Experimental Validation Reveal the Protective Effect of Paeoniflorin Against Coronary Heart Disease in Mice via Inhibiting the C3-Cfd-C3aR Pathway
Source: Int J Mol Sci. 2026 Jul 13;27(14):6236. doi: 10.3390/ijms27146236 (PMC13410309; doi:10.3390/ijms27146236)
Supplement: Supplementary file 1 [file ijms-27-06236-s001.zip › Supplementary Materials/ijms-4276706_Proteomics_Dataset/8-KEGG_pathway_image/Model-vs-Paeoniflorin/mmu05211.html]

KEGG PATHWAY: Renal cell carcinoma - Mus musculus (house mouse)


# Renal cell carcinoma - Mus musculus (house mouse)


[
Pathway menu
| Organism menu
| Pathway entry
| Show description
| Download
| Help
]

Renal cell cancer (RCC) accounts for ~3% of human malignancies and its incidence appears to be rising. Although most cases of RCC seem to occur sporadically, an inherited predisposition to renal cancer accounts for 1-4% of cases. RCC is not a single disease, it has several morphological subtypes. Conventional RCC (clear cell RCC) accounts for ~80% of cases, followed by papillary RCC (10-15%), chromophobe RCC (5%), and collecting duct RCC (<1%). Genes potentially involved in sporadic neoplasms of each particular type are VHL, MET, BHD, and FH respectively. In the absence of VHL, hypoxia-inducible factor alpha (HIF-alpha) accumulates, leading to production of several growth factors, including vascular endothelial growth factor and platelet-derived growth factor. Activated MET mediates a number of biological effects including motility, invasion of extracellular matrix, cellular transformation, prevention of apoptosis and metastasis formation. Loss of functional FH leads to accumulation of fumarate in the cell, triggering inhibition of HPH and preventing targeted pVHL-mediated degradation of HIF-alpha. BHD mutations cause the Birt-Hogg-Dube syndrome and its associated chromophobe, hybrid oncocytic, and conventional (clear cell) RCC.


##### Option

Scale:


100%

Image resolution:


 High

##### Background color

Organism

##### Search

##### ID search

##### Color


KGML

Image (png) file 1x

Image (png) file 2x
