# Supplementary material for: Multi-Omics and Experimental Validation Reveal the Protective Effect of Paeoniflorin Against Coronary Heart Disease in Mice via Inhibiting the C3-Cfd-C3aR Pathway
Source: Int J Mol Sci. 2026 Jul 13;27(14):6236. doi: 10.3390/ijms27146236 (PMC13410309; doi:10.3390/ijms27146236)
Supplement: Supplementary file 1 [file ijms-27-06236-s001.zip › Supplementary Materials/ijms-4276706_Proteomics_Dataset/8-KEGG_pathway_image/Model-vs-Paeoniflorin/mmu04640.html]

KEGG PATHWAY: Hematopoietic cell lineage - Mus musculus (house mouse)


# Hematopoietic cell lineage - Mus musculus (house mouse)


[
Pathway menu
| Organism menu
| Pathway entry
| Show description
| Download
| Help
]

Blood-cell development progresses from a hematopoietic stem cell (HSC), which can undergo either self-renewal or differentiation into a multilineage committed progenitor cell: a common lymphoid progenitor (CLP) or a common myeloid progenitor (CMP). A CLP gives rise to the lymphoid lineage of white blood cells or leukocytes-the natural killer (NK) cells and the T and B lymphocytes. A CMP gives rise to the myeloid lineage, which comprises the rest of the leukocytes, the erythrocytes (red blood cells), and the megakaryocytes that produce platelets important in blood clotting. Cells undergoing these differentiation process express a stage- and lineage-specific set of surface markers. Therefore cellular stages are identified by the specific expression patterns of these genes.


##### Option

Scale:


100%

Image resolution:


 High

##### Background color

Organism

##### Search

##### ID search

##### Color

##### Related Brite

- 04090  CD molecules


KGML

Image (png) file 1x

Image (png) file 2x
