# Supplementary material for: Multi-Omics and Experimental Validation Reveal the Protective Effect of Paeoniflorin Against Coronary Heart Disease in Mice via Inhibiting the C3-Cfd-C3aR Pathway
Source: Int J Mol Sci. 2026 Jul 13;27(14):6236. doi: 10.3390/ijms27146236 (PMC13410309; doi:10.3390/ijms27146236)
Supplement: Supplementary file 1 [file ijms-27-06236-s001.zip › Supplementary Materials/ijms-4276706_Proteomics_Dataset/8-KEGG_pathway_image/Model-vs-Paeoniflorin/mmu05417.html]

KEGG PATHWAY: Lipid and atherosclerosis - Mus musculus (house mouse)


# Lipid and atherosclerosis - Mus musculus (house mouse)


[
Pathway menu
| Organism menu
| Pathway entry
| Show description
| Download
| Help
]

Atherosclerosis is a chronic inflammatory disease marked by a narrowing of the arteries from lipid-rich plaques present within the walls of arterial blood vessels. It represents the root cause of the majority of cardiovascular diseases (CVDs) and their complications, including conditions such as coronary artery disease, myocardial infarction and stroke. An elevated level of low density lipoprotein (LDL) cholesterol constitutes a major risk factor for genesis of atherosclerosis. LDL can accumulate within the blood vessel wall and undergo modification by oxidation. Oxidized LDL (oxLDL) leads to endothelial dysfunction leading to expression of adhesion molecules and recruitment of monocyte in subendothelial space. The monocytes proliferate, differentiate into macrophages, and take up the lipoproteins, forming cholesterol-engorged "foam cells." With the time, the foam cells die, leaving a 'necrotic core' of crystalline cholesterol and cell debris. Smooth muscle cells proliferate and migrate into the region, laying down a protective cap over the lesion. Lectin-like oxidized low-density lipoprotein receptor-1 (LOX-1), which mediates the recognition and internalization of oxLDL, is involved in all of these events critical in the pathogenesis of atherosclerosis.


##### Option

Scale:


100%

Image resolution:


 High

##### Background color

Organism

##### Search

##### ID search

##### Color


KGML

Image (png) file 1x

Image (png) file 2x
