# Supplementary material for: Multi-Omics and Experimental Validation Reveal the Protective Effect of Paeoniflorin Against Coronary Heart Disease in Mice via Inhibiting the C3-Cfd-C3aR Pathway
Source: Int J Mol Sci. 2026 Jul 13;27(14):6236. doi: 10.3390/ijms27146236 (PMC13410309; doi:10.3390/ijms27146236)
Supplement: Supplementary file 1 [file ijms-27-06236-s001.zip › Supplementary Materials/ijms-4276706_Proteomics_Dataset/8-KEGG_pathway_image/Model-vs-Paeoniflorin/mmu05332.html]

KEGG PATHWAY: Graft-versus-host disease - Mus musculus (house mouse)


# Graft-versus-host disease - Mus musculus (house mouse)


[
Pathway menu
| Organism menu
| Pathway entry
| Show description
| Download
| Help
]

Graft-versus-host disease (GVHD) is a lethal complication of allogeneic hematopoietic stem cell transplantation (HSCT) where immunocompetent donor T cells attack the genetically disparate host cells. GVHD pathophysiology can be summerized in a three-step process. Step 1 involves the development of an inflammatory milieu resulting from damage in the host tissues induced by the preparative chemotherapy or radiotherapy regimen. Damaged tissues secrete inflammatory cytokines, including interleukin 1 (IL-1), and tumor necrosis factor (TNF-alpha ). During step 2, antigen-presenting cells (APCs) trigger the activation of donor-derived T cells, which induce further T-cell expansion, induce cytotoxic T lymphocytes (CTL) and natural killer (NK) cells responses and prime additional mononuclear phagocytes to produce TNF-alpha and IL-1. Also, nitric oxide (NO) is produced by activated macrophages, and it may contribute to the tissue damage seen during step 3. During step 3, the effector phase, activated CTL and NK cells mediate cytotoxicity against target host cells through Fas-Fas ligand interactions and perforin-granzyme B.


##### Option

Scale:


100%

Image resolution:


 High

##### Background color

Organism

##### Search

##### ID search

##### Color


KGML

Image (png) file 1x

Image (png) file 2x
