# Supplementary material for: Multi-Omics and Experimental Validation Reveal the Protective Effect of Paeoniflorin Against Coronary Heart Disease in Mice via Inhibiting the C3-Cfd-C3aR Pathway
Source: Int J Mol Sci. 2026 Jul 13;27(14):6236. doi: 10.3390/ijms27146236 (PMC13410309; doi:10.3390/ijms27146236)
Supplement: Supplementary file 1 [file ijms-27-06236-s001.zip › Supplementary Materials/ijms-4276706_Proteomics_Dataset/8-KEGG_pathway_image/Model-vs-Paeoniflorin/mmu04722.html]

KEGG PATHWAY: Neurotrophin signaling pathway - Mus musculus (house mouse)


# Neurotrophin signaling pathway - Mus musculus (house mouse)


[
Pathway menu
| Organism menu
| Pathway entry
| Show description
| Download
| Help
]

Neurotrophins are a family of trophic factors involved in differentiation and survival of neural cells. The neurotrophin family consists of nerve growth factor (NGF), brain derived neurotrophic factor (BDNF), neurotrophin 3 (NT-3), and neurotrophin 4 (NT-4). Neurotrophins exert their functions through engagement of Trk tyrosine kinase receptors or p75 neurotrophin receptor (p75NTR). Neurotrophin/Trk signaling is regulated by connecting a variety of intracellular signaling cascades, which include MAPK pathway, PI-3 kinase pathway, and PLC pathway, transmitting positive signals like enhanced survival and growth. On the other hand, p75NTR transmits both positive and nagative signals. These signals play an important role for neural development and additional higher-order activities such as learning and memory.


##### Option

Scale:


100%

Image resolution:


 High

##### Background color

Organism

##### Search

##### ID search

##### Color


KGML

Image (png) file 1x

Image (png) file 2x
