# Supplementary material for: Multi-Omics and Experimental Validation Reveal the Protective Effect of Paeoniflorin Against Coronary Heart Disease in Mice via Inhibiting the C3-Cfd-C3aR Pathway
Source: Int J Mol Sci. 2026 Jul 13;27(14):6236. doi: 10.3390/ijms27146236 (PMC13410309; doi:10.3390/ijms27146236)
Supplement: Supplementary file 1 [file ijms-27-06236-s001.zip › Supplementary Materials/ijms-4276706_Proteomics_Dataset/8-KEGG_pathway_image/Model-vs-Paeoniflorin/mmu05133.html]

KEGG PATHWAY: Pertussis - Mus musculus (house mouse)


# Pertussis - Mus musculus (house mouse)


[
Pathway menu
| Organism menu
| Pathway entry
| Show description
| Download
| Help
]

Pertussis, also known as whooping cough, is an acute respiratory infectious disease caused by a bacteria called Bordetella Pertussis. The characteristic symptoms are paroxysmal cough, inspiratory wheezing and post-tussive vomiting.
Following the inhalation of respiratory secretions from an infected individual, bacteria enter the upper respiratory tract and adhere to epithelial cells. Several adhesion factors have been implicated: the filamentous hemagglutinin (FHA), fimbriae, and pertactin (Prn).
Pertussis toxin (Ptx) and adenylate cyclase toxin (ACT) have been identified so far as major protein toxins of B. pertussis. PTX is a hexameric AB5-type exotoxin. Catalytic A subunit catalyzes the ADP-ribosylation of the Gi subunits of the heterotrimeric G protein, then inhibits multiple downstream pathways. ACT is able to penetrate the cytoplasmic membrane of host cells and becomes activated through the cleavage and the binding of calmodulin (CaM). Activated ACT converts ATP to cyclic AMP and subverts cellular signaling pathways.


##### Option

Scale:


100%

Image resolution:


 High

##### Background color

Organism

##### Search

##### ID search

##### Color


KGML

Image (png) file 1x

Image (png) file 2x
