# Supplementary material for: Multi-Omics and Experimental Validation Reveal the Protective Effect of Paeoniflorin Against Coronary Heart Disease in Mice via Inhibiting the C3-Cfd-C3aR Pathway
Source: Int J Mol Sci. 2026 Jul 13;27(14):6236. doi: 10.3390/ijms27146236 (PMC13410309; doi:10.3390/ijms27146236)
Supplement: Supplementary file 1 [file ijms-27-06236-s001.zip › Supplementary Materials/ijms-4276706_Proteomics_Dataset/8-KEGG_pathway_image/Model-vs-Paeoniflorin/mmu04610.html]

KEGG PATHWAY: Complement and coagulation cascades - Mus musculus (house mouse)


# Complement and coagulation cascades - Mus musculus (house mouse)


[
Pathway menu
| Organism menu
| Pathway entry
| Show description
| Download
| Help
]

The complement system is a proteolytic cascade in blood plasma and a mediator of innate immunity, a nonspecific defense mechanism against pathogens. There are three pathways of complement activation: the classical pathway, the lectin pathway, and the alternative pathway. All of these pathways generate a crucial enzymatic activity that, in turn, generates the effector molecules of complement. The main consequences of complement activation are the opsonization of pathogens, the recruitment of inflammatory and immunocompetent cells, and the direct killing of pathogens. Blood coagulation is another series of proenzyme-to-serine protease conversions, culminating the formation of thrombin, the enzyme responsible for the conversion of soluble fibrinogen to the insoluble fibrin clot. Protease-activated receptors, such as those activated by thrombin, are members of G protein-coupled receptors and function as a mediator of innate immunity. The kallikrein-kinin system is an endogenous metabolic cascade, triggering of which results in the release of vasoactive kinins (bradykinin-related peptides). Kinin peptides are implicated in many physiological and pathological processes including the regulation of blood pressure and sodium homeostasis, inflammatory processes, and the cardioprotective effects of preconditioning.


##### Option

Scale:


100%

Image resolution:


 High

##### Background color

Organism

##### Search

##### ID search

##### Color


KGML

Image (png) file 1x

Image (png) file 2x
