# Supplementary material for: Multi-Omics and Experimental Validation Reveal the Protective Effect of Paeoniflorin Against Coronary Heart Disease in Mice via Inhibiting the C3-Cfd-C3aR Pathway
Source: Int J Mol Sci. 2026 Jul 13;27(14):6236. doi: 10.3390/ijms27146236 (PMC13410309; doi:10.3390/ijms27146236)
Supplement: Supplementary file 1 [file ijms-27-06236-s001.zip › Supplementary Materials/ijms-4276706_Proteomics_Dataset/8-KEGG_pathway_image/Model-vs-Paeoniflorin/mmu05168.html]

KEGG PATHWAY: Herpes simplex virus 1 infection - Mus musculus (house mouse)


# Herpes simplex virus 1 infection - Mus musculus (house mouse)


[
Pathway menu
| Organism menu
| Pathway entry
| Show description
| Download
| Help
]

Herpes simplex virus 1(HSV-1) is a common human pathogen, which initially infects orofacial mucosal surfaces. The virus replicates in epithelial cells at these sites, causing clinically overt disease characterized by vesicular lesions. HSV-1 then penetrates to the nervous system and establishes latency in sensory neurons. Throughout the lifetime of the host, HSV-1 may reactivate from its latent state and reinitiate a lytic infection cycle. HSV-1 has developed various mechanisms to escape its host innate immune responses and increased its capacity to replicate and to persist. ICP34.5, ICP0, ICP27, Us11 and Vhs (UL41) are the proteins involved in viral interference.


##### Option

Scale:


100%

Image resolution:


 High

##### Background color

Organism

##### Search

##### ID search

##### Color


KGML

Image (png) file 1x

Image (png) file 2x
