# Supplementary material for: Multi-Omics and Experimental Validation Reveal the Protective Effect of Paeoniflorin Against Coronary Heart Disease in Mice via Inhibiting the C3-Cfd-C3aR Pathway
Source: Int J Mol Sci. 2026 Jul 13;27(14):6236. doi: 10.3390/ijms27146236 (PMC13410309; doi:10.3390/ijms27146236)
Supplement: Supplementary file 1 [file ijms-27-06236-s001.zip › Supplementary Materials/ijms-4276706_Proteomics_Dataset/8-KEGG_pathway_image/Model-vs-Paeoniflorin/mmu04145.html]

KEGG PATHWAY: Phagosome - Mus musculus (house mouse)


# Phagosome - Mus musculus (house mouse)


[
Pathway menu
| Organism menu
| Pathway entry
| Show description
| Download
| Help
]

Phagocytosis is the process of taking in relatively large particles by a cell, and is a central mechanism in the tissue remodeling, inflammation, and defense against infectious agents. A phagosome is formed when the specific receptors on the phagocyte surface recognize ligands on the particle surface. After formation, nascent phagosomes progressively acquire digestive characteristics. This maturation of phagosomes involves regulated interaction with the other membrane organelles, including recycling endosomes, late endosomes and lysosomes. The fusion of phagosomes and lysosomes releases toxic products that kill most bacteria and degrade them into fragments. However, some bacteria have strategies to escape the bactericidal mechanisms associated with phagocytosis and survive within host phagocytes.


##### Option

Scale:


100%

Image resolution:


 High

##### Background color

Organism

##### Search

##### ID search

##### Color


KGML

Image (png) file 1x

Image (png) file 2x
