# Supplementary material for: Multi-Omics and Experimental Validation Reveal the Protective Effect of Paeoniflorin Against Coronary Heart Disease in Mice via Inhibiting the C3-Cfd-C3aR Pathway
Source: Int J Mol Sci. 2026 Jul 13;27(14):6236. doi: 10.3390/ijms27146236 (PMC13410309; doi:10.3390/ijms27146236)
Supplement: Supplementary file 1 [file ijms-27-06236-s001.zip › Supplementary Materials/ijms-4276706_Proteomics_Dataset/8-KEGG_pathway_image/Model-vs-Paeoniflorin/mmu04144.html]

KEGG PATHWAY: Endocytosis - Mus musculus (house mouse)


# Endocytosis - Mus musculus (house mouse)


[
Pathway menu
| Organism menu
| Pathway entry
| Show description
| Download
| Help
]

Endocytosis is a mechanism for cells to remove ligands, nutrients, and plasma membrane (PM) proteins, and lipids from the cell surface, bringing them into the cell interior. Transmembrane proteins entering through clathrin-dependent endocytosis (CDE) have sequences in their cytoplasmic domains that bind to the APs (adaptor-related protein complexes) and enable their rapid removal from the PM. In addition to APs and clathrin, there are numerous accessory proteins including dynamin. Depending on the various proteins that enter the endosome membrane, these cargoes are sorted to distinct destinations. Some cargoes, such as nutrient receptors, are recycled back to the PM. Ubiquitylated membrane proteins, such as activated growth-factor receptors, are sorted into intraluminal vesicles and eventually end up in the lysosome lumen via multivesicular endosomes (MVEs). There are distinct mechanisms of clathrin-independent endocytosis (CIE) depending upon the cargo and the cell type.


##### Option

Scale:


100%

Image resolution:


 High

##### Background color

Organism

##### Search

##### ID search

##### Color


KGML

Image (png) file 1x

Image (png) file 2x
