# Supplementary material for: Multi-Omics and Experimental Validation Reveal the Protective Effect of Paeoniflorin Against Coronary Heart Disease in Mice via Inhibiting the C3-Cfd-C3aR Pathway
Source: Int J Mol Sci. 2026 Jul 13;27(14):6236. doi: 10.3390/ijms27146236 (PMC13410309; doi:10.3390/ijms27146236)
Supplement: Supplementary file 1 [file ijms-27-06236-s001.zip › Supplementary Materials/ijms-4276706_Proteomics_Dataset/8-KEGG_pathway_image/Model-vs-Paeoniflorin/mmu05169.html]

KEGG PATHWAY: Epstein-Barr virus infection - Mus musculus (house mouse)


# Epstein-Barr virus infection - Mus musculus (house mouse)


[
Pathway menu
| Organism menu
| Pathway entry
| Show description
| Download
| Help
]

Epstein-Barr virus (EBV) is a gamma-herpes virus that widely infects human populations predominantly at an early age but remains mostly asymptomatic. EBV has been linked to a wide spectrum of human malignancies, including nasopharyngeal carcinoma and other hematologic cancers, like Hodgkin's lymphoma, Burkitt's lymphoma (BL), B-cell immunoblastic lymphoma in HIV patients, and posttransplant-associated lymphoproliferative diseases. EBV has the unique ability to establish life-long latent infection in primary human B lymphocytes. During latent infection, EBV expresses a small subset of genes, including 6 nuclear antigens (EBNA-1, -2, -3A, -3B, -3C, and -LP), 3 latent membrane proteins (LMP-1, -2A, and -2B), 2 small noncoding RNAs (EBER-1 and 2). On the basis of these latent gene expression, three different latency patterns associated with the types of cancers are recognized.


##### Option

Scale:


100%

Image resolution:


 High

##### Background color

Organism

##### Search

##### ID search

##### Color


KGML

Image (png) file 1x

Image (png) file 2x
