# Supplementary material for: Multi-Omics and Experimental Validation Reveal the Protective Effect of Paeoniflorin Against Coronary Heart Disease in Mice via Inhibiting the C3-Cfd-C3aR Pathway
Source: Int J Mol Sci. 2026 Jul 13;27(14):6236. doi: 10.3390/ijms27146236 (PMC13410309; doi:10.3390/ijms27146236)
Supplement: Supplementary file 1 [file ijms-27-06236-s001.zip › Supplementary Materials/ijms-4276706_Proteomics_Dataset/8-KEGG_pathway_image/Model-vs-Paeoniflorin/mmu04611.html]

KEGG PATHWAY: Platelet activation - Mus musculus (house mouse)


# Platelet activation - Mus musculus (house mouse)


[
Pathway menu
| Organism menu
| Pathway entry
| Show description
| Download
| Help
]

Platelets play a key and beneficial role for primary hemostasis on the disruption of the integrity of vessel wall. Platelet adhesion and activation at sites of vascular wall injury is initiated by adhesion to adhesive macromolecules, such as collagen and von Willebrand factor (vWF), or by soluble platelet agonists, such as ADP, thrombin, and thromboxane A2. Different receptors are stimulated by various agonists, almost converging in increasing intracellular Ca2+ concentration that stimulate platelet shape change and granule secretion and ultimately induce the "inside-out" signaling process leading to activation of the ligand-binding function of integrin alpha IIb beta 3. Binding of alpha IIb beta 3 to its ligands, mainly fibrinogen, mediates platelet adhesion and aggregation and triggers "outside-in" signaling, resulting in platelet spreading, additional granule secretion, stabilization of platelet adhesion and aggregation, and clot retraction.


##### Option

Scale:


100%

Image resolution:


 High

##### Background color

Organism

##### Search

##### ID search

##### Color


KGML

Image (png) file 1x

Image (png) file 2x
