# Supplementary material for: Multi-Omics and Experimental Validation Reveal the Protective Effect of Paeoniflorin Against Coronary Heart Disease in Mice via Inhibiting the C3-Cfd-C3aR Pathway
Source: Int J Mol Sci. 2026 Jul 13;27(14):6236. doi: 10.3390/ijms27146236 (PMC13410309; doi:10.3390/ijms27146236)
Supplement: Supplementary file 1 [file ijms-27-06236-s001.zip › Supplementary Materials/ijms-4276706_Proteomics_Dataset/8-KEGG_pathway_image/Model-vs-Paeoniflorin/mmu04670.html]

KEGG PATHWAY: Leukocyte transendothelial migration - Mus musculus (house mouse)


# Leukocyte transendothelial migration - Mus musculus (house mouse)


[
Pathway menu
| Organism menu
| Pathway entry
| Show description
| Download
| Help
]

Leukocyte migaration from the blood into tissues is vital for immune surveillance and inflammation. During this diapedesis of leukocytes, the leukocytes bind to endothelial cell adhesion molecules (CAM) and then migrate across the vascular endothelium. A leukocyte adherent to CAMs on the endothelial cells moves forward by leading-edge protrusion and retraction of its tail. In this process, alphaL /beta2 integrin activates through Vav1, RhoA, which subsequently activates the kinase p160ROCK. ROCK activation leads to MLC phosphorylation, resulting in retraction of the actin cytoskeleton. Moreover, Leukocytes activate endothelial cell signals that stimulate endothelial cell retraction during localized dissociation of the endothelial cell junctions. ICAM-1-mediated signals activate an endothelial cell calcium flux and PKC, which are required for ICAM-1 dependent leukocyte migration. VCAM-1 is involved in the opening of the "endothelial passage" through which leukocytes can extravasate. In this regard, VCAM-1 ligation induces NADPH oxidase activation and the production of reactive oxygen species (ROS) in a Rac-mediated manner, with subsequent activation of matrix metallopoteinases and loss of VE-cadherin-mediated adhesion.


##### Option

Scale:


100%

Image resolution:


 High

##### Background color

Organism

##### Search

##### ID search

##### Color


KGML

Image (png) file 1x

Image (png) file 2x
