# Supplementary material for: Multi-Omics and Experimental Validation Reveal the Protective Effect of Paeoniflorin Against Coronary Heart Disease in Mice via Inhibiting the C3-Cfd-C3aR Pathway
Source: Int J Mol Sci. 2026 Jul 13;27(14):6236. doi: 10.3390/ijms27146236 (PMC13410309; doi:10.3390/ijms27146236)
Supplement: Supplementary file 1 [file ijms-27-06236-s001.zip › Supplementary Materials/ijms-4276706_Proteomics_Dataset/8-KEGG_pathway_image/Model-vs-Paeoniflorin/mmu05165.html]

KEGG PATHWAY: Human papillomavirus infection - Mus musculus (house mouse)


# Human papillomavirus infection - Mus musculus (house mouse)


[
Pathway menu
| Organism menu
| Pathway entry
| Show description
| Download
| Help
]

Human papillomavirus (HPV) is a non-enveloped, double-stranded DNA virus. HPV infect mucoal and cutaneous epithelium resulting in several types of pathologies, most notably, cervical cancer. All types of HPV share a common genomic structure and encode eight proteins: E1, E2, E4, E5, E6, and E7 (early) and L1 and L2 (late). It has been demonstrated that E1 and E2 are involved in viral transcription and replication. The functions of the E4 protein is not yet fully understood. E5, E6, and E7 act as oncoproteins. E5 inhibits the V-ATPase, prolonging EGFR signaling and thereby promoting cell proliferation. The expression of E6 and E7 not only inhibits the tumor suppressors p53 and Rb, but also alters additional signalling pathways. Among these pathways, PI3K/Akt signalling cascade plays a very important role in HPV-induced carcinogenesis. The L1 and L2 proteins form icosahedral capsids for progeny virion generation.


##### Option

Scale:


100%

Image resolution:


 High

##### Background color

Organism

##### Search

##### ID search

##### Color


KGML

Image (png) file 1x

Image (png) file 2x
