# Supplementary material for: Multi-Omics and Experimental Validation Reveal the Protective Effect of Paeoniflorin Against Coronary Heart Disease in Mice via Inhibiting the C3-Cfd-C3aR Pathway
Source: Int J Mol Sci. 2026 Jul 13;27(14):6236. doi: 10.3390/ijms27146236 (PMC13410309; doi:10.3390/ijms27146236)
Supplement: Supplementary file 1 [file ijms-27-06236-s001.zip › Supplementary Materials/ijms-4276706_Proteomics_Dataset/8-KEGG_pathway_image/Model-vs-Paeoniflorin/mmu04666.html]

KEGG PATHWAY: Fc gamma R-mediated phagocytosis - Mus musculus (house mouse)


# Fc gamma R-mediated phagocytosis - Mus musculus (house mouse)


[
Pathway menu
| Organism menu
| Pathway entry
| Show description
| Download
| Help
]

Phagocytosis plays an essential role in host-defense mechanisms through the uptake and destruction of infectious pathogens. Specialized cell types including macrophages, neutrophils, and monocytes take part in this process in higher organisms. After opsonization with antibodies (IgG), foreign extracellular materials are recognized by Fc gamma receptors. Cross-linking of Fc gamma receptors initiates a variety of signals mediated by tyrosine phosphorylation of multiple proteins, which lead through the actin cytoskeleton rearrangements and membrane remodeling to the formation of phagosomes. Nascent phagosomes undergo a process of maturation that involves fusion with lysosomes. The acquisition of lysosomal proteases and release of reactive oxygen species are crucial for digestion of engulfed materials in phagosomes.


##### Option

Scale:


100%

Image resolution:


 High

##### Background color

Organism

##### Search

##### ID search

##### Color


KGML

Image (png) file 1x

Image (png) file 2x
