# Supplementary material for: Multi-Omics and Experimental Validation Reveal the Protective Effect of Paeoniflorin Against Coronary Heart Disease in Mice via Inhibiting the C3-Cfd-C3aR Pathway
Source: Int J Mol Sci. 2026 Jul 13;27(14):6236. doi: 10.3390/ijms27146236 (PMC13410309; doi:10.3390/ijms27146236)
Supplement: Supplementary file 1 [file ijms-27-06236-s001.zip › Supplementary Materials/ijms-4276706_Proteomics_Dataset/8-KEGG_pathway_image/Model-vs-Paeoniflorin/mmu05132.html]

KEGG PATHWAY: Salmonella infection - Mus musculus (house mouse)


# Salmonella infection - Mus musculus (house mouse)


[
Pathway menu
| Organism menu
| Pathway entry
| Show description
| Download
| Help
]

Salmonella infection usually presents as a self-limiting gastroenteritis or the more severe typhoid fever and bacteremia. The common disease-causing Salmonella species in human is a single species, Salmonella enterica, which has numerous serovars.
Following intestinal colonization Salmonella inject effector proteins into the host cells using a type III secretion system (T3SS), T3SS1. Then a small group of effector proteins induce rearrangement of the actin cytoskeleton resulting in membrane ruffles and rapid internalization of the bacteria.The T3SS2 is responsible for translocating effector proteins that direct Salmonella-containing vacuole (SCV) maturation. The majority of the bacteria are known to survive and replicate in SCV.


##### Option

Scale:


100%

Image resolution:


 High

##### Background color

Organism

##### Search

##### ID search

##### Color


KGML

Image (png) file 1x

Image (png) file 2x
