# Supplementary material for: Multi-Omics and Experimental Validation Reveal the Protective Effect of Paeoniflorin Against Coronary Heart Disease in Mice via Inhibiting the C3-Cfd-C3aR Pathway
Source: Int J Mol Sci. 2026 Jul 13;27(14):6236. doi: 10.3390/ijms27146236 (PMC13410309; doi:10.3390/ijms27146236)
Supplement: Supplementary file 1 [file ijms-27-06236-s001.zip › Supplementary Materials/ijms-4276706_Proteomics_Dataset/8-KEGG_pathway_image/Control-vs-Model/mmu03010.html]

KEGG PATHWAY: Ribosome - Mus musculus (house mouse)


# Ribosome - Mus musculus (house mouse)


[
Pathway menu
| Organism menu
| Pathway entry
| Download
| Help
]


##### Option

Scale:


100%

Image resolution:


 High

##### Background color

Organism

##### Search

##### ID search

##### Color

##### Related Brite

- 03011  Ribosome

##### Related Brite table

- br01610  Ribosomal proteins


KGML

Image (png) file 1x

Image (png) file 2x
