# Supplementary material for: Multi-Omics and Experimental Validation Reveal the Protective Effect of Paeoniflorin Against Coronary Heart Disease in Mice via Inhibiting the C3-Cfd-C3aR Pathway
Source: Int J Mol Sci. 2026 Jul 13;27(14):6236. doi: 10.3390/ijms27146236 (PMC13410309; doi:10.3390/ijms27146236)
Supplement: Supplementary file 1 [file ijms-27-06236-s001.zip › Supplementary Materials/ijms-4276706_Proteomics_Dataset/8-KEGG_pathway_image/Control-vs-Model/mmu05140.html]

KEGG PATHWAY: Leishmaniasis - Mus musculus (house mouse)


# Leishmaniasis - Mus musculus (house mouse)


[
Pathway menu
| Organism menu
| Pathway entry
| Show description
| Download
| Help
]

Leishmania is an intracellular protozoan parasite of macrophages that causes visceral, mucosal, and cutaneous diseases. The parasite is transmitted to humans by sandflies, where they survive and proliferate intracellularly by deactivating the macrophage. Successful infection of Leishmania is achieved by alteration of signaling events in the host cell, leading to enhanced production of the autoinhibitory molecules like TGF-beta and decreased induction of cytokines such as IL12 for protective immunity. Nitric oxide production is also inhibited. In addition, defective expression of major histocompatibility complex (MHC) genes silences subsequent T cell activation mediated by macrophages, resulting in abnormal immune responses.


##### Option

Scale:


100%

Image resolution:


 High

##### Background color

Organism

##### Search

##### ID search

##### Color


KGML

Image (png) file 1x

Image (png) file 2x
