# Supplementary material for: Multi-Omics and Experimental Validation Reveal the Protective Effect of Paeoniflorin Against Coronary Heart Disease in Mice via Inhibiting the C3-Cfd-C3aR Pathway
Source: Int J Mol Sci. 2026 Jul 13;27(14):6236. doi: 10.3390/ijms27146236 (PMC13410309; doi:10.3390/ijms27146236)
Supplement: Supplementary file 1 [file ijms-27-06236-s001.zip › Supplementary Materials/ijms-4276706_Proteomics_Dataset/8-KEGG_pathway_image/Control-vs-Model/mmu03060.html]

KEGG PATHWAY: Protein export - Mus musculus (house mouse)


# Protein export - Mus musculus (house mouse)


[
Pathway menu
| Organism menu
| Pathway entry
| Show description
| Download
| Help
]

The protein export is the active transport of proteins from the cytoplasm to the exterior of the cell, or to the periplasmic compartment in Gram-negative bacteria. The sec dependent pathway is the general protein export system that transports newly synthesized proteins into or across the cell membrane. The translocation channel is formed from a conserved trimeric membrane protein complex, called the Sec61/SecY complex. The twin-arginine translocation (Tat) pathway is another protein transport system that transports folded proteins in bacteria, archaea, and chloroplasts. Many Tat systems comprise three functionally different membrane proteins, TatA, TatB, and TatC, but TatA and TatE seem to have overlapping functions, with TatA having by far the more important role.


##### Option

Scale:


100%

Image resolution:


 High

##### Background color

Organism

##### Search

##### ID search

##### Color

##### Related Brite

- 02044  Secretion system


KGML

Image (png) file 1x

Image (png) file 2x
