# Supplementary material for: Multi-Omics and Experimental Validation Reveal the Protective Effect of Paeoniflorin Against Coronary Heart Disease in Mice via Inhibiting the C3-Cfd-C3aR Pathway
Source: Int J Mol Sci. 2026 Jul 13;27(14):6236. doi: 10.3390/ijms27146236 (PMC13410309; doi:10.3390/ijms27146236)
Supplement: Supplementary file 1 [file ijms-27-06236-s001.zip › Supplementary Materials/ijms-4276706_Proteomics_Dataset/8-KEGG_pathway_image/Control-vs-Model/mmu00510.html]

KEGG PATHWAY: N-Glycan biosynthesis - Mus musculus (house mouse)


# N-Glycan biosynthesis - Mus musculus (house mouse)


[
Pathway menu
| Organism menu
| Pathway entry
| Show description
| Download
| Help
]

N-glycans or asparagine-linked glycans are major constituents of glycoproteins in eukaryotes. N-glycans are covalently attached to asparagine with the consensus sequence of Asn-X-Ser/Thr by an N-glycosidic bond, GlcNAc b1- Asn. Biosynthesis of N-glycans begins on the cytoplasmic face of the ER membrane with the transferase reaction of UDP-GlcNAc and the lipid-like precursor P-Dol (dolichol phosphate) to generate GlcNAc a1- PP-Dol. After sequential addition of monosaccharides by ALG glycosyltransferases [MD:M00055], the N-glycan precursor is attached by the OST (oligosaccharyltransferase) complex to the polypeptide chain that is being synthesized and translocated through the ER membrane. The protein-bound N-glycan precursor is subsequently trimmed, extended, and modified in the ER and Golgi by a complex series of reactions catalyzed by membrane-bound glycosidases and glycosyltransferases. N-glycans thus synthesized are classified into three types: high-mannose type, complex type, and hybrid type. Defects in N-glycan biosynthesis lead to a variety of human diseases known as congenital disorders of glycosylation [DS:H00118 H00119].


##### Option

Scale:


100%

Image resolution:


 High

##### Background color

Organism

##### Search

##### ID search

##### Color

##### Module

Complete only
Including 1 block missing
Including any incomplete

- Pathway modules
  - Glycan metabolism
    - Glycan biosynthesis
      - M00055
        N-glycan precursor biosynthesis
      - M00072
        N-glycosylation by oligosaccharyltransferase
      - M00073
        N-glycan precursor trimming
      - M00074
        N-glycan biosynthesis, high-mannose type
      - M00075
        N-glycan biosynthesis, complex type


KGML

Image (png) file 1x

Image (png) file 2x
