# Supplementary material for: Multi-Omics and Experimental Validation Reveal the Protective Effect of Paeoniflorin Against Coronary Heart Disease in Mice via Inhibiting the C3-Cfd-C3aR Pathway
Source: Int J Mol Sci. 2026 Jul 13;27(14):6236. doi: 10.3390/ijms27146236 (PMC13410309; doi:10.3390/ijms27146236)
Supplement: Supplementary file 1 [file ijms-27-06236-s001.zip › Supplementary Materials/ijms-4276706_Proteomics_Dataset/8-KEGG_pathway_image/Control-vs-Model/mmu04928.html]

KEGG PATHWAY: Parathyroid hormone synthesis, secretion and action - Mus musculus (house mouse)


# Parathyroid hormone synthesis, secretion and action - Mus musculus (house mouse)


[
Pathway menu
| Organism menu
| Pathway entry
| Show description
| Download
| Help
]

Parathyroid hormone (PTH) is a key regulator of calcium and phosphorus homeostasis. The principal regulators of PTH secretion are extracellular ionized calcium (Ca2+) and 1,25-dihydroxyvitamin D (1,25(OH)2D3). Under conditions of dietary Ca restriction, a decrement in serum Ca concentration induces release of PTH from the parathyroid gland. PTH acts on bone and kidney to stimulate bone turnover, increase the circulating levels of 1,25(OH)2D3 and calcium and inhibit the reabsorption of phosphate from the glomerular filtrate. This hormone exerts its actions via binding to the PTH/PTH-related peptide receptor (PTH1R). PTH1R primarily activates two sub-types of heterotrimeric Gproteins: Gs and Gq , which in turn regulate the activity of adenylyl cyclases and phospholipase C (PLC) that control the flow of cAMP/PKA and IP/PKC signaling cascades, respectively.


##### Option

Scale:


100%

Image resolution:


 High

##### Background color

Organism

##### Search

##### ID search

##### Color


KGML

Image (png) file 1x

Image (png) file 2x
