# Supplementary material for: Multi-Omics and Experimental Validation Reveal the Protective Effect of Paeoniflorin Against Coronary Heart Disease in Mice via Inhibiting the C3-Cfd-C3aR Pathway
Source: Int J Mol Sci. 2026 Jul 13;27(14):6236. doi: 10.3390/ijms27146236 (PMC13410309; doi:10.3390/ijms27146236)
Supplement: Supplementary file 1 [file ijms-27-06236-s001.zip › Supplementary Materials/ijms-4276706_Proteomics_Dataset/8-KEGG_pathway_image/Control-vs-Model/mmu04061.html]

KEGG PATHWAY: Viral protein interaction with cytokine and cytokine receptor - Mus musculus (house mouse)


# Viral protein interaction with cytokine and cytokine receptor - Mus musculus (house mouse)


[
Pathway menu
| Organism menu
| Pathway entry
| Show description
| Download
| Help
]

Viruses have evolved diverse mechanisms to evade detection and destruction by the immune system, including copying and repurposing host cytokine and cytokine receptor genes. Viral cytokines and cytokine receptors, together with a group of structurally unique, soluble, cytokine-binding or cytokine receptor-binding proteins, represent the three major molecular strategies for subversion and modulation of the host cytokine networks mainly in large DNA viruses. Viral cytokines and cytokine receptor homologs, including other binding proteins, may activate or inhibit cytokine signaling and possibly affect different aspects of immunity.


##### Option

Scale:


100%

Image resolution:


 High

##### Background color

Organism

##### Search

##### ID search

##### Color

##### Related Brite table

- br03223  Viral network perturbations


KGML

Image (png) file 1x

Image (png) file 2x
