# Supplementary material for: Multi-Omics and Experimental Validation Reveal the Protective Effect of Paeoniflorin Against Coronary Heart Disease in Mice via Inhibiting the C3-Cfd-C3aR Pathway
Source: Int J Mol Sci. 2026 Jul 13;27(14):6236. doi: 10.3390/ijms27146236 (PMC13410309; doi:10.3390/ijms27146236)
Supplement: Supplementary file 1 [file ijms-27-06236-s001.zip › Supplementary Materials/ijms-4276706_Proteomics_Dataset/8-KEGG_pathway_image/Control-vs-Model/mmu04979.html]

KEGG PATHWAY: Cholesterol metabolism - Mus musculus (house mouse)


# Cholesterol metabolism - Mus musculus (house mouse)


[
Pathway menu
| Organism menu
| Pathway entry
| Show description
| Download
| Help
]

Cholesterol is an essential component of mammalian cell membranes as well as a precursor of bile acids, vitamin D and steroid hormones. Cholesterol homeostasis in humans is regulated by well-balanced mechanisms of intestinal uptake, endogenous synthesis, transport in lipoprotein particles, and biliary excretion. Its abnormal metabolism can lead to increased risk for various endocrine disorders and cardiovascular diseases.


##### Option

Scale:


100%

Image resolution:


 High

##### Background color

Organism

##### Search

##### ID search

##### Color


KGML

Image (png) file 1x

Image (png) file 2x
