# Supplementary material for: Multi-Omics and Experimental Validation Reveal the Protective Effect of Paeoniflorin Against Coronary Heart Disease in Mice via Inhibiting the C3-Cfd-C3aR Pathway
Source: Int J Mol Sci. 2026 Jul 13;27(14):6236. doi: 10.3390/ijms27146236 (PMC13410309; doi:10.3390/ijms27146236)
Supplement: Supplementary file 1 [file ijms-27-06236-s001.zip › Supplementary Materials/ijms-4276706_Proteomics_Dataset/8-KEGG_pathway_image/Control-vs-Model/mmu03320.html]

KEGG PATHWAY: PPAR signaling pathway - Mus musculus (house mouse)


# PPAR signaling pathway - Mus musculus (house mouse)


[
Pathway menu
| Organism menu
| Pathway entry
| Show description
| Download
| Help
]

Peroxisome proliferator-activated receptors (PPARs) are nuclear hormone receptors that are activated by fatty acids and their derivatives. PPAR has three subtypes (PPARalpha, beta/delta, and gamma) showing different expression patterns in vertebrates. Each of them is encoded in a separate gene and binds fatty acids and eicosanoids. PPARalpha plays a role in the clearance of circulating or cellular lipids via the regulation of gene expression involved in lipid metabolism in liver and skeletal muscle. PPARbeta/delta is involved in lipid oxidation and cell proliferation. PPARgamma promotes adipocyte differentiation to enhance blood glucose uptake.


##### Option

Scale:


100%

Image resolution:


 High

##### Background color

Organism

##### Search

##### ID search

##### Color


KGML

Image (png) file 1x

Image (png) file 2x
