# Supplementary material for: Multi-Omics and Experimental Validation Reveal the Protective Effect of Paeoniflorin Against Coronary Heart Disease in Mice via Inhibiting the C3-Cfd-C3aR Pathway
Source: Int J Mol Sci. 2026 Jul 13;27(14):6236. doi: 10.3390/ijms27146236 (PMC13410309; doi:10.3390/ijms27146236)
Supplement: Supplementary file 1 [file ijms-27-06236-s001.zip › Supplementary Materials/ijms-4276706_Metabolomics_Dataset/4-Functional Annotation and Enrichment/msea_ind/Model-vs-Paeoniflorin/MSEA Enrichment plot for Arginine and proline metabolism.pdf]

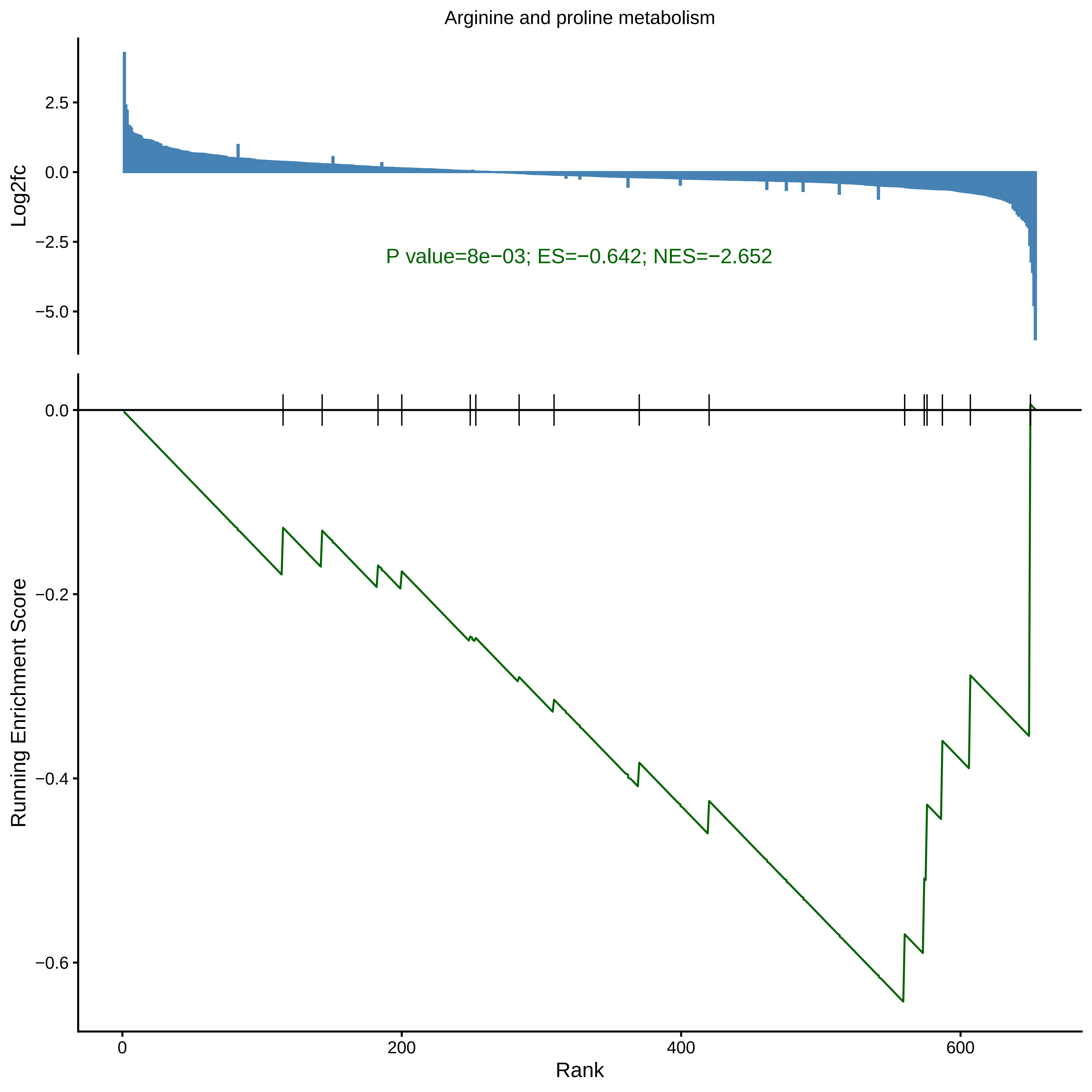
Arginine and proline metabolism
Log2fc
2.5
0.0
−2.5
−5.0
P value=8e−03; ES=−0.642; NES=−2.652
Running Enrichment Score
0.0
−0.2
−0.4
−0.6
0
200
400
600
Rank
