# Supplementary material for: Multi-Omics and Experimental Validation Reveal the Protective Effect of Paeoniflorin Against Coronary Heart Disease in Mice via Inhibiting the C3-Cfd-C3aR Pathway
Source: Int J Mol Sci. 2026 Jul 13;27(14):6236. doi: 10.3390/ijms27146236 (PMC13410309; doi:10.3390/ijms27146236)
Supplement: Supplementary file 1 [file ijms-27-06236-s001.zip › Supplementary Materials/ijms-4276706_Metabolomics_Dataset/4-Functional Annotation and Enrichment/msea_ind/Control-vs-Model/MSEA Enrichment plot for Biosynthesis of unsaturated fatty acids.pdf]

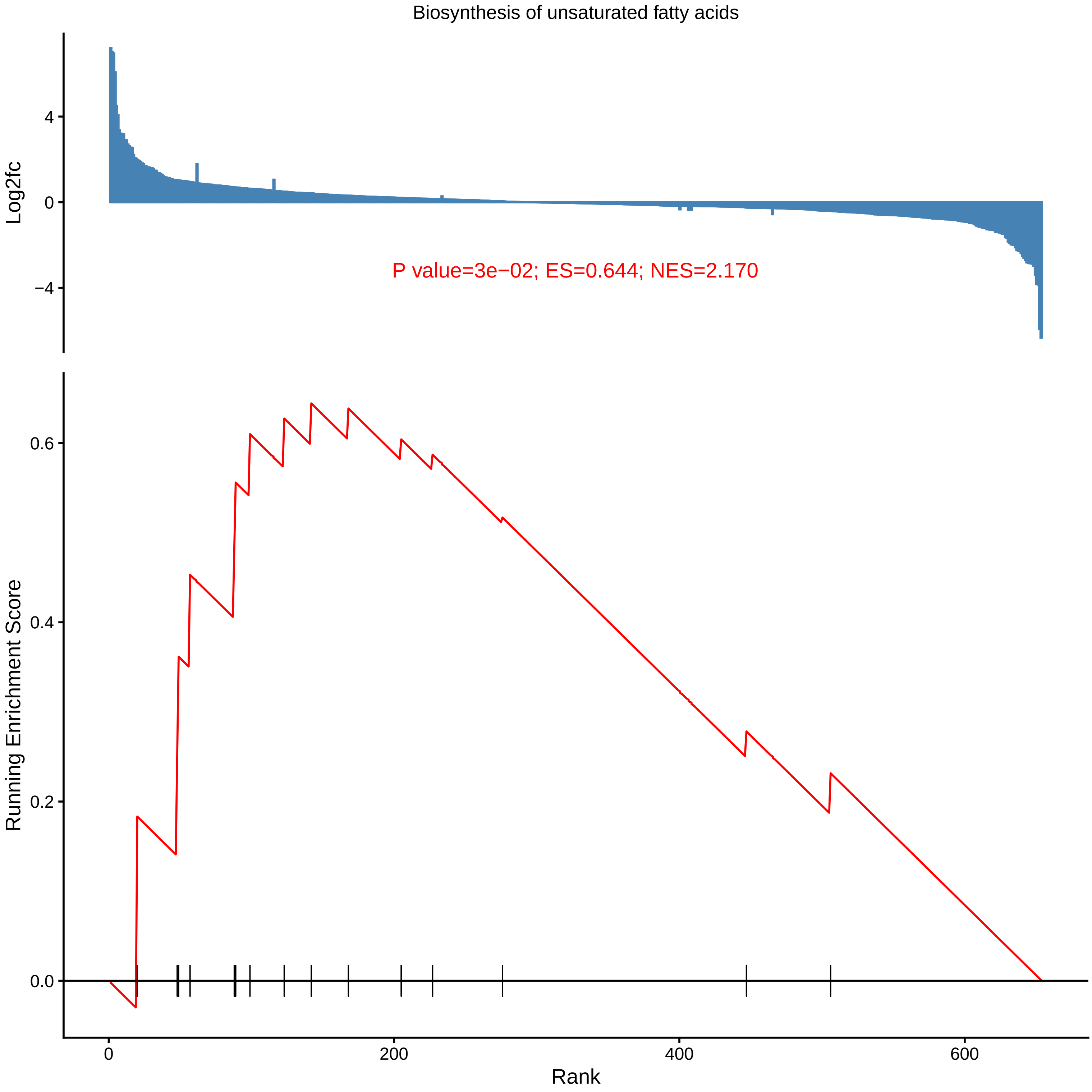

Biosynthesis of unsaturated fatty acids
Log2fc
4
0
−4
P value=3e−02; ES=0.644; NES=2.170
Running Enrichment Score
0.6
0.4
0.2
0.0
0
200
400
600
Rank
